# Comparative safety of anti-epileptic drugs during pregnancy: a systematic review and network meta-analysis of congenital malformations and prenatal outcomes

**DOI:** 10.1186/s12916-017-0845-1

**Published:** 2017-05-05

**Authors:** Areti Angeliki Veroniki, Elise Cogo, Patricia Rios, Sharon E. Straus, Yaron Finkelstein, Ryan Kealey, Emily Reynen, Charlene Soobiah, Kednapa Thavorn, Brian Hutton, Brenda R. Hemmelgarn, Fatemeh Yazdi, Jennifer D’Souza, Heather MacDonald, Andrea C. Tricco

**Affiliations:** 1 Knowledge Translation Program, Li Ka Shing Knowledge Institute, St. Michael’s Hospital, 209 Victoria Street, East Building, Toronto, Ontario M5B 1W8 Canada; 2 Department of Geriatric Medicine, University of Toronto, 27 King’s College Circle, Toronto, Ontario M5S 1A1 Canada; 3 The Hospital for Sick Children, 555 University Avenue, Toronto, Ontario M5G 1X8 Canada; 4 Department of Paediatrics, University of Toronto, 172 St. George Street, Toronto, Ontario M5R 0A3 Canada; 5 Department of Pharmacology and Toxicology, University of Toronto, Medical Sciences Building, Room 4207, 1 King’s College Circle, Toronto, Ontario M5S 1A8 Canada; 6 Institute for Health Policy Management & Evaluation, University of Toronto, 4th Floor, 155 College Street, Toronto, Ontario M5T 3M6 Canada; 7 School of Epidemiology, Public Health and Preventive Medicine, Faculty of Medicine, University of Ottawa, Roger-Guindon Building, 451 Smyth Road, Ottawa, Ontario K1H 8M5 Canada; 8 Clinical Epidemiology Program, Ottawa Hospital Research Institute, The Ottawa Hospital, 501 Smyth Road, Ottawa, Ontario K1H 8L6 Canada; 9 Institute of Clinical and Evaluative Sciences (ICES uOttawa), 1053 Carling Ave, Ottawa, Ontario K1Y 4E9 Canada; 10 Ottawa Hospital Research Institute, Center for Practice Changing Research, The Ottawa Hospital – General Campus, 501 Smyth Road, PO Box 201B, Ottawa, Ontario K1H 8L6 Canada; 11 Departments of Medicine and Community Health Sciences, University of Calgary, TRW Building, 3rd Floor, 3280 Hospital Drive NW, Calgary, Alberta T2N 4Z6 Canada; 12 Epidemiology Division, Dalla Lana School of Public Health, University of Toronto, 6th Floor, 155 College Street, Toronto, Ontario M5T 3M7 Canada

**Keywords:** Network meta-analysis, Systematic review, Epilepsy, Fetus, Pregnancy, Adverse effects, Antiepileptic drugs, Congenital malformations, Miscarriage, Knowledge synthesis

## Abstract

**Background:**

Pregnant women with epilepsy frequently experience seizures related to pregnancy complications and are often prescribed anti-epileptic drugs (AEDs) to manage their symptoms. However, less is known about the comparative safety of AED exposure in utero. We aimed to compare the risk of congenital malformations (CMs) and prenatal outcomes of AEDs in infants/children who were exposed to AEDs in utero through a systematic review and Bayesian random-effects network meta-analysis.

**Methods:**

MEDLINE, EMBASE, and Cochrane CENTRAL were searched from inception to December 15, 2015. Two reviewers independently screened titles/abstracts and full-text papers for experimental and observational studies comparing mono- or poly-therapy AEDs versus control (no AED exposure) or other AEDs, then abstracted data and appraised the risk of bias. The primary outcome was incidence of major CMs, overall and by specific type (cardiac malformations, hypospadias, cleft lip and/or palate, club foot, inguinal hernia, and undescended testes).

**Results:**

After screening 5305 titles and abstracts, 642 potentially relevant full-text articles, and 17 studies from scanning reference lists, 96 studies were eligible (n = 58,461 patients). Across all major CMs, many AEDs were associated with higher risk compared to control. For major CMs, ethosuximide (OR, 3.04; 95% CrI, 1.23–7.07), valproate (OR, 2.93; 95% CrI, 2.36–3.69), topiramate (OR, 1.90; 95% CrI, 1.17–2.97), phenobarbital (OR, 1.83; 95% CrI, 1.35–2.47), phenytoin (OR, 1.67; 95% CrI, 1.30–2.17), carbamazepine (OR, 1.37; 95% CrI, 1.10–1.71), and 11 polytherapies were significantly more harmful than control, but lamotrigine (OR, 0.96; 95% CrI, 0.72–1.25) and levetiracetam (OR, 0.72; 95% CrI, 0.43–1.16) were not.

**Conclusion:**

The newer generation AEDs, lamotrigine and levetiracetam, were not associated with significant increased risks of CMs compared to control, and were significantly less likely to be associated with children experiencing cardiac malformations than control. However, this does not mean that these agents are not harmful to infants/children exposed in utero. Counselling is advised concerning teratogenic risks when the prescription is written for a woman of childbearing age and before women continue with these agents when considering pregnancy, such as switching from polytherapy to monotherapy with evidence of lower risk and avoiding AEDs, such as valproate, that are consistently associated with CMs. These decisions must be balanced against the need for seizure control.

**Systematic Review Registration:**

PROSPERO CRD42014008925

**Electronic supplementary material:**

The online version of this article (doi:10.1186/s12916-017-0845-1) contains supplementary material, which is available to authorized users.

## Background

Epilepsy, the most common chronic neurological condition, affects 0.6–1% of the population [1, 2]. Epilepsy in pregnant women causes frequent seizures, increasing the risk of pregnancy-related complications [3, 4]. Antiepileptic drugs (AEDs) are prescribed to reduce the severity of epilepsy or help manage other conditions such as pain, psychiatric disorders, and migraine [5]. Women taking AEDs have a greater risk of miscarriage and teratogenicity, including a 4–8% chance of giving birth to a child with a major congenital malformation (CM), because these agents can be transferred to the fetus via the placenta [3, 4, 6–8]. Since the first documentation of teratogenicity of AEDs in the 1960s [9, 10], the use of many first-generation AEDs (e.g., valproate) in pregnant women with epilepsy has been studied extensively. Several large-scale pregnancy registries were established to evaluate the safety of first- and newer-generation (e.g., gabapentin) AEDs [11, 12]. However, little is known about the “comparative” safety of AED exposure in utero, and previous studies comparing multiple AEDs are often small and underpowered. As such, we compared the safety of AEDs in infants and children exposed in utero through a systematic review and network meta-analysis (NMA).

## Methods

Our protocol was registered with PROSPERO (CRD42014008925) and published in an open-access journal (Additional file 1) [13]. Our NMA conforms to the ISPOR [14] guidance and PRISMA-NMA (Additional file 2) [15].

### Eligibility criteria

Pregnant women taking AEDs for any indication were eligible. Studies reporting on the following AEDs as monotherapy or polytherapy of any dose were included: first-generation (carbamazepine, clobazam, clonazepam, ethosuximide, phenobarbital, phenytoin, primidone, valproate) and newer-generation (marketed after 1990; gabapentin, lamotrigine, levetiracetam, oxcarbazepine, topiramate, vigabatrin). The comparators were placebo, no AED treatment (women not exposed to AED but with the same indications for their use), or other AEDs alone or in combination. Papers judged to include data from the same patients were excluded from the analysis to avoid double-counting. Companion reports of included studies were used for supplementary information only.

The primary outcomes were the incidence of overall and specific types of major CM, which were defined as malformations present from birth with surgical, medical, functional, or cosmetic importance [16]. When studies also reported on major CM cases that were diagnosed prenatally and resulted in elective terminations, these were included in the CM analysis. For specific CM types, the six most frequently occurring in the literature were selected, namely cardiac, cleft lip/palate, club foot, hypospadias, inguinal hernia, and undescended testes (boys only). The secondary outcomes of interest were the incidence of combined fetal losses, prenatal growth retardation, preterm birth, and minor CMs (i.e., any CM that did not qualify as a major CM; Additional file 3: Appendix A). The “combined” fetal loss types outcome includes total fetal losses reported as well as studies that only report on one type of fetal loss (e.g., stillbirths). Randomized clinical trials (RCTs), quasi-RCTs, and observational studies with a control group examining the effects of AEDs on infants and children (≤12 years of age) who were exposed to AEDs in utero were included. No language or other restrictions were employed.

### Information sources

An experienced librarian developed the search strategies in MEDLINE, EMBASE, and the Cochrane CENTRAL Register of Controlled Trials. The MEDLINE search strategy was peer-reviewed by another librarian using the Peer Review of Electronic Search Strategies checklist [17], and the final version is provided in our protocol [13]. The literature search was initially conducted from inception until March 18, 2014, and a rapid update was conducted on December 15, 2015. Reference lists of all included studies and relevant reviews were scanned. Unpublished studies were sought by locating relevant conference abstracts and contacting authors of included studies and AED manufacturers.

### Study selection and data collection

After the team conducted two pilot-tests of the eligibility criteria among 10 reviewers (12% disagreements), pairs of reviewers screened each title/abstract independently and conflicts (6%) were resolved through discussion. Subsequently, three level 2 screening pilots (26% disagreements) occurred, as well as three data abstraction pilots. The same process was followed for potentially relevant full-text articles (16% conflicts) and data abstraction. Authors were contacted for studies published in the last 10 years to clarify unclear or missing data.

The ‘no AED use’ arms were only included if the control group had the same indication as the active arm in the study (e.g., both had epilepsy). The malformation rates were expressed on a basis of livebirths plus stillbirths, based on the number of pregnant women enrolled in the study.

### Appraisal of methodological quality and risk-of-bias

Two reviewers independently appraised quality using the Cochrane risk-of-bias tool [18] and Newcastle-Ottawa Scale [19]. The comparison-adjusted funnel plot was used to assess publication bias and small-study effects for outcomes including at least 10 studies [20].

In the comparison-adjusted funnel plot, the overall treatment effect for each comparison was estimated under the fixed-effect meta-analysis model and its difference from the study-specific treatment effect versus the study-specific standard error was plotted. All AEDs were ordered from oldest to newest according to their international market approval date. The comparison-adjusted funnel plot does not account for correlations induced by multi-arm trials, which may possibly cause overestimation and mask funnel plot asymmetry. To surmount most correlations in multi-arm trials, only data points corresponding to the study-specific basic parameters (treatment comparisons with common comparator) were plotted. For this, the control group was considered the common comparator or, if this was missing, the oldest treatment comparator was used against the remaining AEDs of the corresponding study.

### Synthesis of included studies

A random-effects meta-analysis model was applied because the studies differed methodologically and clinically. Outcome data were pooled using the odds ratio (OR) and, for two or more studies, the OR was estimated using Bayesian hierarchical models and a Markov Chain Monte Carlo algorithm. When treatment comparisons formed a connected network of evidence, a random-effects NMA was conducted [21] using treatment nodes pre-specified by the team. Multiple doses were combined in nodes, because this information was not reported consistently across the studies. In both pairwise meta-analyses and NMAs, we assumed common within-network between-study variance (*τ* ^2^) across treatment comparisons, since there were many treatment comparisons, including a single study where the (*τ* ^2^) was not estimable.

Prior to applying a NMA, the transitivity assumption was assessed using age, baseline risk, treatment indication, timing of exposure, and risk-of-bias as potential treatment effect modifiers. The mean of each continuous potential effect modifier and the mode (i.e., most frequent value) of each categorical potential effect modifier for each pairwise comparison and outcome were presented in tables [22]. For each outcome, the entire network was evaluated for inconsistency using the design-by-treatment interaction model [23, 24]. The random-effects model was used when multiple studies were available in each design in the network; alternatively, we applied the fixed-effect model. If the global test suggested inconsistency, local inconsistency in specific network paths was assessed using the loop-specific method assuming common within-loop *τ* ^2^ [25, 26]. This was a clinically reasonable assumption, since the treatments were of the same nature. When statistically significant inconsistency or important heterogeneity were detected, the data was checked for errors. If no errors were identified, network meta-regression, subgroup, or sensitivity analyses were conducted. For the overall major CM, combined fetal losses, and prenatal growth outcomes, network meta-regression were performed for age and baseline risk (i.e., using the control group), assuming a common fixed coefficient across comparisons. For these outcomes, a subgroup analysis was conducted for AED generation (i.e., older AEDs versus newer generation AEDs), and study designs (i.e., observational versus RCTs). Sensitivity analyses were conducted on the same outcomes restricting to studies with treatment indication (i.e., including only women with epilepsy), timing of at least first trimester exposure, large study size (i.e., > 300 patients), maternal alcohol intake, and higher methodological quality using two items of the Newcastle-Ottawa Scale for cohort studies (adequacy of follow-up of cohorts, comparability of cohorts) and low overall risk-of-bias for RCTs (component approach using randomization and allocation concealment items) [27]. For the overall major CM outcome, sensitivity analyses were conducted for cohort studies, folic acid used by more than 50% of women and family history of major CMs, including a large international registry study (EURAP) [28, 29] that was not included in the primary analysis due to potential partial overlap of participants with other studies and removing three potentially overlapping studies from Australia, Spain, and Argentina [30–32]. For combined fetal losses and prenatal growth outcomes, sensitivity analysis was conducted for maternal tobacco use. Finally, for the overall major CM, combined fetal losses, and prenatal growth outcomes, the model suggested by Schmitz et al. [33] for different study designs was applied.

In the Schmitz et al. [33] model, bias adjustment to account for over-precision or for over/under-estimation was not introduced, as we were uncertain about the magnitude of bias that might have been introduced from including the observational studies. The goodness-of-fit was measured using the posterior mean of the residual deviance, the degree of between-study heterogeneity, and the deviance information criterion. In a well-fitting model, the posterior mean residual deviance should be close to the number of data points [34, 35]. A difference of three units in the deviance information criterion was considered important and the lowest value of the deviance information criterion corresponded to the model with the best fit [34, 35].

The safety of AED medications was ranked using the surface under the cumulative ranking (SUCRA) curve [36]. The larger the SUCRA value for a treatment, the higher its safety rank among all the available treatment options. Ideally, we would like to observe a steep gradient in the SUCRA curve suggesting that the corresponding treatment is most likely the safest. SUCRA curves are presented along with 95% CrIs. A rank-heat plot was used to depict the SUCRA values for all outcomes (http://rh.ktss.ca/) [37].

Meta-analyses and NMAs were performed within OpenBUGS [38], assuming non-informative priors for all model parameters and a half-normal prior distribution for the between-study standard deviation (*τ* ~ *N*(0,1), *τ* > 0). The models were run for 100,000 iterations to ensure model convergence, which was checked by visual inspection of the mixing of two chains, after discarding the first 10,000 iterations and thinning of 10. These samples were used to calculate the median and 95% credible intervals (CrI) for each parameter value. Medians were presented instead of means, since means may be overly influenced by outliers. The design-by-treatment interaction model was performed in Stata using the *network* command [39]. The meta-analysis and NMA ORs were presented with 95% CrIs for each pair of treatments. For the NMA effect estimates, a 95% predictive interval (PrI) was also presented, capturing the magnitude of *τ* ^2^ and presenting the interval within which we would expect the treatment effect of a future study to lie [40, 41].

In the following sections, the terms ‘safer’ and ‘harmful’ are used to indicate when a treatment is associated with a lower risk (safer) or greater risk (harmful) of experiencing an adverse outcome compared to the alternative (e.g., another AED or control).

## Results

### Literature search

**Fig. 1:**
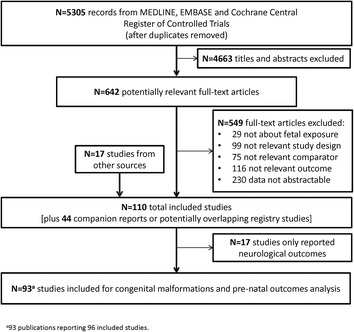
Study flow

After screening 5305 titles and abstracts, 642 potentially relevant full-text articles, and 17 additional studies identified from scanning reference lists, 154 publications describing 110 different studies were included (Fig. 1). Of the included 110 studies, nine were written in languages other than English and three were conference abstracts or letters to the editor with usable data. Scanning of reference lists of included articles and related reviews identified 13 additional studies. Overall, 48% (22/46) of contacted authors responded to our query but only 17% (8/46) were able to provide additional data for our analysis. Further, 29% (13/45) of authors of conference abstracts responded to our query but none were able to provide unpublished data for our analysis. We were unable to contact 11 authors due to non-working email addresses. One author provided a manuscript and four authors provided unpublished data that were included in the analysis.

Seventeen of the eligible studies reported neurological outcomes that were excluded in this paper and reported in another paper (personal communication with Dr. Veroniki), leaving 96 studies with 58,461 patients (reported in 93 articles) included for analysis (Additional file 3: Appendix B). A table of key studies excluded due to reporting only one treatment arm with abstractable data is provided in Additional file 3: Appendix C.

### Study and patient characteristics

We included 92 cohort studies, three case-control studies, and one RCT (Table 1, Additional file 3: Appendices D and E) published between 1964 and 2015. The number of patients included per study ranged from 18 to 7759. The most common study indication was epilepsy (93%), and almost half of the studies (49%) included unmedicated women with epilepsy as a control group. The mean maternal age ranged from 24 to 34 years. Most studies (58%) were conducted in Europe, followed by North America (19%).

**Table 1:** Summary characteristics of included studies

Characteristic	Number of studies (*n* = 96)	Percentage of total
Year of publication		
1964–1980	7	7.29
1981–1990	19	19.79
1991–2000	22	22.92
2001–2005	8	8.33
2006–2010	12	12.50
2011–2015	28	29.17
Continent		
Europe	56	58.33
North America	18	18.75
Asia	10	10.42
Trans-Continental	5	5.21
Australia	3	3.13
South America	3	3.13
Africa	1	1.04
Study design		
Observational cohort	92	95.83
Case-control	3	3.13
Randomized clinical trial	1	1.04
Registry study		
Yes	30	31.25
No	66	68.75
Sample size		
18–50	16	16.67
51–100	26	27.08
101–300	32	33.33
301–500	8	8.33
501–1000	2	2.08
1001–7759	12	12.50
Number of interventions^a^		
2–4	41	42.71
5–7	30	31.25
8–10	15	15.63
11–17	10	10.42
Funding		
Public	21	21.88
Private	7	7.29
Mixed public and private	16	16.67
Not reported	52	54.17
Indication		
Epilepsy	89	92.71
Mixed indications	1	1.04
Mental illness	1	1.04
Not reported	5	5.21
Epileptic control group		
Yes	47	48.96
No/not reported/not applicable	49	51.04
Mean maternal age, years		
24–26	11	11.46
27–29	23	23.96
30–34	7	7.29
Not reported	55	57.29
Anti-epileptic drug exposure timing		
At least 1st trimester	64	66.67
No/not reported	32	33.33
Folic acid use		
Reported	13	13.54
Not reported	83	86.46
Alcohol use		
Reported	5	5.21
Not reported	91	94.79
Tobacco use		
Reported	10	10.42
Not reported	86	89.58

^a^Including any relevant control group

### Methodological quality/risk-of-bias

The RCT was appraised with the Cochrane risk-of-bias tool and had an unclear risk-of-bias for reporting bias and ‘other’ bias (i.e., funding bias), as well as a high risk-of-bias for random sequence generation and allocation concealment (Additional file 3: Appendix F). Three case-control studies and 92 cohort studies were assessed with the Newcastle-Ottawa Scale. The case-control studies had high methodological quality on all items except for the comparability of cohorts on the basis of the design/analysis (Additional file 3: Appendix G). Methodological shortcomings in the cohort studies (Additional file 3: Appendix H) included not controlling for confounders (81%) or reporting number of patients lost to follow-up (59%). The comparison-adjusted funnel plots showed no evidence for publication bias and small-study effects across all outcomes (Additional file 3: Appendix I).

### Statistical analysis

The transitivity assumption was upheld for mean age, mean baseline risk, treatment indication, and timing (Additional file 3: Appendix J). However, the adequacy of follow-up and comparability of cohort items varied across treatment comparisons. The design-by-treatment interaction model suggested that there was no evidence of statistically significant inconsistency for all outcomes and additional analyses (Additional file 3: Appendix J).

**Fig. 2:**
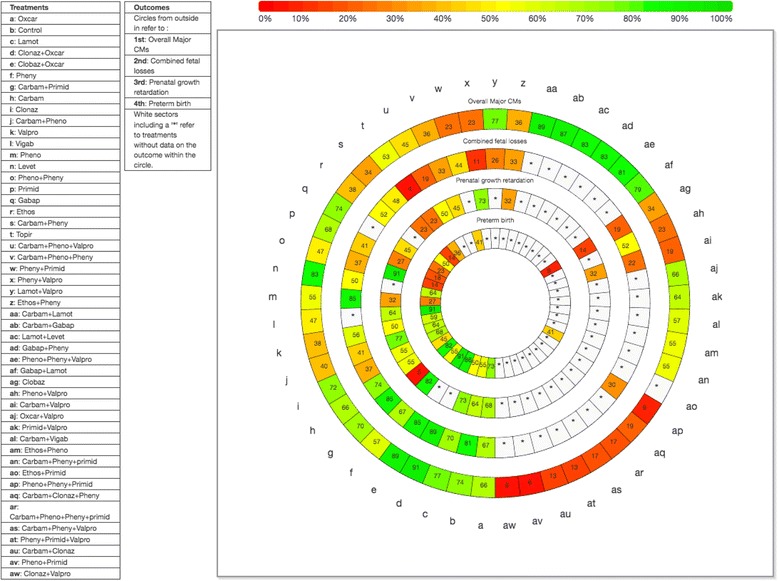
Rank heat plot for overall major congenital malformations (CMs), combined fetal losses, prenatal growth retardation, and preterm birth. Rank-heat plot of 49 treatments (presented in 49 radii) and four outcomes (presented in four concentric circles). Each sector is colored according to the SUCRA value of the corresponding treatment and outcome using the transformation of three colors: red (0%), yellow (50%), and green (100%). *carbam* carbamazepine, *clobaz* clobazam, *clonaz* clonazepam, *ethos* ethosuximide, *gabap* gabapentin, *lamot* lamotrigine, *levet* levetiracetam, *oxcar* oxcarbazepine, *pheno* phenobarbital, *pheny* phenytoin, *primid* primidone, *topir* topiramate, *valpro* valproate, *vigab* vigabatrin

In the following sections, the overall NMA, meta-regression, subgroup, and sensitivity analyses results for each outcome are discussed; the SUCRA curve results are presented in Fig. 2 and Additional file 3: Appendix K. Furthermore, AED sample sizes and absolute risks for each AED can be found in Additional file 3: Appendix K.

### Overall major CMs

**Fig. 3:**
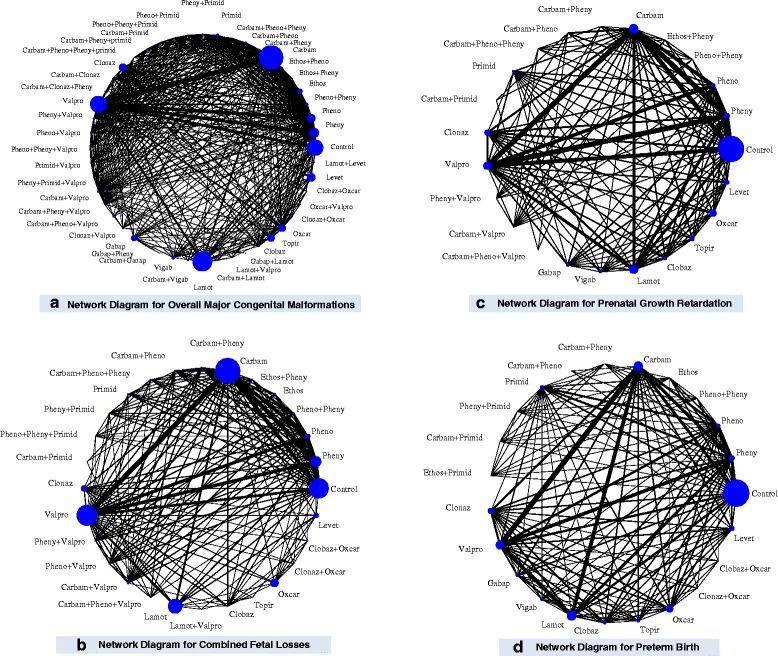
Network plots for overall major congenital malformations, combined fetal losses, prenatal growth retardation, and preterm birth. Each treatment node is weighted according to the number of patients that have received the particular treatment, and each edge is weighted according to the number of studies comparing the treatments it connects. *carbam* carbamazepine, *clobaz* clobazam, *clonaz* clonazepam, *ethos* ethosuximide, *gabap* gabapentin, *lamot* lamotrigine, *levet* levetiracetam, *oxcar* oxcarbazepine, *pheno* phenobarbital, *pheny* phenytoin, *primid* primidone, *topir* topiramate, *valpro* valproate, *vigab* vigabatrin

**Fig. 4:**
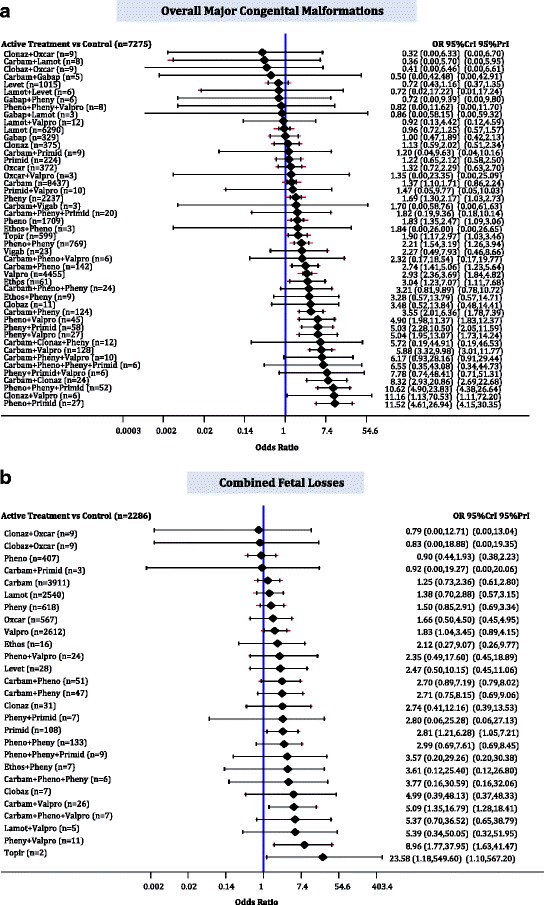
Network meta-analysis forest plots for each treatment versus control. Each rhombus represents the summary treatment effect estimated in the network meta-analysis on the odds ratio (OR) scale. The black horizontal lines represent the credible intervals (CrI) for the summary treatment effects, and the red horizontal lines represent the corresponding predictive intervals (PrI). In the absence of heterogeneity, the CrIs and PrIs should be identical. An OR > 1 suggests that control is safer, whereas an OR < 1 suggests that the comparator active treatment is safer. The vertical blue line corresponds to an OR = 1 (i.e., the treatment groups compared are equally safe). The total sample size (n) included in each treatment is also presented. **a** Overall major congenital malformations (78 studies, 35,016 cases, 48 treatments). **b** Combined fetal losses (31 studies, 13,487 cases, 28 treatments). *carbam* carbamazepine, *clobaz* clobazam, *clonaz* clonazepam, *ethos* ethosuximide, *gabap* gabapentin, *lamot* lamotrigine, *levet* levetiracetam, *oxcar* oxcarbazepine, *pheno* phenobarbital, *pheny* phenytoin, *primid* primidone, *topir* topiramate, *valpro* valproate, *vigab* vigabatrin

The median baseline risk of major CM in the control group (no AED exposure) across all studies was 0.026 (interquartile range, 0.000–0.092; Additional file 3: Appendix K). The NMA on overall major CMs included 75 cohort studies, two case-control studies and one RCT, 35,016 cases, 47 AEDs plus control, with 15% of all pairwise comparisons reaching statistical significance (Fig. 3a Additional file 3: Appendices J and L). The following monotherapies were associated with statistically significantly more cases developing major CMs than control: ethosuximide (OR, 3.04; 95% CrI, 1.23–7.07), valproate (OR, 2.93; 95% CrI, 2.36–3.69), topiramate (OR, 1.90; 95% CrI, 1.17–2.97), phenobarbital (OR, 1.83; 95% CrI, 1.35–2.47), phenytoin (OR, 1.67; 95% CrI, 1.30–2.17), and carbamazepine (OR, 1.37; 95% CrI, 1.10–1.71) (Fig. 4a). Gabapentin (OR, 1.00; 95% CrI, 0.47–1.89), lamotrigine (OR, 0.96; 95% CrI, 0.72–1.25), levetiracetam (OR, 0.72; 95% CrI, 0.43–1.16), and nine polytherapies lacked sufficient evidence to reach statistical significance (Fig. 4a).

The results in subgroup NMA when restricting to observational studies only (2 case-control and 75 cohort studies, 34,966 cases, 48 treatments; *τ* ^2^ = 0.03; 95% CrI, 0.00–0.13) were in agreement with NMA. The sensitivity analysis restricting to cohort studies (75 studies, 34,667 cases, 48 treatments; *τ* ^2^ = 0.02; 95% CrI, 0.00–0.11) found comparable results with NMA, but clonazepam plus valproate was marginally not statistically significant (OR, 12.780; 95% CrI, 0.974–68.810). Similar results were also observed with the Schmitz model (1 RCT, 2 case-control, and 75 cohort studies, 35,016 cases, 48 treatments; *τ* ^2^ = 0.30; 95% CrI, 0.00–3.95), but carbamazepine versus control was not statistically significant (OR, 1.34; 95% CrI, 0.27–5.02) similar to the results obtained from the RCT (1 study, 50 cases, 3 treatments).

Similar results to the NMA were found with the sensitivity analysis including the EURAP study (1 RCT, 2 case-control, and 73 cohort studies, 48 treatments, 38,151 cases; *τ* ^2^ = 0.04; 95% CrI, 0.00–0.13), where control had statistically significantly lower risk of major CM than valproate combined with carbamazepine and phenytoin (OR, 6.14; 95% CrI, 1.06–29.14) or with lamotrigine (OR, 2.94; 95% CrI, 1.61–5.05), but did not have a significantly lower risk of major CM than ethosuximide (OR, 3.13; 95% CrI, 0.77–6.59). The Schmitz model for the sensitivity analysis including the EURAP (1 RCT, 2 case-control, and 73 cohort studies, 38,151 cases, 48 treatments; *τ* ^2^ = 0.31; 95% CrI, 0.00–3.58) suggested a statistically significant OR for the comparison lamotrigine plus valproate versus control (OR, 3.01; 95% CrI, 1.60–5.27), whereas clonazepam plus valproate (OR, 11.17; 95% CrI, 0.77–66.36) and carbamazepine (OR, 1.32; 95% CrI, 0.26–4.64) did not statistically significantly differ from control.

The sensitivity analysis results for timing of first trimester exposure to AED (1 RCT and 49 cohort studies, 25,329 cases, 46 treatments; *τ* ^2^ = 0.04; 95% CrI, 0.00–0.17) for treatment indication of epilepsy (1 RCT, 2 case-control, and 68 cohort studies, 30,289 cases, 47 treatments; *τ* ^2^ = 0.03; 95% CrI, 0.00–0.13) and for older AEDs (i.e., without control, gabapentin, lamotrigine, levetiracetam, oxcarbazepine, topiramate, and vigabatrin; 1 RCT, 2 case-control, and 50 cohort studies, 6982 cases, 31 treatments; *τ* ^2^ = 0.08; 95% CrI, 0.00–0.27) overall agreed with NMA. However, in timing, the polytherapy carbamazepine plus phenytoin plus valproate was associated with statistically significantly more cases developing major CMs than control (OR, 8.00; 95% CrI, 1.02–32.61), whereas clonazepam plus valproate (OR, 13.34; 95% CrI, 0.21–90.51) and ethosuximide (OR, 2.80; 95% CrI, 0.93–6.52) did not statistically differ from control.

Five cohort studies of 5212 women with a history of alcohol comparing 16 treatments (*τ* ^2^ = 0.20; 95% CrI, 0.00–1.49) and two cohort studies comparing 11 treatments in 5057 women reported a family history of CMs (*τ* ^2^ = 0.23; 95% CrI, 0.00–3.42), suggesting that no AED was statistically significantly different than control. Another 5 cohort studies that reported folic acid use in more than 50% of the 10,825 included women compared 15 treatments and showed that valproate was statistically significantly more harmful than control (OR, 2.86; 95% CrI, 1.18–6.22; *τ* ^2^ = 0.09; 95% CrI, 0.00–0.72).

To assess the impact of small studies, we conducted a NMA restricted to studies including more than 300 cases. We included 13 cohort studies, 27,227 cases, and 22 treatments (*τ* ^2^ = 0.03; 95% CrI, 0.00–0.17), and the sensitivity analysis suggested that carbamazepine plus phenytoin plus valproate was associated with statistically significantly more cases developing major CMs compared to control (OR, 20.77; 95% CrI, 1.72–154.20), whereas clonazepam plus valproate (OR, 11.65; 95% CrI, 0.82–71.86) did not statistically differ from the control. The sensitivity analysis for low risk-of-bias in the comparability of cohorts item on the Newcastle-Ottawa Scale, including 10 observational studies, 21,622 cases, and 31 treatments (*τ* ^2^ = 0.03; 95% CrI, 0.00–0.21), suggested that only phenobarbital (OR, 2.22; 95% CrI, 1.12–4.08), topiramate (OR, 1.89; 95% CrI, 1.10–3.24), and valproate (OR, 2.77; 95% CrI, 1.92–4.09) were statistically significantly different from the control. When restricting to low risk-of-bias for the adequacy of follow-up of cohorts (k = 35, n = 20,122; *τ* ^2^ = 0.05; 95% CrI, 0.00–0.22), phenytoin plus primidone (OR, 2.58; 95% CrI, 0.46–9.77), phenytoin plus valproate (OR, 1.90; 95% CrI, 0.23–8.94), and topiramate (OR, 1.59; 95% CrI, 0.63–3.40) were no longer statistically significantly different from zero.

Accounting for baseline risk in a network meta-regression model resulted in a statistically non-significant association with the treatment effect (1 RCT, 2 case-control, and 75 cohort studies, 35,016 cases, 48 treatments, estimated regression coefficient on OR scale, 1.02; 95% CrI, 0.93–1.10; *τ* ^2^ = 0.03; 95% CrI, 0.00–0.14; residual deviance = 411, data points = 468, deviance information criterion = 562). Similarly, a statistically significant association was not observed in our network meta-regression analysis conducted using age as a covariate (32 cohort studies, 15,948 cases, 43 treatments, estimated regression coefficient on OR scale, 0.99; 95% CrI, 0.85–1.15; *τ* ^2^ = 0.03; 95% CrI, 0.00–0.16; residual deviance = 180, data points = 213, deviance information criterion = 267). For more details on the subgroup, meta-regression, and sensitivity analyses see Additional file 3: Appendix M).

### Combined fetal losses

The median baseline risk of combined fetal losses in the control group (no AED exposure) across all studies was 0.000 (interquartile range: 0.000–0.000; Additional file 3: Appendix K). The NMA for combined fetal losses included 1 RCT, 1 case-control study, and 29 cohort studies, 13,487 pregnancies, and 27 AEDs plus control, with 5% of comparisons reaching statistical significance (Fig. 3b; Additional file 3: Appendices A, J and L). Topiramate (OR, 23.58; 95% CrI, 1.18–549.60), primidone (OR, 2.81; 95% CrI, 1.21–6.28), valproate (OR, 1.83; 95% CrI, 1.04–3.45), and two polytherapies (carbamazepine plus valproate: OR, 5.09; 95% CrI, 1.35–16.79; phenytoin plus valproate: OR, 8.96; 95% CrI, 1.77–37.95) were associated with statistically significantly more combined fetal losses than control (Fig. 4b).

Similar results with the NMA analyses were observed in subgroup analysis including observational studies only (1 case-control and 29 cohort studies, 13,437 pregnancies; *τ* ^2^ = 0.03; 95% CrI, 0.00–0.26) and in the Schmitz model (1 RCT, 1 case-control study, and 29 cohort studies, 13,487 pregnancies; *τ* ^2^ = 0.36; 95% CrI, 0.00–4.17), where control was additionally associated with a marginally statistically significantly lower risk of fetal loses than the combination phenobarbital and phenytoin (OR, 3.04; 95% CrI, 1.07–7.18), except for topiramate (OR, 13.06; 95% CrI, 0.77–365.50). The sensitivity analysis results for timing of at least first trimester exposure to AED (1 case-control and 16 cohort studies, 6970 pregnancies; *τ* ^2^ = 0.04; 95% CrI, 0.00–0.17) were in agreement with NMA, and the only statistically significant results of all treatments versus control were for carbamazepine combined with valproate (OR, 7.83; 95% CrI, 1.62–32.08) or phenobarbital (OR, 4.73; 95% CrI, 1.24–17.24), with control statistically significantly safer. Two cohort studies with 318 women with a history of alcohol use during pregnancy compared 10 treatments (*τ* ^2^ = 0.31; 95% CrI, 0.00–3.87) and another 3 cohort studies with 4666 women with a smoking history compared 14 treatments (*τ* ^2^ = 0.14; 95% CrI, 0.00–2.19), and showed that only phenytoin plus valproate was statistically significantly different than control (alcohol use: OR, 269.30; 95% CrI, 2.42–1.19 × 10^6^, smoking history: OR, 180.30; 95% CrI, 6.10–4.17 × 10^5^). The restriction to studies comparing only older AEDs (1 RCT, 1 case-control, and 20 cohort studies, 3054 neonates; *τ* ^2^ = 0.06; 95% CrI, 0.00–0.49) suggested that control was associated with a marginally statistically significantly lower risk of fetal loses than phenobarbital plus phenytoin (OR, 2.93; 95% CrI, 1.04–7.73), whereas valproate (OR, 1.76; 95% CrI, 0.86–3.82) was no longer statistically significantly different than control.

The sensitivity analyses restricting to (1) studies with more than 300 pregnancies (4 cohort studies, 10,224 women, 10 treatments; *τ* ^2^ = 0.25; 95% CrI, 0.00–2.05), (2) low risk-of-bias in the “comparability of cohorts” item on the Newcastle-Ottawa Scale (2 cohort studies, 5539 women, 4 treatments; *τ* ^2^ = 0.75; 95% CrI, 0.00–5.42), and (3) low risk-of-bias for the “adequacy of follow-up of cohorts” item (15 cohort studies, 6236 women, 23 treatments; *τ* ^2^ = 0.07; 95% CrI, 0.00–0.61) suggested no AED differed statistically significantly from the control. The network meta-regression analyses using baseline risk (1 RCT, 1 case-control study, and 29 cohort studies, 13,487 pregnancies, 28 treatments, estimated regression coefficient on OR scale, 1.00; 95% CrI, 0.94–1.08; *τ* ^2^ = 0.05; 95% CrI, 0.00–0.31; residual deviance = 130, data points = 175, deviance information criterion = 199) and age (1 case-control study, 14 cohort studies, 7152 pregnancies, 22 treatments, estimated regression coefficient on OR scale, 0.92; 95% CrI, 0.67–1.33; *τ* ^2^ = 0.09; 95% CrI, 0.00–0.58; residual deviance = 74, data points = 96, deviance information criterion = 118) as covariates suggested no statistically significant associations with the treatment effect (Additional file 3: Appendix M).

### Prenatal growth retardation

**Fig. 5:**
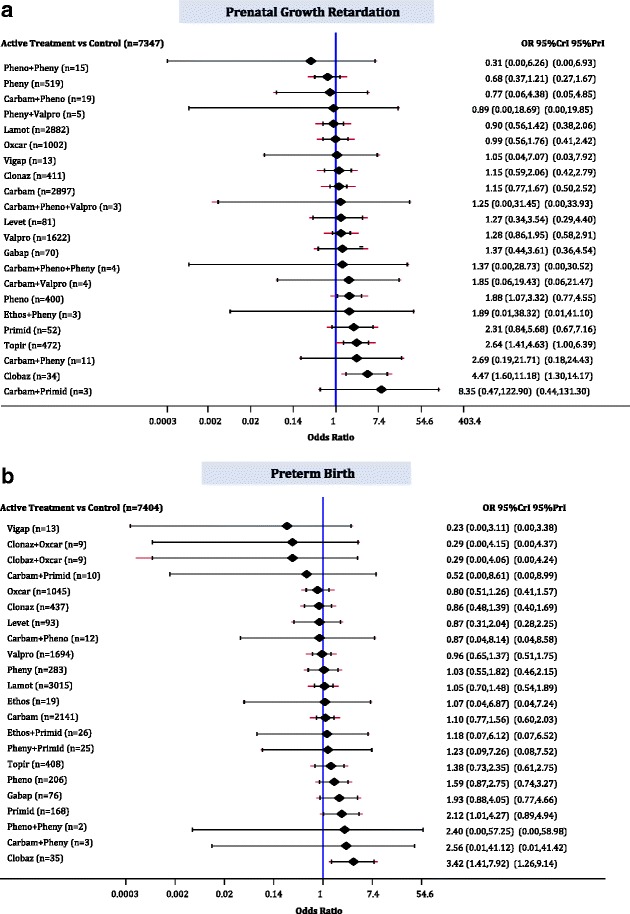
Network meta-analysis forest plots for each treatment versus control. Each rhombus represents the summary treatment effect estimated in the network meta-analysis on the odds ratio (OR) scale. The black horizontal lines represent the credible intervals (CrI) for the summary treatment effects, and the red horizontal lines represent the corresponding predictive intervals (PrI). In the absence of heterogeneity, the CrIs and PrIs should be identical. An OR > 1 suggests that control is safer, whereas an OR < 1 suggests that the comparator active treatment is safer. The vertical blue line corresponds to an OR = 1 (i.e., the treatment groups compared are equally safe). The total sample size (n) included in each treatment is also presented. **a** Prenatal growth retardation (16 studies, 18,177 cases, 23 treatments). **b** Preterm birth (17 studies, 17,133 cases, 23 treatments). *carbam* carbamazepine, *clobaz* clobazam, *clonaz* clonazepam, *ethos* ethosuximide, *gabap* gabapentin, *lamot* lamotrigine, *levet* levetiracetam, *oxcar* oxcarbazepine, *pheno* phenobarbital, *pheny* phenytoin, *primid* primidone, *topir* topiramate, *valpro* valproate, *vigab* vigabatrin

The median baseline risk of prenatal growth retardation in the control group (no AED exposure) across all studies was 0.047 (interquartile range, 0.024–0.100; Additional file 3: Appendix K). The NMA for prenatal growth retardation included 16 cohort studies, 18,117 children, 22 AEDs plus control, with 8% of comparisons reaching statistical significance (Fig. 3c; Additional file 3: Appendices A, J and L). Clobazam (OR, 4.47; 95% CrI, 1.60–11.18), topiramate (OR, 2.64; 95% CrI, 1.41–4.63), and phenobarbital (OR, 1.88; 95% CrI, 1.07–3.32) were associated with statistically significantly more children experiencing prenatal growth retardation than control (Fig. 5a).

The sensitivity analysis results for timing of at least first trimester exposure to AED (6 cohorts, 16,263 children, 14 treatments; *τ* ^2^ = 0.09; 95% CrI, 0.00–0.55) and for treatment indication of epilepsy (15 cohorts, 18,099 children, 23 treatments; *τ* ^2^ = 0.10; 95% CrI, 0.00–0.37) were in agreement with the NMA, where control was not significantly safer than phenobarbital (timing: OR, 1.85; 95% CrI, 0.92–3.97; epilepsy: OR, 1.79; 95% CrI, 1.00–3.10). However, control was associated with a statistically significant lower risk of prenatal growth than carbamazepine for first trimester exposure (OR, 1.51; 95% CrI, 1.01–2.46). The subgroup NMA for different AED generations showed that no AED was statistically significantly different from control, whereas the safest agent when comparing the newer AEDs (topiramate and lamotrigine) was lamotrigine (1 cohort study, 1928 children, 2 treatments; OR, 3.03; 95% CrI, 2.13–4.17). One cohort study with 308 women with a history of alcohol use showed that lamotrigine was statistically significantly better than carbamazepine (OR, 0.29; 95% CrI, 0.09–0.93) and valproate (OR, 0.25; 95% CrI, 0.07–0.85), but not significantly safer than phenytoin (OR, 0.89; 95% CrI, 0.16–5.00). Six cohort studies with 16,263 women with a smoking history compared 14 treatments (*τ* ^2^ = 0.09; 95% CrI, 0.00–0.55) and suggested that only clobazam (OR, 4.07; 95% CrI, 1.24–11.61) and topiramate (OR, 2.79; 95% CrI, 1.43–5.25) were associated with statistically significantly more children experiencing prenatal growth retardation than control.

The restriction to large studies (>300 patients) included 7 cohort studies, 16,899 children, and 14 treatments (*τ* ^2^ = 0.12; 95% CrI, 0.01–0.51) suggesting that only clobazam (OR, 3.73; 95% CrI, 1.11–11.26) was associated with statistically significantly more children experiencing prenatal growth retardation than control. The sensitivity analysis for low risk-of-bias in the “comparability of cohorts” item, including 7 cohort studies, 16,502 children, and 15 treatments (*τ* ^2^ = 0.12; 95% CrI 0.00–0.57), suggested that no AED differed statistically significantly from control. When restricting to low risk-of-bias for the “adequacy of follow-up of cohorts” item (11 cohort studies, 15,200 children, 23 treatments; *τ* ^2^ = 0.10; 95% CrI, 0.00–0.46) clobazam (OR, 4.09; 95% CrI, 1.26–11.82) and topiramate (OR, 2.88; 95% CrI, 1.34–5.88) were associated with statistically significantly more children experiencing prenatal growth retardation than control.

A network meta-regression analysis using baseline risk as a covariate was conducted and a statistically significant association with the treatment effect was not detected, despite a slight drop in the between-study variance (16 cohort studies, 18,117 children, 23 treatments, estimated regression coefficient on OR scale, 0.82; 95% CrI, 0.67–1.00; *τ* ^2^ = 0.05; 95% CrI, 0.00–0.30; residual deviance = 87, data points = 89, deviance information criterion = 135, Additional file 3: Appendix M).

### Preterm birth

The median baseline risk of preterm birth in the control group (no AED exposure) across all studies was 0.051 (interquartile range, 0.025–0.072; Additional file 3: Appendix K). The NMA on preterm birth included 17 cohort studies, 17,133 neonates, and 22 AEDs plus control, with 5% of comparisons reaching statistical significance (Fig. 3d, Additional file 3: Appendices A, J and L). Clobazam (OR, 3.42; 95% CrI, 1.41–7.92) and primidone (OR, 2.12; 95% CrI, 1.01–4.27) were associated with statistically significantly more preterm births than control (Fig. 5b).

### Cardiac malformations

**Fig. 6:**
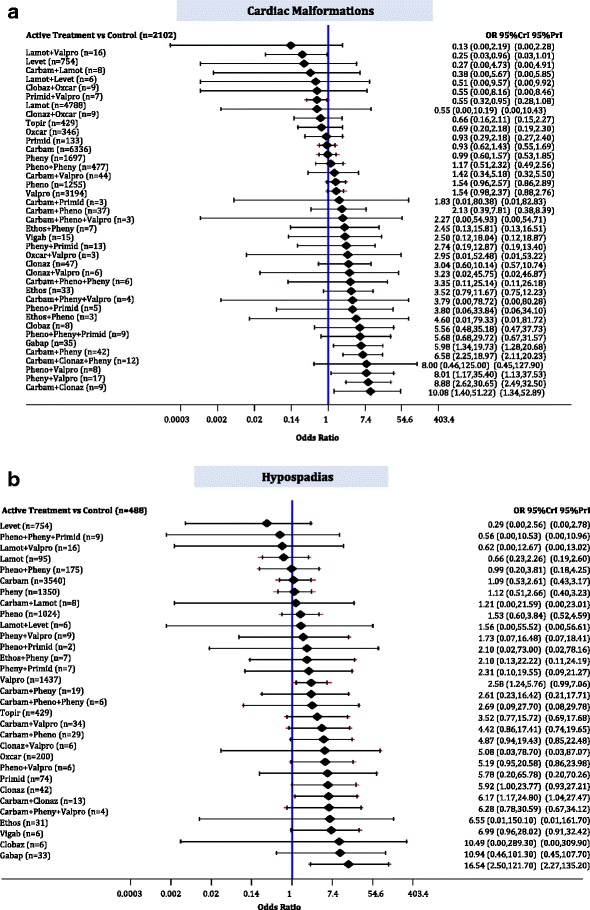
Network meta-analysis forest plots for each treatment versus control. Each rhombus represents the summary treatment effect estimated in the network meta-analysis on the odds ratio (OR) scale. The black horizontal lines represent the credible intervals (CrI) for the summary treatment effects, and the red horizontal lines represent the corresponding predictive intervals (PrI). In the absence of heterogeneity, the CrIs and PrIs should be identical. An OR > 1 suggests that control is safer, whereas an OR < 1 suggests that the comparator active treatment is safer. The vertical blue line corresponds to an OR = 1 (i.e., the treatment groups compared are equally safe). The total sample size (n) included in each treatment is also presented. **a** Cardiac malformations (51 studies, 21,935 cases, 40 treatments). **b** Hypospadias (31 studies, 12,365 cases, 32 treatments). *carbam* carbamazepine, *clobaz* clobazam, *clonaz* clonazepam, *ethos* ethosuximide, *gabap* gabapentin, *lamot* lamotrigine, *levet* levetiracetam, *oxcar* oxcarbazepine, *pheno* phenobarbital, *pheny* phenytoin, *primid* primidone, *topir* topiramate, *valpro* valproate, *vigab* vigabatrin

The median baseline risk of cardiac malformations in the control group (no AED exposure) across all studies was 0.000 (interquartile range, 0.000–0.027; Additional file 3: Appendix K). The NMA on cardiac malformations included 1 RCT, 1 case-control, and 49 cohort studies, 21,935 cases, 39 AEDs plus control, with 11% of comparisons reaching statistical significance (Additional file 3: Appendices J, L, and N). Levetiracetam (OR, 0.25; 95% CrI, 0.03–0.96) and lamotrigine (OR, 0.55; 95% CrI, 0.32–0.95) were monotherapies statistically significantly less likely to be associated with cases experiencing cardiac malformations than control. In contrast, gabapentin (OR, 5.98; 95% CrI, 1.37–19.73), carbamazepine plus phenytoin (OR, 6.58; 95% CrI, 2.25–18.97), phenobarbital plus valproate (OR, 8.01; 95% CrI, 1.17–35.40), phenytoin plus valproate (OR, 8.88; 95% CrI, 2.62–30.65), and carbamazepine plus clonazepam (OR, 10.08; 95% CrI, 1.40–51.22) were associated with statistically significantly more cases developing cardiac malformations compared to control (Fig. 6a).

### Hypospadias

The median baseline risk of hypospadias in the control group (no AED exposure) across all studies was 0.000 (interquartile range, 0.000–0.015; Additional file 3: Appendix K). The NMA for hypospadias included 1 RCT, 1 case-control, and 29 cohort studies, 12,365 cases, and 31 AEDs plus control, with 7% of comparisons reaching statistical significance (Additional file 3: Appendices J, L, and N). Gabapentin (OR, 16.54; 95% CrI, 2.50–121.70), clonazepam (OR, 6.17; 95% CrI, 1.17–24.80), primidone (OR, 5.92; 95% CrI, 1.01–23.77), and valproate (OR, 2.58; 95% CrI, 1.24–5.76) were associated with statistically significantly more cases developing hypospadias compared to control (Fig. 6b).

### Cleft lip/palate

**Fig. 7:**
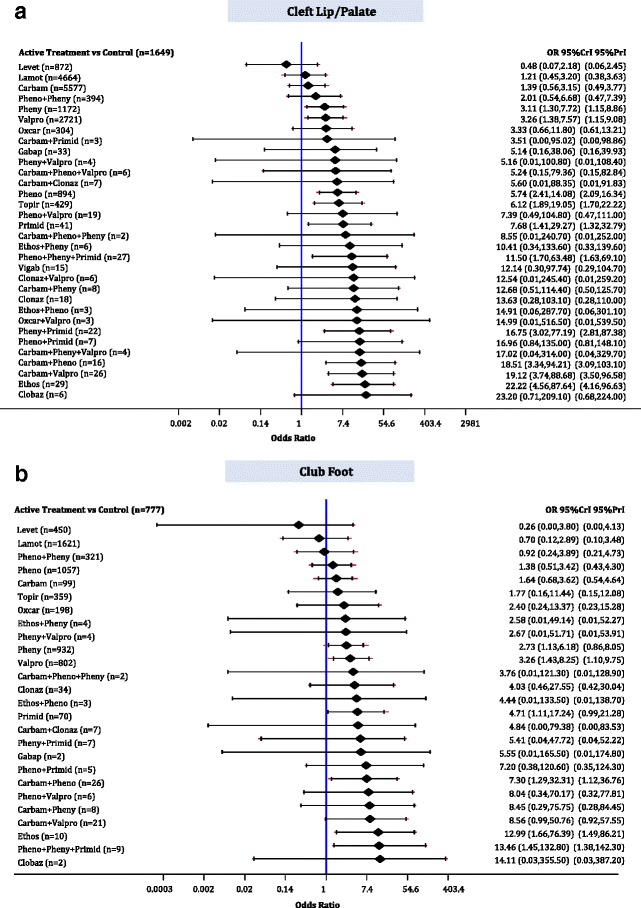
Network meta-analysis forest plots for each treatment versus control. Each rhombus represents the summary treatment effect estimated in the network meta-analysis on the odds ratio (OR) scale. The black horizontal lines represent the credible intervals (CrI) for the summary treatment effects, and the red horizontal lines represent the corresponding predictive intervals (PrI). In the absence of heterogeneity, the CrIs and PrIs should be identical. An OR > 1 suggests that control is safer, whereas an OR < 1 suggests that the comparator active treatment is safer. The vertical blue line corresponds to an OR = 1 (i.e., the treatment groups compared are equally safe). The total sample size (n) included in each treatment is also presented. **a** Cleft lip/palate (29 studies, 18,987 cases, 33 treatments). **b** Club foot (23 studies, 8836 cases 27 treatments). *carbam* carbamazepine, *clobaz* clobazam, *clonaz* clonazepam, *ethos* ethosuximide, *gabap* gabapentin, *lamot* lamotrigine, *levet* levetiracetam, *oxcar* oxcarbazepine, *pheno* phenobarbital, *pheny* phenytoin, *primid* primidone, *topir* topiramate, *valpro* valproate, *vigab* vigabatrin

The median baseline risk of cleft lip/palate in the control group (no AED exposure) across all studies was 0.000 (interquartile range, 0.000–0.000; Additional file 3: Appendix K). The NMA on cleft lip/palate included 1 RCT, 1 case-control, and 27 cohort studies, 18,987 cases, and 32 AEDs plus control, with 11% of comparisons reaching statistical significance (Additional file 3: Appendices J, L, and N). The following monotherapies were associated with statistically significantly more cases developing cleft lip/palate than control (Fig. 7a): ethosuximide (OR, 22.22; 95% CrI, 4.56–87.64), primidone (OR, 7.68; 95% CrI, 1.41–29.27), topiramate (OR, 6.12; 95% CrI, 1.89–19.05), phenobarbital (OR, 5.75; 95% CrI, 2.41–14.08), phenytoin (OR, 3.11; 95% CrI, 1.31–7.72), and valproate (OR, 3.26; 95% CrI, 1.38–5.58). In addition, the following polytherapies were associated with statistically significantly more cases developing cleft lip/palate than control: phenobarbital plus phenytoin plus primidone (OR, 11.50; 95% CrI, 1.70–63.48), phenytoin plus primidone (OR, 16.75; 95% CrI, 3.02–77.19), carbamazepine plus phenobarbital (OR, 18.51; 95% CrI, 3.34–94.21), and carbamazepine plus valproate (OR, 19.12; 95% CrI, 3.74–88.68).

### Club foot

The median baseline risk of club foot in the control group (no AED exposure) across all studies was 0.000 (interquartile range, 0.000–0.000; Additional file 3: Appendix K). The NMA for club foot included 1 RCT, 1 case-control, and 21 cohort studies, 8836 cases, and 26 AEDs plus control, with 7% of comparisons reaching statistical significance (Additional file 3: Appendices J, L, and N). Phenytoin (OR, 2.73; 95% CrI, 1.13–6.18), valproate (OR, 3.26; 95% CrI, 1.43–8.25), primidone (OR, 4.71; 95% CrI, 1.11–17.24), ethosuximide (OR, 12.99; 95% CrI, 1.66–76.39), carbamazepine plus phenobarbital (OR, 7.30; 95% CrI, 1.29–32.31), and phenobarbital plus phenytoin plus primidone (OR, 13.46; 95% CrI, 1.45–132.80) were associated with statistically significantly more cases developing club foot than control (Fig. 7b).

### Inguinal hernia

**Fig. 8:**
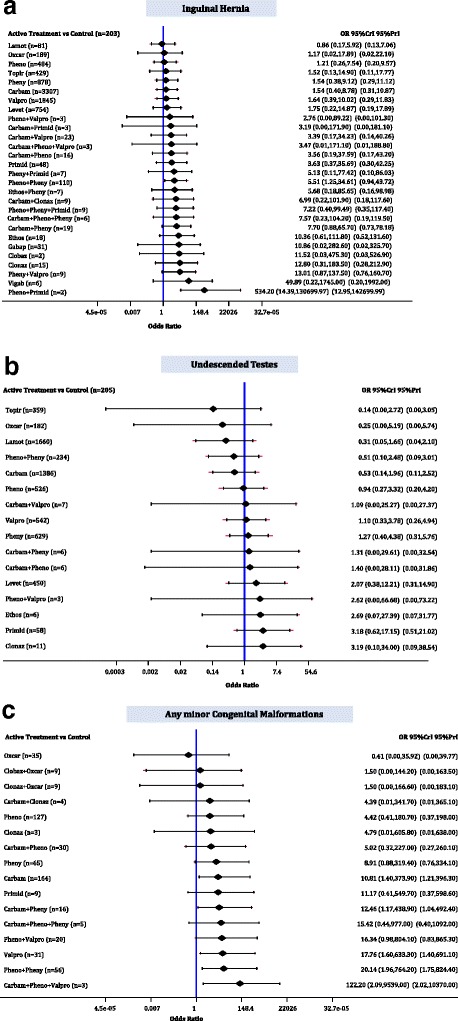
Network meta-analysis forest plots for each treatment versus control. Each rhombus represents the summary treatment effect estimated in the network meta-analysis on the odds ratio (OR) scale. The black horizontal lines represent the credible intervals (CrI) for the summary treatment effects, and the red horizontal lines represent the corresponding predictive intervals (PrI). In the absence of heterogeneity, the CrIs and PrIs should be identical. An OR > 1 suggests that control is safer, whereas an OR < 1 suggests that the comparator active treatment is safer. The vertical blue line corresponds to an OR = 1 (i.e., the treatment groups compared are equally safe). The total sample size (n) included in each treatment is also presented. **a** Inguinal hernia (13 studies, 12,216 cases, 29 treatments). **b** Undescended testes (10 studies, 6270 cases, 17 treatments). **c** Minor congenital malformations (9 studies, 614 cases, 17 treatments). *carbam* carbamazepine, *clobaz* clobazam, *clonaz* clonazepam, *ethos* ethosuximide, *gabap* gabapentin, *lamot* lamotrigine, *levet* levetiracetam, *oxcar* oxcarbazepine, *pheno* phenobarbital, *pheny* phenytoin, *primid* primidone, *topir* topiramate, *valpro* valproate, *vigab* vigabatrin

The median baseline risk of inguinal hernia in the control group (no AED exposure) across all studies was 0.000 (interquartile range, 0.000–0.000; Additional file 3: Appendix K). The NMA for inguinal hernia included 1 RCT, 1 case-control, and 11 cohort studies, 12,216 cases, and 28 AEDs plus control, with 8% of comparisons reaching statistical significance (Additional file 3: Appendices J, L, and N). Phenobarbital plus phenytoin (OR, 5.51; 95% CrI, 1.25–34.61) and phenobarbital plus primidone (OR, 534.20; 95% CrI, 14.39–1.31 × 10^5^) were associated with statistically significantly more cases developing inguinal hernia than control (Fig. 8a).

### Undescended testes

The median baseline risk of undescended testes in the control group (no AED exposure) across all studies was 0.000 (interquartile range, 0.000–0.026; Additional file 3: Appendix K). The NMA for undescended testes included 1 RCT, 1 case-control, and 8 cohort studies, 6270 boys, and 16 AEDs plus control, with 3% of comparisons reaching statistical significance (Additional file 3: Appendices J, L, and N). Nothing was statistically significant versus control (Fig. 8b).

### Any minor CMs

The median baseline risk of any minor CM in the control group (no AED exposure) across all studies was 0.000 (interquartile range, 0.000–0.000; Additional file 3: Appendix K). The NMA for minor CMs included 1 RCT and 8 studies, 614 cases, and 16 AEDs plus control, with 10% of comparisons reaching statistical significance (Additional file 3: Appendices J, L, and N). Carbamazepine (OR, 10.81; 95% CrI, 1.40–373.90), carbamazepine pus phenytoin (OR, 12.46; 95% CrI, 1.17–438.90), valproate (OR, 17.76; 95% CrI, 1.60–633.30), phenobarbital plus phenytoin (OR, 20.14; 95% CrI, 1.96–764.20), and carbamazepine plus phenobarbital plus valproate (OR, 122.20; 95% CrI, 2.09–9539.00) were associated with statistically significantly more cases developing any minor CM than control (Fig. 8c).

## Discussion

There is concern that most AEDs introduce the risk of abnormal or delayed physical development for infants who are exposed in utero. Our results show that, across major and minor CM outcomes, many AEDs were associated with higher risk of CMs than control. The monotherapies associated with statistically significant risk of CMs and prenatal harms compared to control across two or more NMAs were carbamazepine (overall major and minor CMs), clobazam (prenatal growth retardation, preterm birth), ethosuximide (overall major CM, cleft lip/palate, club foot), gabapentin (cardiac malformations, hypospadias), phenobarbital (overall major CM, prenatal growth retardation, cleft lip/palate), phenytoin (overall major CM, cleft lip/palate, club foot), topiramate (overall major CM, combined fetal losses, prenatal growth retardation, cleft lip/palate), and valproate (overall major and minor CMs, combined fetal losses, hypospadias, cleft lip/palate, club foot). Of these, only topiramate and gabapentin are newer generation AEDs. Gabapentin lacked sufficient evidence to reach statistical significance in overall major CM, and had an overall risk of malformations equivalent to control. This finding may be due to the inclusion of major malformations that were detected at birth only, which may decrease the possibility that all cardiac malformations were identified, especially those that can be detected later in childhood (or adulthood). Our results suggest that there is a significant association between topiramate and increased combined fetal losses. However, the treatment effect of topiramate versus control could only be estimated indirectly with high uncertainty. In the network, topiramate was informed by a single, small, five-arm cohort study [42], with only two patients exposed in topiramate (total sample size, *n* = 25) and low methodological quality regarding the comparability of cohorts and adequacy of follow-up. The following newer generation AEDs were not associated with statistically significant risks to physical development compared to control: lamotrigine (*n* = 6290), levetiracetam (*n* = 1015), oxcarbazepine (*n* = 372), and vigabatrin (*n* = 23). However, this does not mean that these agents are not harmful to the offspring of mothers administered these agents (i.e., risks have not been ruled out). Overall, the newer AED agents, including levetiracetam and lamotrigine, were associated with lower risk of overall major CMs and CMs by specific type; however, data from more patients were available for lamotrigine than levetiracetam (6290 versus 1015 total infants, respectively), thereby providing greater confidence in lamotrigine’s safety profile. Further, lamotrigine ranked as the second safest monotherapy for prenatal growth retardation, and was comparable to control for preterm birth. Phenobarbital was the AED monotherapy with the lowest risk of fetal loss, whereas phenytoin was the monotherapy associated with the lowest risk of impaired prenatal growth retardation. Vigabatrin and oxcarbazepine were the least likely monotherapies to increase the risk for preterm birth; however, vigabatrin included only 13 infants compared to the 1045 infants in oxcarbazepine, which contributed to the lower precision in the estimation of vigabatrin’s SUCRA curve value (Fig. 2 and Additional file 3: Appendix N). While gabapentin and clonazepam were ranked as moderately safe, more data are needed to elucidate their potential teratogenicity (329 and 375 infants in total, respectively). Across all outcomes, the following polytherapies were associated with both statistically significant CMs and prenatal harms compared with control across two or more of our NMAs: phenobarbital plus phenytoin, carbamazepine plus phenobarbital, carbamazepine plus phenytoin, phenobarbital plus valproate, phenytoin plus primidone, phenytoin plus valproate, carbamazepine plus valproate, carbamazepine plus clonazepam, phenobarbital plus phenytoin plus primidone, and phenobarbital plus primidone. There is insufficient evidence to make any conclusions regarding polytherapy with newer generation AEDs due to a lack of studies reporting these combinations.

Our study has several strengths. First, we followed the guidelines in the Cochrane Handbook for systematic reviews and ISPOR for NMAs [14], and we reported our findings according to recommendations included the PRISMA-NMA statement [15]. Second, using NMA methods, we were able to compare treatments that have not been compared in previous head-to-head studies, as well as provide a hierarchy of the treatments according to their safety (through the SUCRA curves) [7]. In addition, the complexity of the evidence identified in our systematic review is, in contrast to a pairwise meta-analysis model, properly accounted in a NMA model, which models within-trial correlations induced by the multi-arm studies [43]. Third, our study results are based on a larger number of studies compared to previous knowledge syntheses [7]. A previous systematic review [7] including 59 studies and a total of 65,553 pregnant women examined the risk of malformations in women with epilepsy and showed that the most common were cardiac malformations. The number of pregnancies in this review was higher than our systematic review because of the inclusion of studies that did not analyze the risk by AED and used unspecified polytherapy, which could not be included in our NMA. In contrast to this review, our study assesses each AED separately for both overall and specific malformations, and hence our results are not directly comparable to this review. Fourth, we accounted for the different study designs by applying the Schmitz et al. [33] approach. In this three-level hierarchical model we considered two different sources of evidence, i.e., the observational studies, including cohort and case-control studies, and the RCTs. To account for the potential differences between cohort studies and case-control studies, as the approaches of these two methodologies vary, we conducted a sensitivity analysis restricting to cohort studies (k = 75) for the primary outcome, which included all study designs and the greatest number of case-control studies (k = 2) and RCTs (k = 1). As expected, since the majority of the included study designs were cohort studies, all approaches suggested comparable results. To the best of our knowledge, our study was the first to compare and rank the safety of AEDs using the SUCRA curves and rank-heat plots [36, 37].

Our study has some limitations worth noting. First, we did not incorporate differences in drug dosages of the AEDs because this information was rarely reported across the included studies, although a dose-response relationship has been observed for these agents. For instance, a potential modification of the estimated treatment effects may occur if the doses vary considerably across treatment indications, and accounting for the fact that certain AEDs were more widely utilized in other conditions, while some AEDs are almost exclusively used for epilepsy. Second, the paucity of available data is a limitation; many polytherapies were informed by only a few studies and patients, and many studies included zero events in all arms for the specific CMs and were excluded from those analyses. This impacted the treatment group risk across studies; for example, the median risk of the major congenital anomalies per treatment ranged between 0% and 24%. The lack of adequate knowledge of risks for multiple AEDs impacts the NMA results. This affected the SUCRA estimates, which showed several polytherapies with high OR estimates, but with extremely wide CrIs. For example, in overall major CMs, nine polytherapies had SUCRA curve estimates above 74%, but these all had wide CrIs (95% CrI with shorter length, 28–96%; 95% CrI with wider length, 0–100%) potentially due to the small number of patients (range, 3–21) and studies (range, 1–2) informing these interventions (Additional file 3: Appendix K). Indeed, a simulation study [44] assessing the ranking probability for a treatment of being the best in NMA with a different number of studies per comparison, suggested that the probability of being the best may be biased in favor of treatments with a smaller number of studies. Additionally, another study indicated that the SUCRA curve values might be unreliable [45]. As such, our SUCRA curve values need to be interpreted in conjunction with the ORs and 95% CrIs. Third, quality of reporting of the identified observational studies may have introduced bias [46]; 81% did not control for important cofounders, such as maternal age and epilepsy type and severity, and 59% had large attrition rates. Further, some registries measured CMs and there is a risk that may not have consistently collected data on different types of fetal losses (e.g., stillbirths). However, studies were internally consistent across arms with respect to what was reported. The inclusion of observational studies adds on the evaluation of the safety profile of AED treatments and offers the opportunity to generalize evidence. Fourth, despite no evidence of inconsistency, the assessment of transitivity for most treatment effect modifiers suggested that there was an imbalance in the different levels of quality appraisal across treatment comparisons and most outcomes, which may affect NMA results. A possible approach to address this in a future study would be the use of individual patient data in NMA, to allow for adjustment of the relative treatment effects from the observational studies utilizing patient level covariates. This would also aid decision-making to allow tailoring management to individual patient characteristics [47]. Fifth, although adjusted funnel plots suggested no evidence of publication bias and small-study effects, asymmetry may have been masked given several studies compared multiple arms. To reduce the majority of correlations induced by multi-arm studies, we plotted data points corresponding to the study-specific basic parameters. Additionally, babies born every day are exposed to AEDs and although we searched extensively for grey (i.e., difficult to locate or unpublished) literature, we may have missed unpublished data relevant to our research question. Sixth, the strength of evidence in most NMAs may be low due to the small number of studies compared to the number of treatments included in each network. However, the predictive intervals suggested that our results are robust, overall. Seventh, we combined data across study designs to determine how AEDs behave in the ‘real world’. However, this may have introduced heterogeneity in our analyses. We used the naïve approach and the Schmitz et al. [33] model to combine different study designs, as well as sensitivity analyses on observational and cohort studies separately, and all approaches suggested similar results. Although RCTs are considered to be the gold standard of evidence, we included observational studies in our analyses due to the dearth of available RCTs.

It should be highlighted that, although some of the individual malformations in this review exceeded the number of pregnancies yielding malformations, the unit of analysis in our study was the number of infants with a malformation at birth. Therefore, discussion of the prevalence of multiple malformations would be beyond the scope of the current article. Future studies should assess safety and effectiveness of AEDs for pregnant women considering factors that could affect the results, such as alcohol and folic acid use. Observational studies should follow the STROBE guidance to improve the quality of reporting [48]. Despite recent large-scale registries evaluating rare harms [28, 49–52], more evidence is required to conclude which polytherapy is the safest, especially for the newer-generation AEDs, and to allow better tailoring for patients with different characteristics such as history of alcohol use. Registries should aim to include a suitable control group and collect information on potential confounders to inform which agents are the safest.

## Conclusions

The large volume of evidence in this analysis suggests that the newer generation AEDs, lamotrigine and levetiracetam, were not associated with statistically significant increased risks to CMs compared to control, and were statistically significantly less likely to be associated with children experiencing cardiac malformations than control. In contrast, the risk of malformations was increased for ethosuximide, valproate, topiramate, phenobarbital, phenytoin, carbamazepine, and 11 polytherapies. Additionally, a significant association between topiramate and increased combined fetal losses was identified. However, caution is needed, as the overall low quality of the research available on this subject limits what can be definitively concluded and AEDs may be potentially harmful to infants and children exposed in utero. Counselling is advised concerning teratogenic risks when the prescription is first written for a woman of childbearing potential and before women continue with these agents when considering pregnancy, such as switching from polytherapy to monotherapies with evidence of lower risk and avoiding AEDs, such as valproate, that are consistently associated with CMs. These decisions must be balanced against the need for seizure control.

## Additional files

Additional file 1: Protocol. (PDF 190 kb)

Additional file 2: PRISMA NMA Checklist. (DOCX 25 kb)

Additional file 3: Supplementary Online Content (Appendices A–N). Appendix A. Description of outcomes. Appendix B. List of included articles. Appendix C. Key excluded studies due to only one arm reported with abstractable data. Appendix D. List of studies and their study characteristics. Appendix E. List of studies and their patient characteristics. Appendix F. Risk of bias for randomized controlled trials – Cochrane risk-of-bias tool. Appendix G. Methodological quality of case-control studies – Newcastle-Ottawa Scale. Appendix H. Methodological quality of observational cohort studies – Newcastle-Ottawa Scale. Appendix I. Comparison adjusted funnel plot for each outcome. Appendix J. Statistically significant network meta-analysis results along with meta-analysis results, transitivity, and consistency assessment. Appendix K. Characteristics of the treatment nodes per outcome along with their SUCRA values. Appendix L. Network characteristics per outcome. Appendix M. Meta-regression, subgroup, and sensitivity analyses results. Appendix N. Network diagrams for network meta-analyses of specific and minor congenital malformations. (DOCX 2016 kb)

## Abbreviations

AED: anti-epileptic drugs
CM: congenital malformations
CrI: credible interval
NMA: network meta-analysis
OR: odd ratios
RCT: randomized clinical trials
SUCRA: surface under the cumulative ranking curve
