# Supplementary material for: Comparative safety of anti-epileptic drugs during pregnancy: a systematic review and network meta-analysis of congenital malformations and prenatal outcomes
Source: BMC Med. 2017 May 5;15:95. doi: 10.1186/s12916-017-0845-1 (PMC5418725; doi:10.1186/s12916-017-0845-1)

Supplementary Online Content

[Appendix A. Description of outcomes 2](#_Toc474153629)

[Appendix B. List of included articles 3](#_Toc474153630)

[Appendix C. Key excluded studies due to only one arm reported with abstractable data^a^ 11](#_Toc474153631)

[References 11](#_Toc474153632)

[Appendix D. List of studies and their study characteristics 13](#_Toc474153633)

[Appendix E. List of studies and their patient characteristics 33](#_Toc474153634)

[Appendix F. Risk of bias for randomized controlled trials – Cochrane risk-of-bias tool 38](#_Toc474153635)

[Appendix G. Methodological quality of case-control studies – Newcastle Ottawa Scale 39](#_Toc474153636)

[Appendix H. Methodological quality of observational cohort studies – Newcastle Ottawa Scale 40](#_Toc474153637)

[Appendix I. Comparison adjusted funnel plot for each outcome^a^ 45](#_Toc474153638)

[Appendix J. Statistically significant network meta-analysis results along with meta-analysis results, transitivity, and consistency assessment^a^ 46](#_Toc474153639)

[Appendix K. Characteristics of the treatment nodes per outcome along with their SUCRA values 68](#_Toc474153640)

[Appendix L. Network characteristics per outcome 80](#_Toc474153641)

[Appendix M. Meta-regression, subgroup and sensitivity analyses results 81](#_Toc474153642)

[Statistically Significant Meta-Regression results for Major Congenital Malformations 81](#_Toc474153643)

[Statistically Significant Schmitz Results for Major Congenital Malformations 87](#_Toc474153644)

[Statistically Significant Sensitivity Results for Major Congenital Malformations 102](#_Toc474153645)

[Appendix N. Network diagrams for NMAs of specific and minor congenital malformations 120](#_Toc474153646)

## Appendix A. Description of outcomes

| ***Outcome*** | **Definition/Measure/Cut-off value** |
| --- | --- |
| ***Major congenital malformation^a^*** | Malformation present at birth with surgical, medical, functional, or cosmetic importance |
| ***Minor congenital malformation*** | CM that does not qualify to be classified as a Major CM |
| ***Combined Fetal Losses*** (combined outcome) | |
| Miscarriage/spontaneous abortion | Loss of pregnancy <24 weeks from last menstrual period |
| Stillbirth/Intrauterine death | Loss of pregnancy >24 weeks from last menstrual period |
| Elective termination | Elective termination of pregnancy due to pre-natal diagnosis of congenital malformation |
| ***Pre-natal Growth Retardation*** (combined outcome) | |
| Low Birth Weight | Birth weight <2,500 g; or <10^th^ percentile for gestational age and gender |
| Intrauterine Growth Retardation | Birth weight <10^th^ percentile (corrected for gestational age and gender) |
| Small Head | Head circumference <10^th^ percentile; diagnosis of microcephaly |
| Short Length | Height <5^th^ percentile |
| ***Preterm Birth*** | Delivery at <36 weeks gestation; or as defined by study authors (no cut-off value reported) |

a Includes cases of pre-natal diagnosis of a malformation that resulted in elective termination

## Appendix B. List of included articles

1. Abe K, Hamada H, Yamada T, Obata-Yasuoka M, Minakami H, Yoshikawa H. Impact of planning of pregnancy in women with epilepsy on seizure control during pregnancy and on maternal and neonatal outcomes. Seizure. 2014;23(2):112-116.

2. Al Bunyan M, Abo-Talib Z. Outcome of pregnancies in epileptic women: a study in Saudi Arabia. Seizure. 1999;8(1):26-29.

3. Annegers JF, Elveback LR, Hauser WA, Kurland LT. Do anticonvulsants have a teratogenic effect? Arch Neurol. 1974;31(6):364-373.

4. Annegers JF, Hauser WA, Elveback LR, Anderson VE, Kurland LI. Congenital malformations and seizure disorders in the offspring of parents with epilepsy. Int J Epidemiol. 1978;7(3):241-247.

5. Annegers JF, Kurland LT, Hauser WA. Teratogenicity of anticonvulsant drugs. Res Publ Assoc Res Nerv Ment Dis. 1983;61:239-248.

6. Annegers JF, Elveback LR, Hauser WA, Kurland LT. Epilepsy anticonvulsants and malformations. Birth Defects Orig Artic Ser. 1975;11(5):157-160.

7. Arkilo D, Hanna J, Dickens D, Justesen L, Brunn J, Garland S et al. Pregnancy and neurodevelopmental outcomes with in-utero antiepileptic agent exposure. A pilot study. Eur J Paediatr Neurol. 2015;19(1):37-40.

8. Artama M, Auvinen A, Raudaskoski T, Isojarvi I, Isojarvi J. Antiepileptic drug use of women with epilepsy and congenital malformations in offspring. Neurology. 2005;64(11):1874-1878.

9. Artama M, Gissler M, Malm H, Ritvanen A. Antiepileptic Drug Use during the First Trimester of Pregnancy and Major Congenital Anomalies in Offspring, Finland 1996–2006. In: ICPE: International Conference on Pharmacoepidemiology & Therapeutic Risk Management. Chicago: Pharmacoepidemiology and Drug Safety; 2011: S1–S364.

10. Artama M, Auvinen A, Isojarvi JIT. Pregnancy outcomes in women with epilepsy taking oxcarbazepine: findings from a Finnish national epilepsy cohort. In: European Congress on Epileptology: 2004; Vienna, Austria: Epilepsia; 2004: S3-130-131.

11. Artama M, Gissler M, Malm H, Ritvanen A. Effects of maternal epilepsy and antiepileptic drug use during pregnancy on perinatal health in offspring: nationwide, retrospective cohort study in Finland. Drug Saf. 2013;36(5):359-369.

12. Arteaga-Vázquez J, Luna-Muñoz L, Mutchinick OM. Malformaciones congénitas en hijos de madres epilépticas con y sin tratamiento con anticonvulsivantes. Salud Publica Mex. 2012;54:579-586.

13. Babic M, Jovic N. Postnatal concerns in children born to women with juvenile myoclonic epilepsy. In: European Congress on Epileptology. vol. 55. Stockholm: Epilepsia; 2014: 128.

14. Bag S, Behari M, Ahuja GK, Karmarkar MG. Pregnancy and epilepsy. J Neurol. 1989;236(5):311-313.

15. Banhidy F, Puho EH, Czeizel AE. Efficacy of medical care of epileptic pregnant women based on the rate of congenital abnormalities in their offspring. Congenital anomalies. 2011;51(1):34-42.

16. Czeizel AE, Bod M, Halasz P. Evaluation of anticonvulsant drugs during pregnancy in a population-based Hungarian study. Eur J Epidemiol. 1992;8(1):122-127.

17. Czeizel AE, Dudas I, Banhidy F. Interpretation of controversial teratogenic findings of drugs such as phenobarbital. ISRN Obstet Gynecol. 2011;2011:719675.

18. Puho EH, Szunyogh M, Metneki J, Czeizel AE. Drug treatment during pregnancy and isolated orofacial clefts in hungary. Cleft Palate Craniofac J. 2007;44(2):194-202.

19. Barqawi R. Evaluation of antiepileptic drugs in pregnancy in a Jordanian army hospital. Eastern Mediterranean health journal = La revue de sante de la Mediterranee orientale = al-Majallah al-sihhiyah li-sharq al-mutawassit. 2005;11(4):601-605.

20. Barroso FVL, Araujo Júnior E, Guazelli CAF, Santana EFM, Rolo LC, Martins MdG et al. Perinatal outcomes from the use of antiepileptic drugs during pregnancy: a case-control study. The Journal of Maternal-Fetal & Neonatal Medicine. 2015;28(12):1445-1450.

21. Battino D, Kaneko S, Andermann E, Avanzini G, Canevini MP, Canger R et al. Intrauterine growth in the offspring of epileptic women: a prospective multicenter study. Epilepsy Res. 1999;36(1):53-60.

22. Battino D, Granata T, Binelli S, Caccamo ML, Canevini MP, Canger R et al. Intrauterine growth in the offspring of epileptic mothers. Acta Neurol Scand. 1992;86(6):555-557.

23. Bertollini R, Kallen B, Mastroiacovo P, Robert E. Anticonvulsant drugs in monotherapy. Effect on the fetus. Eur J Epidemiol. 1987;3(2):164-171.

24. Campbell E, Kennedy F, Russell A, Smithson WH, Parsons L, Morrison PJ et al. Malformation risks of antiepileptic drug monotherapies in pregnancy: updated results from the UK and Ireland Epilepsy and Pregnancy Registers. J Neurol Neurosurg Psychiatry. 2014;85(9):1029-1034.

25. Morrow J, Russell A, Guthrie E, Parsons L, Robertson I, Waddell R et al. Malformation risks of antiepileptic drugs in pregnancy: a prospective study from the UK Epilepsy and Pregnancy Register. J Neurol Neurosurg Psychiatry. 2006;77(2):193-198.

26. Mawhinney E, Craig J, Morrow J, Russell A, Smithson WH, Parsons L et al. Levetiracetam in pregnancy: results from the UK and Ireland epilepsy and pregnancy registers. Neurology. 2013;80(4):400-405.

27. Hunt S, Russell A, Smithson WH, Parsons L, Robertson I, Waddell R et al. Topiramate in pregnancy: preliminary experience from the UK Epilepsy and Pregnancy Register. Neurology. 2008;71(4):272-276.

28. Campbell E, Devenney E, Morrow J, Russell A, Smithson WH, Parsons L et al. Recurrence risk of congenital malformations in infants exposed to antiepileptic drugs in utero. Epilepsia. 2013;54(1):165-171.

29. Morrow JI, Hunt SJ, Russell AJ, Smithson WH, Parsons L, Robertson I et al. Folic acid use and major congenital malformations in offspring of women with epilepsy: a prospective study from the UK Epilepsy and Pregnancy Register. J Neurol Neurosurg Psychiatry. 2009;80(5):506-511.

30. Ban L, Fleming KM, Doyle P, Smeeth L, Hubbard RB, Fiaschi L et al. Congenital Anomalies in Children of Mothers Taking Antiepileptic Drugs with and without Periconceptional High Dose Folic Acid Use: A Population-Based Cohort Study. PLoS One. 2015;10(7):e0131130.

31. Canger R, Guidolin L, Canevini M, Battino D, Vignoli A, Fumarola C et al. Malformations in the offspring of women with epilepsy: a prospective study. Boll Lega It Epil. 1997(99):301-304.

32. Bossi L. Fetal Effects of Anticonvulsants. In: Antiepileptic Drug Therapy in Pediatrics. edn. Edited by Morselli P, Pippenger C, Penry J. New York: Raven Press; 1983.

33. Molteni F, Fumarola C, Minotti L, Mai R, Battino D, Canevini MP et al. Pregnancy management and outcome in 505 patients with epilepsy. Bollettino - Lega Italiana contro l'Epilessia. 1992(79/80):267-268.

34. Canger R, Battino D, Canevini MP, Fumarola C, Guidolin L, Vignoli A et al. Malformations in offspring of women with epilepsy: a prospective study. Epilepsia. 1999;40(9):1231-1236.

35. Battino D, Binelli S, Caccamo ML, Canevini MP, Canger R, Como ML et al. Malformations in offspring of 305 epileptic women: a prospective study. Acta Neurol Scand. 1992;85(3):204-207.

36. Cassina M, Dilaghi A, Di Gianantonio E, Cesari E, De Santis M, Mannaioni G et al. Pregnancy outcome in women exposed to antiepileptic drugs: teratogenic role of maternal epilepsy and its pharmacologic treatment. Reprod Toxicol. 2013;39:50-57.

37. Charlton RA, Weil JG, Cunnington MC, Ray S, de Vries CS. Comparing the General Practice Research Database and the UK Epilepsy and Pregnancy Register as tools for postmarketing teratogen surveillance: anticonvulsants and the risk of major congenital malformations. Drug Saf. 2011;34(2):157-171.

38. Cummings C, Stewart M, Stevenson M, Morrow J, Nelson J. Neurodevelopment of children exposed in utero to lamotrigine, sodium valproate and carbamazepine. Arch Dis Child. 2011;96(7):643-647.

39. Tomson T, Battino D, Bonizzoni E, Craig JJ, Lindhout D, Perucca E et al. Antiepileptic drugs and intrauterine death: A prospective observational study from EURAP. Neurology. 2015;85(7):580-588.

40. Dansky L, Andermann E, Andermann F. Major congenital malformations in the offspring of epileptic patients: genetic and environmental risk factors. In: Epilepsy, Pregnancy and the Child. edn. Edited by Janz D, Bossi L, Dam M, Helge H, Richens A, Schmidt D. New York: Raven Press; 1982.

41. Dansky LV. Outcome of Pregnancy ln Epileptic Women: A Prospective Evaluation of Genetic and Environmental Rlsk Factors. Montreal, Canada: McGill University; 1989.

42. Dean JCS, Hailey H, Moore SJ, Lloyd DJ, Turnpenny PD, Little J. Long term health and neurodevelopment in children exposed to antiepileptic drugs before birth. J Med Genet. 2002;39(4):251-259.

43. Rasalam AD, Hailey H, Williams JH, Moore SJ, Turnpenny PD, Lloyd DJ et al. Characteristics of fetal anticonvulsant syndrome associated autistic disorder. Dev Med Child Neurol. 2005;47(8):551-555.

44. Delmis J, Drazancic A, Tkalcevic T, Ivanisevic M. [Epilepsy and pregnancy]. Jugosl Ginekol Perinatol. 1991;31(1-2):23-26.

45. Dessens AB, Cohen-Kettenis PT, Mellenbergh GJ, Koppe JG, Poll NE, Boer K. Association of prenatal phenobarbital and phenytoin exposure with genital anomalies and menstrual disorders. Teratology. 2001;64(4):181-188.

46. Dessens AB. Prenatal exposure to phenobarbital and diphantoin: A study on long-lasting consequences. Amsterdam: University of Amsterdam; 1996.

47. D'Souza SW, Robertson IG, Donnai D, Mawer G. Fetal phenytoin exposure, hypoplastic nails, and jitteriness. Arch Dis Child. 1991;66(3):320-324.

48. Eroglu E, Gokcil Z, Bek S, Ulas UH, Odabasi Z. Pregnancy and teratogenicity of antiepileptic drugs. Acta Neurol Belg. 2008;108(2):53-57.

49. Fairgrieve SD, Jackson M, Jonas P, Walshaw D, White K, Montgomery TL et al. Population based, prospective study of the care of women with epilepsy in pregnancy, vol. 321; 2000.

50. Farmen AH, Grundt J, Tomson T, Nakken KO, Nakling J, Mowinchel P et al. Intrauterine growth retardation in foetuses of women with epilepsy. Seizure. 2015;28:76-80.

51. Fedrick J. Epilepsy and pregnancy: a report from the Oxford Record Linkage Study. Br Med J. 1973;2(5864):442-448.

52. Gaily E. Development and growth in children of epileptic mothers: a prospective controlled study. Helsinki, Finland: University of Helsinki; 1990.

53. Gaily EK, Granstrom ML, Hiilesmaa VK, Bardy AH. Head circumference in children of epileptic mothers: contributions of drug exposure and genetic background. Epilepsy Res. 1990;5(3):217-222.

54. Hiilesmaa V. A prospective study on maternal and fetal outcome in 139 women with epilepsy. Helsinki: University of Helsinki; 1982.

55. Hiilesmaa VK, Bardy A, Teramo K. Obstetric outcome in women with epilepsy. Am J Obstet Gynecol. 1985;152(5):499-504.

56. Hernandez-Diaz S, Smith CR, Shen A, Mittendorf R, Hauser WA, Yerby M et al. Comparative safety of antiepileptic drugs during pregnancy. Neurology. 2012;78(21):1692-1699.

57. Hernandez-Diaz S, Mittendorf R, Smith CR, Hauser WA, Yerby M, Holmes LB. Association between topiramate and zonisamide use during pregnancy and low birth weight. Obstet Gynecol. 2014;123(1):21-28.

58. Hill RM, Verniaud WM, Rettig GM, Tennyson LM, Craig JP. Relationship Between Antiepileptic Drug Exposure of the Infant and Developmental Potential. In: Epilepsy, Pregnancy, and the Child. edn. Edited by Janz D. New York: Raven Press; 1982.

59. Holmes LB, Harvey EA, Coull BA, Huntington KB, Khoshbin S, Hayes AM et al. The teratogenicity of anticonvulsant drugs. N Engl J Med. 2001;344 (15):1132-1138.

60. Hunter RW, Allen EM. The course and outcome of pregnancy in women with epilepsy—a 6-year prospective study. J Obstet Gynaecol. 1990;10(6):483-491.

61. Hvas CL, Henriksen TB, Ostergaard JR, Dam M. Epilepsy and pregnancy: effect of antiepileptic drugs and lifestyle on birthweight. BJOG. 2000;107(7):896-902.

62. Jacobsen PE, Henriksen TB, Haubek D, Ostergaard JR. Prenatal exposure to antiepileptic drugs and dental agenesis. PLoS One. 2014;9(1):e84420.

63. Janz D, Fuchs U. Are anti-epileptic drugs harmful during pregnancy? Dtsch Med Wochenschr. 1964;89:241-248.

64. Jones KL, Lacro RV, Johnson KA, Adams J. Pattern of malformations in the children of women treated with carbamazepine during pregnancy. N Engl J Med. 1989;320(25):1661-1666.

65. Juarez-Olguin H, Belmont-Gomez A, Flores-Perez J, Barranco-Garduno LM, Flores-Perez C. Malformations in newborns associated to anticonvulsant consumption during pregnancy. experience in third level hospital of Mexico. Rev Invest Clin. 2008;60(1):15-20.

66. Kaabi W, El Aidli S, Kastalli S, Lakhoua G, Zaiem A, Srairi S et al. Pregnancy Outcomes in Women Using Antiepileptic Drugs - Abstract Code: SP3556-47. Drug Saf. 2013;36(9):844.

67. Kaaja E, Kaaja R, Hiilesmaa V. Major malformations in offspring of women with epilepsy. Neurology. 2003;60(4):575-579.

68. Kallen B, Borg N, Reis M. The use of central nervous system active drugs during pregnancy. Pharmaceuticals (Basel). 2013;6(10):1221-1286.

69. Wide K, Winbladh B, Kallen B. Major malformations in infants exposed to antiepileptic drugs in utero, with emphasis on carbamazepine and valproic acid: a nation-wide, population-based register study. Acta Paediatr. 2004;93(2):174-176.

70. Kaneko S, Battino D, Andermann E, Wada K, Kan R, Takeda A et al. Congenital malformations due to antiepileptic drugs. Epilepsy Res. 1999;33(2-3):145-158.

71. Kaneko S, Otani K, Fukushima Y, Ogawa Y, Nomura Y, Ono T et al. Teratogenicity of antiepileptic drugs: analysis of possible risk factors. Epilepsia. 1988;29(4):459-467.

72. Kaneko S, Fukushima Y, Sato T, Ogawa Y, Nomura Y, T O et al. Teratogenicity of Antiepileptic Drugs-A Prospective Study. The Japanese Journal of Psychiatry and Neurology. 1986;40(3):447-450.

73. Kelly TE, Edwards P, Rein M, Miller JQ, Dreifuss FE. Teratogenicity of anticonvulsant drugs. II: A prospective study. Am J Med Genet. 1984;19(3):435-443.

74. Kilic D, Pedersen H, Kjaersgaard MI, Parner ET, Vestergaard M, Sorensen MJ et al. Birth outcomes after prenatal exposure to antiepileptic drugs--a population-based study. Epilepsia. 2014;55(11):1714-1721.

75. Molgaard-Nielsen D, Hviid A. Newer-generation antiepileptic drugs and the risk of major birth defects. JAMA. 2011;305(19):1996-2002.

76. Kini U, Adab N, Vinten J, Fryer A, Clayton-Smith J. Dysmorphic features: an important clue to the diagnosis and severity of fetal anticonvulsant syndromes. Arch Dis Child Fetal Neonatal Ed. 2006;91(2):F90-95.

77. Koch S, Lösche G, Jager-Romän E, Jakob S, Rating D, Deichl A et al. Major and minor birth malformations and antiepileptic drugs. Neurology. 1992;42(4 Suppl 5):83-88.

78. Jager-Roman E, Deichl A, Jakob S, Hartmann AM, Koch S, Rating D et al. Fetal growth, major malformations, and minor anomalies in infants born to women receiving valproic acid. J Pediatr. 1986;108(6):997-1004.

79. Koch S, Gopfert-Geyer I, Jager-Roman E, Jakob S, Huth H, Hartmann A et al. [Anticonvulsants during pregnancy: A prospective study of course of pregnancy, malformations and child development]. Dtsch Med Wochenschr. 1983;108(7):250-257.

80. Koch S, Jager-Roman E, Losche G, Nau H, Rating D, Helge H. Antiepileptic drug treatment in pregnancy: drug side effects in the neonate and neurological outcome. Acta Paediatr. 1996;85(6):739-746.

81. Kochen S, Salera C, Seni J. Pregnant women with epilepsy in a developing country. Open Neurol J. 2011;5:63-67.

82. Kulaga S, Sheehy O, Zargarzadeh AH, Moussally K, Berard A. Antiepileptic drug use during pregnancy: perinatal outcomes. Seizure. 2011;20(9):667-672.

83. Laine-Cessac P, Le Jaoen S, Rosenau L, Gamelin L, Allain P, Grosieux P. [Uncontrolled retrospective study of 75 pregnancies in women treated for epilepsy]. J Gynecol Obstet Biol Reprod (Paris). 1995;24(5):537-542.

84. Lowe CR. Congenital malformations among infants born to epileptic women. Lancet. 1973;1(7793):9-10.

85. Markestad T, Ulstein, M. and Strandjord, R. E. OUTCOME OF PREGNANCY IN WOMEN WITH EPILEPSY. Acta Neurol Scand. 1984;69(79-80).

86. Martinez Ferri M, Pena Mayor P, Perez Lopez-Fraile I, Castro Vilanova MD, Escartin Siquier A, Martin Moro M et al. [Malformations and fetal death in the Spanish antiepileptic drug and pregnancy registry: results at 6 years]. Neurologia. 2009;24(6):360-365.

87. Tomson T, Battino D, Bonizzoni E, Craig J, Lindhout D, Sabers A et al. Dose-dependent risk of malformations with antiepileptic drugs: an analysis of data from the EURAP epilepsy and pregnancy registry. Lancet Neurol. 2011;10(7):609-617.

88. Tomson T, Battino D, Bonizzoni E, Craig J, Lindhout D, Perucca E et al. Dose-dependent teratogenicity of valproate in mono- and polytherapy: an observational study. Neurology. 2015;85(10):866-872.

89. Mawer G, Clayton-Smith J, Coyle H, Kini U. Outcome of pregnancy in women attending an outpatient epilepsy clinic: adverse features associated with higher doses of sodium valproate. Seizure. 2002;11(8):512-518.

90. Mawer G, Briggs M, Baker GA, Bromley R, Coyle H, Eatock J et al. Pregnancy with epilepsy: obstetric and neonatal outcome of a controlled study. Seizure. 2010;19(2):112-119.

91. Meador KJ, Baker GA, Finnell RH, Kalayjian LA, Liporace JD, Loring DW et al. In utero antiepileptic drug exposure: fetal death and malformations. Neurology. 2006;67(3):407-412.

92. Meischenguiser R, D'Giano CH, Ferraro SM. Oxcarbazepine in pregnancy: clinical experience in Argentina. Epilepsy Behav. 2004;5(2):163-167.

93. Melchior JC, Svensmark O, Trolle D. Placental transfer of phenobarbitone in epileptic women, and elimination in newborns. Lancet. 1967;2(7521):860-861.

94. Meyer JG. The teratological effects of anticonvulsants and the effects on pregnancy and birth. Eur Neurol. 1973;10(3):179-190.

95. Millar J, Nevin N. Congenital Malformations and Anti-Convulsant Drugs. The Lancet. 1973:328.

96. Miskov S, Juraski RG, Fucic A, Ivicevic Bakulic T, Mikula I, Cvitanovic Sojat L et al. Croatian Pregnant Women with Epilepsy and Effects of Antiepileptic Drugs Exposure in their Offspring - seven years of prospective surveillance. In: American Epilepsy Society. Texas; 2010.

97. Miyakoshi M, Seino M. Malformations in children born to mothers with epilepsy. In: Antiepileptic drugs and pregnancy. edn. Edited by Shinagawa S, Sato T. Amsterdam: Excerpta Medica; 1984: 125-131.

98. Montouris G. Gabapentin exposure in human pregnancy: results from the Gabapentin Pregnancy Registry. Epilepsy Behav. 2003;4(3):310-317.

99. Nadebaum C, Anderson VA, Vajda F, Reutens DC, Barton S, Wood AG. Language skills of school-aged children prenatally exposed to antiepileptic drugs. Neurology. 2011;76(8):719-726.

100. Nulman I, Scolnik D, Chitayat D, Farkas LD, Koren G. Findings in children exposed in utero to phenytoin and carbamazepine monotherapy: independent effects of epilepsy and medications. Am J Med Genet. 1997;68(1):18-24.

101. Gladstone DJ, Bologa M, Maguire C, Pastuszak A, Koren G. Course of pregnancy and fetal outcome following maternal exposure to carbamazepine and phenytoin: a prospective study. Reprod Toxicol. 1992;6(3):257-261.

102. Scolnik D, Nulman I, Rovet J, Gladstone D, Czuchta D, Gardner HA et al. Neurodevelopment of children exposed in utero to phenytoin and carbamazepine monotherapy. JAMA. 1994;271(10):767-770.

103. Oguni M, Dansky L, Andermann E, Sherwin A, Andermann F. Improved pregnancy outcome in epileptic women in the last decade: relationship to maternal anticonvulsant therapy. Brain Dev. 1992;14(6):371-380.

104. Omtzigt JG, Los FJ, Grobbee DE, Pijpers L, Jahoda MG, Brandenburg H et al. The risk of spina bifida aperta after first-trimester exposure to valproate in a prenatal cohort. Neurology. 1992;42(4 Suppl 5):119-125.

105. Omtzigt JG, Los FJ, Meijer JW, Lindhout D. The 10,11-epoxide-10,11-diol pathway of carbamazepine in early pregnancy in maternal serum, urine, and amniotic fluid: effect of dose, comedication, and relation to outcome of pregnancy. Ther Drug Monit. 1993;15(1):1-10.

106. Ornoy A, Zvi N, Arnon J, Wajnberg R, Shechtman S, Diav-Citrin O. The outcome of pregnancy following topiramate treatment: a study on 52 pregnancies. Reprod Toxicol. 2008;25(3):388-389.

107. Pennell PB, Klein AM, Browning N, Baker GA, Clayton-Smith J, Kalayjian LA et al. Differential effects of antiepileptic drugs on neonatal outcomes. Epilepsy Behav. 2012;24(4):449-456.

108. Pittschieler S, Brezinka C, Jahn B, Trinka E, Unterberger I, Dobesberger J et al. Spontaneous abortion and the prophylactic effect of folic acid supplementation in epileptic women undergoing antiepileptic therapy. J Neurol. 2008;255(12):1926-1931.

109. Porter RJ. Teratogenic Risk of Antiepileptic Drugs, with Special Reference to Sodium Valproate (Valproic Acid) Therapy: A Collaborative Study of the French Chapter of I.L.A.E. In: Advances in Epileptology: The XVth Epilepsy International Symposium. edn. Edited by Porter RJ. New York: Raven Press; 1984: 299-307.

110. Bertollini R, Källen B, Mastroiacovo P, Robert E. Anticonvulsant drugs in monotherapy. Effect on the fetus. Eur J Epidemiol. 1987;3(2):164-171.

111. Rating D, Jager-Roman E, Koch S, Deichl A, Hartmann H, Jakob S et al. Major malformations and minor anomalies in the off-spring of epileptic parents: the role of antiepileptic drugs. In: Pharmacokinetics in teratogenesis. edn. Edited by Nau H, Scott WJJ. Boca Raton, FL: CRC Press; 1987: 205-223.

112. Regesta G, Tanganelli P. The risk of malformations and developmental disturbances in children exposed to antiepileptic drugs: a prospective controlled study. Boll Lega It Epil. 1996;95/96:351-354.

113. Rihtman T, Parush S, Ornoy A. Developmental outcomes at preschool age after fetal exposure to valproic acid and lamotrigine: cognitive, motor, sensory and behavioral function. Reprod Toxicol. 2013;41:115-125.

114. Robert E, Lofkvist E, Mauguiere F, Robert JM. Evaluation of drug therapy and teratogenic risk in a Rhone-Alpes district population of pregnant epileptic women. Eur Neurol. 1986;25(6):436-443.

115. Samren EB, van Duijn CM, Christiaens GC, Hofman A, Lindhout D. Antiepileptic drug regimens and major congenital abnormalities in the offspring. Ann Neurol. 1999;46(5):739-746.

116. Sawhney H, Vasishta K, Suri V, Khunnu B, Goel P, Sawhney IM. Pregnancy with epilepsy--a retrospective analysis. Int J Gynaecol Obstet. 1996;54(1):17-22.

117. Sonneveld SW, Correy JF. Outcome of pregnancies complicated by epilepsy in Tasmania 1981-1988. Aust N Z J Obstet Gynaecol. 1990;30(4):286-289.

118. Steegers-Theunissen RP, Renier WO, Borm GF, Thomas CM, Merkus HM, Op de Coul DA et al. Factors influencing the risk of abnormal pregnancy outcome in epileptic women: a multi-centre prospective study. Epilepsy Res. 1994;18(3):261-269.

119. Tanaka H, Takeda A, Izumi M, Okada H, Ishikawa S. [Effects of antiepileptic drugs on delivery and early childhood--comparison among mono-therapies of valproic acid, phenytoin, carbamazepine and phenobarbital]. Rinsho Shinkeigaku - Clinical Neurology. 1991;31(3):266-269.

120. Thomas SV, Ajaykumar B, Sindhu K, Francis E, Namboodiri N, Sivasankaran S et al. Cardiac malformations are increased in infants of mothers with epilepsy. Pediatr Cardiol. 2008;29(3):604-608.

121. Martinez Ferri M, Pena Mayor P, Perez Lopez-Fraile I, Castro Vilanova MD, Escartin Siquier A, Martin Moro M et al. Malformations and fetal death in the Spanish antiepileptic drug and pregnancy registry: results at 6 years. Neurologia. 2009;24(6):360-365.

122. Vajda FJ, O'Brien TJ, Lander CM, Graham J, Eadie MJ. The teratogenicity of the newer antiepileptic drugs - an update. Acta Neurol Scand. 2014;130(4):234-238.

123. Torres LC, Felix R, Canun S, Mazon JJ. [Epilepsy and pregnancy. Risks and benefits of anticonvulsant treatment]. Ginecol Obstet Mex. 1995;63:282-286.

124. Eadie MJ, Vajda FJ. Should valproate be taken during pregnancy? Ther Clin Risk Manag. 2005;1(1):21-26.

125. Vajda FJ, Graham JE, Hitchcock AA, O'Brien TJ, Lander CM, Eadie MJ. Is lamotrigine a significant human teratogen? Observations from the Australian Pregnancy Register. Seizure. 2010;19(9):558-561.

126. Vajda FJ, Graham J, Roten A, Lander CM, O'Brien TJ, Eadie M. Teratogenicity of the newer antiepileptic drugs--the Australian experience. J Clin Neurosci. 2012;19(1):57-59.

127. Vajda FJ, Hitchcock AA, Graham J, O'Brien TJ, Lander CM, Eadie MJ. The teratogenic risk of antiepileptic drug polytherapy. Epilepsia. 2010;51(5):805-810.

128. Vajda FJ, Horgan D, Hollingworth S, Graham J, Hitchcock AA, Roten A et al. The prescribing of antiepileptic drugs for pregnant Australian women. Aust N Z J Obstet Gynaecol. 2012;52(1):49-53.

129. Vajda FJ, O'Brien TJ, Graham JE, Lander CM, Eadie MJ. Dose dependence of fetal malformations associated with valproate. Neurology. 2013;81(11):999-1003.

130. Vajda FJ, O'Brien TJ, Graham J, Lander CM, Eadie MJ. Associations between particular types of fetal malformation and antiepileptic drug exposure in utero. Acta Neurol Scand. 2013;128(4):228-234.

131. van der Pol MC, Hadders-Algra M, Huisjes HJ, Touwen BC. Antiepileptic medication in pregnancy: late effects on the children's central nervous system development. Am J Obstet Gynecol. 1991;164(1 Pt 1):121-128.

132. Vanya M, Arva-Nagy N, Szili K, Szok D, Bartfai G. EFFECTS OF MATERNAL EPILEPSY AND ANTIEPILEPTIC THERAPY IN WOMEN DURING PREGNANCY. Ideggyogyaszati szemle. 2015;68(3-4):105-112.

133. Veiby G, Daltveit AK, Engelsen BA, Gilhus NE. Fetal growth restriction and birth defects with newer and older antiepileptic drugs during pregnancy. J Neurol. 2014;261(3):579-588.

134. Viinikainen K, Heinonen S, Eriksson K, Kalviainen R. Community-based, prospective, controlled study of obstetric and neonatal outcome of 179 pregnancies in women with epilepsy. Epilepsia. 2006;47(1):186-192.

135. Wide K, Winbladh B, Tomson T, Sars-Zimmer K, Berggren E. Psychomotor development and minor anomalies in children exposed to antiepileptic drugs in utero: a prospective population-based study. Dev Med Child Neurol. 2000;42(2):87-92.

136. Wladimiroff JW, Stewart PA, Reuss A, van Swaay E, Lindhout D, Sachs ES. The role of ultrasound in the early diagnosis of fetal structural defects following maternal anticonvulsant therapy. Ultrasound Med Biol. 1988;14(8):657-660.

## Appendix C. Key excluded studies due to only one arm reported with abstractable data^a^

| **First Author, Year** | **Title** | **PMID**^b^ |
| --- | --- | --- |
| Arpino, 2000[1] | Teratogenic effects of antiepileptic drugs: use of an  International Database on Malformations and Drug Exposure (MADRE). | 11077457 |
| Cunnington, 2011[2] | Final results from 18 years of the International Lamotrigine Pregnancy Registry. | 21606453 |
| de Jonge, 2013[3] | Identifying associations between maternal medication use and birth defects using a case-population approach: an exploratory study on signal detection. | 23828658 |
| Dolk, 2008[4] | Does lamotrigine use in pregnancy increase orofacial cleft risk relative to other malformations? | 18650491 |
| Jentink, 2010a[5] | Intrauterine exposure to carbamazepine and specific congenital malformations: systematic review and case-control study. | 21127116 |
| Jentink, 2010b[6] | Valproic acid monotherapy in pregnancy and major congenital malformations. | 20558369 |
| Jentink, 2010c[7] | Does folic acid use decrease the risk for spina bifida after in utero exposure to valproic acid? | 20680999 |
| Lin, 2004[8] | Clonazepam use in pregnancy and the risk of malformations. | 15329832 |
| Mølgaard-Nielsen, 2011[9] | Newer-generation antiepileptic drugs and the risk of major birth defects. | 21586715 |
| Montouris, 2012[10] | Incidence of Congenital Malformations in Infants Born to Patients with Epilepsy: A Comparison of Pregnancy Registries and Cohort Study Data. | Neurology (suppl 1): S56.003^c^ |
| Tennis, 2015^d[11]^ | Topiramate use during pregnancy and major congenital malformations in multiple populations. | 25776342 |
| a Studies where the only comparator was either ‘unspecified’ polytherapy or an unmatched control group.  b PubMed unique identifier.  c Conference abstract not indexed in PubMed.  d Reports monotherapy and polytherapy combined. | | |

### References

1. Arpino C, Brescianini S, Robert E, Castilla EE, Cocchi G, Cornel MC et al. Teratogenic effects of antiepileptic drugs: use of an International Database on Malformations and Drug Exposure (MADRE). Epilepsia. 2000;41(11):1436-1443.

2. Cunnington MC, Weil JG, Messenheimer JA, Ferber S, Yerby M, Tennis P. Final results from 18 years of the International Lamotrigine Pregnancy Registry. Neurology. 2011;76(21):1817-1823.

3. de Jonge L, Zetstra-van der Woude PA, Bos HJ, de Jong-van den Berg LT, Bakker MK. Identifying associations between maternal medication use and birth defects using a case-population approach: an exploratory study on signal detection. Drug Saf. 2013;36(11):1069-1078.

4. Dolk H, Jentink J, Loane M, Morris J, de Jong-van den Berg LT. Does lamotrigine use in pregnancy increase orofacial cleft risk relative to other malformations? Neurology. 2008;71(10):714-722.

5. Jentink J, Dolk H, Loane MA, Morris JK, Wellesley D, Garne E et al. Intrauterine exposure to carbamazepine and specific congenital malformations: systematic review and case-control study. BMJ. 2010;341:c6581.

6. Jentink J, Loane MA, Dolk H, Barisic I, Garne E, Morris JK et al. Valproic acid monotherapy in pregnancy and major congenital malformations. N Engl J Med. 2010;362(23):2185-2193.

7. Jentink J, Bakker MK, Nijenhuis CM, Wilffert B, de Jong-van den Berg LT. Does folic acid use decrease the risk for spina bifida after in utero exposure to valproic acid? Pharmacoepidemiol Drug Saf. 2010;19(8):803-807.

8. Lin AE, Peller AJ, Westgate MN, Houde K, Franz A, Holmes LB. Clonazepam use in pregnancy and the risk of malformations. Birth Defects Res A Clin Mol Teratol. 2004;70(8):534-536.

9. Molgaard-Nielsen D, Hviid A. Newer-generation antiepileptic drugs and the risk of major birth defects. JAMA. 2011;305(19):1996-2002.

10. Montouris G, Harden C, Albano J, Leppik I, Miller D. Incidence of Congenital Malformations in Infants Born to Patients with Epilepsy: A Comparison of Pregnancy Registries and Cohort Study Data (S56.003). Neurology. 2012;78(1 Supplement):S56.003.

11. Tennis P, Chan KA, Curkendall SM, Li DK, Mines D, Peterson C et al. Topiramate use during pregnancy and major congenital malformations in multiple populations. Birth Defects Res A Clin Mol Teratol. 2015;103(4):269-275.

## Appendix D. List of studies and their study characteristics

| **Author, Year** | **Country** | **Setting or Registry** | **Study Period** | **Outcomes** | **Interventions** | **Funding** |
| --- | --- | --- | --- | --- | --- | --- |
| Abe, 2014[1] | Japan | University of Tsukuba Hospital and Hokkaido University Hospital | 2003-2011 | Major CMs, Cardiac CMs, Cleft Lip or Palate, Club Foot, Hypospadias, Inguinal Hernia, Undescended Testes | Control, Pheny, Pheno, Carbam, Clonaz, Valpro | NR |
| Al Bunyan, 1999[2] | Saudi Arabia | King Khalid University Hospital | 1985-1994 | Major CMs, Fetal Losses*, Cardiac CMs, Cleft Lip or Palate, Club Foot, Hypospadias, Inguinal Hernia, Undescended Testes | Control, Pheny, Pheno, Pheno+Pheny, Carbam, Carbam+Pheny, Carbam+Pheno, Valpro | NR |
| Annegers, 1974[3] [CR: Annegers, 1978[4]; Annegers, 1983[5]; Annegers, 1975[6]] | USA | Mayo Clinic | 1939-1972 | Major CMs, Cardiac CMs, Cleft Lip or Palate, Club Foot, Hypospadias, Inguinal Hernia, Undescended Testes | Control, Pheny, Pheno, Pheno+Pheny | public |
| Arkilo, 2015[7] | USA | Minnesota Epilepsy Group | 2006-2011 | Fetal Losses, Preterm Birth | Carbam, Lamot, Levet, Pheny, Valpro | NR |
| Artama, 2005[8] [CR: Artama, 2011[9]; Artama, 2004[10]] | Finland | Medical Birth Register of Finland and Social Insurance Institution Database | 1991-2000 | Cardiac CMs, Cleft Lip or Palate | Control, Pheny, Carbam, Valpro, Oxcar | mixed public & private |
| Artama, 2013[11] | Finland | Finnish Medical Birth Register; Finnish Malformation Register; Special Refund Entitlement Register; Register on Reimbursement Drugs | 1996-2008 | Preterm Birth, Fetal Losses, Pre-natal Growth | Carbam, Clona, Control, Lamot, Levet, Oxcar, Pheny, Valpro | public |
| Arteaga-Vazquez, 2012[12] | Mexico | Registration and Epidemiological Surveillance of Congenital Malformations Registry | 1978-2010 | Major CMs | Control, Pheny, Pheno+Pheny, Carbam, Carbam+Pheny, Valpro | NR |
| Babic, 2014[13] | Serbia | Clinic of Neurology and Psychiatry for Children and Youth, Belgrade | 1998-2008 | Major CMs, Fetal Losses | Lamot, Lamot+Valpro, Levet, Topir, Valpro, |  |
| Bag, 1989[14] | India | All India Institute of Medical Sciences | NR | Major CMs, Fetal Losses, Cardiac CMs, Cleft Lip or Palate, Club Foot, Hypospadias, Inguinal Hernia, Undescended Testes | Pheny, Pheno+Pheny, Carbam, Carbam+Pheny, Carbam+Pheno | NR |
| Banhidy, 2011[15] [CR: Czeizel, 1992[16]; Czeizel, 2011[17]; Puho, 2007[18]] | Hungary | Hungarian Case-Control Surveillance System of Congenital Abnormalities and Hungarian Congenital Abnormalty Registry | 1980-1997 | Major CMs, Cardiac CMs, Cleft Lip or Palate, Club Foot, Hypospadias, Inguinal Hernia, Undescended Testes | Control, Pheny, Ethos, Carbam, Primid, Valpro | NR |
| Barqawi, 2005[19] | Jordan | King Hussein Medical Centre | NR | Major CMs, Fetal Losses, Minor CMs, Cardiac CMs, Cleft Lip or Palate, Club Foot, Hypospadias, Inguinal Hernia, Undescended Testes | Control, Carbam, Carbam+Pheny | NR |
| Barroso, 2015[20] | Brazil | Department of Obstetrics, Federal University of Sao Paulo | 2000-2010 | Major CMs, Minor CMs, Club Foot | Carbam, Lamot, Lamot+Valpro, Levet, Pheno, Topir, Valpro | NR |
| Battino, 1999[21] [CR: Battino, 1992[22]] | Canada; Japan; Italy | NR | 1978-1991 | Pre-natal Growth | Carbam, Control, Pheno, Pheny, Primid, Valpro | NR |
| Bertollini, 1987a[23] | Italy | Italian Multicentric Register on Congenital Malformations | 1983-1985 | Major CMs, Cardiac CMs, Cleft Lip or Palate, Club Foot, Hypospadias, Inguinal Hernia, Undescended Testes | Pheny, Pheno, Carbam, Valpro | mixed public & private |
| Bertollini, 1987b[23] | Sweden | Swedish Medical Birth Registry | 1973-1981 | Major CMs, Cardiac CMs, Cleft Lip or Palate, Club Foot, Hypospadias, Inguinal Hernia, Undescended Testes | Pheny, Pheno, Carbam, Valpro | mixed public & private |
| Campbell, 2013[24-27] [CR: Campbell, 2013[28]; Morrow, 2009[29]; Ban, 2015[30]] | UK; Ireland | UK Epilepsy and Pregnancy Register and Irish Epilepsy and Pregnancy Register | 1996-2012 | Major CMs, Cardiac CMs, Cleft Lip or Palate, Hypospadias, Inguinal Hernia | Control, Pheny, Ethos, Carbam, Clonaz, Valpro, Gabap, Vigab, Lamot, Topir, Oxcar, Levet | mixed public & private |
| Canger, 1997[31] [CR: Bossi, 1983[32]; Molteni, 1992[33]] | Italy | Milan Collaborative Group for the Study of Epilepsy in Pregnancy | 1977-NR | Major CMs, Cardiac CMs, Cleft Lip or Palate, Club Foot, Hypospadias, Inguinal Hernia, Undescended Testes | Control, Pheny, Pheno, Carbam, Primid, Clonaz, Valpro | NR |
| Canger, 1999[34] [CR: Battino, 1992[35]] | Italy | San Paolo Hospital | 1977-1996 | Fetal Losses | Carbam, Clona, Control, Pheno, Pheny, Primid, Valpro | NR |
| Cassina, 2013a[36] | Italy | European Network of Teratology Information Services (ENTIS) | 2000-2008 | Major CMs, Cardiac CMs, Cleft Lip or Palate, Club Foot, Hypospadias, Inguinal Hernia, Undescended Testes | Pheno, Carbam, Valpro, Lamot | NR |
| Cassina, 2013b[36] | Italy | European Network of Teratology Information Services (ENTIS) | 2000-2008 | Major CMs, Cardiac CMs, Cleft Lip or Palate, Club Foot, Hypospadias, Inguinal Hernia, Undescended Testes | Carbam, Valpro, Lamot | NR |
| Charlton, 2011[37] | UK | UK General Practice Research Database (GPRD) | 1990-2006 | Major CMs | Control, Carbam, Valpro, Lamot | mixed public & private |
| Cummings, 2011[38] [CR: Tomson, 2015[39]] | Northern Ireland | UK Epilepsy and Pregnancy Register (Northern Ireland); Northern Ireland Child Health System | 1996-2005 | Preterm Birth | Carbam, Lamot, Valpro | public |
| Dansky, 1982[40] | Canada | Montreal Neurological Hospital | NR | Major CMs, Cardiac CMs, Cleft Lip or Palate | Pheny, Pheno, Pheno+Pheny | NR |
| Dansky, 1989[41] | Canada | Montreal Neurological Hospital | 1972-1984 | Major CMs, Fetal Losses, Cardiac CMs, Cleft Lip or Palate, Club Foot, Hypospadias, Inguinal Hernia, Undescended Testes | Pheny, Pheno, Pheno+Pheny, Ethos+Pheny, Carbam, Carbam+Pheno+Pheny, Pheny+Primid, Pheno+Primid, Pheno+Pheny+Primid, Valpro, Pheny+Valpro | mixed public & private |
| Dean, 2002[42] [CR: Rasalam, 2005[43]] | Scotland | Aberdeen Maternity Hospital | 1976-2000 | Major CMs, Fetal Losses, Cardiac CMs, Cleft Lip or Palate, Club Foot, Hypospadias, Inguinal Hernia, Undescended Testes | Control, Pheny, Pheno, Pheno+Pheny, Ethos, Carbam, Carbam+Pheny, Carbam+Pheno, Primid, Valpro, Pheno+Valpro, Carbam+Valpro | NR |
| Delmis, 1991[44] | Croatia | Zagreb University Clinic | 1978-1989 | Major CMs, Minor CMs, Cardiac CMs, Cleft Lip or Palate | Control, Pheno+Pheny, Carbam, Primid | NR |
| Dessens, 1996[45, 46] | Netherlands | Academic Medical Center, University of Amsterdam | 1957-1972 | Major CMs, Cardiac CMs, Cleft Lip or Palate, Club Foot, Hypospadias, Inguinal Hernia, Undescended Testes | Pheno, Pheno+Pheny | mixed public & private |
| D'Souza, 1991[47] | UK | St. Mary's Hospital | 1980-1982 | Major CMs, Cardiac CMs, Cleft Lip or Palate, Club Foot, Hypospadias, Inguinal Hernia, Undescended Testes | Control, Pheno, Carbam, Valpro | public |
| Eroglu, 2008[48] | Turkey | Gulhane Hospital | 1996-2006 | Major CMs, Cardiac CMs, Cleft Lip or Palate, Club Foot, Hypospadias, Inguinal Hernia, Undescended Testes | Pheny, Pheno, Carbam, Valpro | NR |
| Fairgrieve, 2000[49] | UK | Hospitals in the Northern health region | 1997-1998 | Major CMs, Cardiac CMs, Cleft Lip or Palate, Club Foot, Hypospadias, Inguinal Hernia | Control, Carbam, Valpro | private |
| Farmen, 2015[50] | Norway | 0 | 1989-2012 | Pre-natal Growth | Carbam, Control, Lamot, Levet, Topir, Valpro | mixed public & private |
| Fedrick, 1973[51] | UK | Oxford Record Linkage Study | 1966-1970 | Major CMs, Fetal Losses, Cardiac CMs, Cleft Lip or Palate, Club Foot, Hypospadias, Inguinal Hernia, Undescended Testes | Control, Pheny, Pheno, Pheno+Pheny | NR |
| Gaily, 1990[52] [CR: Gaily, 1990[53]; Hiilesma, 1982[54]; Hiilesma, 1985[55]] | Finland | Helsinki University | 1975-1979 | Major CMs, Fetal Losses, Pre-natal Growth, Cardiac CMs, Cleft Lip or Palate, Club Foot, Hypospadias, Inguinal Hernia, Undescended Testes | Control, Pheny, Pheno+Pheny, Ethos+Pheny, Carbam, Carbam+Pheny, Carbam+Pheno+Pheny, Pheny+Primid, Carbam+Clonaz, Pheny+Valpro, Carbam+Valpro | mixed public & private |
| Hernandez-Diaz, 2012[56] | USA; Canada | North American AED Pregnancy Registry | 1997-2011 | Major CMs, Cardiac CMs, Cleft Lip or Palate, Club Foot, Hypospadias, Inguinal Hernia, Undescended Testes | Pheny, Pheno, Carbam, Clonaz, Valpro, Gabap, Lamot, Topir, Oxcar, Levet | private |
| Hernandez-Diaz, 2014[57] | US; Canada | North American AED Pregnancy Registry | 1997-2012 | Preterm Birth, Pre-natal Growth | Lamot, Topir | private |
| Hill, 1982[58] | USA | Baylor College of Medicine | NR | Major CMs | Pheny, Pheno, Pheno+Pheny | NR |
| Holmes, 2001[59] | USA | 5 hospitals in the Boston area | 1986-1993 | Major CMs, Cardiac CMs, Cleft Lip or Palate, Club Foot, Hypospadias, Inguinal Hernia, Undescended Testes | Control, Pheny, Pheno, Carbam | public |
| Hunter, 1990[60] | UK | Freedom Fields Maternity Hospital | 1982-1988 | Major CMs, Cardiac CMs, Cleft Lip or Palate, Club Foot, Hypospadias, Inguinal Hernia, Undescended Testes | Control, Pheny, Pheno, Carbam, Valpro | NR |
| Hvas, 2000[61] | Denmark | Aarhus University Hospital | 1989-1997 | Major CMs, Cardiac CMs, Cleft Lip or Palate, Club Foot, Hypospadias, Inguinal Hernia, Undescended Testes | Control, Carbam, Clonaz, Valpro, Oxcar | public |
| Jacobsen, 2014[62] | Denmark | Danish Medical Birth Registry and Prescription Database of the Central and North Denmark Regions | 1973-NR | Major CMs, Cardiac CMs, Cleft Lip or Palate, Hypospadias, Inguinal Hernia | Carbam, Valpro, Carbam+Valpro, Vigab, Oxcar, Oxcar+Valpro | private |
| Janz, 1964[63] | Germany | University of Heidelberg Neurological Hospital | 1952-1963 | Major CMs, Preterm Birth, Fetal Losses, Cardiac CMs, Cleft Lip or Palate, Club Foot, Hypospadias, Inguinal Hernia, Undescended Testes | Control, Pheny, Pheno, Ethos, Primid, Oxcar | NR |
| Jones, 1989[64] | USA | California Teratogen Registry | 1979-1988 | Major CMs, Fetal Losses, Pre-natal Growth, Cardiac CMs, Cleft Lip or Palate, Club Foot, Hypospadias, Inguinal Hernia, Undescended Testes | Carbam, Carbam+Pheno, Carbam+Primid, Carbam+Pheno+Valpro | public |
| Juarez-Olguin, 2008a[65] | Mexico | Instituto Nacional de Perinatología hospital | 1988-1992, 1996-2003 | Major CMs, Cardiac CMs, Cleft Lip or Palate, Club Foot, Hypospadias, Inguinal Hernia, Undescended Testes | Pheny, Carbam+Pheny, Carbam+Clonaz+Pheny | NR |
| Juarez-Olguin, 2008b[65] | Mexico | Instituto Nacional de Perinatología hospital | 1988-1992, 1996-2003 | Major CMs, Cardiac CMs, Cleft Lip or Palate, Club Foot, Hypospadias, Inguinal Hernia, Undescended Testes | Pheny, Carbam, Valpro, Pheny+Valpro, Carbam+Valpro | NR |
| Kaabi, 2013[66] | Tunisia | Tunisian National Center of Pharmacovigilance | 2010 | Major CMs, Pre-natal Growth, Cardiac CMs, Cleft Lip or Palate, Club Foot, Hypospadias, Inguinal Hernia, Undescended Testes | Pheno, Carbam, Carbam+Pheno, Valpro, Carbam+Valpro | NR |
| Kaaja, 2003[67] | Finland | Helsinki University Central Hospital | 1980-1998 | Major CMs, Club Foot, Hypospadias, Inguinal Hernia | Control, Pheny, Pheno, Ethos, Carbam, Primid, Clonaz, Valpro, Oxcar | public |
| Kallen, 2013[68] [CR: Wide, 2004[69]] | Sweden | Swedish Medical Birth Register | 1996-2011 | Major CMs | Pheny, Pheno, Ethos, Carbam, Primid, Clonaz, Valpro, Gabap, Vigab, Lamot, Topir, Oxcar, Levet | NR |
| Kaneko, 1999[70] [CR: Kaneko, 1988[71]; Kaneko, 1986[72]] | Japan; Italy; Canada | Multiple hospitals (in 6 cities in Japan, Italy and Canada) | 1978-1991 | Major CMs, Cardiac CMs, Cleft Lip or Palate, Club Foot, Hypospadias, Inguinal Hernia, Undescended Testes | Pheno+Pheny, Carbam+Pheny, Carbam+Pheno, Carbam+Pheno+Pheny, Pheny+Primid, Pheno+Primid, Pheno+Pheny+Primid, Carbam+Primid, Carbam+Pheny+Primid, Carbam+Pheno+Pheny+Primid, Pheny+Valpro, Pheno+Valpro, Pheno+Pheny+Valpro, Primid+Valpro, Pheny+Primid+Valpro, Carbam+Valpro, Carbam+Pheny+Valpro | public |
| Kelly, 1984[73] | USA | Epilepsy clinics and private neurology clinics, University of Virginia | 1977-1980 | Major CMs, Pre-natal Growth, Cardiac CMs, Cleft Lip or Palate, Club Foot, Hypospadias, Inguinal Hernia, Undescended Testes | Control, Pheny, Pheno, Pheno+Pheny, Ethos, Carbam, Valpro | public |
| Kilic, 2014[74] [CR: Molgaard-Nielsen, 2011[75]] | Denmark | Danish Medical Birth Registry | 1997-2008 | Preterm Birth, Pre-natal Growth | Carbam, Cloba, Clona, Control, Gabap, Lamot, Levet, Oxcar, Pheno, Pheny, Primid, Topir, Valpro, Vigab | NR |
| Kini, 2006[76] | UK | Central Manchester Maternity Hospital and regional epilepsy clinics in Manchester and Liverpool | 1989-1999 | Major CMs, Pre-natal Growth, Cardiac CMs, Cleft Lip or Palate, Club Foot, Hypospadias, Inguinal Hernia | Control, Pheny, Pheno, Carbam, Primid, Valpro, Lamot | NR |
| Koch, 1992[77] [CR: Jager-Roman, 1986[78]; Koch, 1983[79]; Koch, 1996[80]] | Germany | NR | 1976-1990 | Major CMs, Cardiac CMs, Cleft Lip or Palate, Club Foot, Hypospadias, Inguinal Hernia, Undescended Testes | Control, Pheny, Pheno, Carbam, Primid, Valpro | public |
| Koch, 1996[80] | Germany | NR | 1976-1983 | Preterm Birth | Pheno, Pheny, Primid, Valpro | public |
| Kochen, 2011[81] | Argentina | Ramos Mejía Hospital | 2002-2009 | Major CMs, Fetal Losses | Carbam, Valpro | NR |
| Kulaga, 2011[82] | Canada | Quebec Pregnancy Registry | 1998-2003 | Major CMs, Preterm Birth, Fetal Losses, Pre-natal Growth, Cardiac CMs, Cleft Lip or Palate, Club Foot, Hypospadias, Inguinal Hernia, Undescended Testes | Control, Pheny, Pheno, Carbam, Clonaz, Valpro, Lamot, Clobaz | public |
| Laine-Cessac, 1995[83] | France | Angers Hospital | 1988-1992 | Major CMs, Fetal Losses, Cardiac CMs, Cleft Lip or Palate, Club Foot, Hypospadias, Inguinal Hernia | Pheno, Carbam, Carbam+Pheno, Valpro, Pheno+Valpro | NR |
| Lowe, 1973[84] | UK | Cardiff Births Study | 1965-1971 | Major CMs, Cardiac CMs, Cleft Lip or Palate, Club Foot, Hypospadias, Inguinal Hernia, Undescended Testes | Control, Pheny, Pheno, Pheno+Pheny, Ethos+Pheno, Primid, Pheny+Primid, Pheno+Primid | public |
| Markestad, 1984[85] | Norway | Bergen University Hospital | 1980-1983 | Major CMs, Preterm Birth, Minor CMs, Cardiac CMs, Cleft Lip or Palate, Club Foot, Hypospadias, Inguinal Hernia, Undescended Testes | Pheny, Pheno, Pheno+Pheny, Carbam, Carbam+Pheno, Carbam+Clonaz, Valpro | NR |
| Martinez Ferri, 2009[86] [CR: Tomson, 2011[87]; Tomson, 2015[88]] | Spain | Spanish EURAP Registry | 2001-2007 | Major CMs, Cardiac CMs, Cleft Lip or Palate, Club Foot, Hypospadias, Inguinal Hernia, Undescended Testes | Pheno, Carbam, Valpro, Lamot | NR |
| Mawer, 2002[89] | England | Manchester Royal Infirmary | 1990-1999 | Fetal Losses | Carbam, Lamot, Pheny, Valpro | NR |
| Mawer, 2010[90] | England | 11 NHS hospitals in the Liverpool and Manchester regions | 2000-2006 | Major CMs, Preterm Birth, Pre-natal Growth, Cardiac CMs, Cleft Lip or Palate, Club Foot, Hypospadias, Inguinal Hernia | Control, Pheny, Carbam, Valpro, Gabap, Lamot | mixed public & private |
| Meador, 2006[91] | USA; UK | 25 epilepsy centers in the USA and UK | 1999-2004 | Major CMs, Fetal Losses, Cardiac CMs, Cleft Lip or Palate, Club Foot, Hypospadias, Inguinal Hernia, Undescended Testes | Pheny, Carbam, Valpro, Lamot | public |
| Meischenguiser, 2004[92] | Argentina | Epilepsy Pregnancy Registry of Argentina | 1995-2002 | Major CMs, Preterm Birth, Fetal Losses, Minor CMs, Cardiac CMs, Cleft Lip or Palate, Club Foot, Hypospadias, Undescended Testes | Pheno, Carbam, Clonaz, Valpro, Clobaz, Oxcar, Clonaz+Oxcar, Clobaz+Oxcar | public |
| Melchior, 1967[93] | Denmark | University of Copenhagen hospital | 1962-1965 | Major CMs, Cardiac CMs, Cleft Lip or Palate, Club Foot, Hypospadias, Inguinal Hernia, Undescended Testes | Pheno, Pheno+Pheny, Pheny+Primid, Primid | NR |
| Meyer, 1973[94] | Germany | NR | NR | Preterm Birth | Carbam+Pheny, Carbam+Primid, Control, Ethos, Ethos+Primid, Pheno, Pheno+Pheny, Pheny, Pheny+Primid, Primid | private |
| Millar, 1973[95] | Ireland | Royal Victoria Hospital | 1969-NR | Major CMs, Cardiac CMs, Cleft Lip or Palate, Club Foot, Hypospadias, Inguinal Hernia, Undescended Testes | Pheny, Pheno, Pheno+Pheny, Ethos+Pheny, Primid, Pheny+Primid, Pheno+Primid, Pheno+Pheny+Primid | NR |
| Miskov, 2010[96] | Croatia | NR | 2003-2010 | Major CMs, Preterm Birth, Fetal Losses, Pre-natal Growth, Cardiac CMs, Cleft Lip or Palate, Club Foot, Hypospadias, Inguinal Hernia | Control, Carbam, Valpro, Gabap, Lamot | NR |
| Miyakoshi, 1984[97] | Japan | Shizuka Hhigashi Hospital | 1975-1983 | Major CMs, Cardiac CMs, Cleft Lip or Palate, Club Foot, Hypospadias, Inguinal Hernia, Undescended Testes | Control, Pheno, Carbam, Valpro | NR |
| Montouris, 2003[98] | USA | Gabapentin (Neurontin) Pregnancy Registry | NR | Major CMs, Cardiac CMs, Cleft Lip or Palate, Club Foot, Hypospadias, Inguinal Hernia, Undescended Testes | Gabap, Gabap+Pheny, Carbam+Gabap, Gabap+Lamot | NR |
| Nadebaum, 2011[99] | Australia | Australian Registry of Antiepileptic Drug Use in Pregnancy | 2007-2009 | Preterm Birth | Carbam, Lamot, Valpro | mixed public & private |
| Nulman, 1997[100] [CR: Gladstone, 1992[101]; Scolnik, 1994[102]] | Canada | Hospital for Sick Children, North York General Hospital, Toronto Western Hospital, Oshawa General Hospital | 1987-1992 | Major CMs, Fetal Losses, Minor CMs, Cardiac CMs, Cleft Lip or Palate, Club Foot, Hypospadias, Inguinal Hernia, Undescended Testes | Pheny, Carbam | mixed public & private |
| Oguni, 1992[103] | Canada | Montreal Neurological Hospital | 1982-1989 | Major CMs, Fetal Losses, Cardiac CMs, Cleft Lip or Palate, Hypospadias, Inguinal Hernia | Control, Pheny, Pheno, Pheno+Pheny, Carbam, Carbam+Pheny, Valpro, Pheny+Valpro, Pheno+Valpro, Carbam+Valpro | NR |
| Omtzigt, 1992[104, 105] | Netherlands | University Hospital Rotterdam Dijkzigt | 1985-1990 | Major CMs, Cardiac CMs, Cleft Lip or Palate, Club Foot, Hypospadias, Inguinal Hernia, Undescended Testes | Pheny, Pheno, Ethos, Carbam, Carbam+Pheny, Carbam+Pheno, Carbam+Clonaz, Valpro, Carbam+Valpro, Clobaz | NR |
| Ornoy, 2008[106] | Israel | Israeli Teratogen Information Service | 1996-2006 | Major CMs | Control, Topir | NR |
| Pennell, 2012[107] | USA, UK | Neurodevelopmental Effects of Antiepileptic Drugs Study Group | 1999-2004 | Preterm Birth, Pre-natal Growth | Carbam, Lamot, Pheny, Valpro | mixed public & private |
| Pittschieler, 2008[108] | Austria | Innsbruck Medical University | 1971-2004 | Preterm Birth, Fetal Losses | Carbam, Valpro | NR |
| Porter, 1984[109] [CR: Bertollini, 1987[110]] | France | 23 doctors in the French Chapter of the International League Against Epilepsy (ILAE) | NR | Major CMs, Cardiac CMs, Cleft Lip or Palate, Club Foot, Hypospadias, Inguinal Hernia, Undescended Testes | Control, Pheny, Pheno, Carbam, Valpro | NR |
| Rating, 1987[111] | Germany | NR | 1976-1984 | Major CMs, Cardiac CMs, Cleft Lip or Palate, Club Foot, Hypospadias, Inguinal Hernia, Undescended Testes | Control, Pheny, Pheno, Pheno+Pheny, Carbam, Carbam+Pheny, Primid, Pheny+Primid, Valpro, Pheny+Valpro, Pheno+Valpro, Primid+Valpro | NR |
| Regesta, 1996[112] | Italy | San Martino Hospital | 1980-1995 | Major CMs, Minor CMs | Pheno, Pheno+Pheny, Carbam, Carbam+Pheno, Carbam+Pheno+Pheny, Valpro, Pheno+Valpro | NR |
| Rihtman, 2013[113] | Israel | Israeli Teratogen Information Service | 2001-2006 | Major CMs, Cardiac CMs, Cleft Lip or Palate, Club Foot, Hypospadias, Inguinal Hernia, Undescended Testes | Valpro, Lamot | mixed public & private |
| Robert, 1986[114] | France | Rhône-Alpes birth defects monitoring registry and a neurological hospital | 1976-1983 | Major CMs, Fetal Losses, Minor CMs, Cardiac CMs, Cleft Lip or Palate, Club Foot, Hypospadias, Inguinal Hernia, Undescended Testes | Pheno+Pheny, Pheno+Valpro, Carbam+Pheno+Valpro | NR |
| Samren, 1999[115] | Netherlands | 28 hospitals in 4 provinces | 1972-1992 | Major CMs, Cardiac CMs, Cleft Lip or Palate, Club Foot, Hypospadias, Inguinal Hernia | Pheny, Pheno, Pheno+Pheny, Ethos, Carbam, Carbam+Pheno, Primid, Pheno+Primid, Clonaz, Carbam+Clonaz, Valpro, Carbam+Valpro, Carbam+Pheny+Valpro, Clonaz+Valpro, Clobaz, Oxcar | public |
| Sawhney, 1996[116] | India | Nehru Hospital | 1987-1994 | Major CMs, Cardiac CMs, Cleft Lip or Palate, Club Foot, Hypospadias, Inguinal Hernia, Undescended Testes | Control, Pheny, Pheno, Pheno+Pheny, Carbam, Carbam+Pheny, Carbam+Pheno | NR |
| Sonneveld, 1990[117] | Australia | Tasmanian Obstetric and Perinatal Audit | 1981-1988 | Major CMs, Fetal Losses, Cardiac CMs, Cleft Lip or Palate, Club Foot, Hypospadias, Inguinal Hernia, Undescended Testes | Control, Pheny, Carbam | NR |
| Steegers-Theunissena, 1994[118] | Netherlands | 5 hospitals | NR | Major CMs, Fetal Losses, Cardiac CMs, Cleft Lip or Palate, Club Foot, Hypospadias, Inguinal Hernia, Undescended Testes | Control, Pheny, Pheno, Carbam, Valpro | public |
| Tanaka, 1991[119] | Japan | Nagoya National Hospital | 1982-1990 | Major CMs, Preterm Birth, Fetal Losses, Cardiac CMs, Cleft Lip or Palate, Club Foot, Hypospadias, Inguinal Hernia, Undescended Testes | Pheny, Pheno, Carbam, Valpro | NR |
| Thomas, 2008[120] | India | Kerala Registry of Epilepsy and Pregnancy | 1998-2004 | Cardiac CMs | Control, Pheny, Pheno, Carbam, Valpro | public |
| Tomson, 2011[87] [CR: Martinez Ferry, 2009[121]; Kochen, 2011[81]; Vajda, 2014[122]] | 42 countries | International Registry of Antiepileptic Drugs and Pregnancy (EURAP) | 1999-2010 | Major CMs | Pheno, Carbam, Primid, Valpro, Lamot, Lamot+Valpro, Clobaz, Topir, Oxcar, Levet | private |
| Torres, 1995[123] | Mexico | Dr. Manuel Gea Gonzáles General Hospital | 1989-1992 | Major CMs, Cardiac CMs, Cleft Lip or Palate, Club Foot, Hypospadias, Inguinal Hernia, Undescended Testes | Pheny, Carbam, Pheny+Primid | NR |
| Vajda, 2014[122] [CR: Eadie, 2005[124]; Vajda, 2010[125]; Vajda, 2012[126]; Vajda, 2010[127]; Vajda, 2012[128]; Vajda, 2013[129]; Vajda, 2013[130]] | Australia | Australian Register of Antiepileptic Drugs in Pregnancy (APR) | 1998-2013 | Major CMs | Control, Pheny, Ethos, Carbam, Clonaz, Valpro, Gabap, Lamot, Topir, Oxcar, Levet | mixed public & private |
| Van der Pol, 1991[131] | Netherlands | Groningen University Hospital | 1973-1981 | Major CMs, Preterm Birth, Fetal Losses, Pre-natal Growth, Cardiac CMs, Cleft Lip or Palate, Club Foot, Hypospadias, Inguinal Hernia, Undescended Testes | Control, Pheno, Carbam, Carbam+Pheno | public |
| Vanya, 2015[132] | Hungary | Albert Szent-Gyorgyi Medical Health Centre, University of Szeged | 2000-2014 | Major CMs, Cardiac CMs, Hypospadias | Control, Carbam, Valpro, Carbam+Valpro, Lamot, Carbam+Lamot, Lamot+Valpro, Lamot+Levet | public |
| Veiby, 2014[133] | Norway | Medical Birth Registry of Norway | 1999-2011 | Major CMs, Pre-natal Growth, Cardiac CMs, Cleft Lip or Palate, Hypospadias | Control, Pheno, Carbam, Clonaz, Valpro, Lamot, Topir, Oxcar, Levet | NR |
| Viinikainen, 2006[134] | Finland | Kuopio University Hospital regional pregnancy registry | 1989-2000 | Major CMs, Club Foot, Hypospadias, Inguinal Hernia, Undescended Testes | Control, Pheny, Carbam, Carbam+Pheny, Carbam+Clonaz, Valpro, Carbam+Valpro, Carbam+Vigab, Oxcar | private |
| Wide, 2000[135] | Sweden | Sodersjukuset Hospital | 1985-1995 | Minor CMs | Pheny, Carbam | mixed public & private |
| Wladimiroff, 1988[136] | Netherlands | NR | 1982-1987 | Major CMs, Fetal Losses, Cardiac CMs, Cleft Lip or Palate, Club Foot, Hypospadias, Inguinal Hernia, Undescended Testes | Pheny, Pheno, Carbam, Valpro | NR |

*Fetal Losses in this table refers to the combined fetal loss outcome.

**Abbreviations:** CM – congenital malformations; NR – not reported

## Appendix E. List of studies and their patient characteristics

| **Author, Year** | **Sample Size^a^** | **Indication** | **Mean Age (years)** | **AED Exposure Timing** | **Folic Acid Use** | **Alcohol use** | **Tobacco Use** |
| --- | --- | --- | --- | --- | --- | --- | --- |
| Abe, 2014[1] | 96 | Epilepsy | NR | 1st trimester^b^ | NR | NR | NR |
| Al Bunyan, 1999[2] | 72 | Epilepsy | 28 | NR | NR | NR | NR |
| Annegers, 1974[3] [CR: Annegers, 1978[4]; Annegers, 1983[5]; Annegers, 1975[6]] | 166 | Epilepsy | NR | 1st trimester | NR | NR | NR |
| Arkilo, 2015[7] | 59 | Epilepsy | NR | 1st trimester | NA | NR | NR |
| Artama, 2005[8] [CR: Artama, 2011[9]; Artama, 2004[10]] | 2144 | Epilepsy | 27.9 | 1st trimester | NR | NR | NR |
| Artama, 2013[11] | 4348 | Epilepsy | NR | whole PG | NA | NR | 878/4867 (18%) |
| Arteaga-Vazquez, 2012[12] | 202 | Epilepsy | NR | NR | NR | NR | NR |
| Babic, 2014[13] | 25 | Epilepsy | 26.4 | NR | Preconceptual folic acid use: 100% | 0/25 | NR |
| Bag, 1989[14] | 30 | Epilepsy | 24 | 1st trimester | NR | NR | NR |
| Banhidy, 2011[15] [CR: Czeizel, 1992[16]; Czeizel, 2011[17]; Puho, 2007[18]] | 97 | Epilepsy | NR | NR | Folic acid use: 42% | NR | NR |
| Barqawi, 2005[19] | 50 | Epilepsy | NR | whole PG | NR | NR | NR |
| Barroso, 2015[20] | 44 | Epilepsy | 27.4 | NR | NR | NR | NR |
| Battino, 1999[21] [CR: Battino, 1992[22]] | 430 | Epilepsy | NR | 1st trimester | NA | NR | NR |
| Bertollini, 1987a[23] | 45 | Epilepsy | NR | 1st trimester | NR | NR | NR |
| Bertollini, 1987b[23] | 229 | Epilepsy | NR | 1st trimester | NR | NR | NR |
| Campbell, 2013[24-27] [CR: Campbell, 2013[28]; Morrow, 2009[29]; Ban, 2015[30]] | 6037 | Epilepsy | 29.2 | 1st trimester | Preconceptual folic acid use: 49% | NR | NR |
| Canger, 1997[31] [CR: Bossi, 1983[32]; Molteni, 1992[33]] | 396 | Epilepsy | 27.8 | NR | NR | NR | NR |
| Canger, 1999[34] [CR: Battino, 1992[35]] | 337 | Epilepsy | 27.6 | NR | NA | NR | NR |
| Cassina, 2013a[36] | 223 | Epilepsy | NR | 1st trimester | NR | NR | NR |
| Cassina, 2013b[36] | 130 | Mental illness | NR | 1st trimester | NR | NR | NR |
| Charlton, 2011[37] | 1536 | Epilepsy | 30 | 1st trimester | NR | NR | NR |
| Cummings, 2011[38] [CR: Tomson, 2015[39]] | 142 | Epilepsy | NR | whole PG | NA | NR | 19/108 (17.5%) |
| Dansky, 1982[40] | 58 | Epilepsy | NR | 1st trimester | NR | NR | NR |
| Dansky, 1989[41] | 97 | Epilepsy | 26 | 1st trimester | NR | NR | NR |
| Dean, 2002[42] [CR: Rasalam, 2005[43]] | 287 | Epilepsy | 27 | 1st trimester | NR | NR | NR |
| Delmis, 1991[44] | 62 | Epilepsy | 26 | NR | NR | NR | NR |
| Dessens, 1996[45, 46] | 191 | Epilepsy | NR | Pheno vs. Pheno+Pheny: 45% vs. 73% in 1st trimester, respectively | NR | NR | NR |
| D'Souza, 1991[47] | 19 | Epilepsy | 26.5 | 1st trimester | NR | NR | NR |
| Eroglu, 2008[48] | 80 | Epilepsy | 26.1 | NR | NR | NR | NR |
| Fairgrieve, 2000[49] | 231 | Epilepsy | NR | NR | NR | NR | NR |
| Farmen, 2015[50] | 253 | Epilepsy | 27.8 | whole PG | NA | NR | 52/253 |
| Fedrick, 1973[51] | 143 | Epilepsy | NR | 1st trimester | NR | NR | NR |
| Gaily, 1990[52] [CR: Gaily, 1990[53]; Hiilesma, 1982[54]; Hiilesma, 1985[55]] | 136 | Epilepsy | 27.8 | 1st 20 weeks | NR | NR | NR |
| Hernandez-Diaz, 2012[56] | 4733 | Epilepsy^c^ | 29.9 | 1st trimester | Folic acid use at last menstrual period: 71% | 1237/4733 (26%) | NR |
| Hernandez-Diaz, 2014[57] | 1928 | Epilepsy | NR | whole PG | NA | NR | 188/1928 (9.8%) |
| Hill, 1982[58] | 47 | Epilepsy | NR | NR | NR | NR | NR |
| Holmes, 2001[59] | 307 | Epilepsy | NR | 1st trimester | NR | NR | NR |
| Hunter, 1990[60] | 67 | Epilepsy | 24.8 | NR | NR | NR | NR |
| Hvas, 2000[61] | 177 | Epilepsy | NR | 1st trimester | NR | NR | NR |
| Jacobsen, 2014[62] | 155 | NR | NR | NR | NR | NR | NR |
| Janz, 1964[63] | 353 | Epilepsy | NR | NR | NR | NR | NR |
| Jones, 1989[64] | 46 | Epilepsy | NR | whole PG | NR | NR | NR |
| Juarez-Olguin, 2008a[65] | 74 | Epilepsy | NR | 1st trimester | NR | NR | NR |
| Juarez-Olguin, 2008b[65] | 170 | Epilepsy | NR | 1st trimester | NR | NR | NR |
| Kaabi, 2013[66] | 18 | Mixed indications | 32 | 1st trimester | NR | NR | NR |
| Kaaja, 2003[67] | 822 | Epilepsy | NR | 1st trimester | NR | NR | NR |
| Kallen, 2013[68] [CR: Wide, 2004[69]] | 3849 | NR | NR | 1st trimester | NR | NR | NR |
| Kaneko, 1999[70] [CR: Kaneko, 1988[71]; Kaneko, 1986[72]] | 280 | Epilepsy | 27 | 1st trimester | NR | NR | NR |
| Kelly, 1984[73] | 134 | Epilepsy | NR | NR | NR | NR | NR |
| Kilic, 2014[74] [CR: Molgaard-Nielsen, 2011[75]] | 7759 | Epilepsy | NR | whole PG | NA | NR | 790/2928 (27%) |
| Kini, 2006[76] | 297 | Epilepsy | NR | NR | NR | NR | NR |
| Koch, 1992[77] [CR: Jager-Roman, 1986[78]; Koch, 1983[79]; Koch, 1996[80]] | 97 | Epilepsy | NR | NR | NR | NR | NR |
| Koch, 1996[80] | 40 | Epilepsy | NR | 1st trimester | NA | NR | NR |
| Kochen, 2011[81] | 50 | Epilepsy | NR | 1st trimester | Folic acid use: 90% | NR | NR |
| Kulaga, 2011[82] | 257 | Epilepsy | 27.2 | 1st trimester | NR | NR | NR |
| Laine-Cessac, 1995[83] | 67 | Epilepsy | 27 | 1st trimester | NR | NR | NR |
| Lowe, 1973[84] | 245 | Epilepsy | NR | NR | NR | NR | NR |
| Markestad, 1984[85] | 51 | Epilepsy | NR | NR | NR | NR | NR |
| Martinez Ferri, 2009[86] [CR: Tomson, 2011[87]; Tomson, 2015[88]] | 240 | Epilepsy | 31 | 1st trimester | NR | NR | NR |
| Mawer, 2002[89] | 52 | Epilepsy | NR | NR | NA | NR | NR |
| Mawer, 2010[90] | 99 | Epilepsy | NR | 1st trimester | NR | NR | NR |
| Meador, 2006[91] | 333 | Epilepsy | 29 | 1st trimester | NR | NR | NR |
| Meischenguiser, 2004[92] | 100 | Epilepsy | 24.6 | 1st trimester | NR | NR | NR |
| Melchior, 1967[93] | 28 | Epilepsy | NR | NR | NR | NR | NR |
| Meyer, 1973[94] | 239 | Epilepsy | NR | whole PG | NA | NR | NR |
| Millar, 1973[95] | 105 | Epilepsy | NR | 1st trimester | NR | NR | NR |
| Miskov, 2010[96] | 55 | Epilepsy | NR | NR | Folic acid taken properly: 20% | NR | NR |
| Miyakoshi, 1984[97] | 37 | Epilepsy | NR | 1st trimester | NR | NR | NR |
| Montouris, 2003[98] | 30 | Epilepsy | 25 | 1st trimester | Folic acid at conception: 67% | NR | NR |
| Nadebaum, 2011[99] | 66 | Epilepsy | 31.6 | 1st trimester | NA | NR | 5/66 (7.5%) |
| Nulman, 1997[100] [CR: Gladstone, 1992[101]; Scolnik, 1994[102]] | 69 | Epilepsy | NR | 1st trimester | NR | NR | NR |
| Oguni, 1992[103] | 82 | Epilepsy | 27.1 | 1st trimester | Deficiency of serum folic acid level: 27% | 14/94(67 known) | 22/94 |
| Omtzigt, 1992[104, 105] | 252 | Epilepsy | NR | 1st trimester | NR | NR | NR |
| Ornoy, 2008[106] | 235 | NR | NR | 1st trimester | NR | NR | NR |
| Pennell, 2012[107] | 308 | Epilepsy | 29.8 | 1st trimester | NA | 24/302 | 37/302 (12%) |
| Pittschieler, 2008[108] | 236 | Epilepsy | 27 | 1st trimester | NA | 2/244 | 28/244 (11%) |
| Porter, 1984[109] [CR: Bertollini, 1987[110]] | 501 | Epilepsy | NR | 1st trimester | NR | NR | NR |
| Rating, 1987[111] | 133 | Epilepsy | NR | NR | NR | NR | NR |
| Regesta, 1996[112] | 154 | NR | 27.4 | NR | NR | NR | NR |
| Rihtman, 2013[113] | 72 | Epilepsy | 34.3 | 1st trimester | NR | NR | NR |
| Robert, 1986[114] | 29 | Epilepsy | NR | 1st trimester | NR | NR | NR |
| Samren, 1999[115] | 1095 | Epilepsy | NR | 1st trimester | NR | NR | NR |
| Sawhney, 1996[116] | 217 | Epilepsy | 25.14 | NR | NR | NR | NR |
| Sonneveld, 1990[117] | 240 | Epilepsy | NR | NR | NR | NR | NR |
| Steegers-Theunissena, 1994[118] | 98 | Epilepsy | 28.6 | 1st trimester | Folic acid use: 39% | NR | NR |
| Tanaka, 1991[119] | 82 | Epilepsy | 27.2 | whole PG | NR | NR | NR |
| Thomas, 2008[120] | 331 | Epilepsy | 25.6 | 1st trimester | Folic acid use: 74% | NR | NR |
| Tomson, 2011[87] [CR: Martinez Ferry, 2009[121]; Kochen, 2011[81]; Vajda, 2014[122]] | 4708 | Epilepsy | 29.7 | at conception | Appropriate use of folic acid: 36% | NR | NR |
| Torres, 1995[123] | 44 | Epilepsy | 24 | NR | NR | NR | NR |
| Vajda, 2014[122] [CR: Eadie, 2005[124]; Vajda, 2010[125]; Vajda, 2012[126]; Vajda, 2010[127]; Vajda, 2012[128]; Vajda, 2013[129]; Vajda, 2013[130]] | 1283 | Epilepsy | NR | 1st trimester | NR | NR | NR |
| Van der Pol, 1991[131] | 57 | Epilepsy | NR | NR | NR | NR | NR |
| Vanya, 2015[132] | 91 | Epilepsy | 29.4 | whole PG | NR | NR | NR |
| Veiby, 2014[133] | 5987 | Epilepsy | NR | NR | Folic acid use preconceptually: 35.9%; Folic acid use during 1st trimester: 73.3 % | NR | 439/2086 (23.8) |
| Viinikainen, 2006[134] | 173 | Epilepsy | 29 | whole PG | NR | NR | NR |
| Wide, 2000[135] | 60 | Epilepsy | 29 | whole PG | NR | NR | NR |
| Wladimiroff, 1988[136] | 122 | NR | NR | NR | NR | NR | NR |

**Abbreviations:** AED – antiepileptic drugs; NR – not reported; PG - pregnancy

a This is the sample size included in our analyses.

b Indicates that most patients were exposed to the AED at least in the first trimester of pregnancy.

c Clonazepam arm was Mixed Indications instead (n=64).

## Appendix F. Risk of bias for randomized controlled trials – Cochrane risk-of-bias tool

| **First Author, Year** | **Selection Bias** | | **Performance Bias** | **Detection Bias** | **Attrition Bias** | **Reporting Bias** | **Other Bias** |
| --- | --- | --- | --- | --- | --- | --- | --- |
|  | **Random sequence generation** | **Allocation concealment** | **Blinding of participants and personnel** | **Blinding of outcome assessment** | **Incomplete outcome data** | **Selective reporting** | **Other Bias** |
| Barqawi, 2005[19] | High | High | Low | Low | Low | Unclear | Unclear |

## Appendix G. Methodological quality of case-control studies – Newcastle Ottawa Scale

| **First Author, Year** | **Representativeness of the exposed cohort** | **Selection of the non-exposed cohort** | **Ascertainment of exposure** | **Demonstration that outcome of interest was not present at start of study** | **Comparability of cohorts on the basis of the design or analysis** | **Assessment of outcome** | **Was follow-up long enough for outcomes to occur** | **Adequacy of follow up of cohorts** |
| --- | --- | --- | --- | --- | --- | --- | --- | --- |
| Arteaga-Vazquez, 2012[12] | A | A | A | A | D | A | A | A |
| Banhidy, 2011[15] | A | A | A | A | A | A | A | A |
| Pittschieler, 2008[108] | B | A | A | A | D | A | A | D |

**Abbreviations:** A – low risk; B – moderate risk; C – high risk; D – unclear risk

## Appendix H. Methodological quality of observational cohort studies – Newcastle Ottawa Scale

| **First Author, Year** | **Representativeness of the exposed cohort** | **Selection of the non-exposed cohort** | **Ascertainment of exposure** | **Demonstration that outcome of interest was not present at start of study** | **Comparability of cohorts on the basis of the design or analysis** | **Assessment of outcome** | **Was follow-up long enough for outcomes to occur** | **Adequacy of follow up of cohorts** |
| --- | --- | --- | --- | --- | --- | --- | --- | --- |
| Abe, 2014[1] | B | A | A | A | D | A | A | D |
| Al Bunyan, 1999[2] | B | A | A | A | D | A | A | D |
| Annegers, 1974[3] | B | B | A | A | D | A | A | A |
| Arkilo, 2015[7] | B | A | B | A | D | A | A | C |
| Artama, 2005[8] | A | A | A | A | B | B | A | B |
| Artama, 2013[11] | A | A | A | A | A | B | A | A |
| Babic, 2014[13] | B | A | A | A | D | A | A | D |
| Bag, 1989[14] | B | A | A | A | D | A | A | A |
| Barroso, 2015[20] | B | A | A | A | D | A | A | B |
| Battino, 1999[21] | A | A | A | A | A | A | A | D |
| Bertollini, 1987a[23] | A | A | A | A | D | A | A | A |
| Bertollini, 1987b[23] | A | A | A | A | D | A | A | A |
| Campbell, 2013[24-27] | A | A | A | A | A | A | A | D |
| Canger, 1997[31] | D | A | A | A | D | A | A | A |
| Canger, 1999[34] | B | A | A | A | D | A | A | B |
| Cassina, 2013a[36] | A | A | B | A | D | A | A | D |
| Cassina, 2013b[36] | A | A | B | A | D | A | A | D |
| Charlton, 2011[37] | A | A | A | A | D | A | A | B |
| Cummings, 2011[38] | A | A | A | A | A | A | A | C |
| Dansky, 1982[40] | B | A | A | A | D | A | A | D |
| Dansky, 1989[41] | B | A | D | A | D | A | A | A |
| Dean, 2002[42] | B | A | A | A | D | A | A | C |
| Delmis, 1991[44] | B | A | A | A | D | A | A | D |
| Dessens, 1996[45, 46] | B | A | A | A | D | A | A | B |
| D'Souza, 1991[47] | B | A | A | A | D | A | A | A |
| Eroglu, 2008[48] | B | A | A | A | D | A | A | A |
| Fairgrieve, 2000[49] | B | A | A | A | D | A | A | C |
| Farmen, 2015[50] | B | A | B | A | C | A | A | B |
| Fedrick, 1973[51] | B | A | A | A | D | B | A | A |
| Martinez Ferri, 2009[86] | A | A | A | A | D | A | A | C |
| Gaily, 1990[52] | B | A | A | A | D | A | A | A |
| Hernandez-Diaz, 2012[56] | A | A | A | A | A | A | A | D |
| Hernandez-Diaz, 2014[57] | A | A | A | A | A | A | A | C |
| Hill, 1982[58] | D | C | D | A | D | A | A | D |
| Holmes, 2001[59] | B | A | B | A | C | A | A | C |
| Hunter, 1990[60] | B | A | A | A | D | A | A | D |
| Hvas, 2000[61] | B | A | B | A | A | A | A | D |
| Jacobsen, 2014[62] | A | A | A | A | D | A | A | B |
| Janz, 1964[63] | B | A | D | A | D | C | A | B |
| Jones, 1989[64] | A | A | B | A | D | A | A | B |
| Juarez-Olguin, 2008a[65] | B | A | A | A | D | A | A | D |
| Juarez-Olguin, 2008b[65] | B | A | A | A | D | A | A | D |
| Kaabi, 2013[66] | B | A | A | A | D | A | A | D |
| Kaaja, 2003[67] | B | A | D | A | C | A | A | B |
| Kallen, 2013[68] | A | A | A | A | B | A | A | D |
| Kaneko, 1999[70] | A | A | A | A | A | A | A | D |
| Kelly, 1984[73] | B | A | A | A | D | A | A | B |
| Kilic, 2014[74] | A | A | A | A | A | B | A | C |
| Kini, 2006[76] | B | A | A | A | D | A | A | D |
| Koch, 1992[77] | D | A | A | A | D | A | A | D |
| Koch, 1996[80] | B | A | B | A | D | A | A | C |
| Kochen, 2011[81] | B | A | A | A | D | A | A | C |
| Kulaga, 2011[82] | A | A | A | A | D | A | A | D |
| Laine-Cessac, 1995[83] | B | A | A | A | D | A | A | A |
| Lowe, 1973[84] | B | A | A | A | D | A | A | A |
| Markestad, 1984[85] | B | A | D | A | D | A | A | D |
| Mawer, 2002[89] | B | A | A | A | D | A | A | A |
| Mawer, 2010[90] | B | A | A | A | D | A | A | B |
| Meador, 2006[91] | A | A | A | A | A | A | A | B |
| Meischenguiser, 2004[92] | A | A | A | A | D | A | A | D |
| Melchior, 1967[93] | B | A | A | A | D | A | A | A |
| Meyer, 1973[94] | D | A | C | A | D | C | A | D |
| Millar, 1973[95] | B | A | D | A | D | A | A | D |
| Miskov, 2010[96] | D | A | D | A | D | D | A | D |
| Miyakoshi, 1984[97] | A | A | D | A | D | A | A | D |
| Montouris, 2003[98] | D | A | A | A | D | A | A | D |
| Nadebaum, 2011[99] | A | A | A | A | A | A | A | B |
| Nulman, 1997[100] | B | A | A | A | D | A | A | A |
| Oguni, 1992[103] | B | A | A | A | D | A | A | C |
| Omtzigt, 1992[104, 105] | B | A | A | A | D | A | A | D |
| Ornoy, 2008[106] | A | A | D | A | D | A | A | D |
| Pennell, 2012[107] | A | A | A | A | A | A | A | B |
| Porter, 1984[109] | A | A | A | A | D | A | A | B |
| Rating, 1987[111] | D | A | D | A | D | A | A | C |
| Regesta, 1996[112] | D | A | D | A | D | A | A | A |
| Rihtman, 2013[113] | A | B | A | A | A | A | A | C |
| Robert, 1986[114] | B | A | C | A | D | A | A | C |
| Samren, 1999[115] | A | A | A | A | D | A | A | D |
| Sawhney, 1996[116] | B | A | D | A | D | A | A | C |
| Sonneveld, 1990[117] | A | A | A | A | D | A | A | D |
| Steegers-Theunissena, 1994[118] | A | A | A | A | D | A | A | D |
| Tanaka, 1991[119] | B | A | A | A | D | A | A | A |
| Thomas, 2008[120] | A | A | A | A | D | A | A | C |
| Tomson, 2011[87] | A | A | A | A | A | A | A | C |
| Torres, 1995[123] | B | A | D | A | D | A | A | C |
| Vajda, 2014[122] | A | A | A | A | D | A | A | D |
| van der Pol, 1991[131] | B | A | D | A | A | A | A | B |
| Vanya, 2015[132] | B | A | A | A | D | D | A | A |
| Veiby, 2014[133] | A | A | A | A | A | A | A | B |
| Viinikainen, 2006[134] | B | A | A | A | D | A | A | A |
| Wide, 2000[135] | B | A | A | A | D | A | A | C |
| Wladimiroff, 1988[136] | D | C | D | A | D | A | A | D |

**Abbreviations:** A – low risk; B – moderate risk; C – high risk; D – unclear risk

## Appendix I. Comparison adjusted funnel plot for each outcome^a^


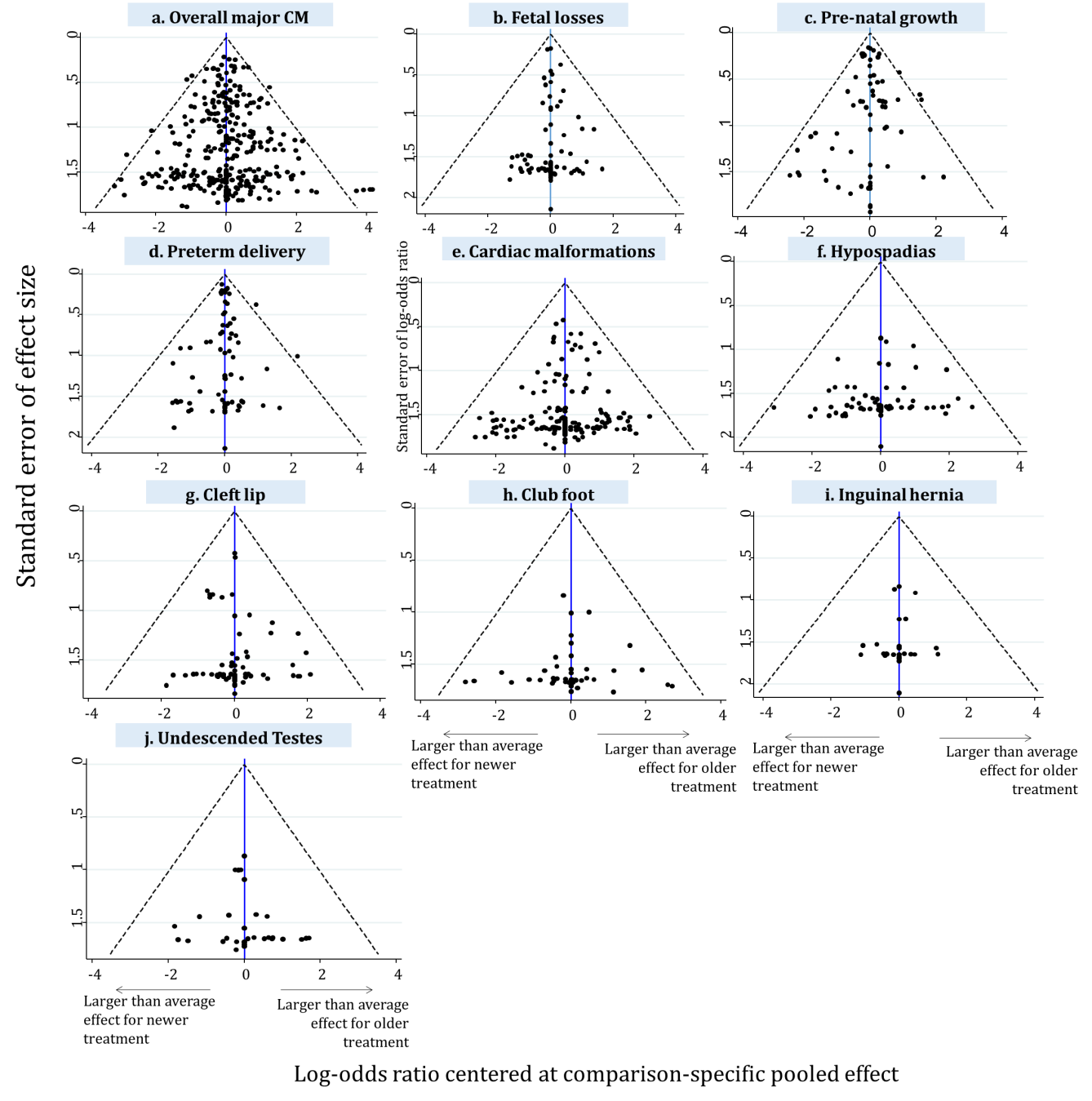


a No funnel plot is presented for any minor congenital malformations (CMs) outcome due to the small number of studies (k=9) in the network meta-analysis.

## Appendix J. Statistically significant network meta-analysis results along with meta-analysis results, transitivity, and consistency assessment^a^

| **Treatment Comparison** | **Number of Studies**  **(Mean Baseline Risk)** | | **Number of patients**  **(Mean Age)** | **Treatment Indication** | **Timing** | **Comparability of cohorts ROB** | | **Adequacy of follow up of cohorts ROB** | | **MA Odds Ratio**  **(95% CrI)** | **NMA Odds Ratio  (95% CrI) (95% PrI)** |
| --- | --- | --- | --- | --- | --- | --- | --- | --- | --- | --- | --- |
| **Overall Major Congenital Malformations (78 studies, 35016 cases, 48 treatments )** | | | | | | | | | | | |
| Carbam vs Control | | 35 (0.06) | 11283 (27.59) | Epilepsy | 1st trimester | | H | H | 1.43 (1.11-1.81) | | 1.37 (1.10-1.71) (0.86-2.24) |
| Carbam vs Pheno | | 38 (NA) | 6654 (27.23) | Epilepsy | 1st trimester | | H | H | 0.75 (0.55-1.06) | | 0.75 (0.57-0.99) (0.45-1.27) |
| Carbam vs Pheno+Pheny | | 14 (NA) | 994 (26.49) | Epilepsy | 1st trimester | | H | H | 0.80 (0.46-1.39) | | 0.62 (0.44-0.88) (0.36-1.08) |
| Carbam+Clonaz vs Carbam | | 5 (NA) | 622 (28.40) | Epilepsy | 1st trimester | | H | H | 4.78 (1.57-13.26) | | 6.07 (2.16-15.41) (1.99-16.92) |
| Carbam+Clonaz vs Clonaz | | 1 (NA) | 17 (NR) | Epilepsy | 1st trimester | | H | H | 6.24  (0.09-3.48 x 10^3^) | | 7.55 (2.35-22.18) (2.09-24.26) |
| Carbam+Clonaz vs Control | | 2 (0.03) | 76 (28.40) | Epilepsy | Whole PG | | H | L | 5.61 (0.17-528.70) | | 8.32 (2.93-20.86) (2.70-22.68) |
| Carbam+Clonaz vs Pheno | | 3 (NA) | 215 (NR) | Epilepsy | 1st trimester | | H | H | 3.77 (0.90-13.81) | | 4.55 (1.62-11.82) (1.46-12.87) |
| Carbam+Clonaz vs Pheno+Pheny | | 3 (NA) | 103 (27.80) | Epilepsy | 1st trimester | | H | H | 4.05 (0.78-17.68) | | 3.69 (1.31-10.02) (1.23-10.80) |
| Carbam+Clonaz vs Pheny | | 5 (NA) | 269 (28.40) | Epilepsy | 1st trimester | | H | H | 8.78 (1.80-34.06) | | 4.93 (1.78-12.49) (1.60-13.61) |
| Carbam+Clonaz vs Primid | | 1 (NA) | 26 (NR) | Epilepsy | 1st trimester | | H | H | 14.33  (0.32-4.39 x 10^4^) | | 6.95 (2.14-19.69) (2.03-20.71) |
| Carbam+Lamot vs Carbam+Clonaz | | NA | NR | NR | NR | | NR | NR | NA | | 0.05 (0.00-0.76) (0.00-0.82) |
| Carbam+Lamot vs Pheno+Pheny+Primid | | NA | NR | NR | NR | | NR | NR | NA | | 0.03 (0.00-0.58) (0.00-0.59) |
| Carbam+Lamot vs Pheno+Primid | | NA | NR | NR | NR | | NR | NR | NA | | 0.03 (0.00-0.55) (0.00-0.57) |
| Carbam+Pheno vs Carbam | | 12 (NA) | 890 (27.22) | Epilepsy | 1st trimester | | H | H | 2.12 (0.98-4.39) | | 2.01 (1.04-3.48) (0.92-4.05) |
| Carbam+Pheno vs Control | | 4 (0.09) | 109 (26.71) | Epilepsy | 1st trimester | | H | H | 1.22 (0.30-4.85) | | 2.74 (1.41-5.06) (1.23-5.64) |
| Carbam+Pheny vs Carbam | | 11 (NA) | 559 (26.86) | Epilepsy | 1st trimester | | H | H | 2.55 (1.24-5.48) | | 2.59 (1.48-4.63) (1.30-5.32) |
| Carbam+Pheny vs Control | | 9 (0.05) | 364 (27.34) | Epilepsy | NR | | H | H | 4.57 (1.99-10.41) | | 3.55 (2.01-6.36) (1.78-7.39) |
| Carbam+Pheny vs Pheno | | 6 (NA) | 139 (26.81) | Epilepsy | 1st trimester | | H | H | 1.74 (0.55-6.17) | | 1.95 (1.06-3.57) (0.96-4.06) |
| Carbam+Pheny vs Pheny | | 11 (NA) | 405 (26.86) | Epilepsy | 1st trimester | | H | H | 3.01 (1.42-6.26) | | 2.12 (1.20-3.86) (1.06-4.40) |
| Carbam+Pheny+primid vs Pheno+Pheny+Primid | | 1 (NA) | 45 (27.00) | Epilepsy | NR | | L | H | 0.19 (0.02-1.02) | | 0.17 (0.02-0.95) (0.02-1.02) |
| Carbam+Pheny+primid vs Pheno+Primid | | 1 (NA) | 26 (27.00) | Epilepsy | NR | | L | H | 0.23 (0.02-4.58) | | 0.16 (0.02-0.94) (0.01-0.99) |
| Carbam+Primid vs Pheno+Pheny+Primid | | 1 (NA) | 31 (27.00) | Epilepsy | NR | | L | H | 0.14 (0.00-2.00) | | 0.11 (0.00-0.97) (0.00-1.04) |
| Carbam+Valpro vs Carbam | | 10 (NA) | 973 (28.72) | Epilepsy | 1st trimester | | H | H | 3.29 (1.72-6.07) | | 4.27 (2.51-7.07) (2.21-8.45) |
| Carbam+Valpro vs Carbam+Pheno | | 5 (NA) | 170 (28.67) | Epilepsy | 1st trimester | | H | H | 3.22 (1.19-10.87) | | 2.14 (1.02-4.58) (0.93-5.14) |
| Carbam+Valpro vs Clonaz | | 1 (NA) | 67 (NR) | Epilepsy | 1st trimester | | H | H | 2.77 (0.20-380.10) | | 5.17 (2.42-12.19) (2.26-13.04) |
| Carbam+Valpro vs Control | | 5 (0.02) | 164 (28.06) | Epilepsy | NR | | H | L | 16.28 (5.03-60.99) | | 5.88 (3.32-9.98) (3.01-11.77) |
| Carbam+Valpro vs Pheno | | 5 (NA) | 340 (28.70) | Epilepsy | 1st trimester | | H | H | 2.85 (0.97-6.82) | | 3.22 (1.81-5.44) (1.61-6.29) |
| Carbam+Valpro vs Pheno+Pheny | | 5 (NA) | 256 (27.23) | Epilepsy | 1st trimester | | H | H | 3.01 (1.24-7.10) | | 2.64 (1.44-4.68) (1.28-5.36) |
| Carbam+Valpro vs Pheny | | 7 (NA) | 413 (27.73) | Epilepsy | 1st trimester | | H | H | 8.13 (3.33-21.88) | | 3.49 (1.98-5.99) (1.73-6.96) |
| Carbam+Valpro vs Primid | | 2 (NA) | 85 (27.00) | Epilepsy | 1st trimester | | H | H | 6.13 (0.90-424.40) | | 4.77 (2.23-10.51) (2.07-11.67) |
| Carbam+Valpro vs Valpro | | 9 (NA) | 515 (28.90) | Epilepsy | 1st trimester | | H | H | 1.64 (0.73-3.12) | | 1.99 (1.14-3.34) (1.02-3.91) |
| Clobaz+Oxcar vs Carbam+Clonaz | | NA | NR | NR | NR | | NR | NR | NA | | 0.05 (0.00-0.80) (0.00-0.81) |
| Clobaz+Oxcar vs Pheno+Pheny+Primid | | NA | NR | NR | NR | | NR | NR | NA | | 0.04 (0.00-0.68) (0.00-0.69) |
| Clobaz+Oxcar vs Pheno+Primid | | NA | NR | NR | NR | | NR | NR | NA | | 0.04 (0.00-0.63) (0.00-0.68) |
| Clonaz vs Carbam+Pheno | | 1 (NA) | 48 (NR) | Epilepsy | 1st trimester | | H | H | 0.69 (0.00-30.64) | | 0.42 (0.17-0.95) (0.15-1.03) |
| Clonaz vs Carbam+Pheny | | NA | NR | NR | NR | | NR | NR | NA | | 0.32 (0.14-0.68) (0.12-0.74) |
| Clonaz vs Pheno+Pheny | | 1 (NA) | 76 (NR) | Epilepsy | 1st trimester | | H | H | 1.32 (0.01-44.07) | | 0.51 (0.25-0.98) (0.23-1.09) |
| Clonaz vs Pheno+Pheny+Primid | | NA | NR | NR | NR | | NR | NR | NA | | 0.11 (0.04-0.29) (0.03-0.31) |
| Clonaz vs Pheno+Primid | | 1 (NA) | 23 (NR) | Epilepsy | 1st trimester | | H | H | 0.11 (0.00-2.71) | | 0.10 (0.04-0.29) (0.03-0.31) |
| Clonaz vs Pheny+Primid | | NA | NR | NR | NR | | NR | NR | NA | | 0.22 (0.09-0.61) (0.08-0.67) |
| Clonaz+Oxcar vs Carbam+Clonaz | | NA | NR | NR | NR | | NR | NR | NA | | 0.04 (0.00-0.97) (0.00-1.02) |
| Clonaz+Oxcar vs Pheno+Pheny+Primid | | NA | NR | NR | NR | | NR | NR | NA | | 0.03 (0.00-0.67) (0.00-0.68) |
| Clonaz+Oxcar vs Pheno+Primid | | NA | NR | NR | NR | | NR | NR | NA | | 0.03 (0.00-0.65) (0.00-0.70) |
| Clonaz+Valpro vs Control | | NA | NR | NR | NR | | NR | NR | NA | | 11.16 (1.13-70.53) (1.11-72.20) |
| Ethos vs Control | | 7 (0.10) | 1182 (28.10) | Epilepsy | 1st trimester | | H | L | 2.86 (0.87-8.87) | | 3.04 (1.23-7.07) (1.11-7.68) |
| Gabap vs Carbam+Clonaz | | NA | NR | NR | NR | | NR | NR | NA | | 0.12 (0.03-0.39) (0.03-0.43) |
| Gabap vs Carbam+Pheno | | NA | NR | NR | NR | | NR | NR | NA | | 0.36 (0.15-0.92) (0.13-1.02) |
| Gabap vs Carbam+Pheny | | NA | NR | NR | NR | | NR | NR | NA | | 0.27 (0.11-0.66) (0.10-0.71) |
| Gabap vs Carbam+Valpro | | NA | NR | NR | NR | | NR | NR | NA | | 0.17 (0.07-0.38) (0.06-0.41) |
| Gabap vs Clonaz+Valpro | | NA | NR | NR | NR | | NR | NR | NA | | 0.09 (0.01-0.93) (0.01-0.93) |
| Gabap vs Ethos | | 3 (NA) | 192 (29.20) | Epilepsy | 1st trimester | | L | L | 0.35 (0.06-2.29) | | 0.33 (0.11-0.99) (0.10-1.05) |
| Gabap vs Pheno+Pheny | | NA | NR | NR | NR | | NR | NR | NA | | 0.45 (0.20-0.92) (0.18-1.02) |
| Gabap vs Pheno+Pheny+Primid | | NA | NR | NR | NR | | NR | NR | NA | | 0.09 (0.03-0.26) (0.03-0.27) |
| Gabap vs Pheno+Primid | | NA | NR | NR | NR | | NR | NR | NA | | 0.09 (0.03-0.27) (0.03-0.28) |
| Gabap vs Pheno+Valpro | | NA | NR | NR | NR | | NR | NR | NA | | 0.20 (0.07-0.64) (0.06-0.67) |
| Gabap vs Pheny+Primid | | NA | NR | NR | NR | | NR | NR | NA | | 0.19 (0.07-0.58) (0.07-0.62) |
| Gabap vs Pheny+Valpro | | NA | NR | NR | NR | | NR | NR | NA | | 0.20 (0.06-0.63) (0.05-0.66) |
| Gabap vs Valpro | | 6 (NA) | 2835 (29.75) | Epilepsy | 1st trimester | | H | H | 0.31 (0.14-0.58) | | 0.34 (0.16-0.61) (0.14-0.70) |
| Lamot vs Carbam | | 15 (NA) | 12356 (29.58) | Epilepsy | 1st trimester | | H | H | 0.80 (0.62-1.00) | | 0.70 (0.54-0.88) (0.42-1.10) |
| Lamot vs Carbam+Clonaz | | NA | NR | NR | NR | | NR | NR | NA | | 0.11 (0.04-0.33) (0.04-0.35) |
| Lamot vs Carbam+Pheno | | NA | NR | NR | NR | | NR | NR | NA | | 0.35 (0.19-0.69) (0.16-0.79) |
| Lamot vs Carbam+Pheny | | NA | NR | NR | NR | | NR | NR | NA | | 0.27 (0.14-0.49) (0.12-0.55) |
| Lamot vs Carbam+Pheny+Valpro | | NA | NR | NR | NR | | NR | NR | NA | | 0.15 (0.03-0.98) (0.03-1.03) |
| Lamot vs Carbam+Valpro | | 1 (NA) | 17 (29.40) | Epilepsy | Whole PG | | H | L | 0.22 (0.00-4.60) | | 0.16 (0.09-0.30) (0.08-0.33) |
| Lamot vs Clonaz+Valpro | | NA | NR | NR | NR | | NR | NR | NA | | 0.09 (0.01-0.84) (0.01-0.91) |
| Lamot vs Ethos | | 3 (NA) | 3517 (29.20) | Epilepsy | 1st trimester | | L | L | 0.43 (0.14-2.31) | | 0.31 (0.14-0.78) (0.13-0.88) |
| Lamot vs Pheno | | 7 (NA) | 3901 (28.75) | Epilepsy | 1st trimester | | H | H | 0.32 (0.19-0.56) | | 0.53 (0.37-0.74) (0.30-0.87) |
| Lamot vs Pheno+Pheny | | NA | NR | NR | NR | | NR | NR | NA | | 0.43 (0.29-0.65) (0.24-0.78) |
| Lamot vs Pheno+Pheny+Primid | | NA | NR | NR | NR | | NR | NR | NA | | 0.09 (0.04-0.20) (0.04-0.22) |
| Lamot vs Pheno+Primid | | NA | NR | NR | NR | | NR | NR | NA | | 0.08 (0.04-0.21) (0.03-0.23) |
| Lamot vs Pheno+Valpro | | NA | NR | NR | NR | | NR | NR | NA | | 0.20 (0.08-0.51) (0.07-0.53) |
| Lamot vs Pheny | | 8 (NA) | 5967 (29.17) | Epilepsy | 1st trimester | | H | H | 0.44 (0.30-0.67) | | 0.57 (0.42-0.76) (0.34-0.95) |
| Lamot vs Pheny+Primid | | NA | NR | NR | NR | | NR | NR | NA | | 0.19 (0.09-0.42) (0.08-0.46) |
| Lamot vs Pheny+Valpro | | NA | NR | NR | NR | | NR | NR | NA | | 0.19 (0.07-0.52) (0.07-0.55) |
| Lamot vs Valpro | | 17 (NA) | 9806 (29.44) | Epilepsy | 1st trimester | | H | H | 0.31 (0.25-0.39) | | 0.33 (0.25-0.41) (0.20-0.53) |
| Lamot+Levet vs Pheno+Primid | | NA | NR | NR | NR | | NR | NR | NA | | 0.06 (0.00-0.92) (0.00-0.97) |
| Lamot+Valpro vs Carbam+Clonaz | | NA | NR | NR | NR | | NR | NR | NA | | 0.11 (0.01-0.73) (0.01-0.76) |
| Lamot+Valpro vs Carbam+Valpro | | 1 (NA) | 27 (29.40) | Epilepsy | Whole PG | | H | L | 0.07 (0.00-1.23) | | 0.16 (0.02-0.75) (0.02-0.80) |
| Lamot+Valpro vs Pheno+Pheny+Primid | | NA | NR | NR | NR | | NR | NR | NA | | 0.09 (0.01-0.50) (0.01-0.53) |
| Lamot+Valpro vs Pheno+Primid | | NA | NR | NR | NR | | NR | NR | NA | | 0.08 (0.01-0.46) (0.01-0.49) |
| Levet vs Carbam | | 5 (NA) | 6243 (29.75) | Epilepsy | 1st trimester | | L | L | 0.59 (0.34-0.94) | | 0.53 (0.32-0.82) (0.27-0.94) |
| Levet vs Carbam+Clonaz | | NA | NR | NR | NR | | NR | NR | NA | | 0.09 (0.03-0.27) (0.03-0.28) |
| Levet vs Carbam+Pheno | | NA | NR | NR | NR | | NR | NR | NA | | 0.26 (0.12-0.60) (0.11-0.65) |
| Levet vs Carbam+Pheno+Pheny | | NA | NR | NR | NR | | NR | NR | NA | | 0.22 (0.07-0.94) (0.06-0.99) |
| Levet vs Carbam+Pheny | | NA | NR | NR | NR | | NR | NR | NA | | 0.20 (0.09-0.42) (0.08-0.45) |
| Levet vs Carbam+Pheny+Valpro | | NA | NR | NR | NR | | NR | NR | NA | | 0.12 (0.02-0.77) (0.02-0.83) |
| Levet vs Carbam+Valpro | | NA | NR | NR | NR | | NR | NR | NA | | 0.12 (0.06-0.25) (0.05-0.28) |
| Levet vs Clonaz+Valpro | | NA | NR | NR | NR | | NR | NR | NA | | 0.06 (0.01-0.65) (0.01-0.66) |
| Levet vs Ethos | | 3 (NA) | 471 (29.20) | Epilepsy | 1st trimester | | L | L | 0.26 (0.05-1.52) | | 0.24 (0.09-0.66) (0.09-0.71) |
| Levet vs Pheno | | 3 (NA) | 868 (30.29) | Epilepsy | 1st trimester | | L | H | 0.34 (0.15-0.73) | | 0.40 (0.23-0.66) (0.20-0.74) |
| Levet vs Pheno+Pheny | | NA | NR | NR | NR | | NR | NR | NA | | 0.33 (0.18-0.57) (0.16-0.65) |
| Levet vs Pheno+Pheny+Primid | | NA | NR | NR | NR | | NR | NR | NA | | 0.07 (0.03-0.16) (0.02-0.18) |
| Levet vs Pheno+Primid | | NA | NR | NR | NR | | NR | NR | NA | | 0.06 (0.02-0.17) (0.02-0.18) |
| Levet vs Pheno+Valpro | | NA | NR | NR | NR | | NR | NR | NA | | 0.15 (0.06-0.39) (0.05-0.42) |
| Levet vs Pheny | | 4 (NA) | 1572 (29.75) | Epilepsy | 1st trimester | | L | H | 0.42 (0.22-0.78) | | 0.43 (0.25-0.70) (0.22-0.81) |
| Levet vs Pheny+Primid | | NA | NR | NR | NR | | NR | NR | NA | | 0.14 (0.06-0.35) (0.06-0.38) |
| Levet vs Pheny+Valpro | | NA | NR | NR | NR | | NR | NR | NA | | 0.14 (0.05-0.42) (0.05-0.46) |
| Levet vs Topir | | 6 (NA) | 1585 (28.63) | Epilepsy | 1st trimester | | L | H | 0.40 (0.21-0.75) | | 0.38 (0.20-0.69) (0.18-0.77) |
| Levet vs Valpro | | 6 (NA) | 3847 (28.63) | Epilepsy | 1st trimester | | L | H | 0.20 (0.12-0.36) | | 0.24 (0.15-0.39) (0.13-0.45) |
| Oxcar vs Carbam+Clonaz | | 2 (NA) | 15 (29.00) | Epilepsy | NR | | H | H | 0.85 (0.03-42.40) | | 0.16 (0.05-0.49) (0.05-0.53) |
| Oxcar vs Carbam+Pheny | | 1 (NA) | 6 (29.00) | Epilepsy | Whole PG | | H | L | 3.02  (0.00-4.17 x 10^3^) | | 0.37 (0.17-0.80) (0.15-0.90) |
| Oxcar vs Carbam+Valpro | | 3 (NA) | 88 (29.00) | Epilepsy | NR | | H | L | 0.89 (0.04-7.80) | | 0.23 (0.10-0.49) (0.09-0.53) |
| Oxcar vs Pheno+Pheny+Primid | | NA | NR | NR | NR | | NR | NR | NA | | 0.12 (0.05-0.31) (0.04-0.33) |
| Oxcar vs Pheno+Primid | | 1 (NA) | 16 (NR) | Epilepsy | 1st trimester | | H | H | 0.40 (0.00-17.81) | | 0.11 (0.04-0.32) (0.04-0.34) |
| Oxcar vs Pheno+Valpro | | NA | NR | NR | NR | | NR | NR | NA | | 0.27 (0.10-0.71) (0.09-0.78) |
| Oxcar vs Pheny+Primid | | NA | NR | NR | NR | | NR | NR | NA | | 0.26 (0.11-0.67) (0.10-0.74) |
| Oxcar vs Pheny+Valpro | | NA | NR | NR | NR | | NR | NR | NA | | 0.26 (0.09-0.81) (0.08-0.86) |
| Oxcar vs Valpro | | 11 (NA) | 3545 (28.27) | Epilepsy | 1st trimester | | H | L | 0.44 (0.24-0.76) | | 0.45 (0.25-0.76) (0.22-0.88) |
| Pheno vs Control | | 24 (0.06) | 5795 (26.94) | Epilepsy | 1st trimester | | H | H | 1.50 (0.92-2.42) | | 1.83 (1.35-2.47) (1.09-3.06) |
| Pheno+Pheny vs Control | | 12 (0.06) | 765 (26.84) | Epilepsy | 1st trimester | | H | H | 2.62 (1.44-4.99) | | 2.21 (1.54-3.19) (1.26-3.94) |
| Pheno+Pheny+Primid vs Carbam | | 1 (NA) | 19 (26.00) | Epilepsy | 1st trimester | | H | L | 13.19 (2.17-137.50) | | 7.77 (3.61-17.15) (3.26-18.64) |
| Pheno+Pheny+Primid vs Carbam+Pheno | | 1 (NA) | 48 (27.00) | Epilepsy | NR | | L | H | 5.86 (1.04-69.47) | | 3.94 (1.56-10.00) (1.41-11.04) |
| Pheno+Pheny+Primid vs Carbam+Pheny | | 1 (NA) | 56 (27.00) | Epilepsy | NR | | L | H | 45.11  (2.48-2.97 x 10^8^) | | 3.01 (1.25-7.26) (1.14-7.95) |
| Pheno+Pheny+Primid vs Control | | NA | NR | NR | NR | | NR | NR | NA | | 10.62 (4.90-23.83) (4.38-26.64) |
| Pheno+Pheny+Primid vs Ethos | | NA | NR | NR | NR | | NR | NR | NA | | 3.42 (1.21-11.81) (1.11-13.27) |
| Pheno+Pheny+Primid vs Pheno | | 2 (NA) | 46 (26.00) | Epilepsy | 1st trimester | | H | H | 6.53 (1.22-50.34) | | 5.83 (2.67-13.23) (2.45-14.60) |
| Pheno+Pheny+Primid vs Pheno+Pheny | | 3 (NA) | 195 (26.50) | Epilepsy | 1st trimester | | H | H | 5.20 (2.19-12.31) | | 4.83 (2.31-10.47) (2.03-11.45) |
| Pheno+Pheny+Primid vs Pheny | | 2 (NA) | 56 (26.00) | Epilepsy | 1st trimester | | H | H | 4.04 (1.08-22.49) | | 6.27 (2.94-14.13) (2.70-15.60) |
| Pheno+Pheny+Primid vs Primid | | 1 (NA) | 20 (NR) | Epilepsy | 1st trimester | | H | H | 1.44 (0.05-638.80) | | 8.85 (3.37-23.83) (3.17-25.50) |
| Pheno+Primid vs Carbam | | 2 (NA) | 402 (26.00) | Epilepsy | 1st trimester | | H | H | 6.02 (1.80-21.65) | | 8.40 (3.44-19.52) (3.18-21.32) |
| Pheno+Primid vs Carbam+Pheno | | 2 (NA) | 82 (27.00) | Epilepsy | 1st trimester | | H | H | 4.49 (1.02-29.34) | | 4.21 (1.39-12.59) (1.27-13.46) |
| Pheno+Primid vs Carbam+Pheny | | 1 (NA) | 37 (27.00) | Epilepsy | NR | | L | H | 30.07  (1.20-4.70 x 10^4^) | | 3.28 (1.16-8.27) (1.06-9.08) |
| Pheno+Primid vs Control | | 1 (0.03) | 114 (NR) | Epilepsy | NR | | H | L | 2.62 (0.01-48.91) | | 11.52 (4.61-26.94) (4.15-30.35) |
| Pheno+Primid vs Ethos | | 1 (NA) | 23 (NR) | Epilepsy | 1st trimester | | H | H | 5.10  (0.30-1.68 x 10^3^) | | 3.81 (1.11-13.48) (1.04-14.71) |
| Pheno+Primid vs Pheno | | 4 (NA) | 265 (26.00) | Epilepsy | 1st trimester | | H | H | 9.00 (2.48-31.49) | | 6.30 (2.51-14.32) (2.30-16.31) |
| Pheno+Primid vs Pheno+Pheny | | 5 (NA) | 297 (26.50) | Epilepsy | 1st trimester | | H | H | 5.83 (2.09-16.05) | | 5.20 (2.02-11.97) (1.83-13.54) |
| Pheno+Primid vs Pheny | | 4 (NA) | 210 (26.00) | Epilepsy | 1st trimester | | H | H | 6.46 (1.78-29.90) | | 6.86 (2.69-15.77) (2.54-17.60) |
| Pheno+Primid vs Primid | | 3 (NA) | 43 (NR) | Epilepsy | 1st trimester | | H | H | 6.75 (0.99-83.31) | | 9.44 (3.35-27.28) (3.14-29.39) |
| Pheno+Valpro vs Carbam | | 5 (NA) | 139 (27.13) | Epilepsy | 1st trimester | | H | H | 4.18 (0.84-15.78) | | 3.61 (1.40-8.38) (1.32-9.28) |
| Pheno+Valpro vs Clonaz | | NA | NR | NR | NR | | NR | NR | NA | | 4.37 (1.55-11.94) (1.47-13.36) |
| Pheno+Valpro vs Control | | 3 (0.05) | 84 (27.05) | Epilepsy | 1st trimester | | H | H | 3.40 (0.42-20.66) | | 4.90 (1.98-11.37) (1.83-12.37) |
| Pheno+Valpro vs Pheno | | 5 (NA) | 191 (27.13) | Epilepsy | 1st trimester | | H | H | 3.81 (0.93-13.52) | | 2.71 (1.05-6.34) (0.96-7.19) |
| Pheno+Valpro vs Pheny | | 3 (NA) | 76 (27.05) | Epilepsy | 1st trimester | | H | H | 3.71 (0.54-20.61) | | 2.92 (1.12-6.86) (1.07-7.65) |
| Pheno+Valpro vs Primid | | 2 (NA) | 28 (27.00) | Epilepsy | 1st trimester | | H | H | 3.15 (0.04-153.00) | | 4.07 (1.46-11.05) (1.35-11.92) |
| Pheny vs Control | | 28 (0.07) | 3165 (27.45) | Epilepsy | 1st trimester | | H | L | 2.00 (1.42-2.86) | | 1.69 (1.30-2.17) (1.03-2.73) |
| Pheny+Primid vs Carbam | | 4 (NA) | 74 (25.93) | Epilepsy | 1st trimester | | H | H | 2.54 (0.54-11.75) | | 3.68 (1.66-7.48) (1.49-8.28) |
| Pheny+Primid vs Control | | 3 (0.06) | 172 (27.80) | Epilepsy | NR | | H | L | 1.89 (0.17-10.97) | | 5.03 (2.28-10.50) (2.05-11.59) |
| Pheny+Primid vs Pheno | | 5 (NA) | 115 (26.00) | Epilepsy | 1st trimester | | H | L | 2.06 (0.48-9.69) | | 2.77 (1.22-5.74) (1.12-6.37) |
| Pheny+Primid vs Pheno+Pheny | | 7 (NA) | 291 (26.93) | Epilepsy | 1st trimester | | H | L | 1.78 (0.74-4.28) | | 2.25 (1.04-4.64) (0.96-5.15) |
| Pheny+Primid vs Pheny | | 6 (NA) | 166 (25.93) | Epilepsy | 1st trimester | | H | H | 1.18 (0.35-3.80) | | 3.00 (1.33-6.18) (1.22-6.77) |
| Pheny+Primid vs Primid | | 4 (NA) | 51 (NR) | Epilepsy | 1st trimester | | H | H | 3.15 (0.39-39.80) | | 4.09 (1.64-10.90) (1.54-11.51) |
| Pheny+Valpro vs Carbam | | 5 (NA) | 193 (26.97) | Epilepsy | 1st trimester | | H | H | 5.30 (1.40-17.25) | | 3.68 (1.38-9.65) (1.25-10.32) |
| Pheny+Valpro vs Clonaz | | NA | NR | NR | NR | | NR | NR | NA | | 4.46 (1.41-13.67) (1.30-14.60) |
| Pheny+Valpro vs Control | | 3 (0.05) | 67 (27.45) | Epilepsy | 1st trimester | | H | H | 3.41 (0.40-25.05) | | 5.04 (1.96-13.07) (1.73-14.24) |
| Pheny+Valpro vs Pheno | | 3 (NA) | 22 (26.55) | Epilepsy | 1st trimester | | H | H | 2.14 (0.25-13.57) | | 2.75 (1.02-7.47) (0.96-8.01) |
| Pheny+Valpro vs Pheny | | 5 (NA) | 178 (26.97) | Epilepsy | 1st trimester | | H | H | 4.26 (1.28-15.30) | | 2.97 (1.16-8.04) (1.03-8.66) |
| Pheny+Valpro vs Primid | | 1 (NA) | 23 (NR) | Epilepsy | NR | | H | H | 118.50  (2.43-2.60 x 10^5^) | | 4.24 (1.33-11.59) (1.23-12.66) |
| Primid vs Carbam+Pheny | | 2 (NA) | 31 (27.00) | Epilepsy | 1st trimester | | H | H | 0.07 (0.00-0.93) | | 0.34 (0.15-0.78) (0.14-0.84) |
| Topir vs Carbam+Clonaz | | NA | NR | NR | NR | | NR | NR | NA | | 0.22 (0.08-0.70) (0.08-0.76) |
| Topir vs Carbam+Valpro | | NA | NR | NR | NR | | NR | NR | NA | | 0.32 (0.17-0.64) (0.15-0.72) |
| Topir vs Control | | 4 (0.03) | 4862 (29.20) | Epilepsy | 1st trimester | | H | L | 1.48 (0.61-3.00) | | 1.90 (1.17-2.97) (1.03-3.46) |
| Topir vs Lamot | | 6 (NA) | 6462 (28.63) | Epilepsy | 1st trimester | | L | H | 2.07 (1.29-3.21) | | 1.98 (1.24-3.13) (1.10-3.78) |
| Topir vs Pheno+Pheny+Primid | | NA | NR | NR | NR | | NR | NR | NA | | 0.18 (0.07-0.43) (0.07-0.46) |
| Topir vs Pheno+Primid | | NA | NR | NR | NR | | NR | NR | NA | | 0.16 (0.06-0.44) (0.06-0.47) |
| Topir vs Pheny+Primid | | NA | NR | NR | NR | | NR | NR | NA | | 0.37 (0.17-0.92) (0.15-0.99) |
| Topir vs Valpro | | 6 (NA) | 3402 (28.63) | Epilepsy | 1st trimester | | L | H | 0.57 (0.35-0.89) | | 0.65 (0.41-0.99) (0.34-1.18) |
| Valpro vs Carbam | | 50 (NA) | 12474 (28.01) | Epilepsy | 1st trimester | | H | H | 2.24 (1.88-2.67) | | 2.15 (1.79-2.57) (1.34-3.36) |
| Valpro vs Clonaz | | 12 (NA) | 3592 (27.82) | Epilepsy | 1st trimester | | H | H | 2.66 (1.53-4.99) | | 2.61 (1.49-4.95) (1.32-5.53) |
| Valpro vs Control | | 28 (0.07) | 9170 (27.91) | Epilepsy | 1st trimester | | H | H | 2.62 (2.04-3.38) | | 2.93 (2.36-3.69) (1.84-4.82) |
| Valpro vs Pheno | | 34 (NA) | 3599 (27.35) | Epilepsy | 1st trimester | | H | H | 1.43 (1.03-2.00) | | 1.62 (1.22-2.16) (0.97-2.65) |
| Valpro vs Pheno+Pheny+Primid | | 1 (NA) | 12 (26.00) | Epilepsy | 1st trimester | | H | L | 0.13 (0.01-2.05) | | 0.28 (0.12-0.59) (0.12-0.65) |
| Valpro vs Pheno+Primid | | 2 (NA) | 177 (26.00) | Epilepsy | 1st trimester | | H | H | 0.27 (0.06-1.14) | | 0.25 (0.11-0.63) (0.10-0.69) |
| Valpro vs Pheny | | 33 (NA) | 5077 (27.74) | Epilepsy | 1st trimester | | H | H | 2.06 (1.57-2.72) | | 1.75 (1.39-2.21) (1.10-2.89) |
| Valpro vs Primid | | 9 (NA) | 1249 (27.40) | Epilepsy | 1st trimester | | H | H | 2.67 (1.35-5.95) | | 2.42 (1.36-4.59) (1.21-5.08) |
| Vigab vs Pheno+Pheny+Primid | | NA | NR | NR | NR | | NR | NR | NA | | 0.21 (0.04-0.91) (0.04-0.99) |
| Vigab vs Pheno+Primid | | NA | NR | NR | NR | | NR | NR | NA | | 0.19 (0.04-0.90) (0.04-0.94) |
| Common between-study variance across treatment comparisons | | | | | | | | | 0.01 (0.00-0.06) | | 0.03 (0.00-0.13) (NA) |
| Residual deviance: 411  Data points: 468  DIC: 558 | |  | | | | | | | | | |
| Evaluation of consistency using the design-by-treatment interaction model | | Chi-square test: 247.8  Degrees of Freedom: 307  P- value: 0 .99  Heterogeneity: 0.000 | | | | | | | | | |
| **Combined Fetal Losses (31 studies, 13487 cases, 28 treatments)** | | | | | | | | | | | |
| Carbam+Pheno vs Pheno | | 5 (NA) | 180 (27.33) | Epilepsy | 1st trimester | | H | H | 2.60 (0.59-13.66) | | 2.93 (1.02-8.12) (0.91-9.02) |
| Carbam+Valpro vs Carbam | | 4 (NA) | 253 (27.30) | Epilepsy | 1st trimester | | H | H | 8.89 (1.66-46.07) | | 4.07 (1.11-12.31) (1.01-13.37) |
| Carbam+Valpro vs Control | | 3 (NA) | 74 (27.30) | Epilepsy | 1st trimester | | H | H | 9.12 (0.77-137.50) | | 5.09 (1.35-16.79) (1.28-18.41) |
| Carbam+Valpro vs Pheno | | 3 (NA) | 106 (27.05) | Epilepsy | 1st trimester | | H | H | 9.76 (1.13-105.60) | | 5.76 (1.43-19.24) (1.35-21.03) |
| Lamot vs Pheny+Valpro | | NA | NR | NR | NR | | NR | NR | NA | | 0.16 (0.04-0.73) (0.03-0.78) |
| Pheno+Pheny vs Pheno | | 6 (NA) | 270 (27.03) | Epilepsy | 1st trimester | | H | H | 2.57 (0.51-12.70) | | 3.14 (1.07-8.99) (1.00-10.14) |
| Pheny+Valpro vs Carbam | | 3 (NA) | 64 (26.97) | Epilepsy | 1st trimester | | H | L | 21.83 (2.78-352.00) | | 7.10 (1.58-27.25) (1.45-29.76) |
| Pheny+Valpro vs Control | | 3 (NA) | 38 (26.97) | Epilepsy | 1st trimester | | H | L | 10.92 (1.03-199.90) | | 8.96 (1.77-37.95) (1.63-41.47) |
| Pheny+Valpro vs Pheno | | 2 (NA) | 16 (26.55) | Epilepsy | 1st trimester | | H | H | 17.06 (0.72-1,045.00) | | 10.12 (2.02-41.18) (1.88-43.36) |
| Pheny+Valpro vs Pheny | | 3 (NA) | 109 (26.97) | Epilepsy | 1st trimester | | H | L | 6.59 (1.25-37.79) | | 5.98 (1.31-22.68) (1.27-24.55) |
| Pheny+Valpro vs Valpro | | 2 (NA) | 21 (26.55) | Epilepsy | 1st trimester | | H | H | 21.33 (1.11-961.50) | | 4.81 (1.07-19.02) (1.00-20.41) |
| Primid vs Control | | 3 (0.02) | 301 (27.30) | Epilepsy | 1st trimester | | H | L | 3.54 (1.45-9.46) | | 2.81 (1.21-6.28) (1.05-7.21) |
| Primid vs Pheno | | 3 (NA) | 310 (27.30) | Epilepsy | 1st trimester | | H | L | 3.91 (1.31-14.41) | | 3.13 (1.26-7.31) (1.13-8.39) |
| Topir vs Control | | NA | NR | NR | NR | | NR | NR | NA | | 23.58 (1.18-549.60) (1.10-567.20) |
| Topir vs Pheno | | NA | NR | NR | NR | | NR | NR | NA | | 26.64 (1.13-618.70) (1.14-628.80) |
| Valpro vs Carbam | | 21 (NA) | 6,312 (26.98) | Epilepsy | 1st trimester | | H | H | 1.48 (1.07-2.00) | | 1.48 (1.05-2.02) (0.78-2.71) |
| Valpro vs Control | | 10 (0.00) | 2,904 (27.38) | Epilepsy | 1st trimester | | H | H | 1.63 (0.73-3.66) | | 1.83 (1.04-3.46) (0.89-4.15) |
| Valpro vs Pheno | | 13 (NA) | 679 (27.31) | Epilepsy | 1st trimester | | H | H | 1.92 (0.86-4.66) | | 2.06 (1.08-3.84) (0.90-4.57) |
| Common between-study variance across treatment comparisons | | | | | | | | | 0.02 (0.00-0.12) | | 0.03 (0.00-0.27) (NA) |
| Residual deviance:131  Data points: 175  DIC: 198 | |  | | | | | | | | | |
| Evaluation of consistency using the design-by-treatment interaction model | | Chi-square test: 57.79  Degrees of Freedom: 108  P- value: 1  Heterogeneity: 0.000 | | | | | | | | | |
| **Pre-natal Growth Retardation (16 studies, 18117 cases, 23 treatments)** | | | | | | | | | | | |
| Clobaz vs Carbam | | 2 (NA) | 459 (NR) | Epilepsy | NR | | H | H | 3.68 (1.21-10.42) | | 3.87 (1.40-9.86) (1.14-12.37) |
| Clobaz vs Clonaz | | 2 (NA) | 281 (NR) | Epilepsy | NR | | H | H | 3.02 (0.96-8.92) | | 3.89 (1.25-11.54) (1.08-14.56) |
| Clobaz vs Control | | 2 (0.00) | 5,351 (NR) | Epilepsy | NR | | H | H | 5.58 (1.88-14.93) | | 4.47 (1.60-11.18) (1.30-14.17) |
| Clobaz vs Lamot | | 2 (NA) | 885 (NR) | Epilepsy | NR | | H | H | 3.52 (1.14-9.86) | | 4.97 (1.74-12.78) (1.46-16.08) |
| Clobaz vs Pheny | | 2 (NA) | 72 (NR) | Epilepsy | NR | | H | H | 3.14 (0.37-29.64) | | 6.62 (2.17-18.82) (1.84-22.74) |
| Clobaz vs Valpro | | 2 (NA) | 443 (NR) | Epilepsy | NR | | H | H | 3.54 (1.20-9.65) | | 3.49 (1.24-8.79) (0.98-11.03) |
| Lamot vs Pheno | | 2 (NA) | 953 (NR) | Epilepsy | NR | | H | H | 0.64 (0.26-1.71) | | 0.48 (0.26-0.88) (0.19-1.21) |
| Oxcar vs Clobaz | | 1 (NA) | 338 (NR) | Epilepsy | NR | | L | L | 0.25 (0.10-0.77) | | 0.22 (0.08-0.68) (0.06-0.80) |
| Oxcar vs Topir | | 1 (NA) | 360 (NR) | Epilepsy | NR | | L | L | 0.77 (0.26-2.91) | | 0.37 (0.19-0.80) (0.14-1.08) |
| Pheno vs Control | | 5 (0.00) | 5,598 (NR) | Epilepsy | NR | | L | L | 1.92 (0.96-3.81) | | 1.88 (1.07-3.32) (0.77-4.55) |
| Pheno vs Pheny | | 5 (NA) | 715 (32.00) | Epilepsy | NR | | H | H | 2.60 (1.23-5.61) | | 2.79 (1.49-5.34) (1.10-7.31) |
| Primid vs Pheny | | 2 (NA) | 157 (NR) | Epilepsy | NR | | L | L | 2.86 (0.76-10.59) | | 3.45 (1.19-9.12) (0.97-10.98) |
| Topir vs Carbam | | 2 (NA) | 1,164 (NR) | Epilepsy | NR | | L | L | 1.97 (0.96-3.85) | | 2.29 (1.30-3.92) (0.92-5.54) |
| Topir vs Clonaz | | 1 (NA) | 292 (NR) | Epilepsy | NR | | L | L | 0.84 (0.24-2.40) | | 2.28 (1.10-5.04) (0.84-6.67) |
| Topir vs Control | | 1 (0.00) | 5,328 (NR) | Epilepsy | NR | | L | L | 1.53 (0.43-3.91) | | 2.64 (1.41-4.63) (1.00-6.39) |
| Topir vs Lamot | | 2 (NA) | 2,827 (NR) | Epilepsy | NR | | H | L | 2.45 (1.27-4.29) | | 2.91 (1.67-4.97) (1.17-7.24) |
| Topir vs Pheny | | 1 (NA) | 58 (NR) | Epilepsy | NR | | L | L | 0.41 (0.03-12.24) | | 3.91 (1.81-8.20) (1.38-10.63) |
| Topir vs Valpro | | 1 (NA) | 396 (NR) | Epilepsy | NR | | L | L | 1.10 (0.30-3.12) | | 2.06 (1.09-3.66) (0.77-4.99) |
| Valpro vs Pheny | | 9 (NA) | 2,080 (NR) | Epilepsy | NR | | L | H | 2.17 (1.14-4.24) | | 1.90 (1.07-3.46) (0.78-4.93) |
| Common between-study variance across treatment comparisons | | | | | | | | | 0.09 (0.00-0.25) | | 0.09 (0.00-0.37) (NA) |
| Residual deviance:88  Data points: 89  DIC: 139 | |  | | | | | | | | | |
| Evaluation of consistency using the design-by-treatment interaction model | | Chi-square test: 54.94  Degrees of Freedom: 51  P- value: 0.32  Heterogeneity: NA^b^ | | | | | | | | | |
| **Preterm Birth (17 studies, 17133 cases, 23 treatments)** | | | | | | | | | | | |
| Clobaz vs Carbam | | 3 (NA) | 492 (NR) | Epilepsy | Whole PG | | H | H | 3.61 (1.33-9.41) | | 3.11 (1.22-7.30) (1.10-8.46) |
| Clobaz vs Clonaz | | 3 (NA) | 308 (NR) | Epilepsy | Whole PG | | H | H | 2.80 (0.94-8.05) | | 4.01 (1.54-10.77) (1.40-12.13) |
| Clobaz vs Control | | 2 (0.07) | 5371 (NR) | Epilepsy | NR | | H | H | 2.50 (0.80-6.68) | | 3.42 (1.42-7.92) (1.26-9.14) |
| Clobaz vs Lamot | | 2 (NA) | 918 (NR) | Epilepsy | NR | | H | H | 1.69 (0.52-4.80) | | 3.27 (1.31-7.77) (1.17-8.86) |
| Clobaz vs Pheny | | 2 (NA) | 71 (NR) | Epilepsy | NR | | H | H | 2.31 (0.27-23.65) | | 3.32 (1.15-9.13) (1.06-10.22) |
| Clobaz vs Valpro | | 3 (NA) | 465 (NR) | Epilepsy | Whole PG | | H | H | 3.94 (1.42-10.45) | | 3.60 (1.45-8.42) (1.29-9.59) |
| Clonaz vs Primid | | 1 (NA) | 282 (NR) | Epilepsy | Whole PG | | L | L | 0.52 (0.16-2.21) | | 0.40 (0.17-0.93) (0.15-1.04) |
| Levet vs Clobaz | | 1 (NA) | 56 (NR) | Epilepsy | Whole PG | | L | L | 0.24 (0.02-1.41) | | 0.25 (0.07-0.85) (0.06-0.90) |
| Oxcar vs Clobaz | | 2 (NA) | 377 (NR) | Epilepsy | Whole PG | | H | H | 0.20 (0.07-0.59) | | 0.23 (0.09-0.60) (0.08-0.67) |
| Oxcar vs Gabap | | 1 (NA) | 382 (NR) | Epilepsy | Whole PG | | L | L | 0.48 (0.21-1.15) | | 0.42 (0.19-0.95) (0.17-1.07) |
| Oxcar vs Primid | | 2 (NA) | 406 (NR) | Epilepsy | Whole PG | | L | H | 0.48 (0.13-2.33) | | 0.38 (0.17-0.86) (0.15-0.98) |
| Primid vs Control | | 3 (0.04) | 5653 (NR) | Epilepsy | Whole PG | | L | H | 2.11 (0.86-5.18) | | 2.12 (1.01-4.27) (0.89-4.94) |
| Valpro vs Primid | | 2 (NA) | 386 (NR) | Epilepsy | NR | | H | H | 0.30 (0.10-1.02) | | 0.45 (0.21-0.98) (0.19-1.12) |
| Common between-study variance across treatment comparisons | | | | | | | | | 0.08 (0.01-0.22) | | 0.03 (0.00-0.22) (NA) |
| Residual deviance:85  Data points: 97  DIC: 133 | |  | | | | | | | | | |
| Evaluation of consistency using the design-by-treatment interaction model | | Chi-square test: 38.66  Degrees of Freedom: 56  P- value: 0.96  Heterogeneity: 0 | | | | | | | | | |
| **Cardiac Malformations (51 studies, 21935 cases, 40 treatments)** | | | | | | | | | | | |
| Carbam vs Pheno | | 15 (NA) | 2848 (27.43) | Epilepsy | 1st trimester | | H | H | 0.48 (0.27-0.89) | | 0.60 (0.38-0.99) (0.34-1.09) |
| Carbam+Clonaz vs Carbam | | 1 (NA) | 121 (NR) | Epilepsy | 1st trimester | | H | H | 106.00  (2.90-4.17 x 10^4^) | | 10.82 (1.39-53.31) (1.37-55.17) |
| Carbam+Clonaz vs Control | | NA | NR | NR | NR | | NR | NR | NA | | 10.08 (1.40-51.22) (1.34-52.89) |
| Carbam+Clonaz vs Pheno+Pheny | | NA | NR | NR | NR | | NR | NR | NA | | 9.00 (1.11-47.96) (1.09-50.11) |
| Carbam+Clonaz vs Pheny | | 2 (NA) | 91 (27.80) | Epilepsy | 1st trimester | | H | H | 11.26 (0.94-105.20) | | 10.21 (1.32-50.44) (1.29-52.97) |
| Carbam+Clonaz vs Primid | | NA | NR | NR | NR | | NR | NR | NA | | 11.49 (1.29-82.68) (1.25-84.76) |
| Carbam+Lamot vs Carbam+Clonaz | | NA | NR | NR | NR | | NR | NR | NA | | 0.03 (0.00-0.97) (0.00-0.98) |
| Carbam+Lamot vs Carbam+Pheny | | NA | NR | NR | NR | | NR | NR | NA | | 0.04 (0.00-0.77) (0.00-0.79) |
| Carbam+Lamot vs Pheno+Valpro | | NA | NR | NR | NR | | NR | NR | NA | | 0.03 (0.00-0.88) (0.00-0.88) |
| Carbam+Lamot vs Pheny+Valpro | | NA | NR | NR | NR | | NR | NR | NA | | 0.03 (0.00-0.75) (0.00-0.78) |
| Carbam+Pheny vs Carbam | | 4 (NA) | 132 (26.33) | Epilepsy | 1st trimester | | H | H | 10.64 (2.02-61.64) | | 7.12 (2.28-20.18) (2.15-21.10) |
| Carbam+Pheny vs Control | | 2 (0.04) | 54 (28.00) | Epilepsy | NR | | H | H | 7.57 (0.84-75.20) | | 6.58 (2.25-18.97) (2.11-20.23) |
| Carbam+Pheny vs Pheno | | 4 (NA) | 103 (27.50) | Epilepsy | 1st trimester | | H | H | 2.50 (0.37-14.65) | | 4.26 (1.42-12.26) (1.34-12.97) |
| Carbam+Pheny vs Pheno+Pheny | | 3 (NA) | 27 (26.00) | Epilepsy | 1st trimester | | H | H | 11.16 (1.11-224.40) | | 5.86 (1.77-18.24) (1.67-18.93) |
| Carbam+Pheny vs Pheny | | 5 (NA) | 192 (26.60) | Epilepsy | 1st trimester | | H | H | 12.12 (2.30-49.04) | | 6.73 (2.24-18.32) (2.11-19.74) |
| Clobaz vs Lamot+Valpro | | NA | NR | NR | NR | | NR | NR | NA | | 46.28 (1.07-2.69 x 104) (1.08-2.68 x 10^4) |
| Gabap vs Carbam | | 3 (NA) | 1732 (29.20) | Epilepsy | 1st trimester | | H | L | 5.94 (1.31-20.00) | | 6.56 (1.54-20.28) (1.49-21.39) |
| Gabap vs Control | | 2 (0.01) | 45 (NR) | Epilepsy | 1st trimester | | H | H | 8.57 (0.26-162.50) | | 5.98 (1.34-19.73) (1.28-20.68) |
| Gabap vs Pheno+Pheny | | NA | NR | NR | NR | | NR | NR | NA | | 5.36 (1.17-18.53) (1.10-19.95) |
| Gabap vs Pheny | | 1 (NA) | 113 (29.20) | Epilepsy | 1st trimester | | L | L | 2.65 (0.18-58.62) | | 6.09 (1.42-19.42) (1.36-20.24) |
| Gabap vs Primid | | NA | NR | NR | NR | | NR | NR | NA | | 6.83 (1.26-29.75) (1.21-31.23) |
| Lamot vs Carbam | | 10 (NA) | 8556 (29.70) | Epilepsy | 1st trimester | | H | H | 0.72 (0.43-1.19) | | 0.59 (0.37-0.91) (0.32-1.05) |
| Lamot vs Carbam+Clonaz | | NA | NR | NR | NR | | NR | NR | NA | | 0.05 (0.01-0.43) (0.01-0.44) |
| Lamot vs Carbam+Pheny | | NA | NR | NR | NR | | NR | NR | NA | | 0.08 (0.03-0.26) (0.03-0.28) |
| Lamot vs Clonaz | | 1 (NA) | 2107 (28.90) | Epilepsy | 1st trimester | | L | L | 0.13 (0.01-61.69) | | 0.18 (0.05-0.99) (0.05-1.03) |
| Lamot vs Control | | 2 (0.02) | 155 (NR) | Epilepsy | 1st trimester | | H | H | 1.73 (0.05-21.66) | | 0.55 (0.31-0.95) (0.28-1.08) |
| Lamot vs Ethos | | 1 (NA) | 2110 (28.90) | Epilepsy | 1st trimester | | L | L | 0.27 (0.02-131.90) | | 0.16 (0.05-0.72) (0.04-0.76) |
| Lamot vs Gabap | | 2 (NA) | 2161 (28.90) | Epilepsy | 1st trimester | | H | H | 0.07 (0.02-0.38) | | 0.09 (0.03-0.39) (0.03-0.41) |
| Lamot vs Pheno | | 2 (NA) | 1851 (30.20) | Epilepsy | 1st trimester | | H | H | 0.14 (0.04-0.50) | | 0.36 (0.20-0.64) (0.18-0.71) |
| Lamot vs Pheno+Pheny+Primid | | NA | NR | NR | NR | | NR | NR | NA | | 0.10 (0.02-0.76) (0.02-0.78) |
| Lamot vs Pheno+Valpro | | NA | NR | NR | NR | | NR | NR | NA | | 0.07 (0.01-0.51) (0.01-0.52) |
| Lamot vs Pheny | | 3 (NA) | 4312 (29.37) | Epilepsy | 1st trimester | | L | L | 0.34 (0.14-0.96) | | 0.56 (0.31-0.95) (0.28-1.08) |
| Lamot vs Pheny+Valpro | | NA | NR | NR | NR | | NR | NR | NA | | 0.06 (0.02-0.22) (0.02-0.23) |
| Lamot vs Valpro | | 10 (NA) | 6950 (30.34) | Epilepsy | 1st trimester | | H | H | 0.32 (0.19-0.52) | | 0.36 (0.23-0.56) (0.20-0.65) |
| Lamot+Levet vs Pheny+Valpro | | NA | NR | NR | NR | | NR | NR | NA | | 0.04 (0.00-0.82) (0.00-0.84) |
| Lamot+Valpro vs Carbam+Clonaz | | NA | NR | NR | NR | | NR | NR | NA | | 0.01 (0.00-0.50) (0.00-0.49) |
| Lamot+Valpro vs Carbam+Clonaz+Pheny | | NA | NR | NR | NR | | NR | NR | NA | | 0.01 (0.00-0.61) (0.00-0.64) |
| Lamot+Valpro vs Carbam+Pheny | | NA | NR | NR | NR | | NR | NR | NA | | 0.02 (0.00-0.40) (0.00-0.41) |
| Lamot+Valpro vs Ethos | | NA | NR | NR | NR | | NR | NR | NA | | 0.04 (0.00-0.79) (0.00-0.82) |
| Lamot+Valpro vs Gabap | | NA | NR | NR | NR | | NR | NR | NA | | 0.02 (0.00-0.51) (0.00-0.52) |
| Lamot+Valpro vs Pheno+Pheny+Primid | | NA | NR | NR | NR | | NR | NR | NA | | 0.02 (0.00-0.78) (0.00-0.79) |
| Lamot+Valpro vs Pheno+Valpro | | NA | NR | NR | NR | | NR | NR | NA | | 0.02 (0.00-0.51) (0.00-0.51) |
| Lamot+Valpro vs Pheny+Valpro | | NA | NR | NR | NR | | NR | NR | NA | | 0.01 (0.00-0.32) (0.00-0.33) |
| Levet vs Carbam+Clonaz | | NA | NR | NR | NR | | NR | NR | NA | | 0.02 (0.00-0.26) (0.00-0.27) |
| Levet vs Carbam+Clonaz+Pheny | | NA | NR | NR | NR | | NR | NR | NA | | 0.03 (0.00-0.88) (0.00-0.90) |
| Levet vs Carbam+Pheno | | NA | NR | NR | NR | | NR | NR | NA | | 0.12 (0.01-0.78) (0.01-0.80) |
| Levet vs Carbam+Pheny | | NA | NR | NR | NR | | NR | NR | NA | | 0.04 (0.00-0.20) (0.00-0.20) |
| Levet vs Clobaz | | NA | NR | NR | NR | | NR | NR | NA | | 0.05 (0.00-0.86) (0.00-0.87) |
| Levet vs Clonaz | | NA | NR | NR | NR | | NR | NR | NA | | 0.08 (0.01-0.85) (0.01-0.88) |
| Levet vs Control | | NA | NR | NR | NR | | NR | NR | NA | | 0.25 (0.03-0.96) (0.03-1.01) |
| Levet vs Ethos | | NA | NR | NR | NR | | NR | NR | NA | | 0.07 (0.01-0.45) (0.01-0.48) |
| Levet vs Gabap | | 1 (NA) | 335 (29.20) | Epilepsy | 1st trimester | | L | L | 0.02 (0.00-0.58) | | 0.04 (0.01-0.28) (0.01-0.29) |
| Levet vs Pheno | | 1 (NA) | 649 (29.60) | Epilepsy | 1st trimester | | L | H | 0.09 (0.01-0.82) | | 0.16 (0.02-0.63) (0.02-0.66) |
| Levet vs Pheno+Pheny | | NA | NR | NR | NR | | NR | NR | NA | | 0.23 (0.03-0.92) (0.03-0.96) |
| Levet vs Pheno+Pheny+Primid | | NA | NR | NR | NR | | NR | NR | NA | | 0.04 (0.00-0.51) (0.00-0.52) |
| Levet vs Pheno+Valpro | | NA | NR | NR | NR | | NR | NR | NA | | 0.03 (0.00-0.33) (0.00-0.34) |
| Levet vs Pheny | | 2 (NA) | 1252 (29.40) | Epilepsy | 1st trimester | | L | H | 0.20 (0.03-0.99) | | 0.25 (0.04-0.96) (0.03-1.03) |
| Levet vs Pheny+Valpro | | NA | NR | NR | NR | | NR | NR | NA | | 0.03 (0.00-0.16) (0.00-0.16) |
| Levet vs Valpro | | 2 (NA) | 2297 (29.40) | Epilepsy | 1st trimester | | L | H | 0.10 (0.01-0.42) | | 0.16 (0.02-0.61) (0.02-0.63) |
| Oxcar vs Carbam+Clonaz | | NA | NR | NR | NR | | NR | NR | NA | | 0.07 (0.01-0.64) (0.01-0.65) |
| Oxcar vs Carbam+Pheny | | NA | NR | NR | NR | | NR | NR | NA | | 0.11 (0.02-0.50) (0.02-0.52) |
| Oxcar vs Gabap | | 1 (NA) | 38 (29.20) | Epilepsy | 1st trimester | | L | L | 0.87 (0.01-31.96) | | 0.12 (0.02-0.65) (0.02-0.68) |
| Oxcar vs Pheno+Valpro | | NA | NR | NR | NR | | NR | NR | NA | | 0.08 (0.01-0.92) (0.01-0.94) |
| Oxcar vs Pheny+Valpro | | NA | NR | NR | NR | | NR | NR | NA | | 0.08 (0.01-0.35) (0.01-0.36) |
| Pheno+Valpro vs Carbam | | 2 (NA) | 84 (27.00) | Epilepsy | 1st trimester | | H | H | 8.26 (0.57-149.30) | | 8.53 (1.23-38.29) (1.19-39.76) |
| Pheno+Valpro vs Control | | 1 (0.09) | 36 (NR) | Epilepsy | NR | | H | H | 1.20 (0.00-40.55) | | 8.01 (1.17-35.40) (1.13-37.53) |
| Pheno+Valpro vs Pheny | | NA | NR | NR | NR | | NR | NR | NA | | 8.02 (1.14-37.71) (1.08-39.16) |
| Pheno+Valpro vs Primid | | NA | NR | NR | NR | | NR | NR | NA | | 9.01 (1.07-55.18) (1.03-56.63) |
| Pheny+Valpro vs Carbam | | 2 (NA) | 129 (NR) | Epilepsy | 1st trimester | | H | H | 97.04  (12.27-2.16 x 10^3^) | | 9.62 (2.70-30.72) (2.63-32.70) |
| Pheny+Valpro vs Control | | 1 (0.09) | 36 (NR) | Epilepsy | NR | | H | H | 10.78 (0.60-189.50) | | 8.88 (2.62-30.65) (2.49-32.50) |
| Pheny+Valpro vs Pheno | | 1 (NA) | 6 (NR) | Epilepsy | NR | | H | H | 23.79  (0.41-9.48 x 10^3^) | | 5.71 (1.61-18.68) (1.50-19.72) |
| Pheny+Valpro vs Pheno+Pheny | | 2 (NA) | 43 (26.00) | Epilepsy | 1st trimester | | H | H | 4.16 (0.31-42.32) | | 7.88 (2.13-26.66) (2.03-28.40) |
| Pheny+Valpro vs Pheny | | 4 (NA) | 156 (26.90) | Epilepsy | 1st trimester | | H | H | 9.55 (2.12-36.45) | | 8.97 (2.55-28.56) (2.45-30.51) |
| Pheny+Valpro vs Primid | | 1 (NA) | 23 (NR) | Epilepsy | NR | | H | H | 102.50  (1.77-9.22 x 10^4^) | | 9.84 (2.35-50.97) (2.22-54.46) |
| Pheny+Valpro vs Valpro | | 2 (NA) | 32 (NR) | Epilepsy | 1st trimester | | H | H | 15.13 (1.70-446.00) | | 5.76 (1.70-18.53) (1.64-19.55) |
| Primid vs Carbam+Pheny | | 1 (NA) | 23 (NR) | Epilepsy | NR | | H | H | 0.01 (0.00-0.38) | | 0.14 (0.03-0.48) (0.03-0.51) |
| Topir vs Carbam+Clonaz | | NA | NR | NR | NR | | NR | NR | NA | | 0.06 (0.01-0.64) (0.01-0.66) |
| Topir vs Carbam+Pheny | | NA | NR | NR | NR | | NR | NR | NA | | 0.10 (0.02-0.48) (0.02-0.51) |
| Topir vs Gabap | | 1 (NA) | 101 (29.20) | Epilepsy | 1st trimester | | L | L | 0.07 (0.00-1.49) | | 0.11 (0.02-0.65) (0.02-0.69) |
| Topir vs Pheno+Valpro | | NA | NR | NR | NR | | NR | NR | NA | | 0.08 (0.01-0.77) (0.01-0.78) |
| Topir vs Pheny+Valpro | | NA | NR | NR | NR | | NR | NR | NA | | 0.08 (0.01-0.35) (0.01-0.37) |
| Valpro vs Carbam | | 25 (NA) | 8338 (27.75) | Epilepsy | 1st trimester | | H | H | 1.70 (1.17-2.36) | | 1.65 (1.18-2.28) (0.98-2.76) |
| Valpro vs Carbam+Pheny | | 2 (NA) | 29 (28.00) | Epilepsy | NR | | H | H | 0.16 (0.01-1.51) | | 0.23 (0.08-0.70) (0.08-0.74) |
| Valpro vs Pheny | | 18 (NA) | 3210 (27.33) | Epilepsy | 1st trimester | | H | L | 1.78 (1.01-3.18) | | 1.55 (1.01-2.46) (0.88-2.79) |
| Common between-study variance across treatment comparisons | | | | | | | | | 0.02 (0.00-0.13) | | 0.02 (0.00-0.16) (NA) |
| Residual deviance:219  Data points: 291  DIC: 322 | |  | | | | | | | | | |
| Evaluation of consistency using the design-by-treatment interaction model | | Chi-square test: 100.89  Degrees of Freedom: 172  P- value: 1  Heterogeneity: 0.000 | | | | | | | | | |
| **Hypospadias (31 studies, 12365 cases, 32 treatments)** | | | | | | | | | | | |
| Clonaz vs Carbam | | 4 (NA) | 924 (27.80) | Epilepsy | 1st trimester | | H | H | 6.37 (0.71-36.22) | | 5.56 (1.13-18.78) (0.97-21.42) |
| Clonaz vs Control | | 2 (0.02) | 72 (27.80) | Epilepsy | 1st trimester | | H | H | 2.64 (0.08-57.62) | | 6.17 (1.17-24.80) (1.04-27.47) |
| Clonaz vs Pheny | | 5 (NA) | 441 (28.50) | Epilepsy | 1st trimester | | H | L | 2.37 (0.38-11.88) | | 5.48 (1.07-19.62) (0.94-22.91) |
| Ethos vs Pheny | | 6 (NA) | 474 (28.10) | Epilepsy | 1st trimester | | H | H | 5.76 (1.11-23.94) | | 6.21 (1.13-22.86) (1.02-25.57) |
| Gabap vs Carbam | | 1 (NA) | 28 (NR) | Epilepsy | 1st trimester | | H | L | 270.80  (6.99-7.86 x 10^5^) | | 15.24 (2.28-100.80)  (2.05-114.90) |
| Gabap vs Control | | 1 (NA) | 39 (NR) | Epilepsy | 1st trimester | | H | L | 4,096.00  (36.18-1.93 x 10^7^) | | 16.54 (2.50-121.70)  (2.27-135.20) |
| Gabap vs Pheno | | NA | NR | NR | NR | | NR | NR | NA | | 11.18 (1.64-82.68) (1.49-92.83) |
| Gabap vs Pheno+Pheny | | NA | NR | NR | NR | | NR | NR | NA | | 17.39 (1.72-176.70)  (1.59-197.50) |
| Gabap vs Pheny | | 2 (NA) | 117 (29.20) | Epilepsy | 1st trimester | | H | L | 14.67 (0.68-854.40) | | 14.86 (2.25-103.80)  (2.08-119.00) |
| Lamot vs Carbam+Clonaz | | NA | NR | NR | NR | | NR | NR | NA | | 0.11 (0.02-0.90) (0.02-1.00) |
| Lamot vs Carbam+Pheno | | NA | NR | NR | NR | | NR | NR | NA | | 0.14 (0.03-0.81) (0.03-0.92) |
| Lamot vs Carbam+Valpro | | 1 (NA) | 17 (29.40) | Epilepsy | whole PG | | H | L | 0.41 (0.00-9.72) | | 0.15 (0.03-0.88) (0.03-1.00) |
| Lamot vs Clonaz | | NA | NR | NR | NR | | NR | NR | NA | | 0.11 (0.02-0.69) (0.02-0.77) |
| Lamot vs Ethos | | NA | NR | NR | NR | | NR | NR | NA | | 0.10 (0.02-0.66) (0.02-0.68) |
| Lamot vs Gabap | | 1 (NA) | 10 (NR) | Epilepsy | 1st trimester | | H | L | 0.00 (0.00-0.18) | | 0.04 (0.01-0.29) (0.00-0.33) |
| Lamot vs Primid | | 1 (NA) | 11 (NR) | Epilepsy | NR | | H | H | 0.16 (0.00-53.70) | | 0.12 (0.03-0.66) (0.02-0.69) |
| Lamot vs Valpro | | 8 (NA) | 3534 (29.90) | Epilepsy | 1st trimester | | H | H | 0.19 (0.07-0.46) | | 0.26 (0.10-0.63) (0.08-0.75) |
| Levet vs Carbam+Clonaz | | NA | NR | NR | NR | | NR | NR | NA | | 0.05 (0.00-0.72) (0.00-0.75) |
| Levet vs Carbam+Pheno | | NA | NR | NR | NR | | NR | NR | NA | | 0.06 (0.00-0.78) (0.00-0.84) |
| Levet vs Clonaz | | 1 (NA) | 313 (29.20) | Epilepsy | 1st trimester | | L | L | 0.02 (0.00-12.81) | | 0.05 (0.00-0.61) (0.00-0.66) |
| Levet vs Ethos | | 1 (NA) | 316 (29.20) | Epilepsy | 1st trimester | | L | L | 0.05 (0.00-45.06) | | 0.04 (0.00-0.62) (0.00-0.69) |
| Levet vs Gabap | | 1 (NA) | 335 (29.20) | Epilepsy | 1st trimester | | L | L | 0.07 (0.00-20.79) | | 0.02 (0.00-0.20) (0.00-0.22) |
| Levet vs Oxcar | | 2 (NA) | 943 (29.40) | Epilepsy | 1st trimester | | L | H | 0.07 (0.00-1.62) | | 0.06 (0.00-0.70) (0.00-0.77) |
| Levet vs Primid | | NA | NR | NR | NR | | NR | NR | NA | | 0.05 (0.00-0.68) (0.00-0.76) |
| Levet vs Topir | | 2 (NA) | 1183 (29.40) | Epilepsy | 1st trimester | | L | H | 0.07 (0.00-0.66) | | 0.09 (0.00-0.82) (0.00-0.90) |
| Levet vs Valpro | | 1 (NA) | 773 (29.60) | Epilepsy | 1st trimester | | L | H | 0.02 (0.00-0.34) | | 0.11 (0.00-0.96) (0.00-1.02) |
| Oxcar vs Lamot | | 1 (NA) | 1744 (30.40) | Epilepsy | 1st trimester | | L | H | 53.48  (1.86-1.03 x 10^4^) | | 7.65 (1.48-33.40) (1.30-37.16) |
| Primid vs Carbam | | 4 (NA) | 1015 (27.40) | Epilepsy | 1st trimester | | H | H | 8.86 (1.47-35.60) | | 5.17 (1.05-18.05) (0.98-20.61) |
| Primid vs Control | | 3 (0.00) | 218 (27.40) | Epilepsy | 1st trimester | | H | H | 7.83 (0.48-93.05) | | 5.92 (1.00-23.77) (0.93-27.21) |
| Primid vs Pheny | | 5 (NA) | 439 (27.40) | Epilepsy | 1st trimester | | H | H | 3.73 (0.70-14.97) | | 5.07 (1.02-19.52) (0.94-21.50) |
| Topir vs Lamot | | 1 (NA) | 1921 (28.80) | Epilepsy | 1st trimester | | L | H | 41.49  (2.00-3.06 x 10^4^) | | 5.27 (1.10-23.48) (0.99-26.64) |
| Valpro vs Carbam | | 24 (NA) | 4473 (27.60) | Epilepsy | 1st trimester | | H | L | 2.39 (1.43-4.24) | | 2.33 (1.39-3.89) (1.01-5.08) |
| Valpro vs Control | | 10 (0.02) | 803 (27.68) | Epilepsy | 1st trimester | | H | L | 2.22 (1.02-4.92) | | 2.58 (1.24-5.76) (0.99-7.06) |
| Valpro vs Pheny | | 19 (NA) | 1904 (27.15) | Epilepsy | 1st trimester | | H | L | 2.31 (1.10-4.60) | | 2.32 (1.24-4.19) (0.94-5.48) |
| Common between-study variance across treatment comparisons | | | | | | | | | 0.02 (0.00-0.16) | | 0.04 (0.00-0.41) (NA) |
| Residual deviance:138  Data points: 183  DIC: 212 | |  | | | | | | | | | |
| Evaluation of consistency using the design-by-treatment interaction model | | Chi-square test: 56.34  Degrees of Freedom: 102  P- value: 0.99  Heterogeneity: 0.000 | | | | | | | | | |
| **Cleft Lip/Palate (29 studies, 18987 cases, 33 treatments)** | | | | | | | | | | | |
| Carbam vs Ethos | | 5 (NA) | 2264 (28.70) | Epilepsy | 1st trimester | | H | H | 0.07 (0.02-0.33) | | 0.06 (0.02-0.27) (0.02-0.30) |
| Carbam vs Pheno | | 10 (NA) | 2292 (28.00) | Epilepsy | 1st trimester | | H | H | 0.24 (0.10-0.58) | | 0.24 (0.11-0.49) (0.10-0.57) |
| Carbam vs Pheny | | 17 (NA) | 5717 (28.19) | Epilepsy | 1st trimester | | H | L | 0.59 (0.29-1.16) | | 0.44 (0.23-0.86) (0.18-1.02) |
| Carbam+Pheno vs Carbam | | 3 (NA) | 235 (27.00) | Epilepsy | 1st trimester | | H | H | 11.59 (1.90-73.64) | | 13.72 (2.72-61.54) (2.54-69.23) |
| Carbam+Pheno vs Control | | 1 (NA) | 44 (27.00) | Epilepsy | 1st trimester | | H | H | 8.79  (0.03-1.27 x 10^3^) | | 18.51 (3.34-94.21)  (3.09-103.10) |
| Carbam+Pheno vs Pheno+Pheny | | 1 (NA) | 24 (27.00) | Epilepsy | 1st trimester | | H | H | 2.53 (0.01-472.50) | | 9.38 (1.63-59.58) (1.49-65.40) |
| Carbam+Pheno vs Pheny | | 2 (NA) | 64 (27.00) | Epilepsy | 1st trimester | | H | H | 5.01 (0.15-153.70) | | 5.97 (1.04-27.70) (0.97-30.03) |
| Carbam+Valpro vs Carbam | | 3 (NA) | 267 (27.00) | Epilepsy | 1st trimester | | H | H | 9.94 (1.87-55.92) | | 14.04 (3.15-55.90) (2.89-62.41) |
| Carbam+Valpro vs Control | | 1 (NA) | 45 (27.00) | Epilepsy | 1st trimester | | H | H | 25.01  (1.12-4.39 x 10^3^) | | 19.12 (3.74-88.68) (3.50-96.58) |
| Carbam+Valpro vs Pheno+Pheny | | 1 (NA) | 25 (27.00) | Epilepsy | 1st trimester | | H | H | 18.24  (0.62-3.15 x 10^3^) | | 9.46 (1.51-54.43) (1.42-58.31) |
| Carbam+Valpro vs Pheny | | 2 (NA) | 74 (27.00) | Epilepsy | 1st trimester | | H | H | 11.03 (1.24-195.80) | | 6.18 (1.26-24.74) (1.17-27.27) |
| Carbam+Valpro vs Valpro | | 3 (NA) | 200 (27.00) | Epilepsy | 1st trimester | | H | H | 10.79 (1.93-82.88) | | 5.92 (1.31-21.95) (1.22-24.84) |
| Ethos vs Control | | 2 (NA) | 66 (27.00) | Epilepsy | 1st trimester | | H | H | 36.15 (2.10-907.10) | | 22.22 (4.56-87.64) (4.16-96.63) |
| Ethos vs Pheno+Pheny | | 1 (NA) | 22 (27.00) | Epilepsy | 1st trimester | | H | H | 3.51  (0.01-1.74 x 10^3^) | | 11.19 (1.95-52.22) (1.76-57.07) |
| Ethos vs Pheny | | 5 (NA) | 348 (28.10) | Epilepsy | 1st trimester | | H | H | 6.56 (1.29-29.27) | | 7.15 (1.54-23.72) (1.42-26.20) |
| Lamot vs Carbam+Pheno | | NA | NR | NR | NR | | NR | NR | NA | | 0.06 (0.01-0.34) (0.01-0.36) |
| Lamot vs Carbam+Valpro | | NA | NR | NR | NR | | NR | NR | NA | | 0.06 (0.02-0.30) (0.01-0.33) |
| Lamot vs Ethos | | 1 (NA) | 2110 (28.90) | Epilepsy | 1st trimester | | L | L | 0.07 (0.00-46.29) | | 0.05 (0.02-0.25) (0.01-0.28) |
| Lamot vs Pheno | | 3 (NA) | 1839 (30.60) | Epilepsy | 1st trimester | | H | H | 0.18 (0.06-0.55) | | 0.21 (0.09-0.50) (0.08-0.59) |
| Lamot vs Pheno+Pheny+Primid | | NA | NR | NR | NR | | NR | NR | NA | | 0.11 (0.02-0.66) (0.02-0.72) |
| Lamot vs Pheny | | 5 (NA) | 4357 (29.37) | Epilepsy | 1st trimester | | L | L | 0.53 (0.17-1.76) | | 0.39 (0.18-0.84) (0.15-1.01) |
| Lamot vs Pheny+Primid | | NA | NR | NR | NR | | NR | NR | NA | | 0.07 (0.02-0.38) (0.01-0.41) |
| Lamot vs Primid | | 1 (NA) | 11 (NR) | Epilepsy | NR | | H | H | 0.19 (0.00-48.19) | | 0.16 (0.04-0.78) (0.04-0.88) |
| Lamot vs Valpro | | 7 (NA) | 6764 (29.78) | Epilepsy | 1st trimester | | L | L | 0.35 (0.19-0.62) | | 0.37 (0.20-0.67) (0.16-0.86) |
| Levet vs Carbam+Pheno | | NA | NR | NR | NR | | NR | NR | NA | | 0.02 (0.00-0.20) (0.00-0.21) |
| Levet vs Carbam+Valpro | | NA | NR | NR | NR | | NR | NR | NA | | 0.02 (0.00-0.18) (0.00-0.19) |
| Levet vs Clobaz | | NA | NR | NR | NR | | NR | NR | NA | | 0.02 (0.00-0.90) (0.00-0.94) |
| Levet vs Ethos | | 1 (NA) | 316 (29.20) | Epilepsy | 1st trimester | | L | L | 0.03 (0.00-7.38) | | 0.02 (0.00-0.14) (0.00-0.15) |
| Levet vs Oxcar | | 2 (NA) | 943 (29.40) | Epilepsy | 1st trimester | | L | H | 0.05 (0.00-1.13) | | 0.15 (0.02-0.88) (0.02-0.94) |
| Levet vs Pheno | | 1 (NA) | 649 (29.60) | Epilepsy | 1st trimester | | L | H | 0.03 (0.00-0.34) | | 0.08 (0.01-0.33) (0.01-0.37) |
| Levet vs Pheno+Pheny+Primid | | NA | NR | NR | NR | | NR | NR | NA | | 0.04 (0.00-0.39) (0.00-0.42) |
| Levet vs Pheno+Primid | | NA | NR | NR | NR | | NR | NR | NA | | 0.03 (0.00-0.67) (0.00-0.69) |
| Levet vs Pheny | | 2 (NA) | 1252 (29.40) | Epilepsy | 1st trimester | | L | H | 0.10 (0.01-0.83) | | 0.15 (0.03-0.60) (0.02-0.67) |
| Levet vs Pheny+Primid | | NA | NR | NR | NR | | NR | NR | NA | | 0.03 (0.00-0.24) (0.00-0.26) |
| Levet vs Primid | | NA | NR | NR | NR | | NR | NR | NA | | 0.06 (0.01-0.44) (0.01-0.46) |
| Levet vs Topir | | 2 (NA) | 1183 (29.40) | Epilepsy | 1st trimester | | L | H | 0.04 (0.00-0.27) | | 0.08 (0.01-0.35) (0.01-0.39) |
| Levet vs Valpro | | 3 (NA) | 2748 (29.40) | Epilepsy | 1st trimester | | L | L | 0.13 (0.02-0.51) | | 0.15 (0.02-0.55) (0.02-0.61) |
| Oxcar vs Ethos | | 2 (NA) | 30 (29.20) | Epilepsy | 1st trimester | | H | H | 3.85 (0.11-343.50) | | 0.15 (0.03-0.83) (0.02-0.90) |
| Pheno vs Control | | 6 (NA) | 1042 (27.00) | Epilepsy | 1st trimester | | H | H | 5.69 (1.56-45.08) | | 5.75 (2.41-14.08) (2.09-16.34) |
| Pheno+Pheny vs Pheno | | 8 (NA) | 644 (26.50) | Epilepsy | 1st trimester | | H | L | 0.45 (0.14-1.51) | | 0.36 (0.11-0.91) (0.10-1.07) |
| Pheno+Pheny+Primid vs Carbam | | 1 (NA) | 19 (26.00) | Epilepsy | 1st trimester | | H | L | 12.72  (0.55-2.52 x 10^3^) | | 8.35 (1.34-43.22) (1.26-46.72) |
| Pheno+Pheny+Primid vs Control | | NA | NR | NR | NR | | NR | NR | NA | | 11.50 (1.70-63.48) (1.63-69.10) |
| Pheno+Pheny+Primid vs Pheno+Pheny | | 2 (NA) | 105 (26.00) | Epilepsy | 1st trimester | | H | H | 5.04 (0.70-46.73) | | 5.53 (1.02-32.14) (0.95-35.89) |
| Pheny vs Control | | 10 (0.00) | 1933 (27.45) | Epilepsy | 1st trimester | | H | L | 4.88 (1.55-18.46) | | 3.11 (1.31-7.72) (1.15-8.86) |
| Pheny+Primid vs Carbam | | 1 (NA) | 15 (26.00) | Epilepsy | 1st trimester | | H | L | 1.69  (0.00-1.17 x 10^3^) | | 12.36 (2.48-50.60) (2.26-56.07) |
| Pheny+Primid vs Control | | 1 (NA) | 113 (NR) | Epilepsy | NR | | H | L | 74.70  (0.15-1.28 x 10^5^) | | 16.75 (3.02-77.19) (2.81-87.38) |
| Pheny+Primid vs Pheno+Pheny | | 4 (NA) | 170 (26.00) | Epilepsy | 1st trimester | | H | L | 8.10 (1.70-60.97) | | 8.17 (1.76-43.85) (1.59-48.70) |
| Pheny+Primid vs Pheny | | 3 (NA) | 58 (26.00) | Epilepsy | 1st trimester | | H | L | 3.46 (0.23-45.27) | | 5.29 (1.07-21.71) (0.98-23.75) |
| Primid vs Carbam | | 4 (NA) | 590 (27.00) | Epilepsy | 1st trimester | | H | H | 5.94 (0.69-36.29) | | 5.56 (1.20-19.27) (1.08-21.57) |
| Primid vs Control | | 4 (NA) | 290 (27.00) | Epilepsy | 1st trimester | | H | H | 14.81 (1.59-166.20) | | 7.68 (1.41-29.27) (1.32-32.79) |
| Topir vs Carbam | | 2 (NA) | 3119 (29.00) | Epilepsy | 1st trimester | | L | H | 4.64 (1.55-14.31) | | 4.48 (1.71-11.07) (1.50-13.34) |
| Topir vs Control | | NA | NR | NR | NR | | NR | NR | NA | | 6.12 (1.89-19.05) (1.70-22.22) |
| Topir vs Lamot | | 2 (NA) | 4089 (29.00) | Epilepsy | 1st trimester | | L | H | 5.86 (1.94-16.86) | | 5.07 (1.90-13.14) (1.67-15.71) |
| Valpro vs Carbam | | 17 (NA) | 7720 (27.74) | Epilepsy | 1st trimester | | H | L | 2.58 (1.56-4.18) | | 2.39 (1.45-3.86) (1.10-4.99) |
| Valpro vs Carbam+Pheno | | 2 (NA) | 118 (27.00) | Epilepsy | 1st trimester | | H | H | 0.14 (0.00-4.48) | | 0.17 (0.04-0.91) (0.03-0.98) |
| Valpro vs Control | | 7 (0.00) | 1827 (27.45) | Epilepsy | 1st trimester | | H | L | 3.02 (1.04-9.48) | | 3.26 (1.38-7.58) (1.16-9.08) |
| Valpro vs Ethos | | 4 (NA) | 1359 (27.65) | Epilepsy | 1st trimester | | H | H | 0.11 (0.03-0.55) | | 0.15 (0.04-0.66) (0.04-0.71) |
| Valpro vs Pheny+Primid | | 1 (NA) | 8 (26.00) | Epilepsy | 1st trimester | | H | L | 2.38  (0.01-2.43 x 10^3^) | | 0.19 (0.05-0.92) (0.04-0.99) |
| Common between-study variance across treatment comparisons | | | | | | | | | 0.02 (0.00-0.18) | | 0.04 (0.00-0.34) (NA) |
| Residual deviance:123  Data points: 164  DIC: 195 | |  | | | | | | | | | |
| Evaluation of consistency using the design-by-treatment interaction model | | Chi-square test: 52.58  Degrees of Freedom: 100  P- value: 1  Heterogeneity: 0.000 | | | | | | | | | |
| **Club Foot (23 studies, 8836 cases, 27 treatments)** | | | | | | | | | | | |
| Carbam+Pheno vs Control | | 2 (NA) | 78 (27.00) | Epilepsy | 1st trimester | | H | H | 7.93 (0.61-443.90) | | 7.30 (1.29-32.31) (1.12-36.76) |
| Carbam+Pheno vs Pheno+Pheny | | 1 (NA) | 24 (27.00) | Epilepsy | 1st trimester | | H | H | 3.31  (0.01-3.50 x 10^3^) | | 7.99 (1.17-49.28) (1.04-56.46) |
| Ethos vs Control | | 2 (NA) | 66 (27.00) | Epilepsy | 1st trimester | | H | H | 9.59 (0.19-422.20) | | 12.99 (1.66-76.39) (1.49-86.21) |
| Ethos vs Pheno | | 3 (NA) | 92 (27.00) | Epilepsy | 1st trimester | | H | H | 8.10 (0.42-187.10) | | 9.62 (1.18-53.98) (1.02-62.24) |
| Ethos vs Pheno+Pheny | | 1 (NA) | 22 (27.00) | Epilepsy | 1st trimester | | H | H | 4.75  (0.01-4.34 x 10^3^) | | 14.09 (1.46-105.80) (1.34-122.20) |
| Lamot vs Carbam+Pheno | | NA | NR | NR | NR | | NR | NR | NA | | 0.10 (0.01-0.84) (0.01-0.94) |
| Lamot vs Carbam+Valpro | | NA | NR | NR | NR | | NR | NR | NA | | 0.07 (0.01-0.86) (0.01-0.95) |
| Lamot vs Ethos | | NA | NR | NR | NR | | NR | NR | NA | | 0.05 (0.01-0.55) (0.00-0.62) |
| Lamot vs Pheno+Pheny+Primid | | NA | NR | NR | NR | | NR | NR | NA | | 0.05 (0.00-0.59) (0.00-0.64) |
| Lamot vs Primid | | 1 (NA) | 11 (NR) | Epilepsy | NR | | H | H | 0.26 (0.00-137.90) | | 0.14 (0.02-0.88) (0.02-1.00) |
| Lamot vs Valpro | | 4 (NA) | 2061 (31.99) | Epilepsy | 1st trimester | | H | H | 0.14 (0.02-0.60) | | 0.22 (0.04-0.78) (0.04-0.96) |
| Levet vs Carbam+Pheno | | NA | NR | NR | NR | | NR | NR | NA | | 0.03 (0.00-0.85) (0.00-0.94) |
| Levet vs Carbam+Valpro | | NA | NR | NR | NR | | NR | NR | NA | | 0.03 (0.00-0.86) (0.00-0.97) |
| Levet vs Ethos | | NA | NR | NR | NR | | NR | NR | NA | | 0.02 (0.00-0.58) (0.00-0.63) |
| Levet vs Pheno+Pheny+Primid | | NA | NR | NR | NR | | NR | NR | NA | | 0.02 (0.00-0.54) (0.00-0.58) |
| Pheno+Pheny+Primid vs Control | | NA | NR | NR | NR | | NR | NR | NA | | 13.46 (1.45-132.80) (1.38-142.30) |
| Pheno+Pheny+Primid vs Pheno | | 1 (NA) | 16 (26.00) | Epilepsy | 1st trimester | | H | L | 8.55  (0.34-2.42 x 10^3^) | | 10.36 (1.13-88.24) (0.99-90.78) |
| Pheno+Pheny+Primid vs Pheno+Pheny | | 1 (NA) | 36 (26.00) | Epilepsy | 1st trimester | | H | L | 30.58  (1.66-8.48 x 10^3^) | | 15.49 (1.64-138.50) (1.49-152.70) |
| Pheny vs Control | | 12 (0.02) | 955 (27.05) | Epilepsy | 1st trimester | | H | L | 2.72 (0.99-8.14) | | 2.73 (1.13-6.18) (0.86-8.05) |
| Primid vs Control | | 5 (0.00) | 365 (27.40) | Epilepsy | 1st trimester | | H | L | 6.90 (0.90-49.71) | | 4.71 (1.11-17.24) (0.99-21.28) |
| Valpro vs Carbam | | 14 (NA) | 2755 (27.30) | Epilepsy | 1st trimester | | H | H | 2.20 (1.13-4.86) | | 2.00 (1.00-4.11) (0.79-5.41) |
| Valpro vs Control | | 9 (0.02) | 768 (27.05) | Epilepsy | 1st trimester | | H | H | 1.83 (0.66-5.83) | | 3.26 (1.43-8.25) (1.10-9.75) |
| Valpro vs Pheno | | 11 (NA) | 1468 (27.47) | Epilepsy | 1st trimester | | H | H | 2.34 (0.83-6.85) | | 2.38 (1.05-6.18) (0.80-7.30) |
| Common between-study variance across treatment comparisons | | | | | | | | | 0.03 (0.00-0.27) | | 0.06 (0.00-0.54) (NA) |
| Residual deviance:102  Data points: 132  DIC: 160 | |  | | | | | | | | | |
| Evaluation of consistency using the design-by-treatment interaction model | | Chi-square test: 41.28  Degrees of Freedom: 72  P- value: 0.99  Heterogeneity: 0.000 | | | | | | | | | |
| **Inguinal Hernia (13 studies, 12216 cases, 29 treatments)** | | | | | | | | | | | |
| Carbam vs Pheno+Pheny | | 3 (NA) | 159 (26.93) | Epilepsy | 1st trimester | | H | L | 0.41 (0.10-1.81) | | 0.28 (0.09-0.94) (0.07-1.33) |
| Carbam+Clonaz vs Pheno+Primid | | NA | NR | NR | NR | | NR | NR | NA | | 0.01 (0.00-0.67) (0.00-0.74) |
| Carbam+Pheno+Valpro vs Pheno+Primid | | NA | NR | NR | NR | | NR | NR | NA | | 0.01 (0.00-0.90) (0.00-0.97) |
| Carbam+Primid vs Pheno+Primid | | NA | NR | NR | NR | | NR | NR | NA | | 0.00 (0.00-0.82) (0.00-0.89) |
| Carbam+Valpro vs Pheno+Primid | | NA | NR | NR | NR | | NR | NR | NA | | 0.01 (0.00-0.25) (0.00-0.28) |
| Lamot vs Carbam+Pheny | | NA | NR | NR | NR | | NR | NR | NA | | 0.11 (0.02-0.84) (0.01-1.02) |
| Lamot vs Pheno+Pheny | | NA | NR | NR | NR | | NR | NR | NA | | 0.16 (0.03-0.69) (0.03-0.89) |
| Lamot vs Pheno+Primid | | NA | NR | NR | NR | | NR | NR | NA | | 0.00 (0.00-0.05) (0.00-0.06) |
| Lamot vs Pheny+Valpro | | NA | NR | NR | NR | | NR | NR | NA | | 0.07 (0.01-0.87) (0.01-1.04) |
| Levet vs Pheno+Primid | | NA | NR | NR | NR | | NR | NR | NA | | 0.00 (0.00-0.12) (0.00-0.14) |
| Oxcar vs Pheno+Primid | | NA | NR | NR | NR | | NR | NR | NA | | 0.00 (0.00-0.11) (0.00-0.12) |
| Pheno+Pheny vs Control | | 3 (NA) | 159 (27.40) | Epilepsy | 1st trimester | | H | L | 7.13 (1.17-84.84) | | 5.51 (1.25-34.61) (0.94-43.72) |
| Pheno+Pheny vs Pheno | | 3 (NA) | 204 (26.50) | Epilepsy | 1st trimester | | H | L | 6.27 (1.30-47.65) | | 4.50 (1.25-20.04) (0.94-24.57) |
| Pheno+Pheny vs Pheny | | 4 (NA) | 246 (26.93) | Epilepsy | 1st trimester | | H | L | 4.06 (1.07-20.79) | | 3.52 (1.07-12.90) (0.80-18.25) |
| Pheno+Pheny+Primid vs Pheno+Primid | | 1 (NA) | 11 (26.00) | Epilepsy | 1st trimester | | H | L | 0.01 (0.00-0.54) | | 0.01 (0.00-0.49) (0.00-0.57) |
| Pheno+Primid vs Carbam | | 1 (NA) | 12 (26.00) | Epilepsy | 1st trimester | | H | L | 555.10  (5.71-2.07 x 10^6^) | | 321.60 (12.25-7.57 x 104) (10.48-7.32 x 10^4) |
| Pheno+Primid vs Carbam+Pheno | | NA | NR | NR | NR | | NR | NR | NA | | 156.90 (3.41-5.58 x 104) (3.10-5.89 x 10^4) |
| Pheno+Primid vs Carbam+Pheno+Pheny | | 1 (NA) | 4 (26.00) | Epilepsy | 1st trimester | | H | L | 184.30  (1.71-6.42 x 10^5^) | | 88.34 (1.49-2.99 x 104) (1.34-3.10 x 10^4) |
| Pheno+Primid vs Carbam+Pheny | | NA | NR | NR | NR | | NR | NR | NA | | 67.64 (1.85-2.10 x 104) (1.65-2.22 x 10^4) |
| Pheno+Primid vs Control | | NA | NR | NR | NR | | NR | NR | NA | | 534.20 (14.39-1.31 x 105) (12.95-1.43 x 10^5) |
| Pheno+Primid vs Ethos | | NA | NR | NR | NR | | NR | NR | NA | | 53.36 (1.11-1.50 x 104) (1.05-1.62 x 10^4) |
| Pheno+Primid vs Ethos+Pheny | | 1 (NA) | 6 (26.00) | Epilepsy | 1st trimester | | H | L | 216.70  (2.52-6.84 x 10^4^) | | 111.10 (2.08-3.74 x 104) (1.95-3.76 x 10^4) |
| Pheno+Primid vs Pheno | | 1 (NA) | 9 (26.00) | Epilepsy | 1st trimester | | H | L | 736.90  (6.01-1.04 x 10^6^) | | 411.90 (15.48-9.67 x 104) (13.90-1.06 x 10^5) |
| Pheno+Primid vs Pheno+Pheny | | 1 (NA) | 29 (26.00) | Epilepsy | 1st trimester | | H | L | 119.10  (4.93-4.70 x 10^4^) | | 92.56 (3.34-1.85 x 104) (2.96-2.10 x 10^4) |
| Pheno+Primid vs Pheny | | 1 (NA) | 26 (26.00) | Epilepsy | 1st trimester | | H | L | 1,105.00  (20.50-3.18 x 10^6^) | | 315.80 (11.77-6.98 x 104) (10.36-7.46 x 10^4) |
| Pheno+Primid vs Pheny+Primid | | 1 (NA) | 7 (26.00) | Epilepsy | 1st trimester | | H | L | 429.40  (4.34-2.51 x 10^6^) | | 123.30 (2.38-6.18 x 104) (2.16-6.49 x 10^4) |
| Pheno+Primid vs Primid | | NA | NR | NR | NR | | NR | NR | NA | | 157.90 (3.59-4.36 x 104) (3.21-4.30 x 10^4) |
| Pheno+Valpro vs Pheno+Primid | | NA | NR | NR | NR | | NR | NR | NA | | 0.00 (0.00-0.59) (0.00-0.67) |
| Pheny+Valpro vs Pheno+Primid | | 1 (NA) | 6 (26.00) | Epilepsy | 1st trimester | | H | L | 0.04 (0.00-1.79) | | 0.02 (0.00-0.87) (0.00-0.97) |
| Topir vs Pheno+Primid | | NA | NR | NR | NR | | NR | NR | NA | | 0.00 (0.00-0.10) (0.00-0.11) |
| Valpro vs Pheno+Primid | | 1 (NA) | 5 (26.00) | Epilepsy | 1st trimester | | H | L | 0.01 (0.00-0.40) | | 0.00 (0.00-0.09) (0.00-0.11) |
| Common between-study variance across treatment comparisons | | | | | | | | | 0.05 (0.00-0.46) | | 0.11 (0.00-0.89) (NA) |
| Residual deviance:78  Data points: 91  DIC: 129 | |  | | | | | | | | | |
| Evaluation of consistency using the design-by-treatment interaction model | | Chi-square test: 32.31  Degrees of Freedom: 50  P- value: 0.97  Heterogeneity: 0.000 | | | | | | | | | |
| **Undescended Testes (10 studies, 6270 cases, 17 treatments)** | | | | | | | | | | | |
| Lamot vs Primid | | NA | NR | NR | NR | | NR | NR | NA | | 0.10 (0.01-0.73) (0.01-0.87) |
| Levet vs Lamot | | 1 (NA) | 2012 (29.60) | Epilepsy | 1st trimester | | L | H | 6.67 (1.34-53.19) | | 6.83 (1.25-42.30) (0.98-51.48) |
| Primid vs Carbam | | 3 (NA) | 280 (27.40) | Epilepsy | 1st trimester | | H | L | 11.17 (1.26-175.30) | | 5.97 (1.20-31.29) (0.94-37.68) |
| Topir vs Primid | | NA | NR | NR | NR | | NR | NR | NA | | 0.05 (0.00-0.97) (0.00-1.09) |
| Common between-study variance across treatment comparisons | | | | | | | | | 0.04 (0.00-0.42) | | 0.08 (0.00-0.91) (NA) |
| Residual deviance:46  Data points: 57  DIC: 77 | |  | | | | | | | | | |
| Evaluation of consistency using the design-by-treatment interaction model | | Chi-square test: 15.52  Degrees of Freedom: 28  P- value: 0.97  Heterogeneity: 0.000 | | | | | | | | | |
| **Any Minor Congenital Malformations (9 studies, 614 cases, 17 treatments)** | | | | | | | | | | | |
| Carbam vs Control | | 2 (NA) | 62 (26.00) | Epilepsy | Whole PG | | H | H | 10.58 (1.27-323.10) | | 10.81 (1.40-373.90) (1.21-396.30) |
| Carbam+Pheno+Valpro vs Control | | NA | NR | NR | NR | | NR | NR | NA | | 122.20 (2.09-9,539.00) (2.02-1.04 x 10^4) |
| Carbam+Pheny vs Control | | 1 (NA) | 34 (NR) | Epilepsy | Whole PG | | H | H | 28.94  (1.65-9.60 x 10^3^) | | 12.46 (1.17-438.90) (1.04-492.40) |
| Oxcar vs Carbam | | 1 (NA) | 51 (24.60) | Epilepsy | 1st trimester | | H | H | 0.04 (0.00-0.86) | | 0.04 (0.00-0.67) (0.00-0.73) |
| Oxcar vs Carbam+Pheno+Valpro | | NA | NR | NR | NR | | NR | NR | NA | | 0.00 (0.00-0.30) (0.00-0.34) |
| Oxcar vs Pheno+Pheny | | NA | NR | NR | NR | | NR | NR | NA | | 0.02 (0.00-0.43) (0.00-0.49) |
| Oxcar vs Pheno+Valpro | | NA | NR | NR | NR | | NR | NR | NA | | 0.02 (0.00-0.93) (0.00-0.98) |
| Oxcar vs Pheny | | NA | NR | NR | NR | | NR | NR | NA | | 0.05 (0.00-0.98) (0.00-1.13) |
| Oxcar vs Valpro | | 1 (NA) | 56 (24.60) | Epilepsy | 1st trimester | | H | H | 0.03 (0.00-0.41) | | 0.03 (0.00-0.41) (0.00-0.46) |
| Pheno+Pheny vs Control | | 1 (NA) | 35 (26.00) | Epilepsy | NR | | H | H | 10.34  (0.75-3.37 x 10^3^) | | 20.14 (1.96-764.20)  (1.75-824.40) |
| Pheno+Pheny vs Pheno | | 2 (NA) | 111 (27.40) | Epilepsy | NR | | H | H | 4.63 (1.15-19.25) | | 4.58 (1.35-16.03) (1.05-20.62) |
| Valpro vs Control | | NA | NR | NR | NR | | NR | NR | NA | | 17.76 (1.60-633.30)  (1.40-691.10) |
| Valpro vs Pheno | | 3 (NA) | 126 (26.00) | Epilepsy | 1st trimester | | H | H | 4.46 (0.89-23.48) | | 3.93 (1.08-16.40) (0.85-20.52) |
| Common between-study variance across treatment comparisons | | | | | | | | | 0.06 (0.00-0.72) | | 0.07 (0.00-0.98) (NA) |
| Residual deviance:33  Data points: 37  DIC: 61 | |  | | | | | | | | | |
| Evaluation of consistency using the design-by-treatment interaction model | | Chi-square test: 4.25  Degrees of Freedom: 11  P- value: 0.96  Heterogeneity: 0.000 | | | | | | | | | |
| **Abbreviations:** CrI - Credible Interval; DIC - Deviance Information Criterion; H – high; L – low; MA - Meta-analysis; NA - Not Applicable; NMA - Network Meta-analysis; NR - Not Reported; PG - pregnancy;PrI - Predictive Interval; ROB - Risk of Bias;  Carbam = Carbamazepine; Clobaz = Clobazam; Clonaz = Clonazepam; Ethos = Ethosuximide; Gabap = Gabapentin; Lamot = Lamotrigine; Levet = Levetiracetam; Oxcar = Oxcarbazepine; Pheno = Phenobarbital; Pheny = Phenytoin; Primid = Primidone; Topir = Topiramate; Valpro = Valproate; Vigab = Vigabatrin  a The results presented here are only for the statistically significant NMA results.  b A fixed-effect model was applied, since each design in the network was represented by a single study. Hence, there were no degrees of freedom for heterogeneity. | | | | | | | | | | | |

## Appendix K. Characteristics of the treatment nodes per outcome along with their SUCRA values

| **Treatment** | **Frequency of treatment**  **in Network** | **# of Events/**  **Sample Size** | **Treatment Group Risk**  **Median (IQR)** | **SUCRA**  **Median (95% CrI)** |
| --- | --- | --- | --- | --- |
| **Overall Major Congenital Malformations** | | | | |
| Carbam | 62 | 350/8437 | 0.046 (0.026-0.094) | 0.66 (0.55-0.77) |
| Carbam+Clonaz | 5 | 4/24 | 0.125 (0.000-0.250) | 0.13 (0.00-0.38) |
| Carbam+Clonaz+Pheny | 1 | 1/12 | 0.083 (0.083-0.083) | 0.19 (0.00-0.91) |
| Carbam+Gabap | 1 | 0/5 | 0.000 (0.000-0.000) | 0.87 (0.02-1.00) |
| Carbam+Lamot | 1 | 0/8 | 0.000 (0.000-0.000) | 0.89 (0.21-1.00) |
| Carbam+Pheno | 13 | 9/142 | 0.026 (0.000-0.167) | 0.40 (0.23-0.64) |
| Carbam+Pheno+Pheny | 4 | 2/24 | 0.038 (0.000-0.138) | 0.36 (0.09-0.81) |
| Carbam+Pheno+Pheny+Primid | 1 | 1/6 | 0.167 (0.167-0.167) | 0.17 (0.00-0.89) |
| Carbam+Pheno+Valpro | 2 | 1/6 | 0.167 (0.000-0.333) | 0.45 (0.02-0.96) |
| Carbam+Pheny | 13 | 15/124 | 0.125 (0.000-0.182) | 0.34 (0.17-0.51) |
| Carbam+Pheny+Primid | 1 | 1/20 | 0.050 (0.050-0.050) | 0.55 (0.11-0.96) |
| Carbam+Pheny+Valpro | 2 | 1/10 | 0.125 (0.000-0.250) | 0.17 (0.00-0.77) |
| Carbam+Primid | 2 | 0/9 | 0.000 (0.000-0.000) | 0.70 (0.11-0.98) |
| Carbam+Valpro | 11 | 18/128 | 0.143 (0.000-0.214) | 0.19 (0.09-0.36) |
| Carbam+Vigab | 1 | 0/3 | 0.000 (0.000-0.000) | 0.57 (0.00-1.00) |
| Clobaz | 4 | 0/11 | 0.000 (0.000-0.000) | 0.34 (0.02-0.89) |
| Clobaz+Oxcar | 1 | 0/9 | 0.000 (0.000-0.000) | 0.89 (0.19-1.00) |
| Clonaz | 12 | 9/375 | 0.000 (0.000-0.009) | 0.72 (0.51-0.87) |
| Clonaz+Oxcar | 1 | 0/9 | 0.000 (0.000-0.000) | 0.91 (0.19-1.00) |
| Clonaz+Valpro | 1 | 1/6 | 0.167 (0.167-0.167) | 0.06 (0.00-0.70) |
| Control | 40 | 228/7275 | 0.026 (0.000-0.092) | 0.74 (0.64-0.87) |
| Ethos | 10 | 3/61 | 0.000 (0.000-0.542) | 0.38 (0.13-0.70) |
| Ethos+Pheno | 1 | 0/3 | 0.000 (0.000-0.000) | 0.55 (0.00-1.00) |
| Ethos+Pheny | 3 | 1/9 | 0.000 (0.000-0.250) | 0.36 (0.06-0.87) |
| Gabap | 7 | 8/329 | 0.032 (0.007-0.500) | 0.74 (0.53-0.91) |
| Gabap+Lamot | 1 | 0/3 | 0.000 (0.000-0.000) | 0.79 (0.00-1.00) |
| Gabap+Pheny | 1 | 0/6 | 0.000 (0.000-0.000) | 0.83 (0.04-1.00) |
| Lamot | 17 | 165/6290 | 0.000 (0.000-0.031) | 0.77 (0.66-0.87) |
| Lamot+Levet | 1 | 0/6 | 0.000 (0.000-0.000) | 0.83 (0.11-1.00) |
| Lamot+Valpro | 2 | 1/21 | 0.100 (0.000-0.200) | 0.77 (0.28-0.96) |
| Levet | 6 | 18/1015 | 0.017 (0.007-0.024) | 0.83 (0.68-0.94) |
| Oxcar | 11 | 11/372 | 0.000 (0.000-0.059) | 0.66 (0.45-0.87) |
| Oxcar+Valpro | 1 | 0/3 | 0.000 (0.000-0.000) | 0.66 (0.02-1.00) |
| Pheno | 46 | 94/1709 | 0.043 (0.000-0.107) | 0.55 (0.43-0.68) |
| Pheno+Pheny | 24 | 70/769 | 0.086 (0.007-0.157) | 0.47 (0.34-0.64) |
| Pheno+Pheny+Primid | 3 | 15/52 | 0.240 (0.111-0.778) | 0.09 (0.00-0.23) |
| Pheno+Pheny+Valpro | 1 | 0/8 | 0.000 (0.000-0.000) | 0.81 (0.09-1.00) |
| Pheno+Primid | 5 | 6/27 | 0.167 (0.143-0.500) | 0.06 (0.00-0.26) |
| Pheno+Valpro | 7 | 8/45 | 0.133 (0.000-0.500) | 0.23 (0.06-0.51) |
| Pheny | 48 | 136/2237 | 0.038 (0.000-0.092) | 0.57 (0.47-0.70) |
| Pheny+Primid | 8 | 8/58 | 0.038 (0.000-0.280) | 0.23 (0.09-0.47) |
| Pheny+Primid+Valpro | 1 | 1/6 | 0.167 (0.167-0.167) | 0.13 (0.00-0.83) |
| Pheny+Valpro | 6 | 4/27 | 0.125 (0.000-0.333) | 0.23 (0.06-0.53) |
| Primid | 14 | 9/224 | 0.000 (0.000-0.167) | 0.68 (0.49-0.87) |
| Primid+Valpro | 2 | 0/10 | 0.000 (0.000-0.000) | 0.64 (0.11-0.98) |
| Topir | 7 | 28/599 | 0.042 (0.024-0.043) | 0.53 (0.36-0.70) |
| Valpro | 52 | 404/4455 | 0.105 (0.040-0.163) | 0.38 (0.28-0.51) |
| Vigab | 3 | 1/23 | 0.000 (0.000-0.111) | 0.47 (0.13-0.89) |
| **Combined Fetal Losses** | | | | |
| Carbam | 28 | 111/3911 | 0.026 (0.000-0.065) | 0.78 (0.56-0.89) |
| Carbam+Pheno | 7 | 6/51 | 0.000 (0.000-0.273) | 0.44 (0.15-0.85) |
| Carbam+Pheno+Pheny | 2 | 0/6 | 0.000 (0.000-0.000) | 0.30 (0.00-1.00) |
| Carbam+Pheno+Valpro | 2 | 1/7 | 0.167 (0.000-0.333) | 0.22 (0.00-0.93) |
| Carbam+Pheny | 6 | 2/47 | 0.000 (0.000-0.000) | 0.44 (0.11-0.89) |
| Carbam+Primid | 1 | 0/3 | 0.000 (0.000-0.000) | 0.85 (0.04-1.00) |
| Carbam+Valpro | 4 | 2/26 | 0.036 (0.000-0.107) | 0.22 (0.04-0.70) |
| Clobaz | 3 | 0/7 | 0.000 (0.000-0.000) | 0.22 (0.00-0.96) |
| Clobaz+Oxcar | 1 | 0/9 | 0.000 (0.000-0.000) | 0.89 (0.04-1.00) |
| Clonaz | 4 | 0/31 | 0.000 (0.000-0.000) | 0.41 (0.07-0.96) |
| Clonaz+Oxcar | 1 | 0/9 | 0.000 (0.000-0.000) | 0.89 (0.07-1.00) |
| Control | 17 | 22/2286 | 0.000 (0.000-0.000) | 0.85 (0.63-0.96) |
| Ethos | 3 | 1/16 | 0.000 (0.000-0.100) | 0.52 (0.11-1.00) |
| Ethos+Pheny | 2 | 0/7 | 0.000 (0.000-0.000) | 0.33 (0.00-1.00) |
| Lamot | 8 | 101/2540 | 0.000 (0.000-0.063) | 0.70 (0.48-0.89) |
| Lamot+Valpro | 1 | 1/5 | 0.200 (0.200-0.200) | 0.22 (0.00-0.96) |
| Levet | 3 | 2/28 | 0.000 (0.000-0.182) | 0.44 (0.11-0.93) |
| Oxcar | 2 | 5/567 | 0.005 (0.000-0.009) | 0.63 (0.30-0.96) |
| Pheno | 16 | 9/407 | 0.000 (0.000-0.000) | 0.85 (0.63-1.00) |
| Pheno+Pheny | 8 | 6/133 | 0.033 (0.000-0.179) | 0.41 (0.15-0.89) |
| Pheno+Pheny+Primid | 1 | 1/9 | 0.111 (0.111-0.111) | 0.33 (0.00-0.96) |
| Pheno+Valpro | 4 | 1/24 | 0.000 (0.000-0.167) | 0.48 (0.11-0.96) |
| Pheny | 20 | 22/618 | 0.006 (0.000-0.038) | 0.67 (0.44-0.89) |
| Pheny+Primid | 2 | 0/7 | 0.000 (0.000-0.000) | 0.41 (0.00-1.00) |
| Pheny+Valpro | 3 | 2/11 | 0.000 (0.000-1.000) | 0.11 (0.00-0.52) |
| Primid | 3 | 15/108 | 0.000 (0.000-0.211) | 0.41 (0.19-0.74) |
| Topir | 1 | 1/2 | 0.500 (0.500-0.500) | 0.00 (0.00-0.78) |
| Valpro | 22 | 115/2612 | 0.047 (0.000-0.091) | 0.59 (0.37-0.74) |
| **Pre-natal Growth Retardation** | | | | |
| Carbam | 14 | 196/2897 | 0.058 (0.039-0.107) | 0.55 (0.36-0.77) |
| Carbam+Pheno | 3 | 0/19 | 0.000 (0.000-0.000) | 0.77 (0.14-1.00) |
| Carbam+Pheno+Pheny | 1 | 0/4 | 0.000 (0.000-0.000) | 0.45 (0.00-1.00) |
| Carbam+Pheno+Valpro | 1 | 0/3 | 0.000 (0.000-0.000) | 0.50 (0.00-1.00) |
| Carbam+Pheny | 1 | 1/11 | 0.091 (0.091-0.091) | 0.23 (0.00-0.95) |
| Carbam+Primid | 1 | 1/3 | 0.333 (0.333-0.333) | 0.05 (0.00-0.86) |
| Carbam+Valpro | 2 | 0/4 | 0.000 (0.000-0.000) | 0.32 (0.00-1.00) |
| Clobaz | 2 | 7/34 | 0.263 (0.194-0.333) | 0.14 (0.00-0.41) |
| Clonaz | 3 | 22/411 | 0.012 (0.000-0.084) | 0.55 (0.27-0.86) |
| Control | 10 | 333/7347 | 0.047 (0.024-0.100) | 0.64 (0.41-0.86) |
| Ethos+Pheny | 1 | 0/3 | 0.000 (0.000-0.000) | 0.32 (0.00-1.00) |
| Gabap | 2 | 5/70 | 0.037 (0.000-0.074) | 0.45 (0.14-0.91) |
| Lamot | 7 | 166/2882 | 0.057 (0.017-0.174) | 0.73 (0.45-0.91) |
| Levet | 2 | 4/81 | 0.058 (0.036-0.080) | 0.50 (0.14-0.95) |
| Oxcar | 2 | 36/1002 | 0.041 (0.027-0.055) | 0.68 (0.36-0.91) |
| Pheno | 7 | 38/400 | 0.154 (0.058-0.173) | 0.32 (0.14-0.59) |
| Pheno+Pheny | 1 | 0/15 | 0.000 (0.000-0.000) | 0.91 (0.09-1.00) |
| Pheny | 10 | 20/519 | 0.035 (0.019-0.060) | 0.82 (0.55-0.95) |
| Pheny+Valpro | 1 | 0/5 | 0.000 (0.000-0.000) | 0.73 (0.00-1.00) |
| Primid | 2 | 8/52 | 0.131 (0.056-0.206) | 0.27 (0.05-0.73) |
| Topir | 4 | 85/472 | 0.221 (0.127-0.465) | 0.23 (0.05-0.45) |
| Valpro | 11 | 86/1622 | 0.070 (0.030-0.121) | 0.50 (0.27-0.73) |
| Vigab | 1 | 1/13 | 0.077 (0.077-0.077) | 0.64 (0.05-1.00) |
| **Preterm Birth** | | | | |
| Carbam | 13 | 119/2141 | 0.056 (0.029-0.091) | 0.45 (0.27-0.73) |
| Carbam+Pheno | 1 | 1/12 | 0.083 (0.083-0.083) | 0.64 (0.00-1.00) |
| Carbam+Pheny | 1 | 0/3 | 0.000 (0.000-0.000) | 0.14 (0.00-1.00) |
| Carbam+Primid | 1 | 0/10 | 0.000 (0.000-0.000) | 0.82 (0.00-1.00) |
| Clobaz | 3 | 7/35 | 0.167 (0.000-1.000) | 0.09 (0.00-0.36) |
| Clobaz+Oxcar | 1 | 0/9 | 0.000 (0.000-0.000) | 0.91 (0.05-1.00) |
| Clonaz | 4 | 24/437 | 0.003 (0.000-0.047) | 0.68 (0.32-0.91) |
| Clonaz+Oxcar | 1 | 0/9 | 0.000 (0.000-0.000) | 0.86 (0.05-1.00) |
| Control | 8 | 480/7404 | 0.051 (0.025-0.072) | 0.55 (0.32-0.77) |
| Ethos | 2 | 0/19 | 0.000 (0.000-0.000) | 0.50 (0.00-1.00) |
| Ethos+Primid | 1 | 1/26 | 0.038 (0.038-0.038) | 0.41 (0.00-0.95) |
| Gabap | 3 | 10/76 | 0.125 (0.000-0.500) | 0.23 (0.05-0.64) |
| Lamot | 10 | 229/3015 | 0.085 (0.061-0.143) | 0.50 (0.27-0.77) |
| Levet | 3 | 5/93 | 0.038 (0.036-0.182) | 0.64 (0.18-0.91) |
| Oxcar | 4 | 44/1045 | 0.031 (0.014-0.049) | 0.73 (0.41-0.86) |
| Pheno | 8 | 17/206 | 0.026 (0.000-0.094) | 0.27 (0.09-0.64) |
| Pheno+Pheny | 1 | 0/2 | 0.000 (0.000-0.000) | 0.14 (0.00-1.00) |
| Pheny | 10 | 14/283 | 0.013 (0.000-0.061) | 0.55 (0.23-0.86) |
| Pheny+Primid | 1 | 1/25 | 0.040 (0.040-0.040) | 0.41 (0.00-0.95) |
| Primid | 4 | 14/168 | 0.123 (0.055-0.218) | 0.18 (0.05-0.50) |
| Topir | 3 | 41/408 | 0.085 (0.000-0.104) | 0.36 (0.14-0.73) |
| Valpro | 13 | 84/1694 | 0.045 (0.009-0.069) | 0.59 (0.32-0.82) |
| Vigab | 1 | 0/13 | 0.000 (0.000-0.000) | 0.91 (0.09-1.00) |
| **Cardiac Malformations** | | | | |
| Carbam | 44 | 74/6336 | 0.004 (0.000-0.029) | 0.67 (0.54-0.79) |
| Carbam+Clonaz | 2 | 1/9 | 0.071 (0.000-0.143) | 0.10 (0.00-0.54) |
| Carbam+Clonaz+Pheny | 1 | 1/12 | 0.083 (0.083-0.083) | 0.15 (0.00-0.82) |
| Carbam+Lamot | 1 | 0/8 | 0.000 (0.000-0.000) | 0.90 (0.26-1.00) |
| Carbam+Pheno | 7 | 0/37 | 0.000 (0.000-0.000) | 0.44 (0.15-0.85) |
| Carbam+Pheno+Pheny | 2 | 0/6 | 0.000 (0.000-0.000) | 0.33 (0.03-0.95) |
| Carbam+Pheno+Valpro | 1 | 0/3 | 0.000 (0.000-0.000) | 0.41 (0.00-1.00) |
| Carbam+Pheny | 7 | 4/42 | 0.091 (0.000-0.500) | 0.18 (0.03-0.41) |
| Carbam+Pheny+Valpro | 1 | 0/4 | 0.000 (0.000-0.000) | 0.31 (0.00-1.00) |
| Carbam+Primid | 1 | 0/3 | 0.000 (0.000-0.000) | 0.46 (0.00-1.00) |
| Carbam+Valpro | 6 | 0/44 | 0.000 (0.000-0.000) | 0.54 (0.23-0.87) |
| Clobaz | 3 | 0/8 | 0.000 (0.000-0.000) | 0.23 (0.00-0.82) |
| Clobaz+Oxcar | 1 | 0/9 | 0.000 (0.000-0.000) | 0.82 (0.10-1.00) |
| Clonaz | 6 | 0/47 | 0.000 (0.000-0.000) | 0.36 (0.10-0.79) |
| Clonaz+Oxcar | 1 | 0/9 | 0.000 (0.000-0.000) | 0.79 (0.13-1.00) |
| Clonaz+Valpro | 1 | 0/6 | 0.000 (0.000-0.000) | 0.33 (0.00-0.97) |
| Control | 27 | 31/2102 | 0.000 (0.000-0.027) | 0.64 (0.51-0.79) |
| Ethos | 6 | 0/33 | 0.000 (0.000-0.000) | 0.33 (0.08-0.72) |
| Ethos+Pheno | 1 | 0/3 | 0.000 (0.000-0.000) | 0.26 (0.00-0.97) |
| Ethos+Pheny | 2 | 0/7 | 0.000 (0.000-0.000) | 0.41 (0.05-0.95) |
| Gabap | 3 | 2/35 | 0.032 (0.000-0.500) | 0.21 (0.03-0.51) |
| Lamot | 12 | 28/4788 | 0.000 (0.000-0.004) | 0.79 (0.69-0.92) |
| Lamot+Levet | 1 | 0/6 | 0.000 (0.000-0.000) | 0.85 (0.23-1.00) |
| Lamot+Valpro | 1 | 0/16 | 0.000 (0.000-0.004) | 0.92 (0.44-1.00) |
| Levet | 2 | 1/754 | 0.001 (0.000-0.002) | 0.90 (0.67-1.00) |
| Oxcar | 7 | 0/346 | 0.000 (0.000-0.000) | 0.74 (0.41-0.92) |
| Oxcar+Valpro | 1 | 0/3 | 0.000 (0.000-0.004) | 0.36 (0.00-0.97) |
| Pheno | 30 | 26/1255 | 0.000 (0.000-0.030) | 0.51 (0.36-0.69) |
| Pheno+Pheny | 13 | 12/477 | 0.000 (0.000-0.034) | 0.62 (0.38-0.82) |
| Pheno+Pheny+Primid | 1 | 2/9 | 0.222 (0.222-0.222) | 0.21 (0.00-0.72) |
| Pheno+Primid | 2 | 0/5 | 0.000 (0.000-0.000) | 0.31 (0.00-0.97) |
| Pheno+Valpro | 3 | 1/8 | 0.000 (0.000-0.333) | 0.15 (0.00-0.59) |
| Pheny | 35 | 21/1697 | 0.000 (0.000-0.024) | 0.67 (0.51-0.82) |
| Pheny+Primid | 4 | 0/13 | 0.000 (0.000-0.000) | 0.38 (0.08-0.90) |
| Pheny+Valpro | 4 | 3/17 | 0.167 (0.000-0.417) | 0.13 (0.00-0.38) |
| Primid | 9 | 1/133 | 0.000 (0.000-0.000) | 0.67 (0.41-0.90) |
| Primid+Valpro | 1 | 0/7 | 0.000 (0.000-0.000) | 0.79 (0.15-1.00) |
| Topir | 2 | 2/429 | 0.003 (0.000-0.006) | 0.77 (0.44-0.95) |
| Valpro | 37 | 69/3194 | 0.015 (0.000-0.034) | 0.51 (0.38-0.64) |
| Vigab | 2 | 0/15 | 0.000 (0.000-0.000) | 0.38 (0.05-0.95) |
| **Hypospadias** | | | | |
| Carbam | 28 | 21/3540 | 0.003 (0.000-0.022) | 0.71 (0.55-0.87) |
| Carbam+Clonaz | 3 | 1/13 | 0.000 (0.000-0.250) | 0.26 (0.03-0.77) |
| Carbam+Lamot | 1 | 0/8 | 0.000 (0.000-0.000) | 0.71 (0.06-1.00) |
| Carbam+Pheno | 5 | 1/29 | 0.000 (0.000-0.000) | 0.32 (0.06-0.74) |
| Carbam+Pheno+Pheny | 2 | 0/6 | 0.000 (0.000-0.000) | 0.48 (0.03-0.97) |
| Carbam+Pheny | 3 | 0/19 | 0.000 (0.000-0.000) | 0.48 (0.10-0.94) |
| Carbam+Pheny+Valpro | 1 | 0/4 | 0.000 (0.000-0.000) | 0.26 (0.00-1.00) |
| Carbam+Valpro | 4 | 1/34 | 0.000 (0.000-0.045) | 0.35 (0.06-0.77) |
| Clobaz | 2 | 0/6 | 0.000 (0.000-0.000) | 0.13 (0.00-0.90) |
| Clonaz | 5 | 0/42 | 0.000 (0.000-0.000) | 0.26 (0.03-0.68) |
| Clonaz+Valpro | 1 | 0/6 | 0.000 (0.000-0.000) | 0.32 (0.00-1.00) |
| Control | 12 | 6/488 | 0.000 (0.000-0.015) | 0.74 (0.55-0.90) |
| Ethos | 6 | 0/31 | 0.000 (0.000-0.000) | 0.23 (0.03-0.68) |
| Ethos+Pheny | 2 | 0/7 | 0.000 (0.000-0.000) | 0.55 (0.06-0.97) |
| Gabap | 2 | 2/33 | 0.500 (0.000-1.000) | 0.06 (0.00-0.48) |
| Lamot | 8 | 4/95 | 0.000 (0.000-0.000) | 0.84 (0.58-0.97) |
| Lamot+Levet | 1 | 0/6 | 0.000 (0.000-0.000) | 0.61 (0.03-1.00) |
| Lamot+Valpro | 1 | 0/16 | 0.000 (0.000-0.000) | 0.84 (0.16-1.00) |
| Levet | 2 | 0/754 | 0.000 (0.000-0.000) | 0.94 (0.52-1.00) |
| Oxcar | 4 | 1/200 | 0.000 (0.000-0.003) | 0.29 (0.06-0.74) |
| Pheno | 21 | 5/1024 | 0.000 (0.000-0.000) | 0.61 (0.42-0.84) |
| Pheno+Pheny | 5 | 1/175 | 0.000 (0.000-0.000) | 0.74 (0.42-0.97) |
| Pheno+Pheny+Primid | 1 | 0/9 | 0.000 (0.000-0.000) | 0.87 (0.16-1.00) |
| Pheno+Primid | 1 | 0/2 | 0.000 (0.000-0.000) | 0.55 (0.00-1.00) |
| Pheno+Valpro | 2 | 0/6 | 0.000 (0.000-0.000) | 0.29 (0.00-0.94) |
| Pheny | 23 | 11/1350 | 0.000 (0.000-0.019) | 0.71 (0.52-0.87) |
| Pheny+Primid | 2 | 0/7 | 0.000 (0.000-0.000) | 0.52 (0.06-0.97) |
| Pheny+Valpro | 2 | 0/9 | 0.000 (0.000-0.000) | 0.61 (0.10-0.97) |
| Primid | 5 | 1/74 | 0.000 (0.000-0.000) | 0.29 (0.03-0.71) |
| Topir | 2 | 3/429 | 0.010 (0.006-0.014) | 0.42 (0.10-0.77) |
| Valpro | 25 | 29/1437 | 0.000 (0.000-0.036) | 0.48 (0.32-0.65) |
| Vigab | 1 | 0/6 | 0.000 (0.000-0.000) | 0.13 (0.00-1.00) |
| **Cleft Lip/Palate** | | | | |
| Carbam | 22 | 27/5577 | 0.001 (0.000-0.014) | 0.84 (0.72-0.94) |
| Carbam+Clonaz | 1 | 0/7 | 0.000 (0.000-0.000) | 0.53 (0.03-1.00) |
| Carbam+Pheno | 3 | 2/16 | 0.000 (0.000-0.400) | 0.25 (0.03-0.66) |
| Carbam+Pheno+Pheny | 1 | 0/2 | 0.000 (0.000-0.000) | 0.44 (0.00-1.00) |
| Carbam+Pheno+Valpro | 2 | 0/6 | 0.000 (0.000-0.000) | 0.53 (0.03-1.00) |
| Carbam+Pheny | 2 | 0/8 | 0.000 (0.000-0.000) | 0.31 (0.00-0.94) |
| Carbam+Pheny+Valpro | 1 | 0/4 | 0.000 (0.000-0.000) | 0.28 (0.00-1.00) |
| Carbam+Primid | 1 | 0/3 | 0.000 (0.000-0.000) | 0.66 (0.03-1.00) |
| Carbam+Valpro | 3 | 2/26 | 0.071 (0.000-0.143) | 0.22 (0.03-0.59) |
| Clobaz | 2 | 0/6 | 0.000 (0.000-0.000) | 0.19 (0.00-0.91) |
| Clonaz | 2 | 0/18 | 0.000 (0.000-0.000) | 0.31 (0.00-0.97) |
| Clonaz+Valpro | 1 | 0/6 | 0.000 (0.000-0.000) | 0.34 (0.00-1.00) |
| Control | 11 | 3/1649 | 0.000 (0.000-0.000) | 0.88 (0.72-1.00) |
| Ethos | 5 | 1/29 | 0.000 (0.000-0.000) | 0.22 (0.03-0.56) |
| Ethos+Pheno | 1 | 0/3 | 0.000 (0.000-0.000) | 0.28 (0.00-1.00) |
| Ethos+Pheny | 2 | 0/6 | 0.000 (0.000-0.000) | 0.38 (0.00-0.97) |
| Gabap | 2 | 0/33 | 0.000 (0.000-0.000) | 0.56 (0.13-0.97) |
| Lamot | 7 | 22/4664 | 0.000 (0.000-0.004) | 0.84 (0.72-0.97) |
| Levet | 3 | 1/872 | 0.000 (0.000-0.008) | 0.97 (0.78-1.00) |
| Oxcar | 5 | 1/304 | 0.000 (0.000-0.000) | 0.66 (0.38-0.91) |
| Oxcar+Valpro | 1 | 0/3 | 0.000 (0.000-0.000) | 0.28 (0.00-1.00) |
| Pheno | 16 | 17/894 | 0.016 (0.000-0.046) | 0.53 (0.34-0.72) |
| Pheno+Pheny | 9 | 3/394 | 0.000 (0.000-0.009) | 0.75 (0.53-0.94) |
| Pheno+Pheny+Primid | 2 | 2/27 | 0.111 (0.000-0.222) | 0.34 (0.06-0.75) |
| Pheno+Primid | 3 | 0/7 | 0.000 (0.000-0.000) | 0.25 (0.00-0.88) |
| Pheno+Valpro | 2 | 1/19 | 0.031 (0.000-0.063) | 0.47 (0.00-0.94) |
| Pheny | 21 | 10/1172 | 0.000 (0.000-0.012) | 0.69 (0.50-0.84) |
| Pheny+Primid | 4 | 2/22 | 0.038 (0.000-0.288) | 0.25 (0.03-0.63) |
| Pheny+Valpro | 1 | 0/4 | 0.000 (0.000-0.000) | 0.56 (0.03-1.00) |
| Primid | 7 | 0/41 | 0.000 (0.000-0.000) | 0.44 (0.16-0.78) |
| Topir | 2 | 7/429 | 0.021 (0.014-0.029) | 0.50 (0.25-0.75) |
| Valpro | 17 | 35/2721 | 0.000 (0.000-0.014) | 0.66 (0.50-0.81) |
| Vigab | 2 | 0/15 | 0.000 (0.000-0.000) | 0.34 (0.03-0.97) |
| **Club Foot** | | | | |
| Carbam | 18 | 12/99 | 0.000 (0.000-0.009) | 0.69 (0.50-0.85) |
| Carbam+Clonaz | 1 | 0/7 | 0.000 (0.000-0.000) | 0.38 (0.00-1.00) |
| Carbam+Pheno | 4 | 1/26 | 0.000 (0.000-0.042) | 0.31 (0.04-0.73) |
| Carbam+Pheno+Pheny | 1 | 0/2 | 0.000 (0.000-0.000) | 0.46 (0.00-1.00) |
| Carbam+Pheny | 2 | 0/8 | 0.000 (0.000-0.000) | 0.27 (0.00-0.92) |
| Carbam+Valpro | 2 | 1/21 | 0.071 (0.000-0.143) | 0.27 (0.04-0.77) |
| Clobaz | 1 | 0/2 | 0.000 (0.000-0.000) | 0.15 (0.00-1.00) |
| Clonaz | 3 | 0/34 | 0.000 (0.000-0.000) | 0.42 (0.04-0.92) |
| Control | 14 | 3/777 | 0.000 (0.000-0.000) | 0.81 (0.62-0.96) |
| Ethos | 4 | 0/10 | 0.000 (0.000-0.000) | 0.15 (0.00-0.65) |
| Ethos+Pheno | 1 | 0/3 | 0.000 (0.000-0.000) | 0.38 (0.00-1.00) |
| Ethos+Pheny | 1 | 0/4 | 0.000 (0.000-0.000) | 0.58 (0.04-1.00) |
| Gabap | 1 | 0/2 | 0.000 (0.000-0.000) | 0.35 (0.00-1.00) |
| Lamot | 4 | 1/1621 | 0.000 (0.000-0.012) | 0.85 (0.58-1.00) |
| Levet | 1 | 0/450 | 0.000 (0.000-0.000) | 0.96 (0.46-1.00) |
| Oxcar | 3 | 0/198 | 0.000 (0.000-0.000) | 0.58 (0.15-0.96) |
| Pheno | 18 | 4/1057 | 0.000 (0.000-0.000) | 0.73 (0.50-0.92) |
| Pheno+Pheny | 6 | 2/321 | 0.000 (0.000-0.009) | 0.81 (0.46-0.96) |
| Pheno+Pheny+Primid | 1 | 2/9 | 0.222 (0.222-0.222) | 0.15 (0.00-0.65) |
| Pheno+Primid | 2 | 0/5 | 0.000 (0.000-0.000) | 0.31 (0.00-0.92) |
| Pheno+Valpro | 2 | 0/6 | 0.000 (0.000-0.000) | 0.27 (0.00-0.92) |
| Pheny | 17 | 10/932 | 0.005 (0.000-0.030) | 0.54 (0.35-0.73) |
| Pheny+Primid | 2 | 0/7 | 0.000 (0.000-0.000) | 0.35 (0.04-0.96) |
| Pheny+Valpro | 1 | 0/4 | 0.000 (0.000-0.000) | 0.54 (0.00-1.00) |
| Primid | 6 | 1/70 | 0.000 (0.000-0.000) | 0.38 (0.15-0.73) |
| Topir | 1 | 1/359 | 0.003 (0.003-0.003) | 0.65 (0.19-0.96) |
| Valpro | 15 | 14/802 | 0.015 (0.000-0.043) | 0.50 (0.27-0.65) |
| **Inguinal Hernia** | | | | |
| Carbam | 12 | 13/3307 | 0.008 (0.000-0.036) | 0.71 (0.50-0.89) |
| Carbam+Clonaz | 2 | 0/9 | 0.000 (0.000-0.000) | 0.36 (0.07-0.96) |
| Carbam+Pheno | 3 | 0/16 | 0.000 (0.000-0.000) | 0.50 (0.14-0.96) |
| Carbam+Pheno+Pheny | 2 | 0/6 | 0.000 (0.000-0.000) | 0.32 (0.04-0.96) |
| Carbam+Pheno+Valpro | 1 | 0/3 | 0.000 (0.000-0.000) | 0.50 (0.04-1.00) |
| Carbam+Pheny | 3 | 1/19 | 0.000 (0.000-0.167) | 0.32 (0.11-0.79) |
| Carbam+Primid | 1 | 0/3 | 0.000 (0.000-0.000) | 0.54 (0.04-1.00) |
| Carbam+Valpro | 3 | 0/23 | 0.000 (0.000-0.000) | 0.50 (0.14-1.00) |
| Clobaz | 1 | 0/2 | 0.000 (0.000-0.000) | 0.25 (0.00-1.00) |
| Clonaz | 2 | 0/15 | 0.000 (0.000-0.000) | 0.21 (0.04-0.96) |
| Control | 5 | 0/203 | 0.000 (0.000-0.000) | 0.82 (0.46-1.00) |
| Ethos | 3 | 0/18 | 0.000 (0.000-0.000) | 0.25 (0.04-0.86) |
| Ethos+Pheny | 2 | 0/7 | 0.000 (0.000-0.000) | 0.39 (0.07-1.00) |
| Gabap | 1 | 0/31 | 0.000 (0.000-0.000) | 0.25 (0.04-1.00) |
| Lamot | 4 | 5/81 | 0.000 (0.000-0.002) | 0.86 (0.57-1.00) |
| Levet | 2 | 2/754 | 0.003 (0.002-0.003) | 0.68 (0.32-0.96) |
| Oxcar | 2 | 0/189 | 0.000 (0.000-0.000) | 0.79 (0.29-1.00) |
| Pheno | 7 | 2/484 | 0.000 (0.000-0.011) | 0.79 (0.50-0.96) |
| Pheno+Pheny | 4 | 7/110 | 0.057 (0.020-0.120) | 0.39 (0.18-0.64) |
| Pheno+Pheny+Primid | 1 | 1/9 | 0.111 (0.111-0.111) | 0.36 (0.07-0.93) |
| Pheno+Primid | 1 | 2/2 | 1.000 (1.000-1.000) | 0.00 (0.00-0.14) |
| Pheno+Valpro | 1 | 0/3 | 0.000 (0.000-0.000) | 0.54 (0.07-1.00) |
| Pheny | 11 | 4/878 | 0.000 (0.000-0.005) | 0.71 (0.50-0.93) |
| Pheny+Primid | 2 | 0/7 | 0.000 (0.000-0.000) | 0.43 (0.07-1.00) |
| Pheny+Valpro | 2 | 1/9 | 0.125 (0.000-0.250) | 0.21 (0.04-0.79) |
| Primid | 2 | 1/48 | 0.011 (0.000-0.022) | 0.50 (0.14-0.93) |
| Topir | 2 | 1/429 | 0.001 (0.000-0.003) | 0.71 (0.32-1.00) |
| Valpro | 8 | 6/1845 | 0.000 (0.000-0.015) | 0.68 (0.46-0.93) |
| Vigab | 1 | 0/6 | 0.000 (0.000-0.000) | 0.07 (0.00-0.96) |
| **Undescended Testes** | | | | |
| Carbam | 7 | 3/1386 | 0.000 (0.000-0.002) | 0.69 (0.38-0.94) |
| Carbam+Pheno | 1 | 0/6 | 0.000 (0.000-0.000) | 0.38 (0.00-1.00) |
| Carbam+Pheny | 1 | 0/6 | 0.000 (0.000-0.000) | 0.38 (0.00-1.00) |
| Carbam+Valpro | 1 | 0/7 | 0.000 (0.000-0.000) | 0.44 (0.00-1.00) |
| Clonaz | 2 | 0/11 | 0.000 (0.000-0.000) | 0.19 (0.00-0.94) |
| Control | 7 | 3/205 | 0.000 (0.000-0.026) | 0.50 (0.19-0.81) |
| Ethos | 2 | 0/6 | 0.000 (0.000-0.000) | 0.19 (0.00-0.94) |
| Lamot | 2 | 2/1660 | 0.001 (0.000-0.001) | 0.81 (0.44-1.00) |
| Levet | 1 | 4/450 | 0.009 (0.009-0.009) | 0.25 (0.00-0.69) |
| Oxcar | 1 | 0/182 | 0.000 (0.000-0.000) | 0.88 (0.13-1.00) |
| Pheno | 8 | 7/526 | 0.000 (0.000-0.008) | 0.50 (0.19-0.81) |
| Pheno+Pheny | 4 | 3/234 | 0.005 (0.015-0.038) | 0.75 (0.31-0.94) |
| Pheno+Valpro | 1 | 0/3 | 0.000 (0.000-0.000) | 0.19 (0.00-1.00) |
| Pheny | 8 | 6/629 | 0.000 (0.000-0.011) | 0.38 (0.13-0.75) |
| Primid | 3 | 2/58 | 0.022 (0.000-0.100) | 0.19 (0.00-0.63) |
| Topir | 1 | 0/359 | 0.000 (0.000-0.000) | 0.94 (0.25-1.00) |
| Valpro | 7 | 5/542 | 0.006 (0.000-0.056) | 0.44 (0.19-0.75) |
| **Minor Congenital Malformations** | | | | |
| Carbam | 8 | 49/164 | 0.121 (0.155-0.243) | 0.38 (0.13-0.63) |
| Carbam+Clonaz | 1 | 0/4 | 0.000 (0.000-0.000) | 0.63 (0.00-1.00) |
| Carbam+Pheno | 2 | 2/30 | 0.037 (0.000-0.074) | 0.63 (0.19-0.88) |
| Carbam+Pheno+Pheny | 1 | 1/5 | 0.200 (0.200-0.200) | 0.31 (0.00-0.88) |
| Carbam+Pheno+Valpro | 1 | 1/3 | 0.333 (0.333-0.333) | 0.00 (0.00-0.63) |
| Carbam+Pheny | 1 | 4/16 | 0.250 (0.250-0.250) | 0.38 (0.00-0.81) |
| Clobaz+Oxcar | 1 | 0/9 | 0.000 (0.000-0.000) | 0.81 (0.13-1.00) |
| Clonaz | 1 | 0/3 | 0.000 (0.000-0.000) | 0.63 (0.00-1.00) |
| Clonaz+Oxcar | 1 | 0/9 | 0.000 (0.000-0.000) | 0.81 (0.13-1.00) |
| Control | 2 | 0/28 | 0.000 (0.000-0.000) | 0.88 (0.50-1.00) |
| Oxcar | 1 | 0/35 | 0.000 (0.000-0.000) | 0.94 (0.56-1.00) |
| Pheno | 4 | 8/127 | 0.016 (0.000-0.057) | 0.63 (0.38-0.88) |
| Pheno+Pheny | 4 | 11/56 | 0.243 (0.100-0.286) | 0.19 (0.06-0.50) |
| Pheno+Valpro | 2 | 2/20 | 0.063 (0.000-0.125) | 0.25 (0.06-0.75) |
| Pheny | 3 | 31/65 | 0.227 (0.000-0.788) | 0.44 (0.13-0.75) |
| Primid | 1 | 1/9 | 0.111 (0.111-0.111) | 0.38 (0.00-0.88) |
| Valpro | 3 | 6/31 | 0.190 (0.000-0.333) | 0.25 (0.00-0.63) |
| **Abbreviations:** CrI - Credible Interval; IQR - Interquartile Range; SUCRA - surface under the cumulative ranking curve;  Carbam = Carbamazepine; Clobaz = Clobazam; Clonaz = Clonazepam; Ethos = Ethosuximide; Gabap = Gabapentin; Lamot = Lamotrigine; Levet = Levetiracetam; Oxcar = Oxcarbazepine; Pheno = Phenobarbital; Pheny = Phenytoin; Primid = Primidone; Topir = Topiramate; Valpro = Valproate; Vigab = Vigabatrin | | | | |

## Appendix L. Network characteristics per outcome

|  | Total studies | | # of cohort or case-control studies | # of RCTs | Range of study arms | # of treatments | | # of patients | | # of direct treatment comparisons | | # of NMA treatment comparisons | | | Statistically significant NMA treatment effects | # of studies with zero events in all arms | # of studies with ineligible outcome deifinition |
| --- | --- | --- | --- | --- | --- | --- | --- | --- | --- | --- | --- | --- | --- | --- | --- | --- | --- |
| Overall major congenital malformations | | | | | | | | | | | | | | | | | |
|  | | 78 | 77 | 1 | (2,17) | | 48 | | 35016 | | 448 | | 1128 | 166 | | 0 | 0 |
| Combined Fetal losses | | | | | | | | | | | | | | | | | |
|  | | 31 | 30 | 1 | (2,12) | | 28 | | 13487 | | 181 | | 378 | 18 | | 2 | 2 |
| Pre-natal growth retardation | | | | | | | | | | | | | | | | | |
|  | | 16 | 16 | 0 | (2,14) | | 23 | | 18117 | | 136 | | 253 | 19 | | 0 | 6 |
| Preterm birth | | | | | | | | | | | | | | | | | |
|  | | 17 | 17 | 0 | (2,14) | | 23 | | 17133 | | 147 | | 253 | 13 | | 0 | 1 |
| Cardiac malformations | | | | | | | | | | | | | | | | | |
|  | | 51 | 51 | 0 | (2,12) | | 40 | | 21935 | | 295 | | 780 | 85 | | 18 | 0 |
| Hypospadias | | | | | | | | | | | | | | | | | |
|  | | 31 | 31 | 0 | (2,12) | | 32 | | 12365 | | 238 | | 496 | 34 | | 24 | 0 |
| Cleft lip/palate | | | | | | | | | | | | | | | | | |
|  | | 29 | 29 | 0 | (2,12) | | 33 | | 18987 | | 233 | | 528 | 58 | | 39 | 0 |
| Club foot | | | | | | | | | | | | | | | | | |
|  | | 23 | 23 | 0 | (2,12) | | 27 | | 8836 | | 179 | | 351 | 23 | | 41 | 0 |
| Inguinal hernia | | | | | | | | | | | | | | | | | |
|  | | 13 | 13 | 0 | (2,12) | | 29 | | 12216 | | 205 | | 406 | 31 | | 52 | 0 |
| Undescended testes | | | | | | | | | | | | | | | | | |
|  | | 10 | 10 | 0 | (2,12) | | 17 | | 6270 | | 94 | | 136 | 4 | | 46 | 0 |
| Any minor congenital malformations | | | | | | | | | | | | | | | | | |
|  | | 9 | 8 | 1 | (2,7) | | 17 | | 614 | | 59 | | 136 | 13 | | 0 | 0 |
| Abbreviations: NMA - Network Meta-analysis; RCTs - Randomized Controlled Trials; | | | | | | | | | | | | | | | | | |

## Appendix M. Meta-regression, subgroup and sensitivity analyses results

### Statistically Significant Meta-Regression results for Major Congenital Malformations

| **Treatment Comparison** | **Median Odds Ratio** | **Low CrI-High CrI** |
| --- | --- | --- |
| **Major Congenital Malformations – Baseline Risk** | | |
| Carbam vs Control | 1.47 | 1.07-1.93 |
| Carbam vs Pheno+Pheny | 0.65 | 0.44-0.90 |
| Carbam+Clonaz vs Carbam | 6.52 | 2.17-19.44 |
| Carbam+Clonaz vs Clonaz | 7.95 | 2.33-37.94 |
| Carbam+Clonaz vs Control | 9.51 | 3.07-29.32 |
| Carbam+Clonaz vs Pheno | 5.03 | 1.61-16.78 |
| Carbam+Clonaz vs Pheno+Pheny | 4.12 | 1.30-15.40 |
| Carbam+Clonaz vs Pheny | 5.32 | 1.74-18.59 |
| Carbam+Clonaz vs Primid | 7.84 | 2.13-23.47 |
| Carbam+Lamot vs Carbam+Clonaz | 0.04 | 0.00-0.84 |
| Carbam+Lamot vs Pheno+Pheny+Primid | 0.03 | 0.00-0.67 |
| Carbam+Lamot vs Pheno+Primid | 0.03 | 0.00-0.65 |
| Carbam+Pheno vs Carbam | 1.89 | 1.04-3.48 |
| Carbam+Pheno vs Control | 2.73 | 1.35-5.30 |
| Carbam+Pheno+Pheny vs Control | 3.94 | 1.08-10.25 |
| Carbam+Pheny vs Carbam | 2.73 | 1.53-4.52 |
| Carbam+Pheny vs Control | 4.03 | 2.09-6.72 |
| Carbam+Pheny vs Pheno | 2.16 | 1.12-3.62 |
| Carbam+Pheny vs Pheny | 2.29 | 1.26-3.76 |
| Carbam+Pheny+primid vs Pheno+Primid | 0.14 | 0.01-0.98 |
| Carbam+Primid vs Pheno+Pheny+Primid | 0.14 | 0.00-0.97 |
| Carbam+Primid vs Pheno+Primid | 0.13 | 0.00-0.92 |
| Carbam+Valpro vs Carbam | 4.54 | 2.62-7.24 |
| Carbam+Valpro vs Carbam+Pheno | 2.35 | 1.09-4.89 |
| Carbam+Valpro vs Clonaz | 5.55 | 2.51-11.80 |
| Carbam+Valpro vs Control | 6.67 | 3.41-10.95 |
| Carbam+Valpro vs Pheno | 3.46 | 1.91-5.83 |
| Carbam+Valpro vs Pheno+Pheny | 2.92 | 1.49-4.96 |
| Carbam+Valpro vs Pheny | 3.72 | 2.09-6.04 |
| Carbam+Valpro vs Primid | 4.99 | 2.46-11.43 |
| Carbam+Valpro vs Valpro | 2.14 | 1.19-3.35 |
| Clobaz+Oxcar vs Carbam+Clonaz | 0.05 | 0.00-0.88 |
| Clobaz+Oxcar vs Pheno+Pheny+Primid | 0.04 | 0.00-0.62 |
| Clobaz+Oxcar vs Pheno+Primid | 0.04 | 0.00-0.65 |
| Clonaz vs Carbam+Pheno | 0.42 | 0.19-0.96 |
| Clonaz vs Carbam+Pheny | 0.29 | 0.14-0.68 |
| Clonaz vs Ethos | 0.35 | 0.13-0.98 |
| Clonaz vs Pheno+Pheny | 0.50 | 0.27-0.97 |
| Clonaz vs Pheno+Pheny+Primid | 0.10 | 0.04-0.26 |
| Clonaz vs Pheno+Primid | 0.09 | 0.04-0.27 |
| Clonaz vs Pheny+Primid | 0.23 | 0.09-0.57 |
| Clonaz+Oxcar vs Carbam+Clonaz | 0.05 | 0.00-0.77 |
| Clonaz+Oxcar vs Carbam+Valpro | 0.08 | 0.00-0.98 |
| Clonaz+Oxcar vs Clonaz+Valpro | 0.04 | 0.00-0.98 |
| Clonaz+Oxcar vs Pheno+Pheny+Primid | 0.05 | 0.00-0.58 |
| Clonaz+Oxcar vs Pheno+Primid | 0.05 | 0.00-0.54 |
| Clonaz+Valpro vs Control | 14.74 | 1.07-79.94 |
| Ethos vs Control | 3.55 | 1.28-7.36 |
| Gabap vs Carbam+Clonaz | 0.10 | 0.03-0.41 |
| Gabap vs Carbam+Pheno | 0.37 | 0.14-0.83 |
| Gabap vs Carbam+Pheny | 0.25 | 0.11-0.59 |
| Gabap vs Carbam+Valpro | 0.15 | 0.07-0.34 |
| Gabap vs Clonaz+Valpro | 0.07 | 0.01-0.80 |
| Gabap vs Ethos | 0.29 | 0.12-0.95 |
| Gabap vs Pheno+Pheny | 0.46 | 0.20-0.86 |
| Gabap vs Pheno+Pheny+Primid | 0.09 | 0.03-0.23 |
| Gabap vs Pheno+Primid | 0.08 | 0.03-0.27 |
| Gabap vs Pheno+Valpro | 0.18 | 0.06-0.49 |
| Gabap vs Pheny+Primid | 0.19 | 0.07-0.48 |
| Gabap vs Pheny+Valpro | 0.21 | 0.06-0.66 |
| Gabap vs Valpro | 0.33 | 0.16-0.62 |
| Lamot vs Carbam | 0.70 | 0.54-0.87 |
| Lamot vs Carbam+Clonaz | 0.11 | 0.04-0.33 |
| Lamot vs Carbam+Pheno | 0.37 | 0.19-0.67 |
| Lamot vs Carbam+Pheno+Pheny | 0.26 | 0.10-0.93 |
| Lamot vs Carbam+Pheny | 0.25 | 0.15-0.46 |
| Lamot vs Carbam+Valpro | 0.15 | 0.09-0.28 |
| Lamot vs Clonaz+Valpro | 0.07 | 0.01-0.89 |
| Lamot vs Ethos | 0.30 | 0.14-0.73 |
| Lamot vs Pheno | 0.53 | 0.38-0.73 |
| Lamot vs Pheno+Pheny | 0.45 | 0.28-0.65 |
| Lamot vs Pheno+Pheny+Primid | 0.09 | 0.04-0.19 |
| Lamot vs Pheno+Primid | 0.08 | 0.04-0.20 |
| Lamot vs Pheno+Valpro | 0.19 | 0.08-0.41 |
| Lamot vs Pheny | 0.57 | 0.42-0.76 |
| Lamot vs Pheny+Primid | 0.19 | 0.09-0.41 |
| Lamot vs Pheny+Valpro | 0.20 | 0.08-0.56 |
| Lamot vs Valpro | 0.33 | 0.25-0.41 |
| Lamot+Levet vs Pheno+Pheny+Primid | 0.03 | 0.00-0.96 |
| Lamot+Levet vs Pheno+Primid | 0.03 | 0.00-0.92 |
| Lamot+Valpro vs Carbam+Clonaz | 0.12 | 0.02-0.81 |
| Lamot+Valpro vs Carbam+Valpro | 0.20 | 0.04-0.86 |
| Lamot+Valpro vs Pheno+Pheny+Primid | 0.12 | 0.02-0.55 |
| Lamot+Valpro vs Pheno+Primid | 0.11 | 0.02-0.53 |
| Levet vs Carbam | 0.53 | 0.31-0.82 |
| Levet vs Carbam+Clonaz | 0.08 | 0.02-0.27 |
| Levet vs Carbam+Pheno | 0.28 | 0.13-0.56 |
| Levet vs Carbam+Pheno+Pheny | 0.19 | 0.07-0.72 |
| Levet vs Carbam+Pheny | 0.19 | 0.09-0.39 |
| Levet vs Carbam+Valpro | 0.12 | 0.06-0.23 |
| Levet vs Clonaz+Valpro | 0.05 | 0.01-0.61 |
| Levet vs Ethos | 0.23 | 0.09-0.60 |
| Levet vs Pheno | 0.41 | 0.23-0.65 |
| Levet vs Pheno+Pheny | 0.34 | 0.18-0.56 |
| Levet vs Pheno+Pheny+Primid | 0.07 | 0.03-0.16 |
| Levet vs Pheno+Primid | 0.06 | 0.02-0.16 |
| Levet vs Pheno+Valpro | 0.14 | 0.05-0.34 |
| Levet vs Pheny | 0.43 | 0.25-0.68 |
| Levet vs Pheny+Primid | 0.14 | 0.06-0.36 |
| Levet vs Pheny+Valpro | 0.16 | 0.05-0.45 |
| Levet vs Topir | 0.38 | 0.19-0.68 |
| Levet vs Valpro | 0.25 | 0.14-0.38 |
| Oxcar vs Carbam+Clonaz | 0.15 | 0.05-0.53 |
| Oxcar vs Carbam+Pheny | 0.37 | 0.17-0.80 |
| Oxcar vs Carbam+Valpro | 0.22 | 0.11-0.47 |
| Oxcar vs Pheno+Pheny+Primid | 0.13 | 0.05-0.32 |
| Oxcar vs Pheno+Primid | 0.11 | 0.04-0.33 |
| Oxcar vs Pheno+Valpro | 0.26 | 0.10-0.68 |
| Oxcar vs Pheny+Primid | 0.29 | 0.12-0.69 |
| Oxcar vs Pheny+Valpro | 0.30 | 0.10-0.84 |
| Oxcar vs Valpro | 0.47 | 0.27-0.79 |
| Pheno vs Control | 1.87 | 1.34-2.73 |
| Pheno+Pheny vs Control | 2.28 | 1.49-3.57 |
| Pheno+Pheny+Primid vs Carbam | 7.48 | 3.65-17.49 |
| Pheno+Pheny+Primid vs Carbam+Pheno | 4.02 | 1.59-10.15 |
| Pheno+Pheny+Primid vs Carbam+Pheny | 2.81 | 1.21-6.64 |
| Pheno+Pheny+Primid vs Control | 11.30 | 4.88-25.16 |
| Pheno+Pheny+Primid vs Ethos | 3.21 | 1.20-10.03 |
| Pheno+Pheny+Primid vs Pheno | 5.80 | 2.75-13.75 |
| Pheno+Pheny+Primid vs Pheno+Pheny | 4.99 | 2.27-10.82 |
| Pheno+Pheny+Primid vs Pheny | 6.31 | 2.97-14.13 |
| Pheno+Pheny+Primid vs Primid | 8.48 | 3.48-25.81 |
| Pheno+Primid vs Carbam | 8.63 | 3.46-18.71 |
| Pheno+Primid vs Carbam+Pheno | 4.46 | 1.65-11.39 |
| Pheno+Primid vs Carbam+Pheny | 3.20 | 1.05-8.03 |
| Pheno+Primid vs Control | 13.03 | 4.72-28.63 |
| Pheno+Primid vs Ethos | 3.56 | 1.13-11.66 |
| Pheno+Primid vs Pheno | 6.47 | 2.52-14.44 |
| Pheno+Primid vs Pheno+Pheny | 5.60 | 2.04-12.42 |
| Pheno+Primid vs Pheny | 7.09 | 2.77-15.44 |
| Pheno+Primid vs Primid | 9.52 | 3.56-26.85 |
| Pheno+Valpro vs Carbam | 3.74 | 1.73-8.66 |
| Pheno+Valpro vs Clonaz | 4.93 | 1.77-12.18 |
| Pheno+Valpro vs Control | 5.62 | 2.38-12.45 |
| Pheno+Valpro vs Pheno | 2.88 | 1.28-7.09 |
| Pheno+Valpro vs Pheno+Pheny | 2.51 | 1.08-5.38 |
| Pheno+Valpro vs Pheny | 3.12 | 1.41-7.02 |
| Pheno+Valpro vs Primid | 4.17 | 1.58-13.02 |
| Pheny vs Control | 1.78 | 1.27-2.39 |
| Pheny+Primid vs Carbam | 3.62 | 1.70-7.28 |
| Pheny+Primid vs Control | 5.19 | 2.45-10.70 |
| Pheny+Primid vs Pheno | 2.76 | 1.28-5.48 |
| Pheny+Primid vs Pheno+Pheny | 2.27 | 1.08-4.62 |
| Pheny+Primid vs Pheny | 2.95 | 1.37-5.97 |
| Pheny+Primid vs Primid | 4.02 | 1.46-10.43 |
| Pheny+Valpro vs Carbam | 3.39 | 1.26-8.44 |
| Pheny+Valpro vs Clonaz | 4.09 | 1.42-12.28 |
| Pheny+Valpro vs Control | 4.84 | 1.77-12.26 |
| Pheny+Valpro vs Pheny | 2.72 | 1.02-6.99 |
| Pheny+Valpro vs Primid | 3.96 | 1.19-12.29 |
| Primid vs Carbam+Pheny | 0.33 | 0.14-0.71 |
| Primid+Valpro vs Pheno+Pheny+Primid | 0.12 | 0.00-0.90 |
| Primid+Valpro vs Pheno+Primid | 0.10 | 0.00-0.79 |
| Topir vs Carbam+Clonaz | 0.21 | 0.08-0.68 |
| Topir vs Carbam+Pheny | 0.51 | 0.26-1.00 |
| Topir vs Carbam+Valpro | 0.31 | 0.17-0.61 |
| Topir vs Control | 2.05 | 1.21-3.40 |
| Topir vs Lamot | 2.03 | 1.27-3.16 |
| Topir vs Pheno+Pheny+Primid | 0.19 | 0.07-0.41 |
| Topir vs Pheno+Primid | 0.17 | 0.07-0.44 |
| Topir vs Pheno+Valpro | 0.38 | 0.14-0.88 |
| Topir vs Pheny+Primid | 0.41 | 0.17-0.89 |
| Valpro vs Carbam | 2.12 | 1.78-2.55 |
| Valpro vs Carbam+Clonaz | 0.33 | 0.10-0.98 |
| Valpro vs Clonaz | 2.68 | 1.50-4.73 |
| Valpro vs Control | 3.10 | 2.26-4.14 |
| Valpro vs Pheno | 1.63 | 1.24-2.14 |
| Valpro vs Pheno+Pheny+Primid | 0.28 | 0.13-0.58 |
| Valpro vs Pheno+Primid | 0.25 | 0.11-0.62 |
| Valpro vs Pheny | 1.77 | 1.38-2.23 |
| Valpro vs Primid | 2.44 | 1.39-4.69 |
| Vigab vs Pheno+Pheny+Primid | 0.25 | 0.04-1.00 |
| *Common within-network between-study variance* | *0.03* | *0.00-0.14* |
| *Regression coefficient* | *0.02* | *-0.08-0.10* |
| **Major Congenital Malformations – Age** | | |
| Carbam vs Pheno+Pheny | 0.49 | 0.28-0.89 |
| Carbam+Gabap vs Pheno+Pheny+Primid | 0.02 | 0.00-0.87 |
| Carbam+Lamot vs Carbam+Valpro | 0.06 | 0.00-0.92 |
| Carbam+Lamot vs Pheno+Pheny+Primid | 0.03 | 0.00-0.63 |
| Carbam+Lamot vs Pheno+Primid | 0.03 | 0.00-0.77 |
| Carbam+Pheny+primid vs Pheno+Pheny+Primid | 0.15 | 0.01-0.87 |
| Carbam+Valpro vs Carbam | 5.35 | 2.57-11.30 |
| Carbam+Valpro vs Carbam+Pheno | 2.94 | 1.11-9.30 |
| Carbam+Valpro vs Carbam+Pheny | 3.22 | 1.24-9.43 |
| Carbam+Valpro vs Clonaz | 5.73 | 1.94-26.62 |
| Carbam+Valpro vs Control | 6.94 | 3.21-15.40 |
| Carbam+Valpro vs Pheno | 4.32 | 1.92-9.71 |
| Carbam+Valpro vs Pheno+Pheny | 2.58 | 1.16-6.19 |
| Carbam+Valpro vs Pheny | 5.05 | 2.45-11.14 |
| Carbam+Valpro vs Primid | 5.68 | 1.82-19.92 |
| Carbam+Valpro vs Valpro | 2.31 | 1.14-4.89 |
| Clobaz+Oxcar vs Carbam+Valpro | 0.04 | 0.00-0.70 |
| Clobaz+Oxcar vs Pheno+Pheny+Primid | 0.02 | 0.00-0.42 |
| Clobaz+Oxcar vs Pheno+Primid | 0.02 | 0.00-0.58 |
| Clobaz+Oxcar vs Pheno+Valpro | 0.04 | 0.00-0.98 |
| Clonaz vs Pheno+Pheny+Primid | 0.09 | 0.02-0.34 |
| Clonaz vs Pheno+Primid | 0.10 | 0.01-0.60 |
| Clonaz vs Pheny+Primid | 0.26 | 0.05-0.98 |
| Clonaz+Oxcar vs Carbam+Valpro | 0.05 | 0.00-0.75 |
| Clonaz+Oxcar vs Pheno+Pheny+Primid | 0.02 | 0.00-0.43 |
| Clonaz+Oxcar vs Pheno+Primid | 0.02 | 0.00-0.56 |
| Clonaz+Oxcar vs Pheno+Valpro | 0.05 | 0.00-0.96 |
| Gabap vs Carbam+Pheno | 0.21 | 0.03-0.91 |
| Gabap vs Carbam+Pheno+Pheny | 0.15 | 0.02-0.92 |
| Gabap vs Carbam+Pheny | 0.22 | 0.03-0.97 |
| Gabap vs Carbam+Valpro | 0.07 | 0.01-0.25 |
| Gabap vs Pheno | 0.29 | 0.05-0.95 |
| Gabap vs Pheno+Pheny | 0.17 | 0.03-0.65 |
| Gabap vs Pheno+Pheny+Primid | 0.04 | 0.01-0.17 |
| Gabap vs Pheno+Primid | 0.04 | 0.00-0.30 |
| Gabap vs Pheno+Valpro | 0.08 | 0.01-0.38 |
| Gabap vs Pheny+Primid | 0.10 | 0.01-0.50 |
| Gabap vs Valpro | 0.16 | 0.03-0.49 |
| Gabap+Pheny vs Pheno+Pheny+Primid | 0.02 | 0.00-0.79 |
| Lamot vs Carbam | 0.64 | 0.43-0.89 |
| Lamot vs Carbam+Pheno | 0.34 | 0.15-0.95 |
| Lamot vs Carbam+Pheny | 0.38 | 0.17-0.98 |
| Lamot vs Carbam+Valpro | 0.12 | 0.05-0.26 |
| Lamot vs Pheno | 0.51 | 0.31-0.84 |
| Lamot vs Pheno+Pheny | 0.31 | 0.17-0.61 |
| Lamot vs Pheno+Pheny+Primid | 0.07 | 0.03-0.18 |
| Lamot vs Pheno+Primid | 0.07 | 0.01-0.33 |
| Lamot vs Pheno+Valpro | 0.15 | 0.05-0.39 |
| Lamot vs Pheny | 0.61 | 0.38-0.94 |
| Lamot vs Pheny+Primid | 0.18 | 0.06-0.52 |
| Lamot vs Valpro | 0.28 | 0.19-0.38 |
| Lamot+Levet vs Pheno+Pheny+Primid | 0.04 | 0.00-0.86 |
| Lamot+Valpro vs Pheno+Pheny+Primid | 0.10 | 0.01-0.73 |
| Levet vs Carbam | 0.52 | 0.27-0.93 |
| Levet vs Carbam+Pheno | 0.29 | 0.11-0.86 |
| Levet vs Carbam+Pheny | 0.32 | 0.12-0.88 |
| Levet vs Carbam+Valpro | 0.10 | 0.04-0.25 |
| Levet vs Pheno | 0.42 | 0.20-0.84 |
| Levet vs Pheno+Pheny | 0.25 | 0.11-0.56 |
| Levet vs Pheno+Pheny+Primid | 0.05 | 0.02-0.16 |
| Levet vs Pheno+Primid | 0.06 | 0.01-0.30 |
| Levet vs Pheno+Valpro | 0.12 | 0.04-0.36 |
| Levet vs Pheny+Primid | 0.15 | 0.05-0.48 |
| Levet vs Topir | 0.41 | 0.18-0.87 |
| Levet vs Valpro | 0.23 | 0.12-0.40 |
| Oxcar vs Carbam+Valpro | 0.09 | 0.03-0.29 |
| Oxcar vs Pheno+Pheny | 0.23 | 0.08-0.65 |
| Oxcar vs Pheno+Pheny+Primid | 0.05 | 0.01-0.18 |
| Oxcar vs Pheno+Primid | 0.05 | 0.01-0.31 |
| Oxcar vs Pheno+Valpro | 0.11 | 0.03-0.39 |
| Oxcar vs Pheny+Primid | 0.14 | 0.03-0.56 |
| Oxcar vs Valpro | 0.21 | 0.07-0.53 |
| Pheno+Pheny vs Control | 2.71 | 1.31-5.26 |
| Pheno+Pheny vs Pheny | 1.97 | 1.08-3.59 |
| Pheno+Pheny+Primid vs Carbam | 9.71 | 3.54-24.04 |
| Pheno+Pheny+Primid vs Carbam+Pheno | 5.41 | 1.67-18.27 |
| Pheno+Pheny+Primid vs Carbam+Pheno+Pheny | 3.96 | 1.07-21.70 |
| Pheno+Pheny+Primid vs Carbam+Pheny | 5.98 | 1.94-18.64 |
| Pheno+Pheny+Primid vs Control | 12.73 | 4.49-33.99 |
| Pheno+Pheny+Primid vs Ethos | 11.31 | 1.40-393.00 |
| Pheno+Pheny+Primid vs Pheno | 7.80 | 2.78-20.95 |
| Pheno+Pheny+Primid vs Pheno+Pheny | 4.68 | 1.84-12.06 |
| Pheno+Pheny+Primid vs Pheny | 9.33 | 3.52-23.00 |
| Pheno+Pheny+Primid vs Primid | 10.24 | 2.77-40.09 |
| Pheno+Primid vs Carbam | 9.42 | 1.91-44.29 |
| Pheno+Primid vs Carbam+Pheny | 5.80 | 1.06-28.63 |
| Pheno+Primid vs Control | 12.48 | 2.44-61.94 |
| Pheno+Primid vs Ethos | 11.41 | 1.04-440.40 |
| Pheno+Primid vs Pheno | 7.64 | 1.51-37.71 |
| Pheno+Primid vs Pheny | 9.02 | 1.88-42.67 |
| Pheno+Primid vs Primid | 9.96 | 1.63-64.23 |
| Pheno+Valpro vs Carbam | 4.34 | 1.72-11.55 |
| Pheno+Valpro vs Clonaz | 4.84 | 1.28-24.80 |
| Pheno+Valpro vs Control | 5.69 | 2.13-15.85 |
| Pheno+Valpro vs Pheno | 3.47 | 1.27-9.77 |
| Pheno+Valpro vs Pheny | 4.26 | 1.61-11.13 |
| Pheno+Valpro vs Primid | 4.62 | 1.28-19.75 |
| Pheny+Primid vs Carbam | 3.56 | 1.25-9.62 |
| Pheny+Primid vs Control | 4.65 | 1.56-13.08 |
| Pheny+Primid vs Pheny | 3.38 | 1.21-9.21 |
| Pheny+Valpro vs Pheno+Pheny+Primid | 0.14 | 0.03-0.58 |
| Pheny+Valpro vs Pheno+Primid | 0.14 | 0.02-1.00 |
| Topir vs Carbam+Valpro | 0.24 | 0.09-0.59 |
| Topir vs Lamot | 2.04 | 1.11-3.72 |
| Topir vs Pheno+Pheny+Primid | 0.14 | 0.05-0.39 |
| Topir vs Pheno+Primid | 0.14 | 0.03-0.72 |
| Topir vs Pheno+Valpro | 0.29 | 0.09-0.88 |
| Topir vs Valpro | 0.56 | 0.31-0.98 |
| Valpro vs Carbam | 2.31 | 1.77-2.99 |
| Valpro vs Control | 3.03 | 2.05-4.58 |
| Valpro vs Pheno | 1.86 | 1.24-2.87 |
| Valpro vs Pheno+Pheny+Primid | 0.24 | 0.09-0.62 |
| Valpro vs Pheny | 2.22 | 1.52-3.36 |
| *Common within-network between-study variance* | *0.03* | *0.00-0.16* |
| *Regression coefficient* | *-0.01* | *-0.16-0.14* |

### Statistically Significant Schmitz Results for Major Congenital Malformations

| **Treatment Comparison** | **Median Odds Ratio** | **Low CrI-High CrI** |
| --- | --- | --- |
| **Major Congenital Malformations – Overall** | | |
| Carbam+Clonaz vs Carbam | 6.58 | 1.34-42.79 |
| Carbam+Clonaz vs Clonaz | 7.71 | 2.36-23.90 |
| Carbam+Clonaz vs Control | 8.67 | 2.97-22.46 |
| Carbam+Clonaz vs Pheno | 4.67 | 1.64-12.31 |
| Carbam+Clonaz vs Pheno+Pheny | 3.92 | 1.34-10.11 |
| Carbam+Clonaz vs Pheny | 5.09 | 1.78-12.61 |
| Carbam+Clonaz vs Primid | 7.43 | 2.15-20.86 |
| Carbam+Lamot vs Carbam+Clonaz | 0.04 | 0.00-0.87 |
| Carbam+Lamot vs Pheno+Pheny+Primid | 0.03 | 0.00-0.71 |
| Carbam+Lamot vs Pheno+Primid | 0.02 | 0.00-0.67 |
| Carbam+Pheno vs Control | 2.69 | 1.36-5.73 |
| Carbam+Pheny vs Control | 4.27 | 1.33-22.88 |
| Carbam+Valpro vs Carbam | 4.65 | 1.14-24.83 |
| Carbam+Valpro vs Clonaz | 5.30 | 2.44-11.60 |
| Carbam+Valpro vs Control | 6.05 | 3.32-10.59 |
| Carbam+Valpro vs Pheno | 3.26 | 1.82-5.56 |
| Carbam+Valpro vs Pheno+Pheny | 2.72 | 1.47-4.89 |
| Carbam+Valpro vs Pheny | 3.56 | 1.89-6.01 |
| Carbam+Valpro vs Primid | 5.09 | 2.20-11.50 |
| Carbam+Valpro vs Valpro | 2.05 | 1.12-3.46 |
| Clobaz+Oxcar vs Pheno+Pheny+Primid | 0.04 | 0.00-0.70 |
| Clobaz+Oxcar vs Pheno+Primid | 0.03 | 0.00-0.63 |
| Clonaz vs Carbam+Pheny | 0.27 | 0.05-0.95 |
| Clonaz vs Pheno+Pheny+Primid | 0.11 | 0.04-0.27 |
| Clonaz vs Pheno+Primid | 0.09 | 0.03-0.26 |
| Clonaz vs Pheny+Primid | 0.24 | 0.09-0.68 |
| Clonaz+Oxcar vs Carbam+Clonaz | 0.04 | 0.00-0.92 |
| Clonaz+Oxcar vs Pheno+Pheny+Primid | 0.03 | 0.00-0.66 |
| Clonaz+Oxcar vs Pheno+Primid | 0.03 | 0.00-0.64 |
| Clonaz+Valpro vs Control | 11.96 | 1.15-75.96 |
| Ethos vs Control | 3.29 | 1.24-7.11 |
| Gabap vs Carbam+Clonaz | 0.11 | 0.03-0.39 |
| Gabap vs Carbam+Pheno | 0.37 | 0.14-0.86 |
| Gabap vs Carbam+Pheny | 0.23 | 0.04-0.84 |
| Gabap vs Carbam+Valpro | 0.17 | 0.06-0.39 |
| Gabap vs Clonaz+Valpro | 0.08 | 0.01-0.91 |
| Gabap vs Ethos | 0.30 | 0.11-0.97 |
| Gabap vs Pheno+Pheny | 0.45 | 0.18-0.91 |
| Gabap vs Pheno+Pheny+Primid | 0.09 | 0.03-0.24 |
| Gabap vs Pheno+Primid | 0.08 | 0.03-0.24 |
| Gabap vs Pheno+Valpro | 0.18 | 0.06-0.51 |
| Gabap vs Pheny+Primid | 0.21 | 0.07-0.61 |
| Gabap vs Pheny+Valpro | 0.19 | 0.06-0.64 |
| Gabap vs Valpro | 0.33 | 0.16-0.62 |
| Lamot vs Carbam+Clonaz | 0.11 | 0.04-0.32 |
| Lamot vs Carbam+Pheno | 0.36 | 0.17-0.75 |
| Lamot vs Carbam+Pheny | 0.23 | 0.04-0.73 |
| Lamot vs Carbam+Valpro | 0.16 | 0.09-0.30 |
| Lamot vs Clonaz+Valpro | 0.08 | 0.01-0.85 |
| Lamot vs Ethos | 0.30 | 0.14-0.80 |
| Lamot vs Pheno | 0.53 | 0.37-0.72 |
| Lamot vs Pheno+Pheny | 0.44 | 0.28-0.65 |
| Lamot vs Pheno+Pheny+Primid | 0.09 | 0.04-0.19 |
| Lamot vs Pheno+Primid | 0.08 | 0.03-0.20 |
| Lamot vs Pheno+Valpro | 0.18 | 0.08-0.42 |
| Lamot vs Pheny | 0.57 | 0.41-0.75 |
| Lamot vs Pheny+Primid | 0.20 | 0.09-0.56 |
| Lamot vs Pheny+Valpro | 0.19 | 0.07-0.49 |
| Lamot vs Valpro | 0.33 | 0.25-0.40 |
| Lamot+Levet vs Pheno+Primid | 0.06 | 0.00-0.98 |
| Lamot+Valpro vs Carbam+Clonaz | 0.14 | 0.02-0.86 |
| Lamot+Valpro vs Carbam+Valpro | 0.19 | 0.03-0.98 |
| Lamot+Valpro vs Pheno+Pheny+Primid | 0.11 | 0.01-0.67 |
| Lamot+Valpro vs Pheno+Primid | 0.10 | 0.01-0.67 |
| Levet vs Carbam+Clonaz | 0.08 | 0.03-0.25 |
| Levet vs Carbam+Pheno | 0.26 | 0.11-0.65 |
| Levet vs Carbam+Pheno+Pheny | 0.21 | 0.06-0.85 |
| Levet vs Carbam+Pheny | 0.17 | 0.03-0.57 |
| Levet vs Carbam+Valpro | 0.12 | 0.06-0.27 |
| Levet vs Clonaz+Valpro | 0.06 | 0.01-0.65 |
| Levet vs Ethos | 0.23 | 0.09-0.62 |
| Levet vs Pheno | 0.39 | 0.21-0.65 |
| Levet vs Pheno+Pheny | 0.33 | 0.17-0.58 |
| Levet vs Pheno+Pheny+Primid | 0.07 | 0.03-0.15 |
| Levet vs Pheno+Primid | 0.06 | 0.02-0.16 |
| Levet vs Pheno+Valpro | 0.13 | 0.05-0.33 |
| Levet vs Pheny | 0.43 | 0.24-0.69 |
| Levet vs Pheny+Primid | 0.15 | 0.06-0.46 |
| Levet vs Pheny+Valpro | 0.14 | 0.05-0.43 |
| Levet vs Topir | 0.38 | 0.19-0.68 |
| Levet vs Valpro | 0.25 | 0.14-0.38 |
| Oxcar vs Carbam+Clonaz | 0.16 | 0.05-0.50 |
| Oxcar vs Carbam+Valpro | 0.23 | 0.11-0.46 |
| Oxcar vs Pheno+Pheny+Primid | 0.13 | 0.05-0.30 |
| Oxcar vs Pheno+Primid | 0.11 | 0.04-0.31 |
| Oxcar vs Pheno+Valpro | 0.26 | 0.10-0.66 |
| Oxcar vs Pheny+Primid | 0.29 | 0.11-0.76 |
| Oxcar vs Pheny+Valpro | 0.26 | 0.09-0.78 |
| Oxcar vs Valpro | 0.45 | 0.26-0.76 |
| Pheno vs Control | 1.86 | 1.39-2.55 |
| Pheno+Pheny vs Control | 2.23 | 1.51-3.32 |
| Pheno+Pheny+Primid vs Carbam | 8.28 | 1.95-46.90 |
| Pheno+Pheny+Primid vs Carbam+Pheno | 3.98 | 1.44-10.70 |
| Pheno+Pheny+Primid vs Control | 10.73 | 5.07-23.39 |
| Pheno+Pheny+Primid vs Ethos | 3.32 | 1.17-11.27 |
| Pheno+Pheny+Primid vs Pheno | 5.73 | 2.74-12.23 |
| Pheno+Pheny+Primid vs Pheno+Pheny | 4.76 | 2.29-10.28 |
| Pheno+Pheny+Primid vs Pheny | 6.20 | 2.96-13.33 |
| Pheno+Pheny+Primid vs Primid | 9.12 | 3.53-23.37 |
| Pheno+Primid vs Carbam | 9.38 | 2.02-52.40 |
| Pheno+Primid vs Carbam+Pheno | 4.35 | 1.55-13.02 |
| Pheno+Primid vs Control | 12.01 | 4.94-29.31 |
| Pheno+Primid vs Ethos | 3.77 | 1.21-12.95 |
| Pheno+Primid vs Pheno | 6.42 | 2.60-15.68 |
| Pheno+Primid vs Pheno+Pheny | 5.34 | 2.14-13.50 |
| Pheno+Primid vs Pheny | 6.99 | 2.86-16.83 |
| Pheno+Primid vs Primid | 10.12 | 3.53-29.86 |
| Pheno+Valpro vs Clonaz | 4.85 | 1.70-12.95 |
| Pheno+Valpro vs Control | 5.45 | 2.36-11.85 |
| Pheno+Valpro vs Pheno | 2.90 | 1.28-6.33 |
| Pheno+Valpro vs Pheno+Pheny | 2.43 | 1.04-5.50 |
| Pheno+Valpro vs Pheny | 3.16 | 1.34-6.80 |
| Pheno+Valpro vs Primid | 4.62 | 1.62-12.02 |
| Pheny vs Control | 1.72 | 1.34-2.26 |
| Pheny+Primid vs Control | 4.84 | 1.83-10.16 |
| Pheny+Primid vs Pheno | 2.59 | 1.02-5.47 |
| Pheny+Primid vs Pheny | 2.82 | 1.09-5.84 |
| Pheny+Primid vs Primid | 4.07 | 1.40-10.41 |
| Pheny+Valpro vs Clonaz | 4.44 | 1.52-14.41 |
| Pheny+Valpro vs Control | 5.17 | 1.98-13.46 |
| Pheny+Valpro vs Pheno | 2.80 | 1.03-7.11 |
| Pheny+Valpro vs Pheny | 3.03 | 1.14-7.44 |
| Pheny+Valpro vs Primid | 4.34 | 1.38-12.81 |
| Primid vs Carbam+Pheny | 0.28 | 0.05-0.99 |
| Primid+Valpro vs Pheno+Pheny+Primid | 0.12 | 0.01-0.83 |
| Primid+Valpro vs Pheno+Primid | 0.11 | 0.00-0.92 |
| Topir vs Carbam+Clonaz | 0.22 | 0.08-0.67 |
| Topir vs Carbam+Valpro | 0.32 | 0.16-0.66 |
| Topir vs Control | 1.95 | 1.22-3.04 |
| Topir vs Lamot | 2.00 | 1.26-3.11 |
| Topir vs Pheno+Pheny+Primid | 0.18 | 0.07-0.42 |
| Topir vs Pheno+Primid | 0.16 | 0.06-0.42 |
| Topir vs Pheno+Valpro | 0.36 | 0.14-0.89 |
| Topir vs Valpro | 0.65 | 0.42-0.99 |
| Valpro vs Carbam+Clonaz | 0.34 | 0.14-0.97 |
| Valpro vs Clonaz | 2.62 | 1.48-4.89 |
| Valpro vs Control | 2.99 | 2.40-3.71 |
| Valpro vs Pheno | 1.61 | 1.19-2.12 |
| Valpro vs Pheno+Pheny+Primid | 0.28 | 0.13-0.58 |
| Valpro vs Pheno+Primid | 0.25 | 0.10-0.60 |
| Valpro vs Pheny | 1.73 | 1.34-2.22 |
| Valpro vs Primid | 2.46 | 1.36-4.75 |
| *Common within-network between-study variance* | *0.30* | *0.00-3.95* |
| **Major Congenital Malformations – Observational Studies Only (Sensitivity Analysis)** | | |
| Carbam vs Control | 1.40 | 1.13-1.75 |
| Carbam vs Pheno | 0.75 | 0.57-0.99 |
| Carbam vs Pheno+Pheny | 0.63 | 0.43-0.91 |
| Carbam+Clonaz vs Carbam | 6.21 | 2.20-15.85 |
| Carbam+Clonaz vs Clonaz | 7.71 | 2.36-23.90 |
| Carbam+Clonaz vs Control | 8.67 | 2.97-22.46 |
| Carbam+Clonaz vs Pheno | 4.67 | 1.64-12.31 |
| Carbam+Clonaz vs Pheno+Pheny | 3.92 | 1.34-10.11 |
| Carbam+Clonaz vs Pheny | 5.09 | 1.78-12.61 |
| Carbam+Clonaz vs Primid | 7.43 | 2.15-20.86 |
| Carbam+Lamot vs Carbam+Clonaz | 0.04 | 0.00-0.87 |
| Carbam+Lamot vs Pheno+Pheny+Primid | 0.03 | 0.00-0.71 |
| Carbam+Lamot vs Pheno+Primid | 0.02 | 0.00-0.67 |
| Carbam+Pheno vs Control | 2.69 | 1.36-5.73 |
| Carbam+Pheny vs Carbam | 2.69 | 1.53-4.71 |
| Carbam+Pheny vs Control | 3.75 | 2.06-6.76 |
| Carbam+Pheny vs Pheno | 2.01 | 1.10-3.54 |
| Carbam+Pheny vs Pheny | 2.17 | 1.21-3.83 |
| Carbam+Valpro vs Carbam | 4.34 | 2.41-7.43 |
| Carbam+Valpro vs Clonaz | 5.30 | 2.44-11.60 |
| Carbam+Valpro vs Control | 6.05 | 3.32-10.59 |
| Carbam+Valpro vs Pheno | 3.26 | 1.82-5.56 |
| Carbam+Valpro vs Pheno+Pheny | 2.72 | 1.47-4.89 |
| Carbam+Valpro vs Pheny | 3.56 | 1.89-6.01 |
| Carbam+Valpro vs Primid | 5.09 | 2.20-11.50 |
| Carbam+Valpro vs Valpro | 2.05 | 1.12-3.46 |
| Clobaz+Oxcar vs Pheno+Pheny+Primid | 0.04 | 0.00-0.70 |
| Clobaz+Oxcar vs Pheno+Primid | 0.03 | 0.00-0.63 |
| Clonaz vs Carbam+Pheny | 0.30 | 0.13-0.66 |
| Clonaz vs Pheno+Pheny+Primid | 0.11 | 0.04-0.27 |
| Clonaz vs Pheno+Primid | 0.09 | 0.03-0.26 |
| Clonaz vs Pheny+Primid | 0.24 | 0.09-0.68 |
| Clonaz+Oxcar vs Carbam+Clonaz | 0.04 | 0.00-0.92 |
| Clonaz+Oxcar vs Pheno+Pheny+Primid | 0.03 | 0.00-0.66 |
| Clonaz+Oxcar vs Pheno+Primid | 0.03 | 0.00-0.64 |
| Clonaz+Valpro vs Control | 11.96 | 1.15-75.96 |
| Ethos vs Control | 3.29 | 1.24-7.11 |
| Gabap vs Carbam+Clonaz | 0.11 | 0.03-0.39 |
| Gabap vs Carbam+Pheno | 0.37 | 0.14-0.86 |
| Gabap vs Carbam+Pheny | 0.26 | 0.10-0.62 |
| Gabap vs Carbam+Valpro | 0.17 | 0.06-0.39 |
| Gabap vs Clonaz+Valpro | 0.08 | 0.01-0.91 |
| Gabap vs Ethos | 0.30 | 0.11-0.97 |
| Gabap vs Pheno+Pheny | 0.45 | 0.18-0.91 |
| Gabap vs Pheno+Pheny+Primid | 0.09 | 0.03-0.24 |
| Gabap vs Pheno+Primid | 0.08 | 0.03-0.24 |
| Gabap vs Pheno+Valpro | 0.18 | 0.06-0.51 |
| Gabap vs Pheny+Primid | 0.21 | 0.07-0.61 |
| Gabap vs Pheny+Valpro | 0.19 | 0.06-0.64 |
| Gabap vs Valpro | 0.33 | 0.16-0.62 |
| Lamot vs Carbam | 0.70 | 0.53-0.87 |
| Lamot vs Carbam+Clonaz | 0.11 | 0.04-0.32 |
| Lamot vs Carbam+Pheno | 0.36 | 0.17-0.75 |
| Lamot vs Carbam+Pheny | 0.26 | 0.14-0.47 |
| Lamot vs Carbam+Valpro | 0.16 | 0.09-0.30 |
| Lamot vs Clonaz+Valpro | 0.08 | 0.01-0.85 |
| Lamot vs Ethos | 0.30 | 0.14-0.80 |
| Lamot vs Pheno | 0.53 | 0.37-0.72 |
| Lamot vs Pheno+Pheny | 0.44 | 0.28-0.65 |
| Lamot vs Pheno+Pheny+Primid | 0.09 | 0.04-0.19 |
| Lamot vs Pheno+Primid | 0.08 | 0.03-0.20 |
| Lamot vs Pheno+Valpro | 0.18 | 0.08-0.42 |
| Lamot vs Pheny | 0.57 | 0.41-0.75 |
| Lamot vs Pheny+Primid | 0.20 | 0.09-0.56 |
| Lamot vs Pheny+Valpro | 0.19 | 0.07-0.49 |
| Lamot vs Valpro | 0.33 | 0.25-0.40 |
| Lamot+Levet vs Pheno+Primid | 0.06 | 0.00-0.98 |
| Lamot+Valpro vs Carbam+Clonaz | 0.14 | 0.02-0.86 |
| Lamot+Valpro vs Carbam+Valpro | 0.19 | 0.03-0.98 |
| Lamot+Valpro vs Pheno+Pheny+Primid | 0.11 | 0.01-0.67 |
| Lamot+Valpro vs Pheno+Primid | 0.10 | 0.01-0.67 |
| Levet vs Carbam | 0.53 | 0.30-0.82 |
| Levet vs Carbam+Clonaz | 0.08 | 0.03-0.25 |
| Levet vs Carbam+Pheno | 0.26 | 0.11-0.65 |
| Levet vs Carbam+Pheno+Pheny | 0.21 | 0.06-0.85 |
| Levet vs Carbam+Pheny | 0.19 | 0.09-0.40 |
| Levet vs Carbam+Valpro | 0.12 | 0.06-0.27 |
| Levet vs Clonaz+Valpro | 0.06 | 0.01-0.65 |
| Levet vs Ethos | 0.23 | 0.09-0.62 |
| Levet vs Pheno | 0.39 | 0.21-0.65 |
| Levet vs Pheno+Pheny | 0.33 | 0.17-0.58 |
| Levet vs Pheno+Pheny+Primid | 0.07 | 0.03-0.15 |
| Levet vs Pheno+Primid | 0.06 | 0.02-0.16 |
| Levet vs Pheno+Valpro | 0.13 | 0.05-0.33 |
| Levet vs Pheny | 0.43 | 0.24-0.69 |
| Levet vs Pheny+Primid | 0.15 | 0.06-0.46 |
| Levet vs Pheny+Valpro | 0.14 | 0.05-0.43 |
| Levet vs Topir | 0.38 | 0.19-0.68 |
| Levet vs Valpro | 0.25 | 0.14-0.38 |
| Oxcar vs Carbam+Clonaz | 0.16 | 0.05-0.50 |
| Oxcar vs Carbam+Pheny | 0.36 | 0.17-0.76 |
| Oxcar vs Carbam+Valpro | 0.23 | 0.11-0.46 |
| Oxcar vs Pheno+Pheny+Primid | 0.13 | 0.05-0.30 |
| Oxcar vs Pheno+Primid | 0.11 | 0.04-0.31 |
| Oxcar vs Pheno+Valpro | 0.26 | 0.10-0.66 |
| Oxcar vs Pheny+Primid | 0.29 | 0.11-0.76 |
| Oxcar vs Pheny+Valpro | 0.26 | 0.09-0.78 |
| Oxcar vs Valpro | 0.45 | 0.26-0.76 |
| Pheno vs Control | 1.86 | 1.39-2.55 |
| Pheno+Pheny vs Control | 2.23 | 1.51-3.32 |
| Pheno+Pheny+Primid vs Carbam | 7.66 | 3.72-15.96 |
| Pheno+Pheny+Primid vs Carbam+Pheno | 3.98 | 1.44-10.70 |
| Pheno+Pheny+Primid vs Carbam+Pheny | 2.83 | 1.22-7.01 |
| Pheno+Pheny+Primid vs Control | 10.73 | 5.07-23.39 |
| Pheno+Pheny+Primid vs Ethos | 3.32 | 1.17-11.27 |
| Pheno+Pheny+Primid vs Pheno | 5.73 | 2.74-12.23 |
| Pheno+Pheny+Primid vs Pheno+Pheny | 4.76 | 2.29-10.28 |
| Pheno+Pheny+Primid vs Pheny | 6.20 | 2.96-13.33 |
| Pheno+Pheny+Primid vs Primid | 9.12 | 3.53-23.37 |
| Pheno+Primid vs Carbam | 8.60 | 3.63-20.56 |
| Pheno+Primid vs Carbam+Pheno | 4.35 | 1.55-13.02 |
| Pheno+Primid vs Carbam+Pheny | 3.20 | 1.16-8.75 |
| Pheno+Primid vs Control | 12.01 | 4.94-29.31 |
| Pheno+Primid vs Ethos | 3.77 | 1.21-12.95 |
| Pheno+Primid vs Pheno | 6.42 | 2.60-15.68 |
| Pheno+Primid vs Pheno+Pheny | 5.34 | 2.14-13.50 |
| Pheno+Primid vs Pheny | 6.99 | 2.86-16.83 |
| Pheno+Primid vs Primid | 10.12 | 3.53-29.86 |
| Pheno+Valpro vs Carbam | 3.84 | 1.67-8.67 |
| Pheno+Valpro vs Clonaz | 4.85 | 1.70-12.95 |
| Pheno+Valpro vs Control | 5.45 | 2.36-11.85 |
| Pheno+Valpro vs Pheno | 2.90 | 1.28-6.33 |
| Pheno+Valpro vs Pheno+Pheny | 2.43 | 1.04-5.50 |
| Pheno+Valpro vs Pheny | 3.16 | 1.34-6.80 |
| Pheno+Valpro vs Primid | 4.62 | 1.62-12.02 |
| Pheny vs Control | 1.72 | 1.34-2.26 |
| Pheny+Primid vs Carbam | 3.47 | 1.31-7.26 |
| Pheny+Primid vs Control | 4.84 | 1.83-10.16 |
| Pheny+Primid vs Pheno | 2.59 | 1.02-5.47 |
| Pheny+Primid vs Pheny | 2.82 | 1.09-5.84 |
| Pheny+Primid vs Primid | 4.07 | 1.40-10.41 |
| Pheny+Valpro vs Carbam | 3.72 | 1.41-9.32 |
| Pheny+Valpro vs Clonaz | 4.44 | 1.52-14.41 |
| Pheny+Valpro vs Control | 5.17 | 1.98-13.46 |
| Pheny+Valpro vs Pheno | 2.80 | 1.03-7.11 |
| Pheny+Valpro vs Pheny | 3.03 | 1.14-7.44 |
| Pheny+Valpro vs Primid | 4.34 | 1.38-12.81 |
| Primid vs Carbam+Pheny | 0.32 | 0.14-0.71 |
| Primid+Valpro vs Pheno+Pheny+Primid | 0.12 | 0.01-0.83 |
| Primid+Valpro vs Pheno+Primid | 0.11 | 0.00-0.92 |
| Topir vs Carbam+Clonaz | 0.22 | 0.08-0.67 |
| Topir vs Carbam+Valpro | 0.32 | 0.16-0.66 |
| Topir vs Control | 1.95 | 1.22-3.04 |
| Topir vs Lamot | 2.00 | 1.26-3.11 |
| Topir vs Pheno+Pheny+Primid | 0.18 | 0.07-0.42 |
| Topir vs Pheno+Primid | 0.16 | 0.06-0.42 |
| Topir vs Pheno+Valpro | 0.36 | 0.14-0.89 |
| Topir vs Valpro | 0.65 | 0.42-0.99 |
| Valpro vs Carbam | 2.13 | 1.77-2.58 |
| Valpro vs Carbam+Clonaz | 0.34 | 0.14-0.97 |
| Valpro vs Clonaz | 2.62 | 1.48-4.89 |
| Valpro vs Control | 2.99 | 2.40-3.71 |
| Valpro vs Pheno | 1.61 | 1.19-2.12 |
| Valpro vs Pheno+Pheny+Primid | 0.28 | 0.13-0.58 |
| Valpro vs Pheno+Primid | 0.25 | 0.10-0.60 |
| Valpro vs Pheny | 1.73 | 1.34-2.22 |
| Valpro vs Primid | 2.46 | 1.36-4.75 |
| *Common within-network between-study variance* | *0.03* | *0.00-0.13* |
| **Major Congenital Malformations – Randomised Controlled Trials Only (Sensitivity Analysis)** | | |
| Carbam+Pheny vs Control | 4.23 | 1.33-22.88 |
| *Common within-network between-study variance* | NA | NA |
| **Major Congenital Malformations SA Overlap^a^ – Overall** | | |
| Carbam+Clonaz vs Carbam | 6.34 | 1.29-36.73 |
| Carbam+Clonaz vs Clonaz | 6.96 | 2.02-19.20 |
| Carbam+Clonaz vs Control | 8.01 | 2.81-19.30 |
| Carbam+Clonaz vs Pheno | 4.64 | 1.44-10.49 |
| Carbam+Clonaz vs Pheno+Pheny | 3.85 | 1.20-9.23 |
| Carbam+Clonaz vs Pheny | 5.04 | 1.62-11.51 |
| Carbam+Clonaz vs Primid | 6.42 | 1.96-17.54 |
| Carbam+Lamot vs Pheno+Pheny+Primid | 0.05 | 0.00-0.70 |
| Carbam+Lamot vs Pheno+Primid | 0.05 | 0.00-0.63 |
| Carbam+Pheno vs Control | 2.78 | 1.43-5.27 |
| Carbam+Pheny vs Control | 4.07 | 1.20-21.71 |
| Carbam+Pheny+primid vs Pheno+Pheny+Primid | 0.20 | 0.02-0.96 |
| Carbam+Pheny+primid vs Pheno+Primid | 0.16 | 0.02-0.98 |
| Carbam+Primid vs Pheno+Primid | 0.14 | 0.00-0.96 |
| Carbam+Valpro vs Carbam | 4.87 | 1.29-27.09 |
| Carbam+Valpro vs Carbam+Pheno | 2.31 | 1.06-5.23 |
| Carbam+Valpro vs Clonaz | 5.30 | 2.46-12.02 |
| Carbam+Valpro vs Control | 6.29 | 3.71-10.71 |
| Carbam+Valpro vs Pheno | 3.51 | 1.93-5.57 |
| Carbam+Valpro vs Pheno+Pheny | 2.92 | 1.55-5.13 |
| Carbam+Valpro vs Pheny | 3.89 | 2.24-6.32 |
| Carbam+Valpro vs Primid | 4.96 | 2.30-10.17 |
| Carbam+Valpro vs Valpro | 2.25 | 1.28-3.47 |
| Clobaz vs Pheno+Pheny+Primid | 0.22 | 0.06-0.89 |
| Clobaz vs Pheno+Primid | 0.21 | 0.05-0.87 |
| Clobaz+Oxcar vs Carbam+Clonaz | 0.05 | 0.00-0.88 |
| Clobaz+Oxcar vs Pheno+Pheny+Primid | 0.04 | 0.00-0.59 |
| Clobaz+Oxcar vs Pheno+Primid | 0.04 | 0.00-0.59 |
| Clonaz vs Carbam+Pheno | 0.43 | 0.17-0.97 |
| Clonaz vs Pheno+Pheny+Primid | 0.12 | 0.04-0.28 |
| Clonaz vs Pheno+Primid | 0.10 | 0.04-0.28 |
| Clonaz vs Pheny+Primid | 0.24 | 0.09-0.59 |
| Clonaz+Oxcar vs Carbam+Clonaz | 0.06 | 0.00-0.86 |
| Clonaz+Oxcar vs Pheno+Pheny+Primid | 0.05 | 0.00-0.67 |
| Clonaz+Oxcar vs Pheno+Primid | 0.05 | 0.00-0.58 |
| Ethos vs Control | 3.21 | 1.37-7.28 |
| Gabap vs Carbam+Clonaz | 0.13 | 0.04-0.42 |
| Gabap vs Carbam+Pheno | 0.34 | 0.15-0.89 |
| Gabap vs Carbam+Pheny | 0.23 | 0.04-0.93 |
| Gabap vs Carbam+Valpro | 0.16 | 0.06-0.35 |
| Gabap vs Ethos | 0.30 | 0.11-0.89 |
| Gabap vs Pheno+Pheny | 0.45 | 0.20-0.91 |
| Gabap vs Pheno+Pheny+Primid | 0.10 | 0.03-0.24 |
| Gabap vs Pheno+Primid | 0.08 | 0.03-0.25 |
| Gabap vs Pheno+Valpro | 0.19 | 0.06-0.51 |
| Gabap vs Pheny+Primid | 0.20 | 0.07-0.50 |
| Gabap vs Pheny+Valpro | 0.19 | 0.06-0.63 |
| Gabap vs Valpro | 0.34 | 0.16-0.64 |
| Lamot vs Carbam+Clonaz | 0.12 | 0.05-0.34 |
| Lamot vs Carbam+Pheno | 0.34 | 0.18-0.67 |
| Lamot vs Carbam+Pheny | 0.23 | 0.04-0.77 |
| Lamot vs Carbam+Valpro | 0.15 | 0.09-0.25 |
| Lamot vs Ethos | 0.30 | 0.13-0.69 |
| Lamot vs Pheno | 0.50 | 0.37-0.66 |
| Lamot vs Pheno+Pheny | 0.42 | 0.29-0.62 |
| Lamot vs Pheno+Pheny+Primid | 0.09 | 0.04-0.18 |
| Lamot vs Pheno+Primid | 0.08 | 0.03-0.21 |
| Lamot vs Pheno+Valpro | 0.18 | 0.07-0.40 |
| Lamot vs Pheny | 0.57 | 0.42-0.74 |
| Lamot vs Pheny+Primid | 0.19 | 0.09-0.38 |
| Lamot vs Pheny+Valpro | 0.19 | 0.08-0.49 |
| Lamot vs Valpro | 0.32 | 0.25-0.40 |
| Lamot+Levet vs Pheno+Primid | 0.05 | 0.00-0.95 |
| Lamot+Valpro vs Carbam+Valpro | 0.48 | 0.23-0.94 |
| Lamot+Valpro vs Clonaz | 2.64 | 1.08-5.45 |
| Lamot+Valpro vs Control | 3.01 | 1.60-5.27 |
| Lamot+Valpro vs Gabap | 3.13 | 1.28-7.48 |
| Lamot+Valpro vs Lamot | 3.26 | 1.78-5.57 |
| Lamot+Valpro vs Pheno+Pheny+Primid | 0.29 | 0.11-0.66 |
| Lamot+Valpro vs Pheno+Primid | 0.26 | 0.09-0.74 |
| Lamot+Valpro vs Primid | 2.32 | 1.09-4.94 |
| Levet vs Carbam+Clonaz | 0.08 | 0.03-0.28 |
| Levet vs Carbam+Pheno | 0.26 | 0.11-0.56 |
| Levet vs Carbam+Pheny | 0.17 | 0.03-0.61 |
| Levet vs Carbam+Pheny+Valpro | 0.10 | 0.02-0.98 |
| Levet vs Carbam+Valpro | 0.11 | 0.05-0.22 |
| Levet vs Clonaz+Valpro | 0.06 | 0.01-0.91 |
| Levet vs Ethos | 0.22 | 0.09-0.56 |
| Levet vs Lamot+Valpro | 0.23 | 0.12-0.48 |
| Levet vs Pheno | 0.39 | 0.22-0.65 |
| Levet vs Pheno+Pheny | 0.32 | 0.17-0.56 |
| Levet vs Pheno+Pheny+Primid | 0.07 | 0.03-0.16 |
| Levet vs Pheno+Primid | 0.06 | 0.02-0.16 |
| Levet vs Pheno+Valpro | 0.13 | 0.05-0.36 |
| Levet vs Pheny | 0.42 | 0.24-0.71 |
| Levet vs Pheny+Primid | 0.15 | 0.06-0.31 |
| Levet vs Pheny+Valpro | 0.14 | 0.05-0.41 |
| Levet vs Topir | 0.33 | 0.18-0.61 |
| Levet vs Valpro | 0.25 | 0.14-0.39 |
| Oxcar vs Carbam+Clonaz | 0.14 | 0.05-0.44 |
| Oxcar vs Carbam+Pheno | 0.40 | 0.21-0.92 |
| Oxcar vs Carbam+Pheny | 0.28 | 0.05-0.96 |
| Oxcar vs Carbam+Valpro | 0.18 | 0.09-0.35 |
| Oxcar vs Ethos | 0.36 | 0.14-0.93 |
| Oxcar vs Lamot+Valpro | 0.38 | 0.19-0.78 |
| Oxcar vs Pheno | 0.62 | 0.35-0.99 |
| Oxcar vs Pheno+Pheny | 0.52 | 0.29-0.88 |
| Oxcar vs Pheno+Pheny+Primid | 0.11 | 0.05-0.25 |
| Oxcar vs Pheno+Primid | 0.09 | 0.04-0.27 |
| Oxcar vs Pheno+Valpro | 0.22 | 0.09-0.57 |
| Oxcar vs Pheny+Primid | 0.23 | 0.10-0.52 |
| Oxcar vs Pheny+Valpro | 0.23 | 0.10-0.67 |
| Oxcar vs Topir | 0.54 | 0.28-0.94 |
| Oxcar vs Valpro | 0.40 | 0.23-0.62 |
| Pheno vs Control | 1.86 | 1.35-2.47 |
| Pheno+Pheny vs Control | 2.21 | 1.56-3.19 |
| Pheno+Pheny+Primid vs Carbam | 8.06 | 1.94-44.83 |
| Pheno+Pheny+Primid vs Carbam+Pheno | 3.69 | 1.62-10.13 |
| Pheno+Pheny+Primid vs Control | 10.23 | 5.21-22.02 |
| Pheno+Pheny+Primid vs Ethos | 3.37 | 1.12-9.59 |
| Pheno+Pheny+Primid vs Pheno | 5.70 | 2.72-11.59 |
| Pheno+Pheny+Primid vs Pheno+Pheny | 4.67 | 2.32-9.99 |
| Pheno+Pheny+Primid vs Pheny | 6.22 | 3.05-13.17 |
| Pheno+Pheny+Primid vs Primid | 7.96 | 3.16-20.41 |
| Pheno+Primid vs Carbam | 9.07 | 2.07-51.84 |
| Pheno+Primid vs Carbam+Pheno | 3.99 | 1.61-11.84 |
| Pheno+Primid vs Carbam+Pheno+Pheny | 3.73 | 1.09-24.49 |
| Pheno+Primid vs Control | 11.42 | 5.18-27.23 |
| Pheno+Primid vs Ethos | 3.59 | 1.19-13.03 |
| Pheno+Primid vs Pheno | 6.37 | 2.47-14.28 |
| Pheno+Primid vs Pheno+Pheny | 5.26 | 2.16-12.91 |
| Pheno+Primid vs Pheny | 7.33 | 2.92-16.11 |
| Pheno+Primid vs Primid | 9.44 | 3.47-23.15 |
| Pheno+Valpro vs Clonaz | 4.30 | 1.78-11.25 |
| Pheno+Valpro vs Control | 4.97 | 2.21-11.94 |
| Pheno+Valpro vs Pheno | 2.81 | 1.26-6.19 |
| Pheno+Valpro vs Pheny | 3.14 | 1.43-7.16 |
| Pheno+Valpro vs Primid | 4.13 | 1.51-10.20 |
| Pheny vs Control | 1.65 | 1.25-2.13 |
| Pheny+Primid vs Control | 5.01 | 2.46-10.77 |
| Pheny+Primid vs Pheno | 2.68 | 1.32-5.66 |
| Pheny+Primid vs Pheno+Pheny | 2.24 | 1.11-4.82 |
| Pheny+Primid vs Pheny | 2.96 | 1.50-6.55 |
| Pheny+Primid vs Primid | 3.78 | 1.55-9.98 |
| Pheny+Valpro vs Clonaz | 4.26 | 1.40-11.31 |
| Pheny+Valpro vs Control | 4.98 | 1.81-11.55 |
| Pheny+Valpro vs Pheno | 2.66 | 1.02-5.93 |
| Pheny+Valpro vs Pheny | 3.01 | 1.10-6.78 |
| Pheny+Valpro vs Primid | 3.86 | 1.25-10.62 |
| Primid+Valpro vs Pheno+Pheny+Primid | 0.10 | 0.01-0.89 |
| Primid+Valpro vs Pheno+Primid | 0.09 | 0.01-0.91 |
| Topir vs Carbam+Clonaz | 0.26 | 0.10-0.81 |
| Topir vs Carbam+Valpro | 0.34 | 0.18-0.64 |
| Topir vs Control | 2.11 | 1.41-3.20 |
| Topir vs Gabap | 2.17 | 1.04-4.93 |
| Topir vs Lamot | 2.29 | 1.53-3.46 |
| Topir vs Pheno+Pheny+Primid | 0.21 | 0.09-0.44 |
| Topir vs Pheno+Primid | 0.18 | 0.08-0.47 |
| Topir vs Pheno+Valpro | 0.41 | 0.17-0.96 |
| Topir vs Pheny+Primid | 0.43 | 0.19-0.94 |
| Valpro vs Clonaz | 2.41 | 1.39-4.71 |
| Valpro vs Control | 2.90 | 2.21-3.67 |
| Valpro vs Pheno | 1.58 | 1.23-2.00 |
| Valpro vs Pheno+Pheny+Primid | 0.28 | 0.13-0.56 |
| Valpro vs Pheno+Primid | 0.24 | 0.11-0.63 |
| Valpro vs Pheny | 1.76 | 1.39-2.22 |
| Valpro vs Primid | 2.24 | 1.25-3.89 |
| Vigab vs Pheno+Pheny+Primid | 0.21 | 0.04-0.82 |
| Vigab vs Pheno+Primid | 0.19 | 0.03-0.96 |
| *Common within-network between-study variance* | *0.31* | *0.00-3.58* |
| **Major Congenital Malformations SA Overlap^a^ – Observational Studies Only** | | |
| Carbam vs Control | 1.41 | 1.13-1.75 |
| Carbam vs Pheno | 0.75 | 0.59-0.97 |
| Carbam vs Pheno+Pheny | 0.64 | 0.45-0.91 |
| Carbam+Clonaz vs Carbam | 5.82 | 1.96-13.30 |
| Carbam+Clonaz vs Clonaz | 6.96 | 2.02-19.20 |
| Carbam+Clonaz vs Control | 8.01 | 2.81-19.30 |
| Carbam+Clonaz vs Pheno | 4.64 | 1.44-10.49 |
| Carbam+Clonaz vs Pheno+Pheny | 3.85 | 1.20-9.23 |
| Carbam+Clonaz vs Pheny | 5.04 | 1.62-11.51 |
| Carbam+Clonaz vs Primid | 6.42 | 1.96-17.54 |
| Carbam+Lamot vs Pheno+Pheny+Primid | 0.05 | 0.00-0.70 |
| Carbam+Lamot vs Pheno+Primid | 0.05 | 0.00-0.63 |
| Carbam+Pheno vs Carbam | 1.95 | 1.03-3.64 |
| Carbam+Pheno vs Control | 2.78 | 1.43-5.27 |
| Carbam+Pheny vs Carbam | 2.54 | 1.34-4.71 |
| Carbam+Pheny vs Control | 3.62 | 1.72-6.93 |
| Carbam+Pheny vs Pheno | 1.92 | 1.07-3.64 |
| Carbam+Pheny vs Pheny | 2.15 | 1.17-4.23 |
| Carbam+Pheny+primid vs Pheno+Pheny+Primid | 0.20 | 0.02-0.96 |
| Carbam+Pheny+primid vs Pheno+Primid | 0.16 | 0.02-0.98 |
| Carbam+Primid vs Pheno+Primid | 0.14 | 0.00-0.96 |
| Carbam+Valpro vs Carbam | 4.48 | 2.66-7.18 |
| Carbam+Valpro vs Carbam+Pheno | 2.31 | 1.06-5.23 |
| Carbam+Valpro vs Clonaz | 5.30 | 2.46-12.02 |
| Carbam+Valpro vs Control | 6.29 | 3.71-10.71 |
| Carbam+Valpro vs Pheno | 3.51 | 1.93-5.57 |
| Carbam+Valpro vs Pheno+Pheny | 2.92 | 1.55-5.13 |
| Carbam+Valpro vs Pheny | 3.89 | 2.24-6.32 |
| Carbam+Valpro vs Primid | 4.96 | 2.30-10.17 |
| Carbam+Valpro vs Valpro | 2.25 | 1.28-3.47 |
| Clobaz vs Pheno+Pheny+Primid | 0.22 | 0.06-0.89 |
| Clobaz vs Pheno+Primid | 0.21 | 0.05-0.87 |
| Clobaz+Oxcar vs Carbam+Clonaz | 0.05 | 0.00-0.88 |
| Clobaz+Oxcar vs Pheno+Pheny+Primid | 0.04 | 0.00-0.59 |
| Clobaz+Oxcar vs Pheno+Primid | 0.04 | 0.00-0.59 |
| Clonaz vs Carbam+Pheno | 0.43 | 0.17-0.97 |
| Clonaz vs Carbam+Pheny | 0.34 | 0.12-0.73 |
| Clonaz vs Pheno+Pheny+Primid | 0.12 | 0.04-0.28 |
| Clonaz vs Pheno+Primid | 0.10 | 0.04-0.28 |
| Clonaz vs Pheny+Primid | 0.24 | 0.09-0.59 |
| Clonaz+Oxcar vs Carbam+Clonaz | 0.06 | 0.00-0.86 |
| Clonaz+Oxcar vs Pheno+Pheny+Primid | 0.05 | 0.00-0.67 |
| Clonaz+Oxcar vs Pheno+Primid | 0.05 | 0.00-0.58 |
| Ethos vs Control | 3.21 | 1.37-7.28 |
| Gabap vs Carbam+Clonaz | 0.13 | 0.04-0.42 |
| Gabap vs Carbam+Pheno | 0.34 | 0.15-0.89 |
| Gabap vs Carbam+Pheny | 0.26 | 0.11-0.67 |
| Gabap vs Carbam+Valpro | 0.16 | 0.06-0.35 |
| Gabap vs Ethos | 0.30 | 0.11-0.89 |
| Gabap vs Pheno+Pheny | 0.45 | 0.20-0.91 |
| Gabap vs Pheno+Pheny+Primid | 0.10 | 0.03-0.24 |
| Gabap vs Pheno+Primid | 0.08 | 0.03-0.25 |
| Gabap vs Pheno+Valpro | 0.19 | 0.06-0.51 |
| Gabap vs Pheny+Primid | 0.20 | 0.07-0.50 |
| Gabap vs Pheny+Valpro | 0.19 | 0.06-0.63 |
| Gabap vs Valpro | 0.34 | 0.16-0.64 |
| Lamot vs Carbam | 0.66 | 0.52-0.82 |
| Lamot vs Carbam+Clonaz | 0.12 | 0.05-0.34 |
| Lamot vs Carbam+Pheno | 0.34 | 0.18-0.67 |
| Lamot vs Carbam+Pheny | 0.26 | 0.13-0.51 |
| Lamot vs Carbam+Valpro | 0.15 | 0.09-0.25 |
| Lamot vs Ethos | 0.30 | 0.13-0.69 |
| Lamot vs Pheno | 0.50 | 0.37-0.66 |
| Lamot vs Pheno+Pheny | 0.42 | 0.29-0.62 |
| Lamot vs Pheno+Pheny+Primid | 0.09 | 0.04-0.18 |
| Lamot vs Pheno+Primid | 0.08 | 0.03-0.21 |
| Lamot vs Pheno+Valpro | 0.18 | 0.07-0.40 |
| Lamot vs Pheny | 0.57 | 0.42-0.74 |
| Lamot vs Pheny+Primid | 0.19 | 0.09-0.38 |
| Lamot vs Pheny+Valpro | 0.19 | 0.08-0.49 |
| Lamot vs Valpro | 0.32 | 0.25-0.40 |
| Lamot+Levet vs Pheno+Primid | 0.05 | 0.00-0.95 |
| Lamot+Valpro vs Carbam | 2.15 | 1.17-3.60 |
| Lamot+Valpro vs Carbam+Valpro | 0.48 | 0.23-0.94 |
| Lamot+Valpro vs Clonaz | 2.64 | 1.08-5.45 |
| Lamot+Valpro vs Control | 3.01 | 1.60-5.27 |
| Lamot+Valpro vs Gabap | 3.13 | 1.28-7.48 |
| Lamot+Valpro vs Lamot | 3.26 | 1.78-5.57 |
| Lamot+Valpro vs Pheno+Pheny+Primid | 0.29 | 0.11-0.66 |
| Lamot+Valpro vs Pheno+Primid | 0.26 | 0.09-0.74 |
| Lamot+Valpro vs Primid | 2.32 | 1.09-4.94 |
| Levet vs Carbam | 0.50 | 0.30-0.83 |
| Levet vs Carbam+Clonaz | 0.08 | 0.03-0.28 |
| Levet vs Carbam+Pheno | 0.26 | 0.11-0.56 |
| Levet vs Carbam+Pheny | 0.20 | 0.08-0.39 |
| Levet vs Carbam+Pheny+Valpro | 0.10 | 0.02-0.98 |
| Levet vs Carbam+Valpro | 0.11 | 0.05-0.22 |
| Levet vs Clonaz+Valpro | 0.06 | 0.01-0.91 |
| Levet vs Ethos | 0.22 | 0.09-0.56 |
| Levet vs Lamot+Valpro | 0.23 | 0.12-0.48 |
| Levet vs Pheno | 0.39 | 0.22-0.65 |
| Levet vs Pheno+Pheny | 0.32 | 0.17-0.56 |
| Levet vs Pheno+Pheny+Primid | 0.07 | 0.03-0.16 |
| Levet vs Pheno+Primid | 0.06 | 0.02-0.16 |
| Levet vs Pheno+Valpro | 0.13 | 0.05-0.36 |
| Levet vs Pheny | 0.42 | 0.24-0.71 |
| Levet vs Pheny+Primid | 0.15 | 0.06-0.31 |
| Levet vs Pheny+Valpro | 0.14 | 0.05-0.41 |
| Levet vs Topir | 0.33 | 0.18-0.61 |
| Levet vs Valpro | 0.25 | 0.14-0.39 |
| Oxcar vs Carbam+Clonaz | 0.14 | 0.05-0.44 |
| Oxcar vs Carbam+Pheno | 0.40 | 0.21-0.92 |
| Oxcar vs Carbam+Pheny | 0.32 | 0.15-0.65 |
| Oxcar vs Carbam+Valpro | 0.18 | 0.09-0.35 |
| Oxcar vs Ethos | 0.36 | 0.14-0.93 |
| Oxcar vs Lamot+Valpro | 0.38 | 0.19-0.78 |
| Oxcar vs Pheno | 0.62 | 0.35-0.99 |
| Oxcar vs Pheno+Pheny | 0.52 | 0.29-0.88 |
| Oxcar vs Pheno+Pheny+Primid | 0.11 | 0.05-0.25 |
| Oxcar vs Pheno+Primid | 0.09 | 0.04-0.27 |
| Oxcar vs Pheno+Valpro | 0.22 | 0.09-0.57 |
| Oxcar vs Pheny+Primid | 0.23 | 0.10-0.52 |
| Oxcar vs Pheny+Valpro | 0.23 | 0.10-0.67 |
| Oxcar vs Topir | 0.54 | 0.28-0.94 |
| Oxcar vs Valpro | 0.40 | 0.23-0.62 |
| Pheno vs Control | 1.86 | 1.35-2.47 |
| Pheno+Pheny vs Control | 2.21 | 1.56-3.19 |
| Pheno+Pheny+Primid vs Carbam | 7.32 | 3.70-15.40 |
| Pheno+Pheny+Primid vs Carbam+Pheno | 3.69 | 1.62-10.13 |
| Pheno+Pheny+Primid vs Carbam+Pheny | 2.97 | 1.16-7.27 |
| Pheno+Pheny+Primid vs Control | 10.23 | 5.21-22.02 |
| Pheno+Pheny+Primid vs Ethos | 3.37 | 1.12-9.59 |
| Pheno+Pheny+Primid vs Pheno | 5.70 | 2.72-11.59 |
| Pheno+Pheny+Primid vs Pheno+Pheny | 4.67 | 2.32-9.99 |
| Pheno+Pheny+Primid vs Pheny | 6.22 | 3.05-13.17 |
| Pheno+Pheny+Primid vs Primid | 7.96 | 3.16-20.41 |
| Pheno+Primid vs Carbam | 8.49 | 3.39-18.78 |
| Pheno+Primid vs Carbam+Pheno | 3.99 | 1.61-11.84 |
| Pheno+Primid vs Carbam+Pheno+Pheny | 3.73 | 1.09-24.49 |
| Pheno+Primid vs Carbam+Pheny | 3.23 | 1.18-8.17 |
| Pheno+Primid vs Control | 11.42 | 5.18-27.23 |
| Pheno+Primid vs Ethos | 3.59 | 1.19-13.03 |
| Pheno+Primid vs Pheno | 6.37 | 2.47-14.28 |
| Pheno+Primid vs Pheno+Pheny | 5.26 | 2.16-12.91 |
| Pheno+Primid vs Pheny | 7.33 | 2.92-16.11 |
| Pheno+Primid vs Primid | 9.44 | 3.47-23.15 |
| Pheno+Valpro vs Carbam | 3.72 | 1.67-8.31 |
| Pheno+Valpro vs Clonaz | 4.30 | 1.78-11.25 |
| Pheno+Valpro vs Control | 4.97 | 2.21-11.94 |
| Pheno+Valpro vs Pheno | 2.81 | 1.26-6.19 |
| Pheno+Valpro vs Pheny | 3.14 | 1.43-7.16 |
| Pheno+Valpro vs Primid | 4.13 | 1.51-10.20 |
| Pheny vs Control | 1.65 | 1.25-2.13 |
| Pheny+Primid vs Carbam | 3.52 | 1.77-7.42 |
| Pheny+Primid vs Control | 5.01 | 2.46-10.77 |
| Pheny+Primid vs Pheno | 2.68 | 1.32-5.66 |
| Pheny+Primid vs Pheno+Pheny | 2.24 | 1.11-4.82 |
| Pheny+Primid vs Pheny | 2.96 | 1.50-6.55 |
| Pheny+Primid vs Primid | 3.78 | 1.55-9.98 |
| Pheny+Valpro vs Carbam | 3.52 | 1.34-7.92 |
| Pheny+Valpro vs Clonaz | 4.26 | 1.40-11.31 |
| Pheny+Valpro vs Control | 4.98 | 1.81-11.55 |
| Pheny+Valpro vs Pheno | 2.66 | 1.02-5.93 |
| Pheny+Valpro vs Pheny | 3.01 | 1.10-6.78 |
| Pheny+Valpro vs Primid | 3.86 | 1.25-10.62 |
| Primid vs Carbam+Pheny | 0.37 | 0.16-0.81 |
| Primid+Valpro vs Pheno+Pheny+Primid | 0.10 | 0.01-0.89 |
| Primid+Valpro vs Pheno+Primid | 0.09 | 0.01-0.91 |
| Topir vs Carbam | 1.52 | 1.01-2.20 |
| Topir vs Carbam+Clonaz | 0.26 | 0.10-0.81 |
| Topir vs Carbam+Valpro | 0.34 | 0.18-0.64 |
| Topir vs Control | 2.11 | 1.41-3.20 |
| Topir vs Gabap | 2.17 | 1.04-4.93 |
| Topir vs Lamot | 2.29 | 1.53-3.46 |
| Topir vs Pheno+Pheny+Primid | 0.21 | 0.09-0.44 |
| Topir vs Pheno+Primid | 0.18 | 0.08-0.47 |
| Topir vs Pheno+Valpro | 0.41 | 0.17-0.96 |
| Topir vs Pheny+Primid | 0.43 | 0.19-0.94 |
| Valpro vs Carbam | 2.07 | 1.74-2.44 |
| Valpro vs Clonaz | 2.41 | 1.39-4.71 |
| Valpro vs Control | 2.90 | 2.21-3.67 |
| Valpro vs Pheno | 1.58 | 1.23-2.00 |
| Valpro vs Pheno+Pheny+Primid | 0.28 | 0.13-0.56 |
| Valpro vs Pheno+Primid | 0.24 | 0.11-0.63 |
| Valpro vs Pheny | 1.76 | 1.39-2.22 |
| Valpro vs Primid | 2.24 | 1.25-3.89 |
| Vigab vs Pheno+Pheny+Primid | 0.21 | 0.04-0.82 |
| Vigab vs Pheno+Primid | 0.19 | 0.03-0.96 |
| *Common within-network between-study variance* | *0.03* | *0.00-0.12* |
| **Major Congenital Malformations SA Overlap^a^ – Randomised Controlled Trials Only** | | |
| Pheno vs Control | 4.07 | 1.20-21.71 |
| *Common within-network between-study variance* | *NA* | *NA* |

a Includes a large international registry study (EURAP)

1. Tomson T, Battino D, Bonizzoni E, Craig J, Lindhout D, Sabers A et al. Dose-dependent risk of malformations with antiepileptic drugs: an analysis of data from the EURAP epilepsy and pregnancy registry. Lancet Neurol. 2011;10(7):609-617.
2. Tomson T, Battino D, Bonizzoni E, Craig J, Lindhout D, Perucca E et al. Dose-dependent teratogenicity of valproate in mono- and polytherapy: an observational study. Neurology. 2015;85(10):866-872.

### Statistically Significant Sensitivity Results for Major Congenital Malformations

| **Treatment Comparison** | **Median Odds Ratio** | **Low CrI-High CrI** |
| --- | --- | --- |
| **Cohorts – 75 studies, 34667 cases, 48 treatments** | | |
| Carbam vs Control | 1.30 | 1.05-1.65 |
| Carbam vs Pheno | 0.74 | 0.57-0.99 |
| Carbam vs Pheno+Pheny | 0.61 | 0.43-0.90 |
| Carbam+Clonaz vs Carbam | 6.00 | 2.20-14.94 |
| Carbam+Clonaz vs Clonaz | 6.82 | 2.22-20.71 |
| Carbam+Clonaz vs Control | 7.69 | 2.82-20.05 |
| Carbam+Clonaz vs Pheno | 4.38 | 1.59-11.31 |
| Carbam+Clonaz vs Pheno+Pheny | 3.61 | 1.34-9.82 |
| Carbam+Clonaz vs Pheny | 4.82 | 1.81-12.68 |
| Carbam+Clonaz vs Primid | 6.44 | 2.06-23.63 |
| Carbam+Lamot vs Carbam+Clonaz | 0.06 | 0.00-0.93 |
| Carbam+Lamot vs Pheno+Pheny+Primid | 0.05 | 0.00-0.65 |
| Carbam+Lamot vs Pheno+Primid | 0.04 | 0.00-0.66 |
| Carbam+Pheno vs Carbam | 1.98 | 1.05-3.62 |
| Carbam+Pheno vs Control | 2.59 | 1.36-4.71 |
| Carbam+Pheny vs Carbam | 2.50 | 1.20-5.17 |
| Carbam+Pheny vs Control | 3.27 | 1.53-6.71 |
| Carbam+Primid vs Pheno+Pheny+Primid | 0.12 | 0.00-0.96 |
| Carbam+Primid vs Pheno+Primid | 0.11 | 0.00-0.94 |
| Carbam+Valpro vs Carbam | 4.29 | 2.41-7.41 |
| Carbam+Valpro vs Clonaz | 4.97 | 2.27-11.20 |
| Carbam+Valpro vs Control | 5.62 | 3.00-9.72 |
| Carbam+Valpro vs Pheno | 3.19 | 1.73-5.76 |
| Carbam+Valpro vs Pheno+Pheny | 2.66 | 1.39-4.99 |
| Carbam+Valpro vs Pheny | 3.47 | 1.91-6.18 |
| Carbam+Valpro vs Primid | 4.75 | 2.08-12.23 |
| Carbam+Valpro vs Valpro | 1.95 | 1.09-3.41 |
| Clobaz+Oxcar vs Carbam+Clonaz | 0.04 | 0.00-0.93 |
| Clobaz+Oxcar vs Pheno+Pheny+Primid | 0.03 | 0.00-0.67 |
| Clobaz+Oxcar vs Pheno+Primid | 0.03 | 0.00-0.66 |
| Clonaz vs Carbam+Pheno | 0.44 | 0.19-0.97 |
| Clonaz vs Carbam+Pheny | 0.34 | 0.14-0.84 |
| Clonaz vs Pheno+Pheny | 0.53 | 0.28-0.98 |
| Clonaz vs Pheno+Pheny+Primid | 0.11 | 0.04-0.29 |
| Clonaz vs Pheno+Primid | 0.10 | 0.04-0.28 |
| Clonaz vs Pheny+Primid | 0.23 | 0.10-0.57 |
| Clonaz+Oxcar vs Carbam+Clonaz | 0.05 | 0.00-0.99 |
| Clonaz+Oxcar vs Pheno+Pheny+Primid | 0.04 | 0.00-0.63 |
| Clonaz+Oxcar vs Pheno+Primid | 0.04 | 0.00-0.73 |
| Ethos vs Control | 2.79 | 1.04-6.95 |
| Gabap vs Carbam+Clonaz | 0.12 | 0.04-0.40 |
| Gabap vs Carbam+Pheno | 0.37 | 0.13-0.97 |
| Gabap vs Carbam+Pheny | 0.29 | 0.10-0.83 |
| Gabap vs Carbam+Valpro | 0.17 | 0.06-0.40 |
| Gabap vs Clonaz+Valpro | 0.07 | 0.01-0.95 |
| Gabap vs Pheno+Pheny | 0.46 | 0.18-0.94 |
| Gabap vs Pheno+Pheny+Primid | 0.10 | 0.03-0.25 |
| Gabap vs Pheno+Primid | 0.08 | 0.03-0.28 |
| Gabap vs Pheno+Valpro | 0.19 | 0.06-0.51 |
| Gabap vs Pheny+Primid | 0.20 | 0.07-0.52 |
| Gabap vs Pheny+Valpro | 0.20 | 0.06-0.60 |
| Gabap vs Valpro | 0.34 | 0.14-0.63 |
| Lamot vs Carbam | 0.72 | 0.56-0.90 |
| Lamot vs Carbam+Clonaz | 0.12 | 0.05-0.34 |
| Lamot vs Carbam+Pheno | 0.36 | 0.19-0.70 |
| Lamot vs Carbam+Pheny | 0.29 | 0.14-0.60 |
| Lamot vs Carbam+Valpro | 0.17 | 0.09-0.31 |
| Lamot vs Clonaz+Valpro | 0.07 | 0.01-0.93 |
| Lamot vs Ethos | 0.33 | 0.14-0.91 |
| Lamot vs Pheno | 0.53 | 0.39-0.75 |
| Lamot vs Pheno+Pheny | 0.45 | 0.30-0.67 |
| Lamot vs Pheno+Pheny+Primid | 0.09 | 0.04-0.21 |
| Lamot vs Pheno+Primid | 0.09 | 0.03-0.22 |
| Lamot vs Pheno+Valpro | 0.19 | 0.08-0.44 |
| Lamot vs Pheny | 0.59 | 0.43-0.78 |
| Lamot vs Pheny+Primid | 0.2 | 0.10-0.42 |
| Lamot vs Pheny+Valpro | 0.19 | 0.08-0.50 |
| Lamot vs Valpro | 0.33 | 0.26-0.41 |
| Lamot+Valpro vs Carbam+Clonaz | 0.15 | 0.02-0.95 |
| Lamot+Valpro vs Carbam+Valpro | 0.20 | 0.03-1.00 |
| Lamot+Valpro vs Pheno+Pheny+Primid | 0.11 | 0.02-0.61 |
| Lamot+Valpro vs Pheno+Primid | 0.10 | 0.01-0.63 |
| Levet vs Carbam | 0.55 | 0.32-0.87 |
| Levet vs Carbam+Clonaz | 0.09 | 0.03-0.27 |
| Levet vs Carbam+Pheno | 0.27 | 0.13-0.61 |
| Levet vs Carbam+Pheno+Pheny | 0.22 | 0.06-0.78 |
| Levet vs Carbam+Pheny | 0.22 | 0.09-0.50 |
| Levet vs Carbam+Pheny+Valpro | 0.12 | 0.03-0.89 |
| Levet vs Carbam+Valpro | 0.13 | 0.06-0.27 |
| Levet vs Clonaz+Valpro | 0.06 | 0.01-0.72 |
| Levet vs Ethos | 0.25 | 0.10-0.73 |
| Levet vs Pheno | 0.41 | 0.23-0.69 |
| Levet vs Pheno+Pheny | 0.34 | 0.18-0.59 |
| Levet vs Pheno+Pheny+Primid | 0.07 | 0.03-0.17 |
| Levet vs Pheno+Primid | 0.06 | 0.02-0.18 |
| Levet vs Pheno+Valpro | 0.14 | 0.05-0.36 |
| Levet vs Pheny | 0.45 | 0.26-0.72 |
| Levet vs Pheny+Primid | 0.15 | 0.06-0.35 |
| Levet vs Pheny+Valpro | 0.14 | 0.05-0.43 |
| Levet vs Topir | 0.38 | 0.20-0.70 |
| Levet vs Valpro | 0.25 | 0.15-0.39 |
| Oxcar vs Carbam+Clonaz | 0.16 | 0.05-0.50 |
| Oxcar vs Carbam+Valpro | 0.23 | 0.10-0.48 |
| Oxcar vs Pheno+Pheny+Primid | 0.13 | 0.05-0.32 |
| Oxcar vs Pheno+Primid | 0.11 | 0.04-0.34 |
| Oxcar vs Pheno+Valpro | 0.25 | 0.09-0.68 |
| Oxcar vs Pheny+Primid | 0.26 | 0.11-0.66 |
| Oxcar vs Pheny+Valpro | 0.26 | 0.09-0.82 |
| Oxcar vs Valpro | 0.44 | 0.24-0.77 |
| Pheno vs Control | 1.76 | 1.28-2.37 |
| Pheno+Pheny vs Control | 2.10 | 1.43-3.05 |
| Pheno+Pheny+Primid vs Carbam | 7.70 | 3.54-17.50 |
| Pheno+Pheny+Primid vs Carbam+Pheno | 3.91 | 1.55-9.71 |
| Pheno+Pheny+Primid vs Carbam+Pheny | 3.08 | 1.18-8.28 |
| Pheno+Pheny+Primid vs Control | 10.02 | 4.50-22.15 |
| Pheno+Pheny+Primid vs Ethos | 3.58 | 1.10-11.93 |
| Pheno+Pheny+Primid vs Pheno | 5.70 | 2.62-12.29 |
| Pheno+Pheny+Primid vs Pheno+Pheny | 4.76 | 2.16-10.21 |
| Pheno+Pheny+Primid vs Pheny | 6.27 | 2.86-13.77 |
| Pheno+Pheny+Primid vs Primid | 8.60 | 3.05-24.89 |
| Pheno+Primid vs Carbam | 8.42 | 3.27-20.37 |
| Pheno+Primid vs Carbam+Pheno | 4.26 | 1.42-12.91 |
| Pheno+Primid vs Carbam+Pheny | 3.37 | 1.10-10.62 |
| Pheno+Primid vs Control | 11.02 | 4.24-27.48 |
| Pheno+Primid vs Ethos | 4.01 | 1.07-15.44 |
| Pheno+Primid vs Pheno | 6.25 | 2.40-15.94 |
| Pheno+Primid vs Pheno+Pheny | 5.22 | 1.96-12.99 |
| Pheno+Primid vs Pheny | 6.88 | 2.66-17.03 |
| Pheno+Primid vs Primid | 9.32 | 3.00-31.12 |
| Pheno+Valpro vs Carbam | 3.85 | 1.67-8.81 |
| Pheno+Valpro vs Clonaz | 4.38 | 1.71-12.66 |
| Pheno+Valpro vs Control | 5.04 | 2.19-11.63 |
| Pheno+Valpro vs Pheno | 2.88 | 1.27-6.64 |
| Pheno+Valpro vs Pheno+Pheny | 2.39 | 1.05-5.55 |
| Pheno+Valpro vs Pheny | 3.13 | 1.34-7.48 |
| Pheno+Valpro vs Primid | 4.28 | 1.49-12.70 |
| Pheny vs Control | 1.60 | 1.22-2.10 |
| Pheny+Primid vs Carbam | 3.74 | 1.74-7.32 |
| Pheny+Primid vs Control | 4.85 | 2.26-9.94 |
| Pheny+Primid vs Pheno | 2.72 | 1.29-5.65 |
| Pheny+Primid vs Pheno+Pheny | 2.28 | 1.09-4.75 |
| Pheny+Primid vs Pheny | 3.04 | 1.43-5.97 |
| Pheny+Primid vs Primid | 4.04 | 1.58-11.51 |
| Pheny+Valpro vs Carbam | 3.80 | 1.45-8.62 |
| Pheny+Valpro vs Clonaz | 4.31 | 1.54-11.94 |
| Pheny+Valpro vs Control | 4.93 | 1.87-11.27 |
| Pheny+Valpro vs Pheno | 2.82 | 1.07-6.49 |
| Pheny+Valpro vs Pheny | 3.10 | 1.17-7.00 |
| Pheny+Valpro vs Primid | 4.06 | 1.31-13.83 |
| Primid vs Carbam+Pheny | 0.36 | 0.13-0.94 |
| Primid+Valpro vs Pheno+Pheny+Primid | 0.12 | 0.00-0.92 |
| Primid+Valpro vs Pheno+Primid | 0.11 | 0.00-0.92 |
| Topir vs Carbam+Clonaz | 0.24 | 0.08-0.70 |
| Topir vs Carbam+Valpro | 0.33 | 0.17-0.65 |
| Topir vs Control | 1.86 | 1.19-2.90 |
| Topir vs Lamot | 1.98 | 1.26-3.03 |
| Topir vs Pheno+Pheny+Primid | 0.19 | 0.08-0.45 |
| Topir vs Pheno+Primid | 0.17 | 0.06-0.47 |
| Topir vs Pheno+Valpro | 0.37 | 0.15-0.91 |
| Topir vs Pheny+Primid | 0.38 | 0.17-0.88 |
| Topir vs Valpro | 0.65 | 0.42-0.98 |
| Valpro vs Carbam | 2.20 | 1.84-2.60 |
| Valpro vs Clonaz | 2.54 | 1.49-4.62 |
| Valpro vs Control | 2.85 | 2.30-3.57 |
| Valpro vs Pheno | 1.62 | 1.24-2.21 |
| Valpro vs Pheno+Pheny+Primid | 0.29 | 0.13-0.62 |
| Valpro vs Pheno+Primid | 0.26 | 0.11-0.67 |
| Valpro vs Pheny | 1.79 | 1.40-2.30 |
| Valpro vs Primid | 2.40 | 1.26-5.67 |
| Vigab vs Pheno+Primid | 0.20 | 0.03-1.00 |
| *Common within-network between-study variance* | *0.02* | *0.00-0.11* |
| **Timing (1^st^ Trimester & Whole PG) – 50 studies, 25329 cases, 46 treatments** | | |
| Carbam vs Pheno | 0.65 | 0.46-0.91 |
| Carbam vs Pheno+Pheny | 0.60 | 0.40-0.94 |
| Carbam+Clonaz vs Carbam | 5.55 | 1.77-14.76 |
| Carbam+Clonaz vs Clonaz | 5.45 | 1.66-18.51 |
| Carbam+Clonaz vs Control | 7.76 | 2.59-22.13 |
| Carbam+Clonaz vs Pheno | 3.62 | 1.14-9.88 |
| Carbam+Clonaz vs Pheno+Pheny | 3.34 | 1.07-9.34 |
| Carbam+Clonaz vs Pheny | 4.39 | 1.39-12.08 |
| Carbam+Lamot vs Carbam+Clonaz | 0.05 | 0.00-0.83 |
| Carbam+Lamot vs Carbam+Valpro | 0.06 | 0.00-0.78 |
| Carbam+Lamot vs Pheno+Pheny+Primid | 0.03 | 0.00-0.53 |
| Carbam+Lamot vs Pheno+Primid | 0.02 | 0.00-0.42 |
| Carbam+Pheno vs Carbam | 2.23 | 1.01-4.36 |
| Carbam+Pheno vs Control | 3.14 | 1.43-6.75 |
| Carbam+Pheny vs Carbam | 2.26 | 1.07-4.59 |
| Carbam+Pheny vs Control | 3.22 | 1.56-6.96 |
| Carbam+Pheny+primid vs Pheno+Primid | 0.13 | 0.01-0.86 |
| Carbam+Pheny+Valpro vs Control | 8.00 | 1.02-32.61 |
| Carbam+Primid vs Pheno+Pheny+Primid | 0.14 | 0.01-0.99 |
| Carbam+Primid vs Pheno+Primid | 0.10 | 0.01-0.77 |
| Carbam+Valpro vs Carbam | 4.70 | 2.71-7.73 |
| Carbam+Valpro vs Clonaz | 4.59 | 2.18-11.00 |
| Carbam+Valpro vs Control | 6.63 | 3.83-11.74 |
| Carbam+Valpro vs Pheno | 3.02 | 1.71-5.12 |
| Carbam+Valpro vs Pheno+Pheny | 2.81 | 1.52-5.00 |
| Carbam+Valpro vs Pheny | 3.74 | 2.14-6.27 |
| Carbam+Valpro vs Valpro | 2.12 | 1.24-3.51 |
| Clobaz+Oxcar vs Pheno+Pheny+Primid | 0.05 | 0.00-0.90 |
| Clobaz+Oxcar vs Pheno+Primid | 0.04 | 0.00-0.60 |
| Clonaz vs Pheno+Pheny+Primid | 0.13 | 0.04-0.35 |
| Clonaz vs Pheno+Primid | 0.09 | 0.02-0.31 |
| Clonaz+Oxcar vs Carbam+Clonaz | 0.06 | 0.00-1.00 |
| Clonaz+Oxcar vs Pheno+Pheny+Primid | 0.04 | 0.00-0.73 |
| Clonaz+Oxcar vs Pheno+Primid | 0.03 | 0.00-0.54 |
| Gabap vs Carbam+Clonaz | 0.11 | 0.03-0.38 |
| Gabap vs Carbam+Pheno | 0.26 | 0.10-0.71 |
| Gabap vs Carbam+Pheny | 0.26 | 0.09-0.69 |
| Gabap vs Carbam+Pheny+Valpro | 0.10 | 0.02-0.89 |
| Gabap vs Carbam+Valpro | 0.13 | 0.05-0.30 |
| Gabap vs Pheno | 0.38 | 0.16-0.82 |
| Gabap vs Pheno+Pheny | 0.36 | 0.14-0.81 |
| Gabap vs Pheno+Pheny+Primid | 0.08 | 0.03-0.21 |
| Gabap vs Pheno+Primid | 0.06 | 0.02-0.19 |
| Gabap vs Pheno+Valpro | 0.14 | 0.04-0.41 |
| Gabap vs Pheny+Primid | 0.19 | 0.06-0.74 |
| Gabap vs Pheny+Valpro | 0.19 | 0.06-0.73 |
| Gabap vs Valpro | 0.27 | 0.11-0.53 |
| Lamot vs Carbam | 0.68 | 0.49-0.86 |
| Lamot vs Carbam+Clonaz | 0.12 | 0.04-0.38 |
| Lamot vs Carbam+Pheno | 0.30 | 0.14-0.67 |
| Lamot vs Carbam+Pheny | 0.29 | 0.14-0.63 |
| Lamot vs Carbam+Pheny+Valpro | 0.12 | 0.03-0.91 |
| Lamot vs Carbam+Valpro | 0.14 | 0.08-0.25 |
| Lamot vs Pheno | 0.43 | 0.30-0.63 |
| Lamot vs Pheno+Pheny | 0.40 | 0.25-0.66 |
| Lamot vs Pheno+Pheny+Primid | 0.09 | 0.04-0.19 |
| Lamot vs Pheno+Primid | 0.06 | 0.02-0.18 |
| Lamot vs Pheno+Valpro | 0.16 | 0.06-0.39 |
| Lamot vs Pheny | 0.53 | 0.38-0.75 |
| Lamot vs Pheny+Primid | 0.22 | 0.10-0.68 |
| Lamot vs Pheny+Valpro | 0.22 | 0.09-0.67 |
| Lamot vs Valpro | 0.30 | 0.23-0.39 |
| Lamot+Levet vs Pheno+Pheny+Primid | 0.02 | 0.00-0.65 |
| Lamot+Levet vs Pheno+Primid | 0.02 | 0.00-0.54 |
| Lamot+Valpro vs Carbam+Clonaz | 0.03 | 0.00-0.51 |
| Lamot+Valpro vs Carbam+Pheno+Pheny+primid | 0.03 | 0.00-0.94 |
| Lamot+Valpro vs Carbam+Pheny | 0.07 | 0.00-1.00 |
| Lamot+Valpro vs Carbam+Pheny+Valpro | 0.03 | 0.00-0.72 |
| Lamot+Valpro vs Carbam+Valpro | 0.03 | 0.00-0.46 |
| Lamot+Valpro vs Pheno+Pheny+Primid | 0.02 | 0.00-0.30 |
| Lamot+Valpro vs Pheno+Primid | 0.01 | 0.00-0.24 |
| Lamot+Valpro vs Pheno+Valpro | 0.04 | 0.00-0.57 |
| Lamot+Valpro vs Pheny+Primid | 0.05 | 0.00-0.84 |
| Lamot+Valpro vs Pheny+Primid+Valpro | 0.03 | 0.00-0.97 |
| Lamot+Valpro vs Pheny+Valpro | 0.05 | 0.00-0.89 |
| Lamot+Valpro vs Valpro | 0.07 | 0.00-0.89 |
| Levet vs Carbam | 0.53 | 0.30-0.86 |
| Levet vs Carbam+Clonaz | 0.10 | 0.03-0.32 |
| Levet vs Carbam+Pheno | 0.24 | 0.10-0.58 |
| Levet vs Carbam+Pheny | 0.24 | 0.10-0.55 |
| Levet vs Carbam+Pheny+Valpro | 0.09 | 0.02-0.80 |
| Levet vs Carbam+Valpro | 0.11 | 0.05-0.23 |
| Levet vs Ethos | 0.28 | 0.11-0.88 |
| Levet vs Pheno | 0.34 | 0.19-0.60 |
| Levet vs Pheno+Pheny | 0.32 | 0.16-0.60 |
| Levet vs Pheno+Pheny+Primid | 0.07 | 0.03-0.17 |
| Levet vs Pheno+Primid | 0.05 | 0.02-0.16 |
| Levet vs Pheno+Valpro | 0.12 | 0.04-0.34 |
| Levet vs Pheny | 0.43 | 0.24-0.74 |
| Levet vs Pheny+Primid | 0.18 | 0.07-0.57 |
| Levet vs Pheny+Valpro | 0.17 | 0.06-0.57 |
| Levet vs Primid | 0.24 | 0.08-0.98 |
| Levet vs Topir | 0.38 | 0.19-0.70 |
| Levet vs Valpro | 0.24 | 0.14-0.39 |
| Oxcar vs Carbam+Clonaz | 0.21 | 0.06-0.73 |
| Oxcar vs Carbam+Valpro | 0.25 | 0.11-0.54 |
| Oxcar vs Pheno+Pheny+Primid | 0.14 | 0.05-0.40 |
| Oxcar vs Pheno+Primid | 0.10 | 0.04-0.34 |
| Oxcar vs Pheno+Valpro | 0.27 | 0.08-0.81 |
| Oxcar vs Valpro | 0.52 | 0.27-0.93 |
| Pheno vs Control | 2.21 | 1.52-3.25 |
| Pheno+Pheny vs Control | 2.41 | 1.28-3.84 |
| Pheno+Pheny+Primid vs Carbam | 7.67 | 3.49-16.70 |
| Pheno+Pheny+Primid vs Carbam+Pheno | 3.47 | 1.33-9.85 |
| Pheno+Pheny+Primid vs Carbam+Pheno+Pheny | 4.38 | 1.06-28.40 |
| Pheno+Pheny+Primid vs Carbam+Pheny | 3.47 | 1.30-9.17 |
| Pheno+Pheny+Primid vs Control | 10.85 | 5.04-24.63 |
| Pheno+Pheny+Primid vs Ethos | 4.05 | 1.35-14.29 |
| Pheno+Pheny+Primid vs Pheno | 4.93 | 2.30-10.88 |
| Pheno+Pheny+Primid vs Pheno+Pheny | 4.60 | 2.13-10.68 |
| Pheno+Pheny+Primid vs Pheny | 6.12 | 2.82-13.93 |
| Pheno+Pheny+Primid vs Pheny+Primid | 2.67 | 1.02-8.10 |
| Pheno+Pheny+Primid vs Primid | 3.65 | 1.09-14.96 |
| Pheno+Primid vs Carbam | 10.86 | 3.73-31.06 |
| Pheno+Primid vs Carbam+Pheno | 4.86 | 1.59-15.56 |
| Pheno+Primid vs Carbam+Pheno+Pheny | 6.01 | 1.40-43.55 |
| Pheno+Primid vs Carbam+Pheny | 4.87 | 1.51-15.15 |
| Pheno+Primid vs Control | 15.51 | 5.26-41.11 |
| Pheno+Primid vs Ethos | 5.51 | 1.47-23.32 |
| Pheno+Primid vs Pheno | 7.05 | 2.52-17.69 |
| Pheno+Primid vs Pheno+Pheny | 6.57 | 2.15-21.97 |
| Pheno+Primid vs Pheny | 8.64 | 2.89-22.86 |
| Pheno+Primid vs Pheny+Primid | 3.74 | 1.04-14.36 |
| Pheno+Primid vs Primid | 5.14 | 1.15-23.09 |
| Pheno+Valpro vs Carbam | 4.16 | 1.71-10.53 |
| Pheno+Valpro vs Clonaz | 4.20 | 1.43-13.51 |
| Pheno+Valpro vs Control | 5.99 | 2.39-15.94 |
| Pheno+Valpro vs Pheno | 2.84 | 1.11-6.92 |
| Pheno+Valpro vs Pheno+Pheny | 2.47 | 1.01-7.34 |
| Pheno+Valpro vs Pheny | 3.34 | 1.35-8.61 |
| Pheny vs Control | 1.80 | 1.25-2.62 |
| Pheny+Primid vs Control | 4.20 | 1.48-10.19 |
| Pheny+Valpro vs Control | 4.34 | 1.44-11.11 |
| Topir vs Carbam+Clonaz | 0.26 | 0.09-0.86 |
| Topir vs Carbam+Valpro | 0.31 | 0.16-0.61 |
| Topir vs Control | 2.04 | 1.22-3.42 |
| Topir vs Gabap | 2.41 | 1.10-6.12 |
| Topir vs Lamot | 2.14 | 1.31-3.46 |
| Topir vs Pheno+Pheny+Primid | 0.18 | 0.08-0.45 |
| Topir vs Pheno+Primid | 0.13 | 0.05-0.41 |
| Topir vs Pheno+Valpro | 0.33 | 0.12-0.92 |
| Valpro vs Carbam | 2.22 | 1.76-2.68 |
| Valpro vs Clonaz | 2.21 | 1.17-4.52 |
| Valpro vs Control | 3.18 | 2.27-4.39 |
| Valpro vs Pheno+Pheny+Primid | 0.29 | 0.13-0.63 |
| Valpro vs Pheno+Primid | 0.20 | 0.08-0.59 |
| Valpro vs Pheny | 1.77 | 1.33-2.38 |
| Vigab vs Pheno+Pheny+Primid | 0.11 | 0.00-0.92 |
| Vigab vs Pheno+Primid | 0.08 | 0.00-0.70 |
| *Common within-network between-study variance* | *0.04* | *0.00-0.17* |
| **Treatment indication = Epilepsy – 71 studies, 30289 cases, 47 treatments** | | |
| Carbam vs Control | 1.39 | 1.10-1.75 |
| Carbam vs Pheno+Pheny | 0.65 | 0.45-0.94 |
| Carbam+Clonaz vs Carbam | 6.33 | 2.29-15.17 |
| Carbam+Clonaz vs Clonaz | 9.52 | 2.97-31.35 |
| Carbam+Clonaz vs Control | 8.85 | 3.23-21.69 |
| Carbam+Clonaz vs Pheno | 4.90 | 1.72-12.02 |
| Carbam+Clonaz vs Pheno+Pheny | 4.10 | 1.48-10.51 |
| Carbam+Clonaz vs Pheny | 5.45 | 1.97-12.86 |
| Carbam+Clonaz vs Primid | 7.48 | 2.50-22.58 |
| Carbam+Lamot vs Pheno+Pheny+Primid | 0.04 | 0.00-0.76 |
| Carbam+Lamot vs Pheno+Primid | 0.04 | 0.00-0.73 |
| Carbam+Pheno vs Carbam | 2.12 | 1.12-3.69 |
| Carbam+Pheno vs Control | 2.96 | 1.46-5.20 |
| Carbam+Pheny vs Carbam | 2.71 | 1.53-4.45 |
| Carbam+Pheny vs Control | 3.76 | 2.16-6.32 |
| Carbam+Pheny vs Pheno | 2.07 | 1.15-3.59 |
| Carbam+Pheny vs Pheny | 2.34 | 1.31-3.89 |
| Carbam+Pheny+Valpro vs Control | 6.83 | 1.02-29.34 |
| Carbam+Valpro vs Carbam | 4.64 | 2.68-7.83 |
| Carbam+Valpro vs Carbam+Pheno | 2.15 | 1.08-5.10 |
| Carbam+Valpro vs Clonaz | 7.09 | 2.98-18.18 |
| Carbam+Valpro vs Control | 6.46 | 3.62-11.00 |
| Carbam+Valpro vs Pheno | 3.55 | 1.98-6.23 |
| Carbam+Valpro vs Pheno+Pheny | 3.04 | 1.60-5.37 |
| Carbam+Valpro vs Pheny | 4.02 | 2.23-6.88 |
| Carbam+Valpro vs Primid | 5.60 | 2.57-12.56 |
| Carbam+Valpro vs Valpro | 2.07 | 1.18-3.51 |
| Clobaz+Oxcar vs Carbam+Clonaz | 0.03 | 0.00-0.62 |
| Clobaz+Oxcar vs Carbam+Valpro | 0.04 | 0.00-0.77 |
| Clobaz+Oxcar vs Clonaz+Valpro | 0.02 | 0.00-0.95 |
| Clobaz+Oxcar vs Pheno+Pheny+Primid | 0.02 | 0.00-0.53 |
| Clobaz+Oxcar vs Pheno+Primid | 0.02 | 0.00-0.51 |
| Clonaz vs Carbam+Pheno | 0.31 | 0.11-0.80 |
| Clonaz vs Carbam+Pheny | 0.24 | 0.09-0.58 |
| Clonaz vs Pheno+Pheny | 0.43 | 0.18-0.95 |
| Clonaz vs Pheno+Pheny+Primid | 0.09 | 0.03-0.24 |
| Clonaz vs Pheno+Primid | 0.08 | 0.03-0.25 |
| Clonaz vs Pheny+Primid | 0.18 | 0.06-0.56 |
| Clonaz+Oxcar vs Carbam+Clonaz | 0.04 | 0.00-0.71 |
| Clonaz+Oxcar vs Carbam+Valpro | 0.06 | 0.00-0.91 |
| Clonaz+Oxcar vs Pheno+Pheny+Primid | 0.04 | 0.00-0.62 |
| Clonaz+Oxcar vs Pheno+Primid | 0.03 | 0.00-0.52 |
| Clonaz+Valpro vs Clonaz | 13.57 | 1.36-95.51 |
| Clonaz+Valpro vs Control | 12.66 | 1.26-75.43 |
| Ethos vs Control | 3.23 | 1.05-7.91 |
| Gabap vs Carbam+Clonaz | 0.15 | 0.04-0.50 |
| Gabap vs Carbam+Pheny | 0.35 | 0.13-0.92 |
| Gabap vs Carbam+Valpro | 0.21 | 0.07-0.50 |
| Gabap vs Pheno+Pheny+Primid | 0.13 | 0.04-0.38 |
| Gabap vs Pheno+Primid | 0.11 | 0.03-0.39 |
| Gabap vs Pheno+Valpro | 0.24 | 0.07-0.82 |
| Gabap vs Pheny+Primid | 0.26 | 0.08-0.85 |
| Gabap vs Pheny+Valpro | 0.27 | 0.07-0.85 |
| Gabap vs Valpro | 0.42 | 0.17-0.95 |
| Lamot vs Carbam | 0.69 | 0.52-0.90 |
| Lamot vs Carbam+Clonaz | 0.11 | 0.04-0.30 |
| Lamot vs Carbam+Pheno | 0.33 | 0.18-0.65 |
| Lamot vs Carbam+Pheny | 0.26 | 0.15-0.46 |
| Lamot vs Carbam+Pheny+Valpro | 0.14 | 0.03-0.98 |
| Lamot vs Carbam+Valpro | 0.15 | 0.09-0.27 |
| Lamot vs Clonaz+Valpro | 0.08 | 0.01-0.78 |
| Lamot vs Ethos | 0.29 | 0.12-0.99 |
| Lamot vs Pheno | 0.53 | 0.37-0.76 |
| Lamot vs Pheno+Pheny | 0.45 | 0.30-0.69 |
| Lamot vs Pheno+Pheny+Primid | 0.09 | 0.04-0.20 |
| Lamot vs Pheno+Primid | 0.08 | 0.04-0.22 |
| Lamot vs Pheno+Valpro | 0.18 | 0.07-0.42 |
| Lamot vs Pheny | 0.60 | 0.43-0.82 |
| Lamot vs Pheny+Primid | 0.19 | 0.09-0.48 |
| Lamot vs Pheny+Valpro | 0.20 | 0.07-0.49 |
| Lamot vs Valpro | 0.31 | 0.24-0.40 |
| Lamot+Levet vs Pheno+Pheny+Primid | 0.06 | 0.00-0.86 |
| Lamot+Levet vs Pheno+Primid | 0.05 | 0.00-0.81 |
| Lamot+Valpro vs Carbam+Clonaz | 0.12 | 0.01-0.85 |
| Lamot+Valpro vs Carbam+Valpro | 0.18 | 0.02-0.85 |
| Lamot+Valpro vs Pheno+Pheny+Primid | 0.11 | 0.01-0.59 |
| Lamot+Valpro vs Pheno+Primid | 0.10 | 0.01-0.58 |
| Levet vs Carbam | 0.51 | 0.30-0.85 |
| Levet vs Carbam+Clonaz | 0.08 | 0.03-0.24 |
| Levet vs Carbam+Pheno | 0.24 | 0.12-0.53 |
| Levet vs Carbam+Pheny | 0.19 | 0.09-0.39 |
| Levet vs Carbam+Pheny+Valpro | 0.10 | 0.02-0.79 |
| Levet vs Carbam+Valpro | 0.11 | 0.05-0.22 |
| Levet vs Clonaz+Valpro | 0.06 | 0.01-0.61 |
| Levet vs Ethos | 0.22 | 0.08-0.76 |
| Levet vs Pheno | 0.39 | 0.22-0.68 |
| Levet vs Pheno+Pheny | 0.34 | 0.18-0.60 |
| Levet vs Pheno+Pheny+Primid | 0.07 | 0.03-0.17 |
| Levet vs Pheno+Primid | 0.06 | 0.02-0.17 |
| Levet vs Pheno+Valpro | 0.13 | 0.05-0.34 |
| Levet vs Pheny | 0.45 | 0.25-0.75 |
| Levet vs Pheny+Primid | 0.14 | 0.06-0.37 |
| Levet vs Pheny+Valpro | 0.14 | 0.05-0.41 |
| Levet vs Topir | 0.43 | 0.22-0.83 |
| Levet vs Valpro | 0.23 | 0.13-0.37 |
| Oxcar vs Carbam+Clonaz | 0.12 | 0.04-0.41 |
| Oxcar vs Carbam+Pheno | 0.36 | 0.15-0.86 |
| Oxcar vs Carbam+Pheny | 0.28 | 0.12-0.69 |
| Oxcar vs Carbam+Valpro | 0.16 | 0.07-0.37 |
| Oxcar vs Clonaz+Valpro | 0.08 | 0.01-0.94 |
| Oxcar vs Pheno+Pheny+Primid | 0.10 | 0.04-0.28 |
| Oxcar vs Pheno+Primid | 0.09 | 0.03-0.29 |
| Oxcar vs Pheno+Valpro | 0.19 | 0.06-0.55 |
| Oxcar vs Pheny+Primid | 0.21 | 0.08-0.63 |
| Oxcar vs Pheny+Valpro | 0.22 | 0.07-0.68 |
| Oxcar vs Valpro | 0.34 | 0.17-0.64 |
| Pheno vs Control | 1.81 | 1.32-2.44 |
| Pheno+Pheny vs Control | 2.13 | 1.47-3.10 |
| Pheno+Pheny+Primid vs Carbam | 7.36 | 3.43-15.55 |
| Pheno+Pheny+Primid vs Carbam+Pheno | 3.52 | 1.38-9.26 |
| Pheno+Pheny+Primid vs Carbam+Pheny | 2.77 | 1.14-6.58 |
| Pheno+Pheny+Primid vs Control | 10.35 | 4.76-22.08 |
| Pheno+Pheny+Primid vs Ethos | 3.17 | 1.02-12.39 |
| Pheno+Pheny+Primid vs Pheno | 5.74 | 2.58-12.22 |
| Pheno+Pheny+Primid vs Pheno+Pheny | 4.79 | 2.26-10.32 |
| Pheno+Pheny+Primid vs Pheny | 6.42 | 2.94-13.46 |
| Pheno+Pheny+Primid vs Primid | 9.07 | 3.42-22.79 |
| Pheno+Primid vs Carbam | 8.23 | 3.25-18.96 |
| Pheno+Primid vs Carbam+Pheno | 3.9 | 1.32-11.12 |
| Pheno+Primid vs Carbam+Pheno+Pheny | 5.02 | 1.07-32.05 |
| Pheno+Primid vs Carbam+Pheny | 3.09 | 1.09-7.95 |
| Pheno+Primid vs Control | 11.47 | 4.52-26.42 |
| Pheno+Primid vs Pheno | 6.34 | 2.46-14.68 |
| Pheno+Primid vs Pheno+Pheny | 5.44 | 2.06-12.45 |
| Pheno+Primid vs Pheny | 7.11 | 2.77-16.61 |
| Pheno+Primid vs Primid | 9.80 | 3.60-26.16 |
| Pheno+Valpro vs Carbam | 3.89 | 1.71-9.10 |
| Pheno+Valpro vs Clonaz | 5.98 | 2.00-20.02 |
| Pheno+Valpro vs Control | 5.38 | 2.33-13.02 |
| Pheno+Valpro vs Pheno | 2.99 | 1.30-6.96 |
| Pheno+Valpro vs Pheno+Pheny | 2.55 | 1.07-6.15 |
| Pheno+Valpro vs Pheny | 3.36 | 1.42-7.86 |
| Pheno+Valpro vs Primid | 4.72 | 1.77-13.28 |
| Pheny vs Control | 1.61 | 1.25-2.13 |
| Pheny+Primid vs Carbam | 3.63 | 1.48-7.34 |
| Pheny+Primid vs Control | 5.06 | 2.12-10.31 |
| Pheny+Primid vs Pheno | 2.80 | 1.17-5.80 |
| Pheny+Primid vs Pheny | 3.12 | 1.30-6.36 |
| Pheny+Primid vs Primid | 4.41 | 1.52-10.62 |
| Pheny+Valpro vs Carbam | 3.50 | 1.42-9.04 |
| Pheny+Valpro vs Clonaz | 5.50 | 1.62-17.65 |
| Pheny+Valpro vs Control | 4.88 | 2.00-12.44 |
| Pheny+Valpro vs Pheno | 2.71 | 1.03-7.01 |
| Pheny+Valpro vs Pheny | 2.99 | 1.23-7.92 |
| Pheny+Valpro vs Primid | 4.30 | 1.41-12.67 |
| Primid vs Carbam+Pheno | 0.39 | 0.17-0.95 |
| Primid vs Carbam+Pheny | 0.31 | 0.14-0.67 |
| Primid+Valpro vs Pheno+Pheny+Primid | 0.12 | 0.00-0.77 |
| Primid+Valpro vs Pheno+Primid | 0.11 | 0.00-0.76 |
| Topir vs Carbam+Clonaz | 0.19 | 0.07-0.60 |
| Topir vs Carbam+Pheny | 0.45 | 0.22-0.91 |
| Topir vs Carbam+Valpro | 0.26 | 0.12-0.54 |
| Topir vs Lamot | 1.75 | 1.01-2.90 |
| Topir vs Pheno+Pheny+Primid | 0.16 | 0.07-0.38 |
| Topir vs Pheno+Primid | 0.15 | 0.06-0.41 |
| Topir vs Pheno+Valpro | 0.31 | 0.12-0.77 |
| Topir vs Pheny+Primid | 0.34 | 0.13-0.89 |
| Topir vs Pheny+Valpro | 0.34 | 0.12-1.00 |
| Topir vs Valpro | 0.54 | 0.32-0.87 |
| Valpro vs Carbam | 2.24 | 1.83-2.75 |
| Valpro vs Carbam+Clonaz | 0.35 | 0.15-0.97 |
| Valpro vs Clonaz | 3.41 | 1.70-7.70 |
| Valpro vs Control | 3.10 | 2.51-3.90 |
| Valpro vs Pheno | 1.72 | 1.30-2.30 |
| Valpro vs Pheno+Pheny | 1.45 | 1.01-2.12 |
| Valpro vs Pheno+Pheny+Primid | 0.30 | 0.14-0.66 |
| Valpro vs Pheno+Primid | 0.27 | 0.12-0.70 |
| Valpro vs Pheny | 1.94 | 1.49-2.47 |
| Valpro vs Primid | 2.68 | 1.48-5.17 |
| *Common within-network between-study variance* | *0.03* | *0.00-0.13* |
| **Old Treatments compared only – 53 studies, 6982 cases, 31 treatments** | | |
| Carbam vs Pheno+Pheny | 0.64 | 0.42-0.98 |
| Carbam+Clonaz vs Carbam | 6.23 | 1.49-20.58 |
| Carbam+Clonaz vs Control | 9.13 | 2.12-31.92 |
| Carbam+Clonaz vs Pheno | 5.54 | 1.32-18.43 |
| Carbam+Clonaz vs Pheny | 5.11 | 1.23-16.94 |
| Carbam+Clonaz vs Primid | 9.10 | 1.93-34.78 |
| Carbam+Pheno vs Control | 3.01 | 1.35-6.53 |
| Carbam+Pheny vs Carbam | 2.69 | 1.45-4.95 |
| Carbam+Pheny vs Control | 3.95 | 2.05-7.45 |
| Carbam+Pheny vs Pheno | 2.37 | 1.23-4.49 |
| Carbam+Pheny vs Pheny | 2.20 | 1.19-4.03 |
| Carbam+Valpro vs Carbam | 6.09 | 2.87-12.32 |
| Carbam+Valpro vs Carbam+Pheno | 2.94 | 1.11-7.62 |
| Carbam+Valpro vs Clonaz | 9.26 | 1.22-426.20 |
| Carbam+Valpro vs Control | 8.88 | 4.12-18.75 |
| Carbam+Valpro vs Pheno | 5.35 | 2.48-11.20 |
| Carbam+Valpro vs Pheno+Pheny | 3.93 | 1.75-8.21 |
| Carbam+Valpro vs Pheny | 4.97 | 2.33-10.14 |
| Carbam+Valpro vs Primid | 8.84 | 3.37-23.16 |
| Carbam+Valpro vs Valpro | 3.08 | 1.42-6.17 |
| Clonaz vs Pheno+Pheny+Primid | 0.09 | 0.00-0.68 |
| Clonaz vs Pheno+Primid | 0.07 | 0.00-0.79 |
| Pheno vs Control | 1.67 | 1.09-2.49 |
| Pheno+Pheny vs Control | 2.28 | 1.44-3.62 |
| Pheno+Pheny+Primid vs Carbam | 7.51 | 3.25-17.33 |
| Pheno+Pheny+Primid vs Carbam+Pheno | 3.65 | 1.27-10.11 |
| Pheno+Pheny+Primid vs Carbam+Pheny | 2.77 | 1.08-7.21 |
| Pheno+Pheny+Primid vs Control | 10.94 | 4.66-25.46 |
| Pheno+Pheny+Primid vs Pheno | 6.59 | 2.84-15.02 |
| Pheno+Pheny+Primid vs Pheno+Pheny | 4.81 | 2.13-10.81 |
| Pheno+Pheny+Primid vs Pheny | 6.11 | 2.64-13.93 |
| Pheno+Pheny+Primid vs Primid | 10.83 | 3.80-31.05 |
| Pheno+Primid vs Carbam | 8.71 | 2.41-28.85 |
| Pheno+Primid vs Carbam+Pheno | 4.26 | 1.08-16.24 |
| Pheno+Primid vs Control | 12.83 | 3.40-44.02 |
| Pheno+Primid vs Pheno | 7.67 | 2.13-24.97 |
| Pheno+Primid vs Pheno+Pheny | 5.56 | 1.59-19.03 |
| Pheno+Primid vs Pheny | 7.06 | 2.00-23.88 |
| Pheno+Primid vs Primid | 12.66 | 3.20-48.48 |
| Pheno+Valpro vs Carbam | 3.62 | 1.43-8.54 |
| Pheno+Valpro vs Control | 5.33 | 2.06-12.63 |
| Pheno+Valpro vs Pheno | 3.21 | 1.24-7.64 |
| Pheno+Valpro vs Pheny | 2.97 | 1.17-6.99 |
| Pheno+Valpro vs Primid | 5.34 | 1.62-15.72 |
| Pheny vs Control | 1.80 | 1.20-2.64 |
| Pheny+Primid vs Carbam | 3.45 | 1.53-7.45 |
| Pheny+Primid vs Control | 5.07 | 2.20-11.35 |
| Pheny+Primid vs Pheno | 3.06 | 1.32-6.68 |
| Pheny+Primid vs Pheny | 2.83 | 1.24-6.09 |
| Pheny+Primid vs Primid | 5.01 | 1.83-13.47 |
| Pheny+Valpro vs Carbam | 3.54 | 1.25-9.18 |
| Pheny+Valpro vs Control | 5.13 | 1.78-14.26 |
| Pheny+Valpro vs Pheno | 3.13 | 1.11-8.53 |
| Pheny+Valpro vs Pheny | 2.88 | 1.04-7.53 |
| Pheny+Valpro vs Primid | 5.21 | 1.54-16.29 |
| Primid vs Carbam+Pheno | 0.33 | 0.13-0.88 |
| Primid vs Carbam+Pheny | 0.26 | 0.10-0.63 |
| Primid vs Pheno+Pheny | 0.45 | 0.20-0.92 |
| Primid+Valpro vs Pheno+Pheny+Primid | 0.11 | 0.00-0.93 |
| Valpro vs Carbam | 1.99 | 1.40-2.85 |
| Valpro vs Control | 2.93 | 1.91-4.47 |
| Valpro vs Pheno | 1.77 | 1.17-2.60 |
| Valpro vs Pheno+Pheny+Primid | 0.27 | 0.12-0.63 |
| Valpro vs Pheno+Primid | 0.23 | 0.07-0.83 |
| Valpro vs Pheny | 1.63 | 1.11-2.39 |
| Valpro vs Primid | 2.87 | 1.42-6.10 |
| *Common within-network between-study variance* | *0.08* | *0.00-0.27* |
| **Alcohol Use – 5 studies, 5212 cases, 16 treatments** | | |
| Gabap vs Carbam | 0.12 | 0.00-0.88 |
| Gabap vs Pheno | 0.08 | 0.00-0.64 |
| Gabap vs Pheno+Valpro | 0.01 | 0.00-0.54 |
| Gabap vs Valpro | 0.06 | 0.00-0.46 |
| Lamot vs Pheno | 0.33 | 0.10-0.91 |
| Lamot vs Valpro | 0.24 | 0.08-0.63 |
| Valpro vs Pheny | 3.43 | 1.19-11.59 |
| *Common within-network between-study variance* | *0.20* | *0.00-1.49* |
| **Large Studies (Study size > 300) – 13 studies, 27227 cases, 22 treatments** | | |
| Carbam vs Control | 1.38 | 1.03-1.98 |
| Carbam+Pheny+Valpro vs Carbam | 15.18 | 1.25-103.80 |
| Carbam+Pheny+Valpro vs Clonaz | 17.45 | 1.40-136.60 |
| Carbam+Pheny+Valpro vs Control | 20.77 | 1.72-154.20 |
| Carbam+Pheny+Valpro vs Pheno+Pheny | 21.79 | 1.20-453.60 |
| Carbam+Valpro vs Control | 4.09 | 1.19-11.94 |
| Clonaz vs Pheno+Primid | 0.13 | 0.03-0.77 |
| Gabap vs Carbam | 0.44 | 0.16-0.97 |
| Gabap vs Carbam+Clonaz | 0.07 | 0.01-0.89 |
| Gabap vs Carbam+Pheny+Valpro | 0.03 | 0.00-0.39 |
| Gabap vs Carbam+Valpro | 0.15 | 0.04-0.62 |
| Gabap vs Clonaz+Valpro | 0.05 | 0.01-0.84 |
| Gabap vs Ethos | 0.20 | 0.05-0.87 |
| Gabap vs Pheno | 0.32 | 0.11-0.75 |
| Gabap vs Pheno+Primid | 0.06 | 0.01-0.43 |
| Gabap vs Pheny | 0.33 | 0.12-0.77 |
| Gabap vs Valpro | 0.19 | 0.06-0.41 |
| Lamot vs Carbam | 0.77 | 0.57-0.99 |
| Lamot vs Carbam+Pheny+Valpro | 0.05 | 0.01-0.61 |
| Lamot vs Carbam+Valpro | 0.26 | 0.09-0.88 |
| Lamot vs Pheno | 0.55 | 0.34-0.85 |
| Lamot vs Pheno+Primid | 0.11 | 0.03-0.64 |
| Lamot vs Pheny | 0.58 | 0.39-0.84 |
| Lamot vs Valpro | 0.32 | 0.24-0.41 |
| Levet vs Carbam | 0.55 | 0.32-0.87 |
| Levet vs Carbam+Clonaz | 0.08 | 0.01-0.98 |
| Levet vs Carbam+Pheny+Valpro | 0.04 | 0.00-0.47 |
| Levet vs Carbam+Valpro | 0.18 | 0.06-0.67 |
| Levet vs Clonaz+Valpro | 0.06 | 0.01-0.91 |
| Levet vs Ethos | 0.24 | 0.08-0.98 |
| Levet vs Oxcar | 0.44 | 0.21-0.94 |
| Levet vs Pheno | 0.39 | 0.20-0.71 |
| Levet vs Pheno+Primid | 0.08 | 0.02-0.48 |
| Levet vs Pheny | 0.41 | 0.23-0.71 |
| Levet vs Topir | 0.36 | 0.19-0.69 |
| Levet vs Valpro | 0.23 | 0.13-0.37 |
| Oxcar vs Gabap | 2.84 | 1.05-9.00 |
| Oxcar vs Valpro | 0.52 | 0.27-0.90 |
| Pheno vs Control | 1.95 | 1.21-3.16 |
| Pheno+Primid vs Carbam | 6.86 | 1.21-25.19 |
| Pheno+Primid vs Control | 9.48 | 1.69-36.81 |
| Pheno+Primid vs Pheno+Pheny | 9.83 | 1.07-129.00 |
| Pheny vs Control | 1.83 | 1.23-2.81 |
| Topir vs Control | 2.07 | 1.24-3.43 |
| Topir vs Gabap | 3.36 | 1.38-9.92 |
| Topir vs Lamot | 1.96 | 1.18-3.09 |
| Topir vs Valpro | 0.62 | 0.39-0.97 |
| Valpro vs Carbam | 2.40 | 1.86-3.02 |
| Valpro vs Clonaz | 2.74 | 1.54-5.51 |
| Valpro vs Control | 3.31 | 2.49-4.64 |
| Valpro vs Pheno | 1.71 | 1.11-2.62 |
| Valpro vs Pheny | 1.81 | 1.28-2.58 |
| *Common within-network between-study variance* | *0.03* | *0.00-0.17* |
| **Studies with Folic Acid use in 50% of patients – 5 studies, 10825 cases, 15 treatments** | | |
| Gabap vs Pheno | 0.08 | 0.00-0.57 |
| Gabap vs Valpro | 0.06 | 0.00-0.38 |
| Lamot vs Valpro | 0.32 | 0.17-0.65 |
| Lamot+Valpro vs Gabap | 35.6 | 1.15-1.85 x 10^3^ |
| Levet vs Valpro | 0.28 | 0.12-0.66 |
| Oxcar vs Valpro | 0.24 | 0.07-0.68 |
| Topir vs Gabap | 8.78 | 1.33-244.30 |
| Valpro vs Carbam | 2.72 | 1.38-5.16 |
| Valpro vs Clonaz | 3.80 | 1.27-14.48 |
| Valpro vs Control | 2.86 | 1.18-6.22 |
| Valpro vs Pheny | 2.80 | 1.06-7.54 |
| *Common within-network between-study variance* | *0.09* | *0.00-0.72* |
| **Family History of Congenital Malformations – 2 studies, 5057 cases, 11 treatments** | | |
| Pheno+Valpro vs Pheny | 96.42 | 1.59-6.42 x 10^4^ |
| Valpro vs Pheny | 31.64 | 1.50-1.53 x 10^4^ |
| *Common within-network between-study variance* | *0.23* | *0.00-3.42* |
| **Risk of Bias Comparability of Cohorts (Low) – 10 studies, 21622 cases, 31 treatments** | | |
| Carbam vs Pheno | 0.47 | 0.26-0.88 |
| Carbam+Pheny vs Pheno+Pheny | 0.06 | 0.00-0.89 |
| Carbam+Valpro vs Carbam+Pheny | 34.94 | 2.19-1.88 x 10^4^ |
| Gabap vs Carbam+Valpro | 0.03 | 0.00-0.71 |
| Gabap vs Ethos | 0.18 | 0.04-0.87 |
| Gabap vs Pheno | 0.22 | 0.07-0.60 |
| Gabap vs Pheno+Pheny+Primid | 0.02 | 0.00-0.54 |
| Gabap vs Pheny | 0.35 | 0.12-0.89 |
| Gabap vs Valpro | 0.18 | 0.06-0.41 |
| Lamot vs Pheno | 0.39 | 0.22-0.74 |
| Lamot vs Pheno+Pheny+Primid | 0.04 | 0.00-0.90 |
| Lamot vs Pheny | 0.64 | 0.41-1.00 |
| Lamot vs Valpro | 0.31 | 0.23-0.42 |
| Levet vs Carbam+Valpro | 0.04 | 0.00-0.85 |
| Levet vs Ethos | 0.24 | 0.07-0.98 |
| Levet vs Pheno | 0.29 | 0.14-0.61 |
| Levet vs Pheno+Pheny+Primid | 0.03 | 0.00-0.66 |
| Levet vs Pheny | 0.47 | 0.25-0.89 |
| Levet vs Topir | 0.34 | 0.17-0.66 |
| Levet vs Valpro | 0.23 | 0.13-0.40 |
| Oxcar vs Valpro | 0.43 | 0.21-0.82 |
| Pheno vs Control | 2.22 | 1.13-4.08 |
| Pheno+Pheny+Primid vs Carbam+Pheno | 8.26 | 1.14-170.80 |
| Pheno+Pheny+Primid vs Carbam+Pheny | 40.88 | 2.88-1.85 x 10^4^ |
| Pheno+Valpro vs Carbam+Pheny | 21.12 | 1.06-1.26 x 10^4^ |
| Pheny+Primid vs Carbam+Pheny | 24.31 | 1.55-1.17 x 10^4^ |
| Pheny+Primid+Valpro vs Carbam+Pheny | 31.29 | 1.09-1.85 x 10^4^ |
| Topir vs Carbam | 1.83 | 1.11-2.94 |
| Topir vs Control | 1.90 | 1.10-3.24 |
| Topir vs Gabap | 3.88 | 1.53-12.07 |
| Topir vs Lamot | 2.19 | 1.32-3.56 |
| Valpro vs Carbam | 2.67 | 2.00-3.60 |
| Valpro vs Clonaz | 2.76 | 1.46-5.74 |
| Valpro vs Control | 2.77 | 1.92-4.09 |
| Valpro vs Pheny | 2.04 | 1.35-3.18 |
| *Common within-network between-study variance* | *0.03* | *0.00-0.21* |
| **Risk of Bias Adequacy of Follow-Up of Cohorts (Low) – 35 studies, 20122 cases, 34 treatments** | | |
| Carbam vs Control | 1.54 | 1.18-2.15 |
| Carbam+Lamot vs Pheno+Pheny+Primid | 0.01 | 0.00-0.40 |
| Carbam+Pheno vs Control | 3.19 | 1.13-8.38 |
| Carbam+Pheno+Valpro vs Pheno+Pheny+Primid | 0.03 | 0.00-0.95 |
| Carbam+Pheny vs Carbam | 2.84 | 1.06-6.74 |
| Carbam+Pheny vs Control | 4.35 | 1.68-10.63 |
| Carbam+Pheny vs Pheno | 2.81 | 1.03-7.21 |
| Carbam+Valpro vs Clonaz | 5.83 | 1.45-28.09 |
| Carbam+Valpro vs Control | 4.73 | 1.59-13.12 |
| Clonaz vs Carbam+Pheno | 0.25 | 0.06-0.99 |
| Clonaz vs Carbam+Pheny | 0.19 | 0.04-0.70 |
| Clonaz vs Ethos | 0.20 | 0.04-0.87 |
| Clonaz vs Pheno+Pheny+Primid | 0.03 | 0.00-0.20 |
| Clonaz vs Pheno+Primid | 0.08 | 0.01-0.64 |
| Clonaz vs Pheny | 0.37 | 0.11-0.96 |
| Ethos vs Control | 3.88 | 1.20-11.18 |
| Gabap vs Pheno+Pheny+Primid | 0.12 | 0.01-0.73 |
| Lamot vs Carbam+Pheny | 0.26 | 0.10-0.71 |
| Lamot vs Carbam+Valpro | 0.24 | 0.08-0.75 |
| Lamot vs Ethos | 0.29 | 0.10-0.98 |
| Lamot vs Pheno+Pheny | 0.56 | 0.31-0.99 |
| Lamot vs Pheno+Pheny+Primid | 0.05 | 0.01-0.22 |
| Lamot vs Pheno+Primid | 0.11 | 0.02-0.74 |
| Lamot vs Pheno+Valpro | 0.13 | 0.02-0.96 |
| Lamot vs Pheny | 0.53 | 0.34-0.80 |
| Lamot vs Valpro | 0.36 | 0.25-0.50 |
| Lamot+Levet vs Pheno+Pheny+Primid | 0.02 | 0.00-0.52 |
| Lamot+Valpro vs Carbam+Pheny | 0.04 | 0.00-0.73 |
| Lamot+Valpro vs Carbam+Valpro | 0.04 | 0.00-0.71 |
| Lamot+Valpro vs Ethos | 0.04 | 0.00-0.92 |
| Lamot+Valpro vs Pheno+Pheny+Primid | 0.01 | 0.00-0.18 |
| Lamot+Valpro vs Pheno+Primid | 0.02 | 0.00-0.53 |
| Lamot+Valpro vs Pheno+Valpro | 0.02 | 0.00-0.63 |
| Lamot+Valpro vs Valpro | 0.06 | 0.00-0.89 |
| Levet vs Carbam | 0.33 | 0.13-0.72 |
| Levet vs Carbam+Pheno | 0.16 | 0.04-0.60 |
| Levet vs Carbam+Pheny | 0.11 | 0.03-0.41 |
| Levet vs Carbam+Valpro | 0.11 | 0.03-0.40 |
| Levet vs Ethos | 0.13 | 0.03-0.52 |
| Levet vs Gabap | 0.18 | 0.05-0.75 |
| Levet vs Lamot | 0.45 | 0.17-0.99 |
| Levet vs Pheno | 0.33 | 0.12-0.75 |
| Levet vs Pheno+Pheny | 0.25 | 0.08-0.61 |
| Levet vs Pheno+Pheny+Primid | 0.02 | 0.00-0.12 |
| Levet vs Pheno+Primid | 0.05 | 0.01-0.37 |
| Levet vs Pheno+Valpro | 0.06 | 0.01-0.53 |
| Levet vs Pheny | 0.23 | 0.09-0.54 |
| Levet vs Primid | 0.28 | 0.09-0.85 |
| Levet vs Valpro | 0.16 | 0.06-0.35 |
| Oxcar vs Pheno+Pheny+Primid | 0.07 | 0.01-0.40 |
| Pheno vs Control | 1.56 | 1.02-2.39 |
| Pheno+Pheny vs Control | 2.05 | 1.23-3.44 |
| Pheno+Pheny+Primid vs Carbam | 14.86 | 3.36-97.58 |
| Pheno+Pheny+Primid vs Carbam+Pheno | 7.38 | 1.27-57.94 |
| Pheno+Pheny+Primid vs Carbam+Pheny | 5.52 | 1.01-40.75 |
| Pheno+Pheny+Primid vs Control | 23.29 | 5.25-149.30 |
| Pheno+Pheny+Primid vs Ethos+Pheny | 9.19 | 1.06-117.10 |
| Pheno+Pheny+Primid vs Pheno | 14.91 | 3.30-97.33 |
| Pheno+Pheny+Primid vs Pheno+Pheny | 11.46 | 2.54-70.79 |
| Pheno+Pheny+Primid vs Pheny | 10.79 | 2.40-68.42 |
| Pheno+Pheny+Primid vs Pheny+Primid | 9.63 | 1.33-92.77 |
| Pheno+Pheny+Primid vs Primid | 13.31 | 2.58-92.65 |
| Pheno+Primid vs Carbam | 6.66 | 1.00-39.32 |
| Pheno+Primid vs Control | 10.18 | 1.57-63.81 |
| Pheno+Valpro vs Clonaz | 10.87 | 1.12-88.90 |
| Pheno+Valpro vs Control | 8.53 | 1.19-46.53 |
| Pheny vs Control | 2.16 | 1.53-3.14 |
| Pheny+Valpro vs Pheno+Pheny+Primid | 0.08 | 0.01-0.66 |
| Topir vs Pheno+Pheny+Primid | 0.07 | 0.01-0.36 |
| Valpro vs Carbam | 2.06 | 1.54-2.70 |
| Valpro vs Clonaz | 3.98 | 1.60-13.47 |
| Valpro vs Control | 3.20 | 2.40-4.35 |
| Valpro vs Pheno | 2.04 | 1.33-3.16 |
| Valpro vs Pheno+Pheny+Primid | 0.14 | 0.02-0.61 |
| Valpro vs Pheny | 1.47 | 1.03-2.10 |
| *Common within-network between-study variance* | *0.05* | *0.00-0.22* |

## Appendix N. Network diagrams for NMAs of specific and minor congenital malformations


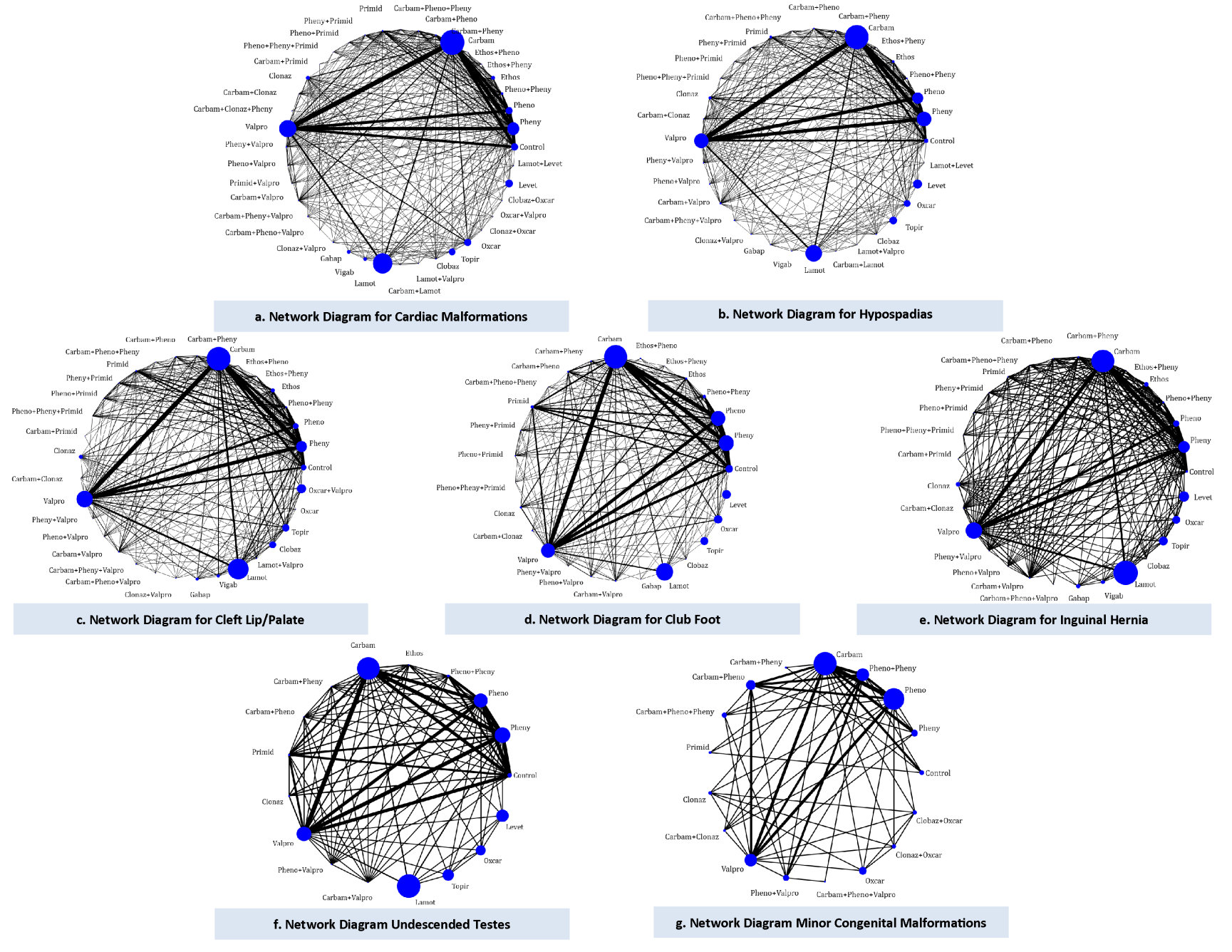

Supplement: Supplementary file 3 — Supplementary Online Content (Appendices A–N). Appendix A. Description of outcomes. Appendix B. List of included articles. Appendix C. Key excluded studies due to only one arm reported with abstractable data. Appendix D. List of studies and their study characteristics. Appendix E. List of studies and their patient characteristics. Appendix F. Risk of bias for randomized controlled trials – Cochrane risk-of-bias tool. Appendix G. Methodological quality of case-control studies – Newcastle-Ottawa Scale. Appendix H. Methodological quality of observational cohort studies – Newcastle-Ottawa Scale. Appendix I. Comparison adjusted funnel plot for each outcome. Appendix J. Statistically significant network meta-analysis results along with meta-analysis results, transitivity, and consistency assessment. Appendix K. Characteristics of the treatment nodes per outcome along with their SUCRA values. Appendix L. Network characteristics per outcome. Appendix M. Meta-regression, subgroup, and sensitivity analyses results. Appendix N. Network diagrams for network meta-analyses of specific and minor congenital malformations. (DOCX 2016 kb) [file 12916_2017_845_MOESM3_ESM.docx]
